# Supplementary material for: Systematic In Silico Assessment of Antimicrobial Resistance Dissemination across the Global Plasmidome
Source: Antibiotics (Basel). 2023 Feb 1;12(2):281. doi: 10.3390/antibiotics12020281 (PMC9951915; doi:10.3390/antibiotics12020281)
Supplement: Supplementary file 1 [file antibiotics-12-00281-s001.zip › FigureS1.pdf]

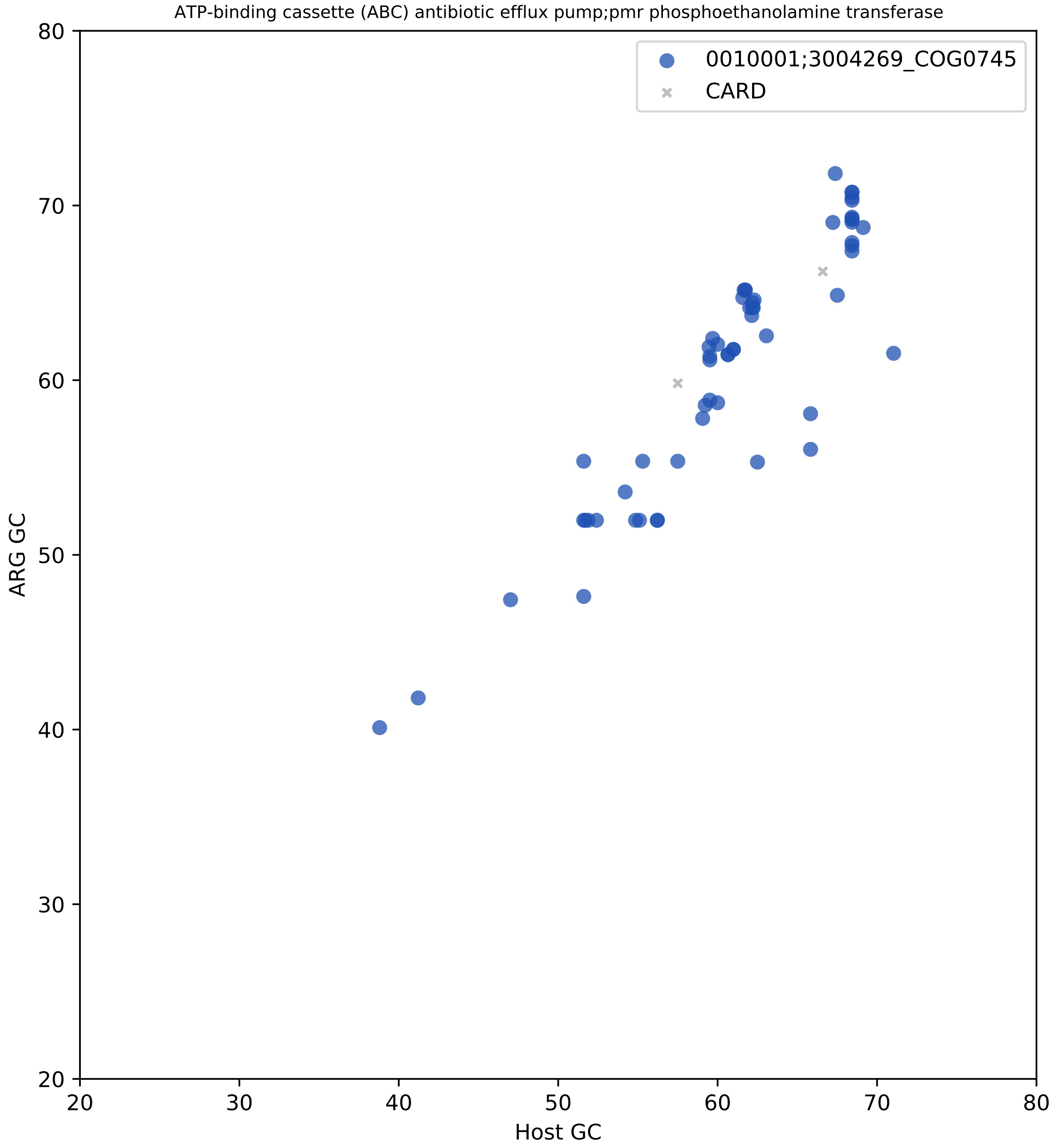

Supplementary Figure S1: Scatter plots depicting the correlation between the %GC content of ARG genes and that of their host chromosome for each ARO-COGs group. Experimentally-validated resistance genes annotated in CARD that map to each ARO-COG group are shown as grey crosses.

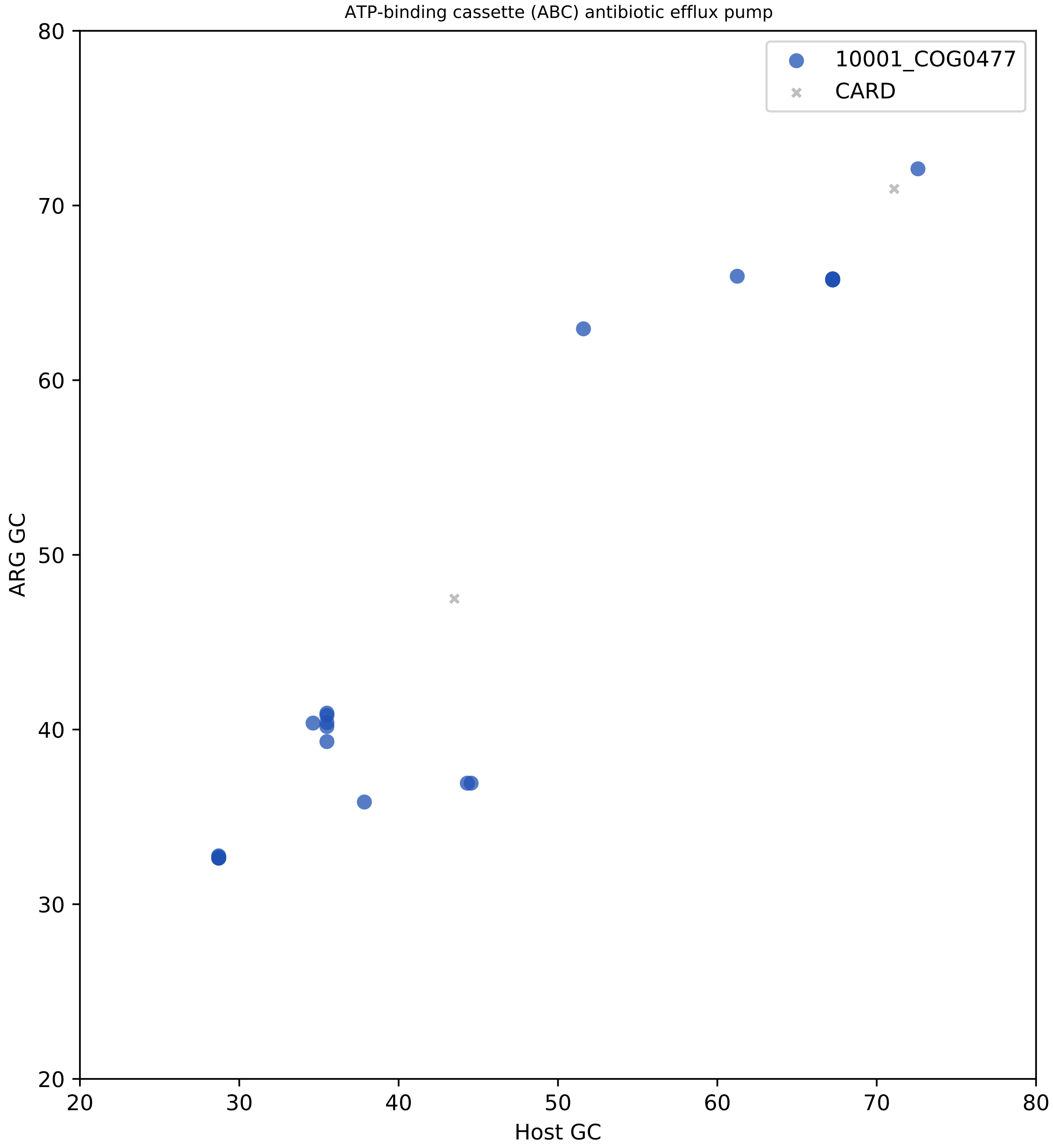

Supplementary Figure S1: (continued).

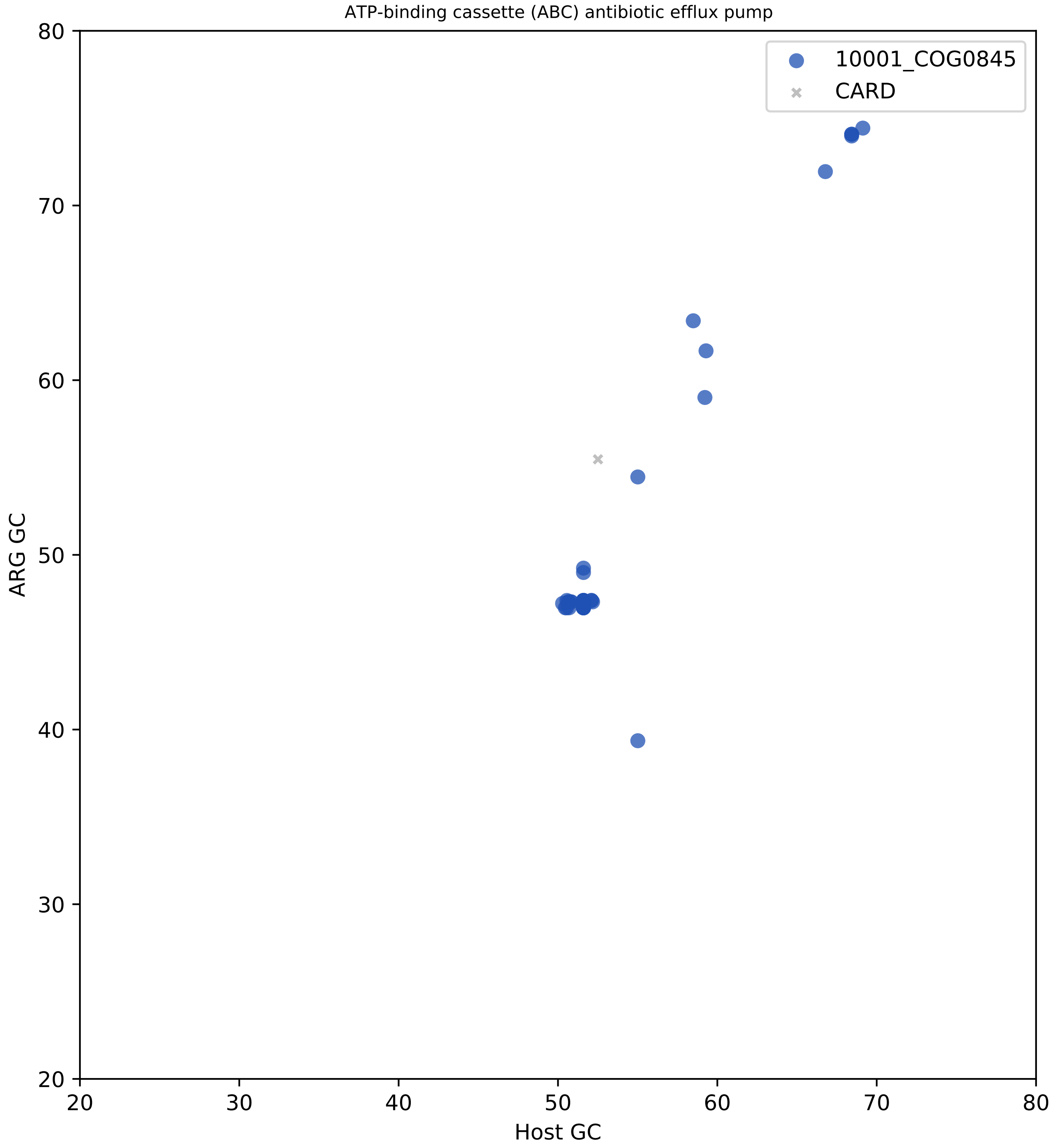

Supplementary Figure S1: (continued).

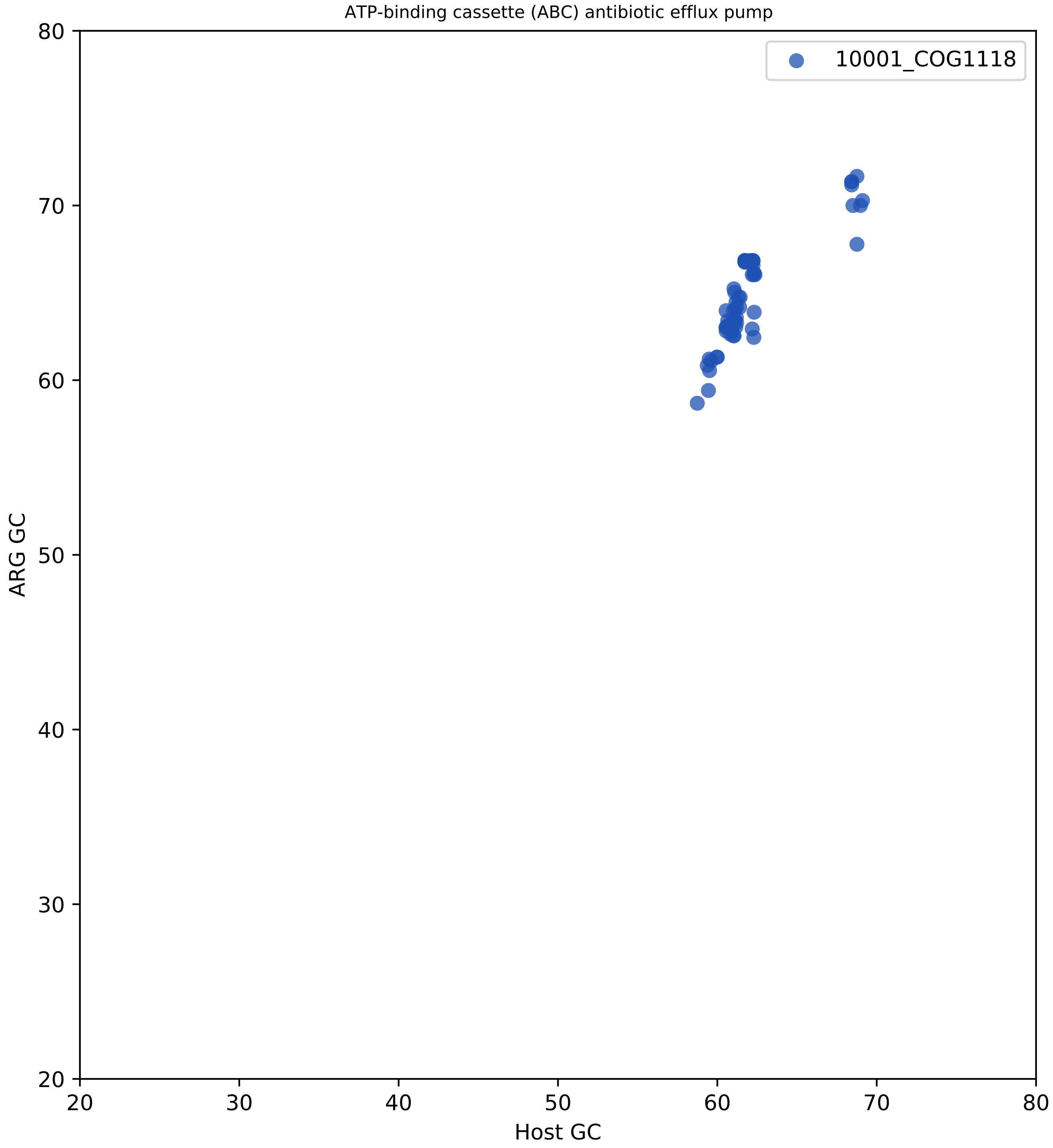

Supplementary Figure S1: (continued).

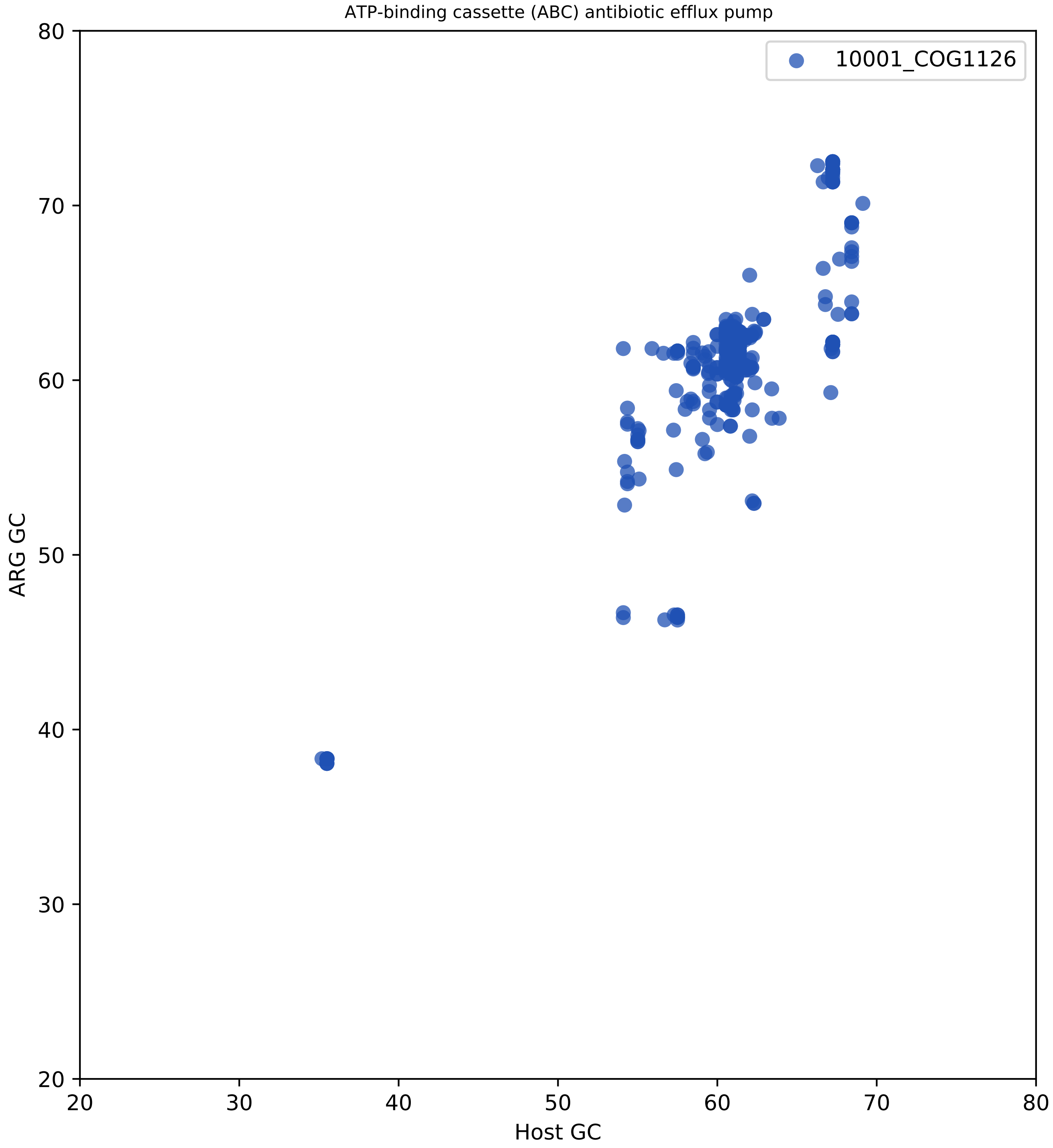

Supplementary Figure S1: (continued).

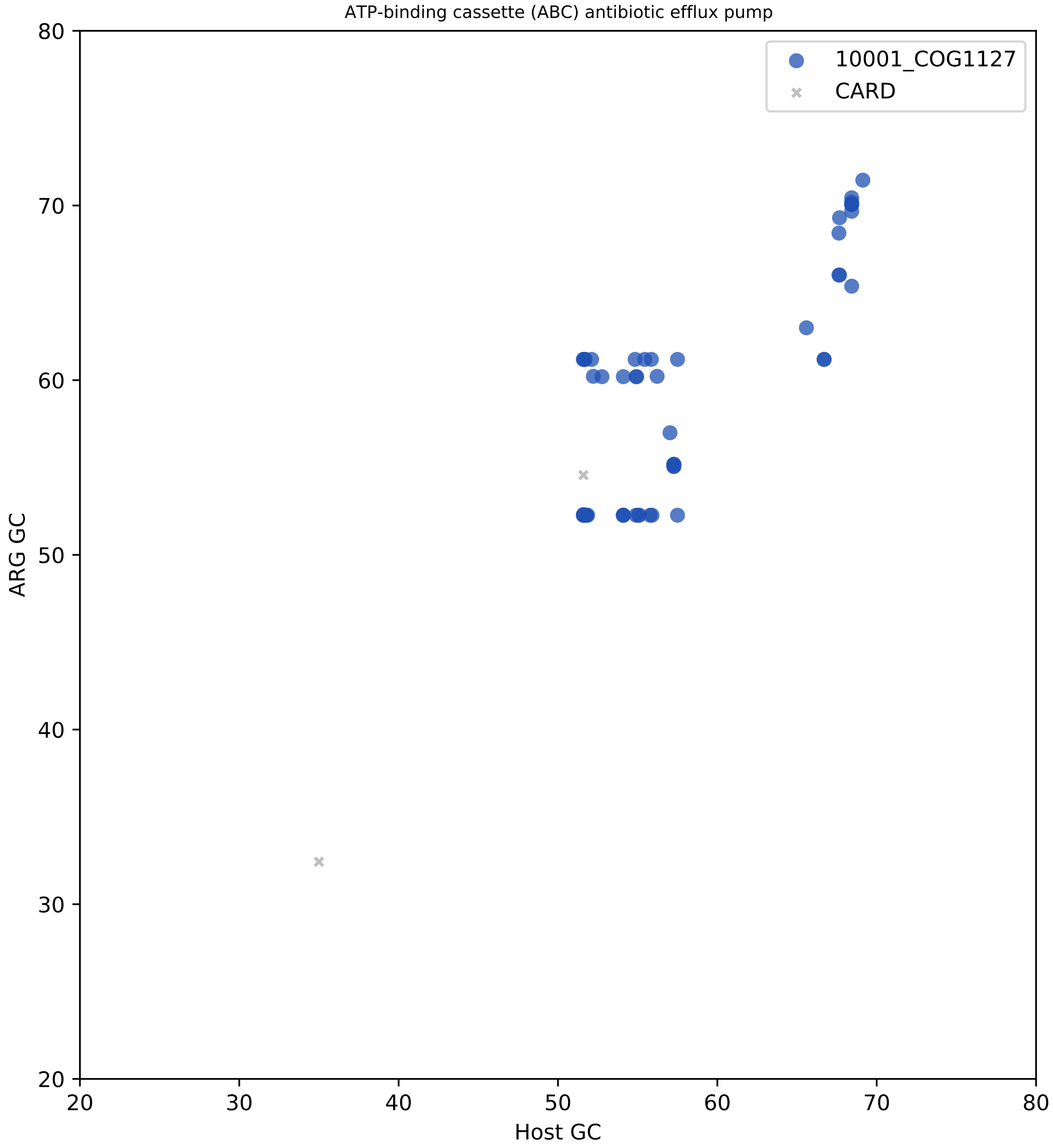

Supplementary Figure S1: (continued).

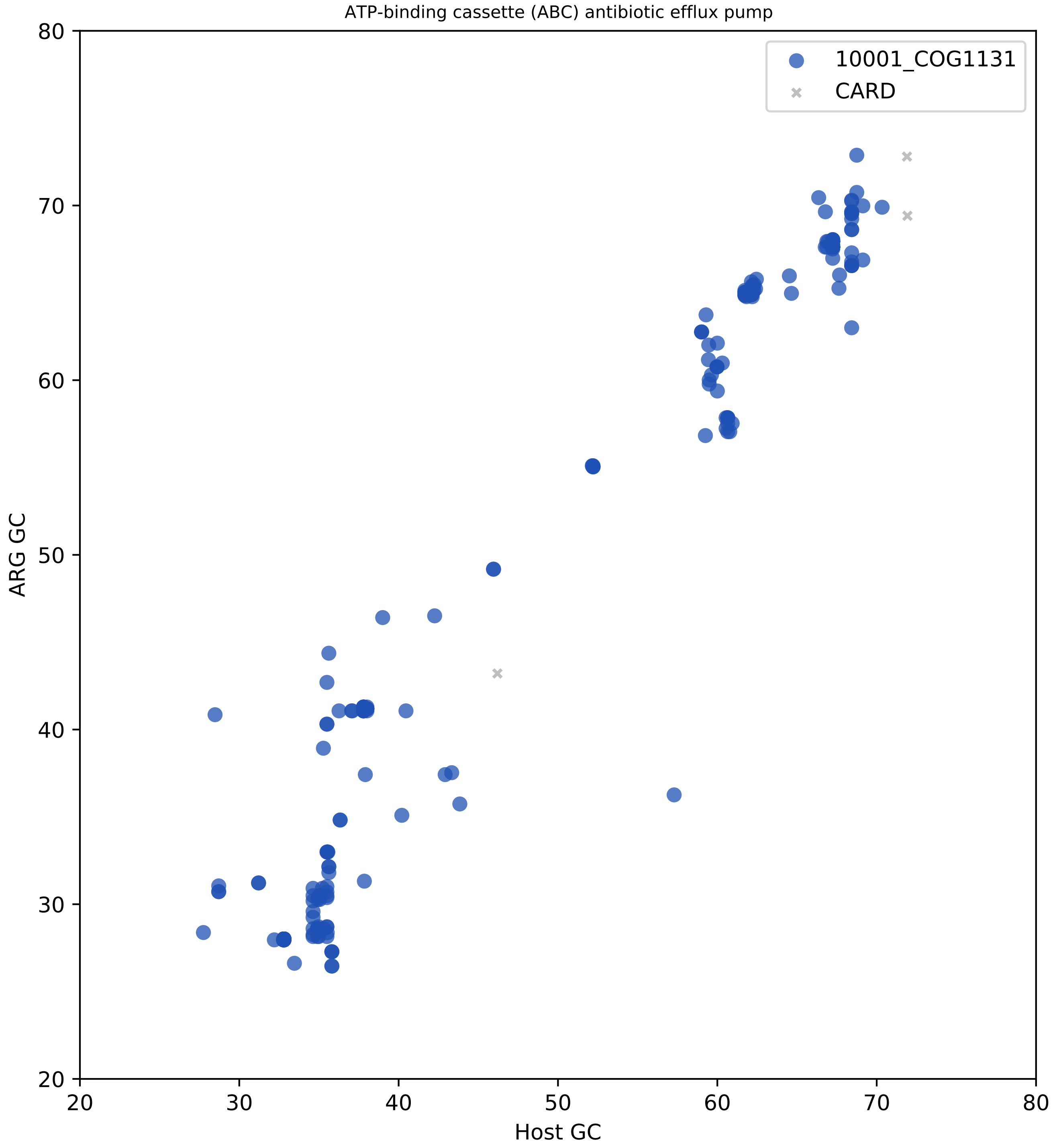

Supplementary Figure S1: (continued).

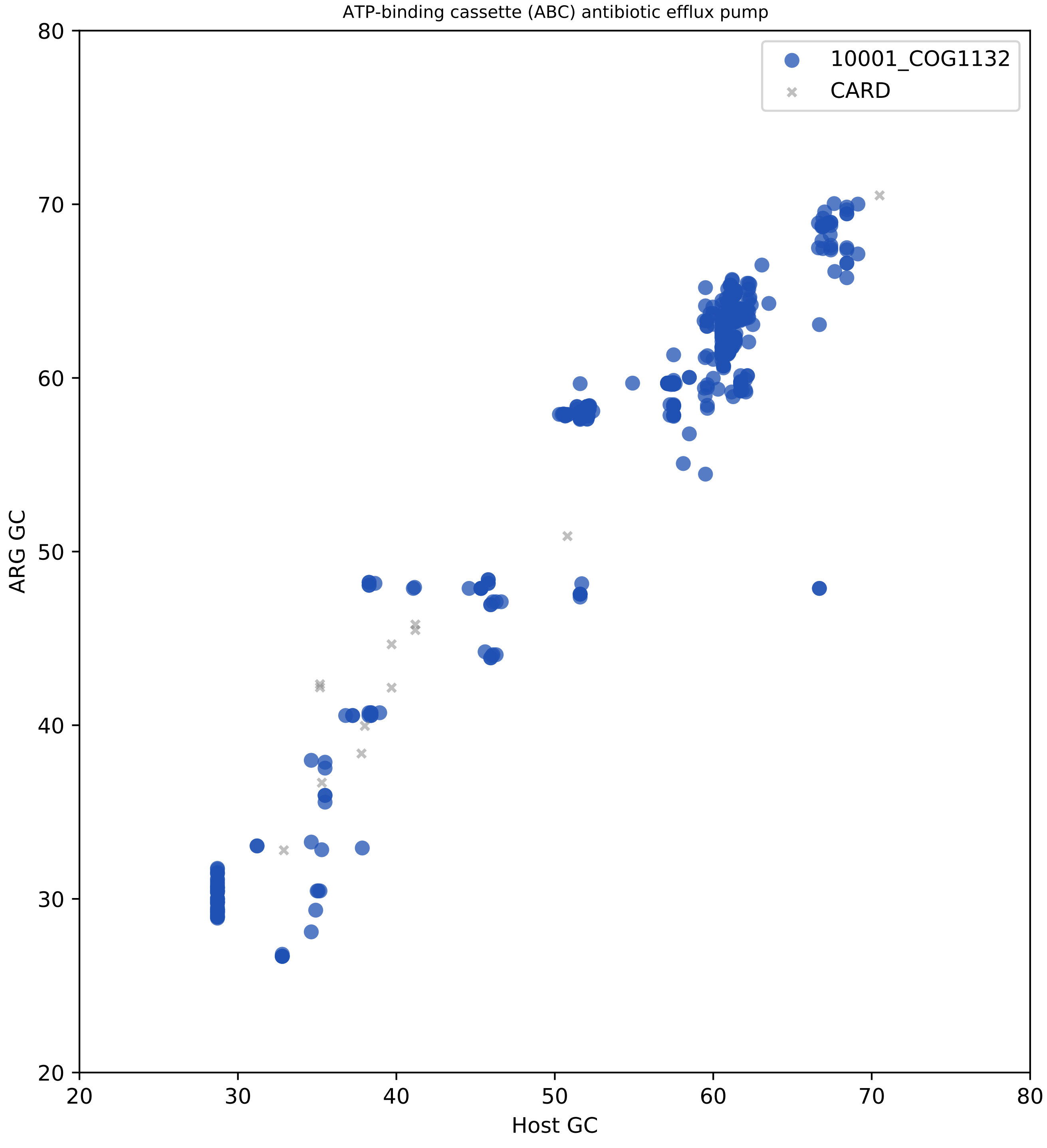

Supplementary Figure S1: (continued).

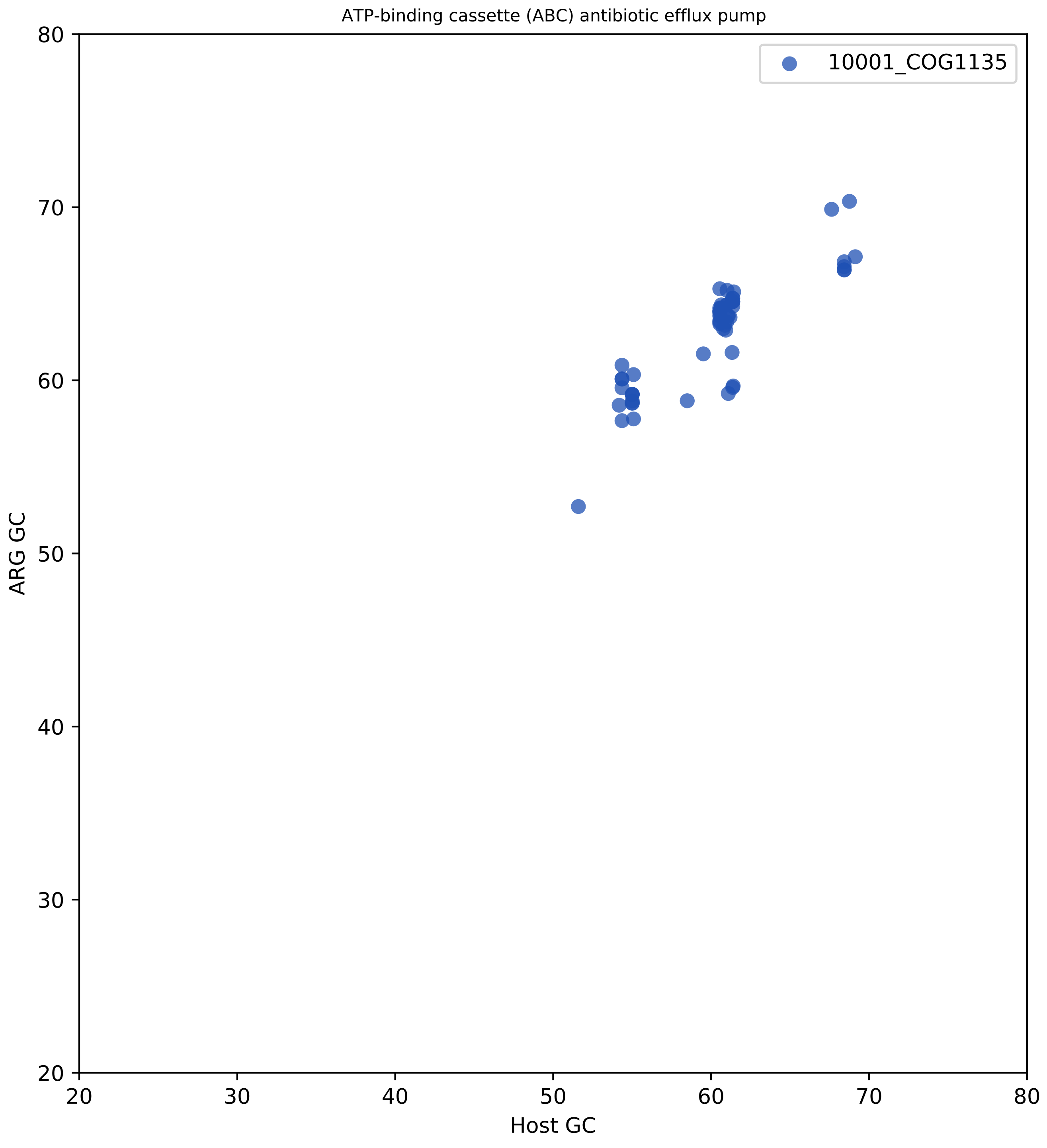

Supplementary Figure S1: (continued).

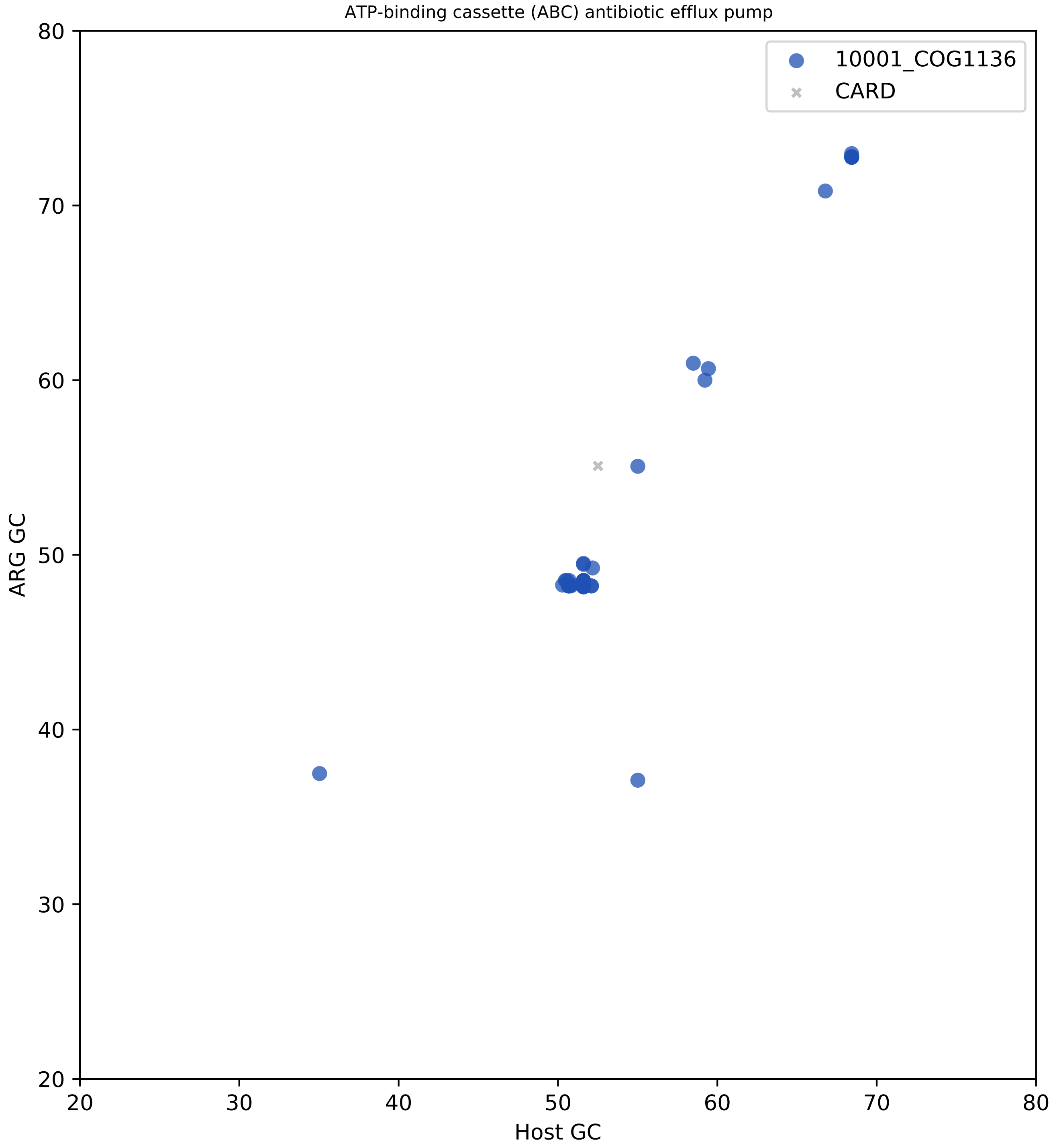

Supplementary Figure S1: (continued).

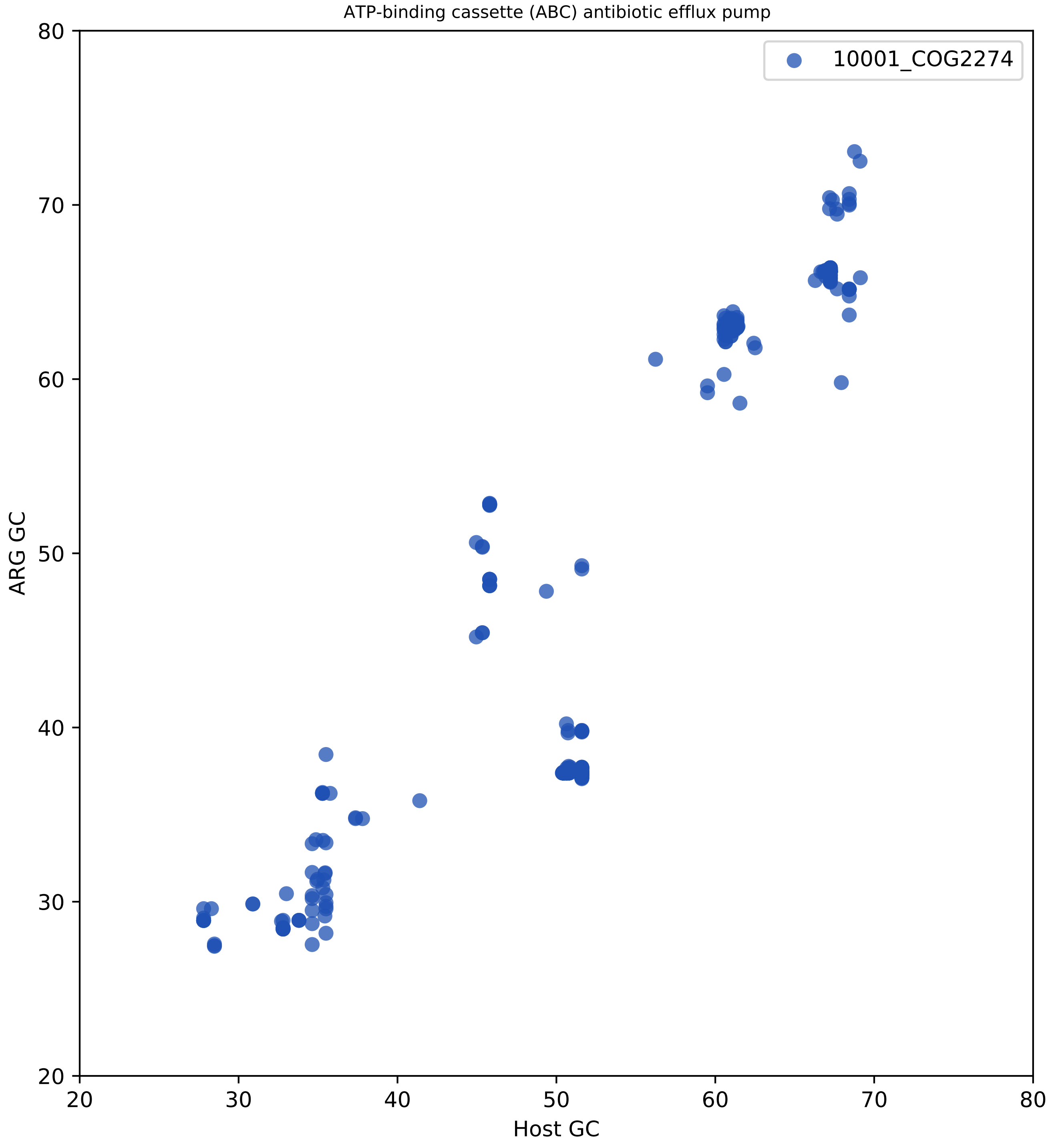

Supplementary Figure S1: (continued).

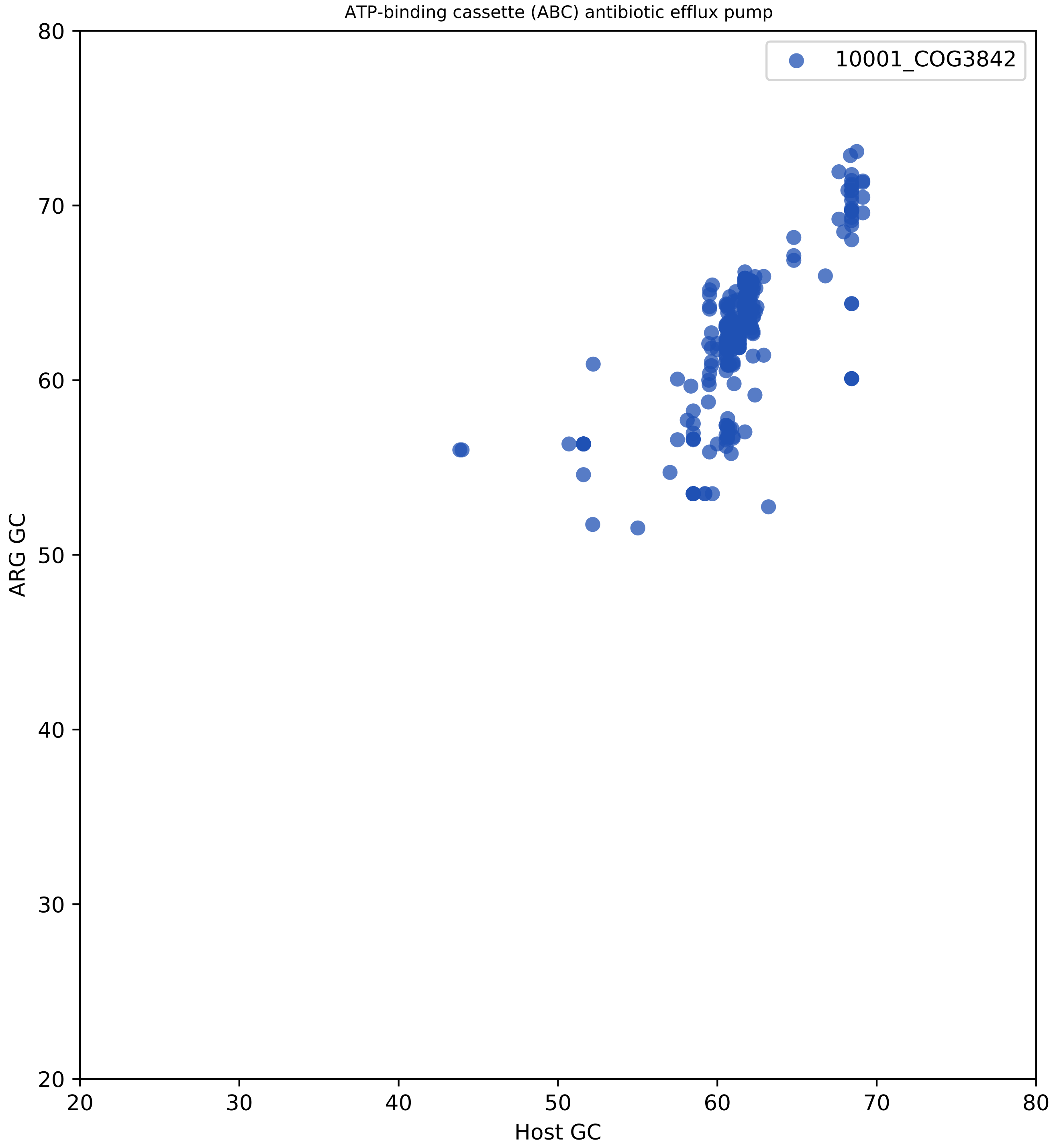

Supplementary Figure S1: (continued).

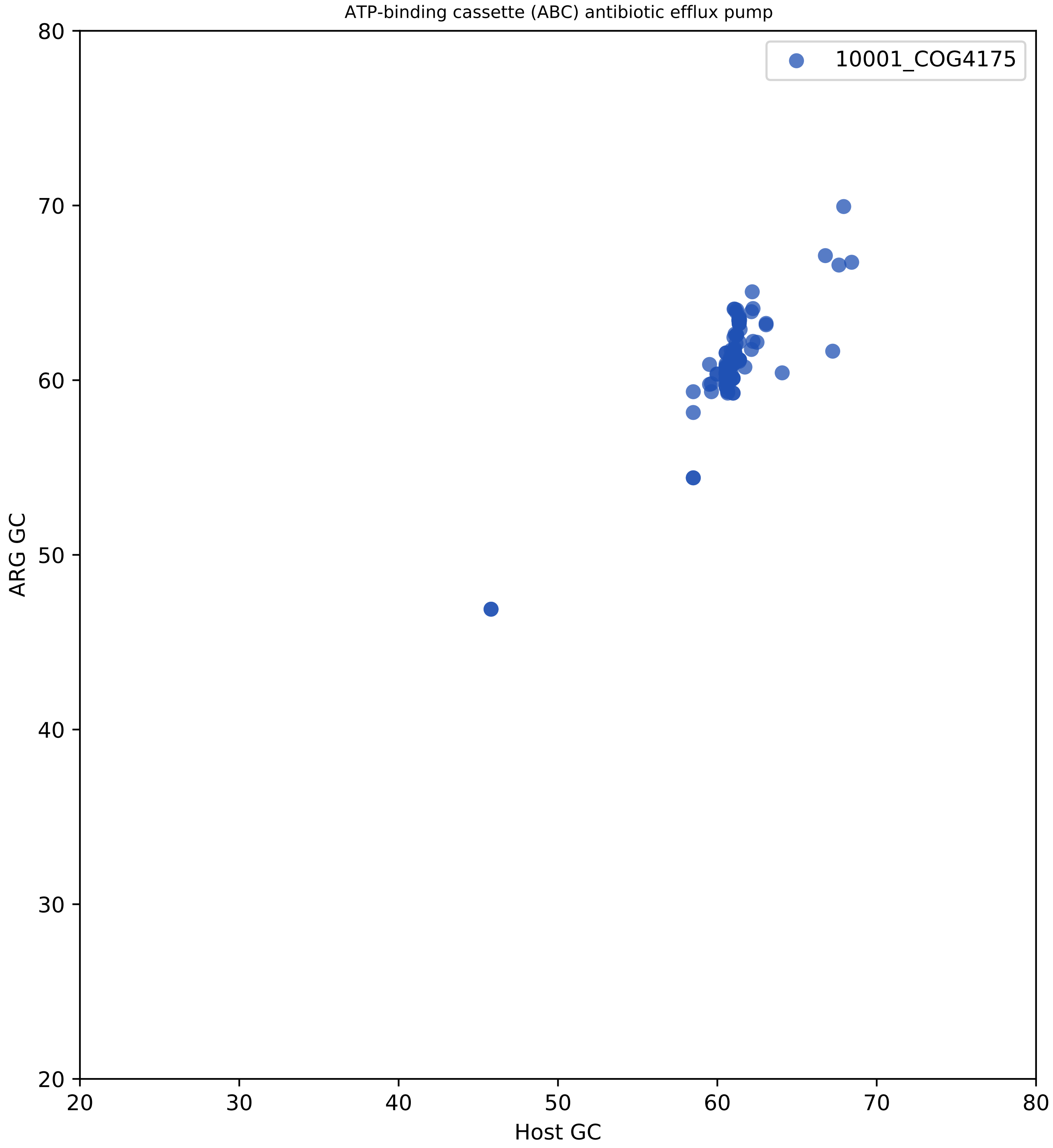

Supplementary Figure S1: (continued).

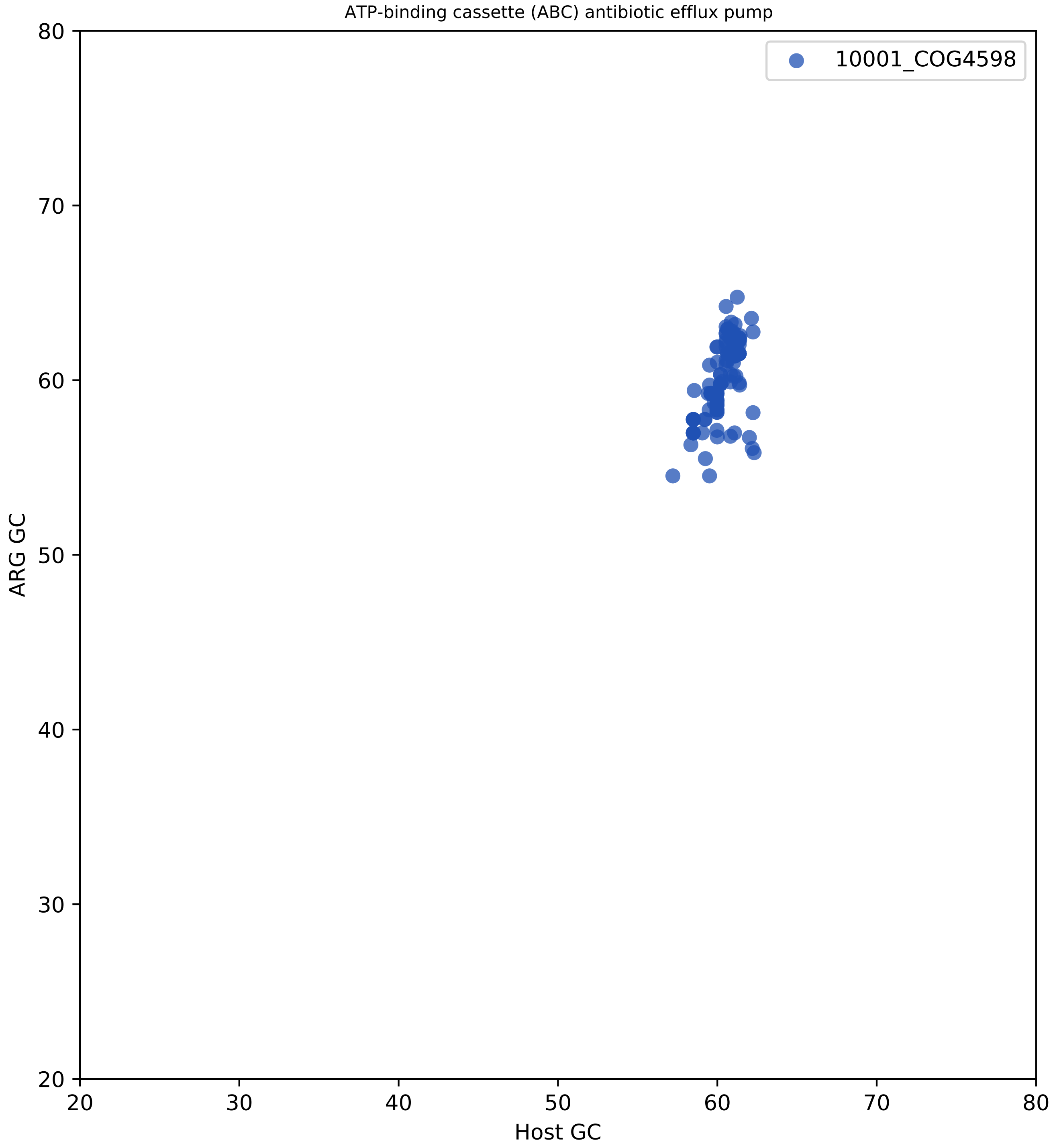

Supplementary Figure S1: (continued).

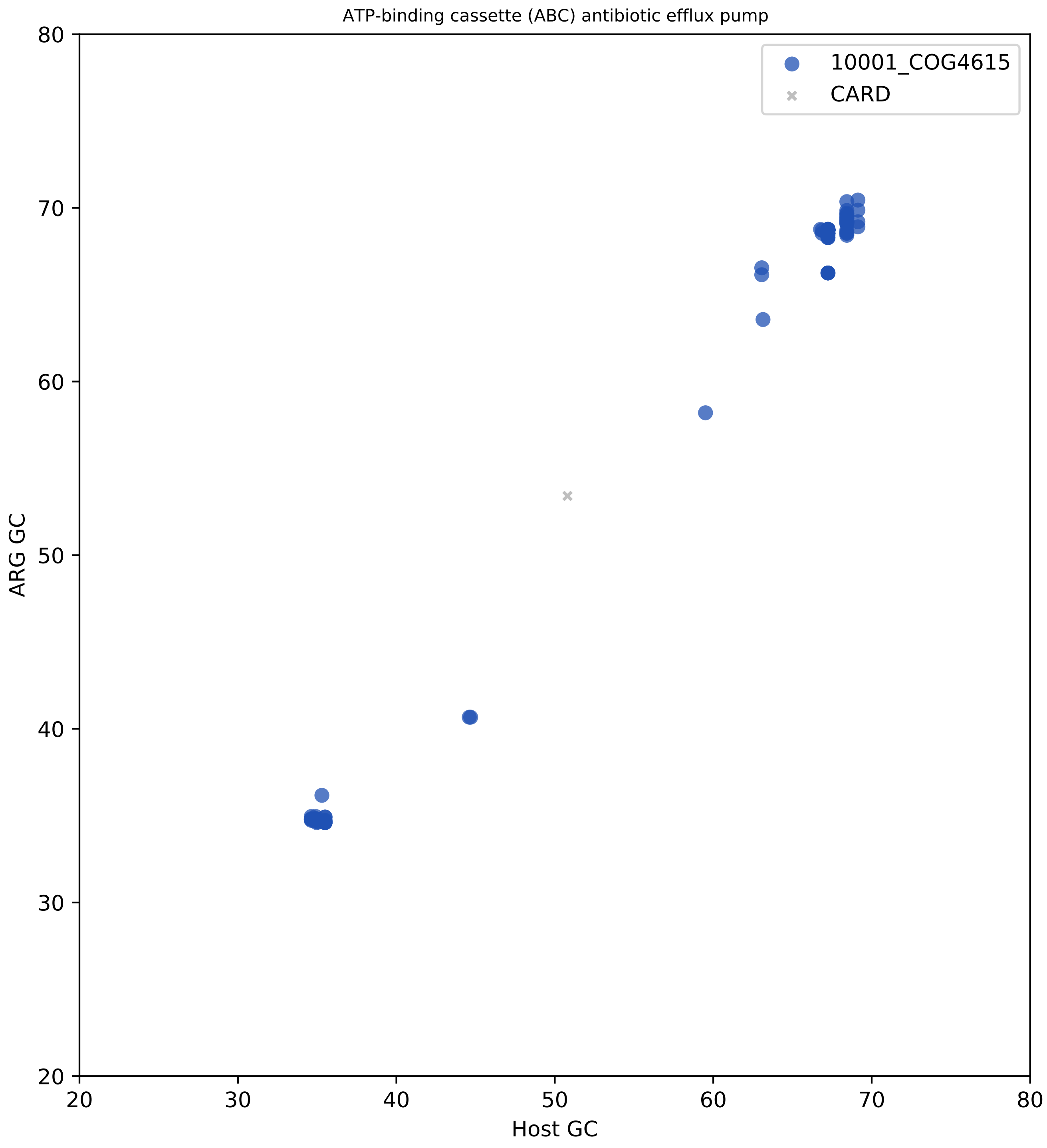

Supplementary Figure S1: (continued).

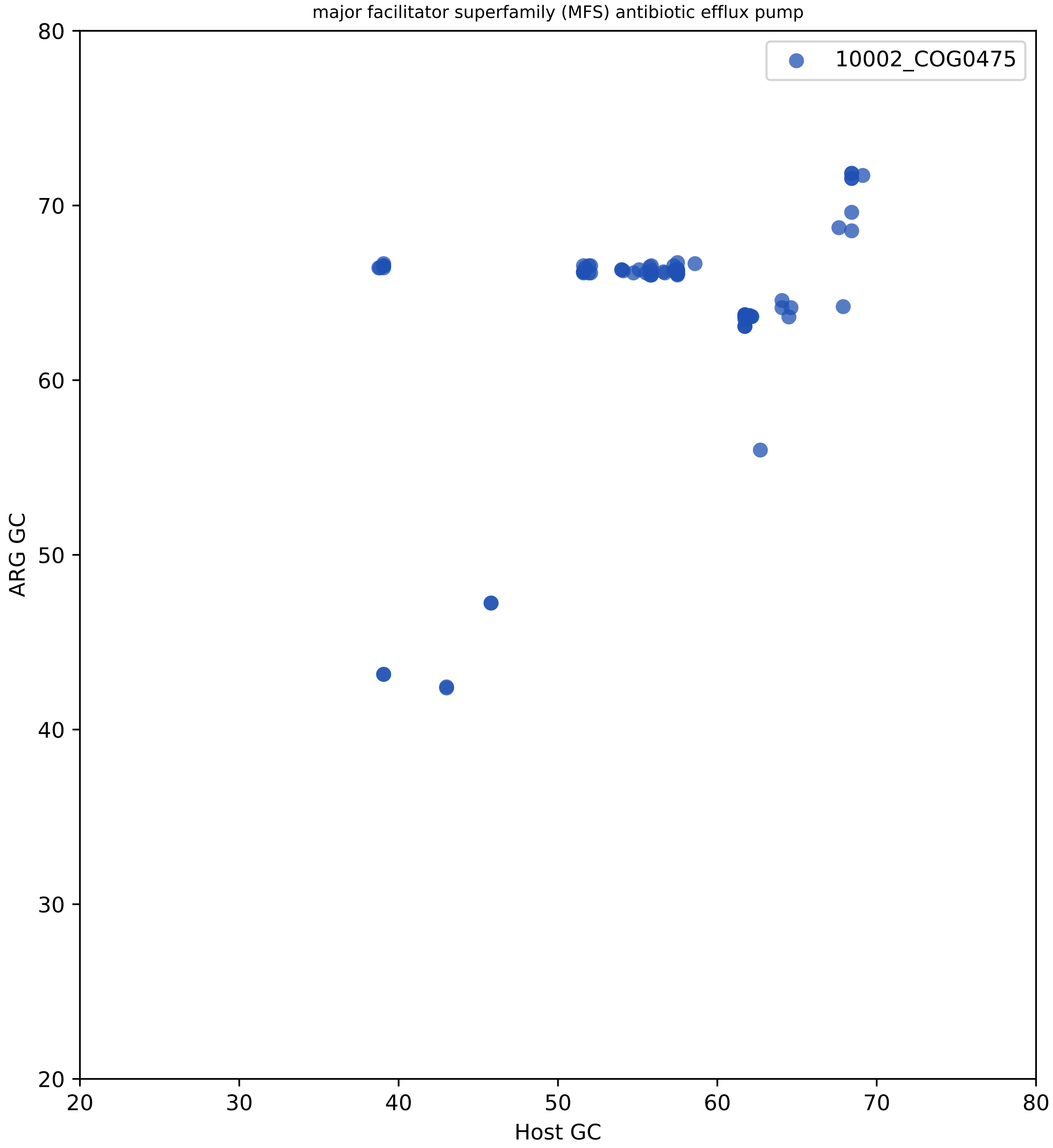

Supplementary Figure S1: (continued).

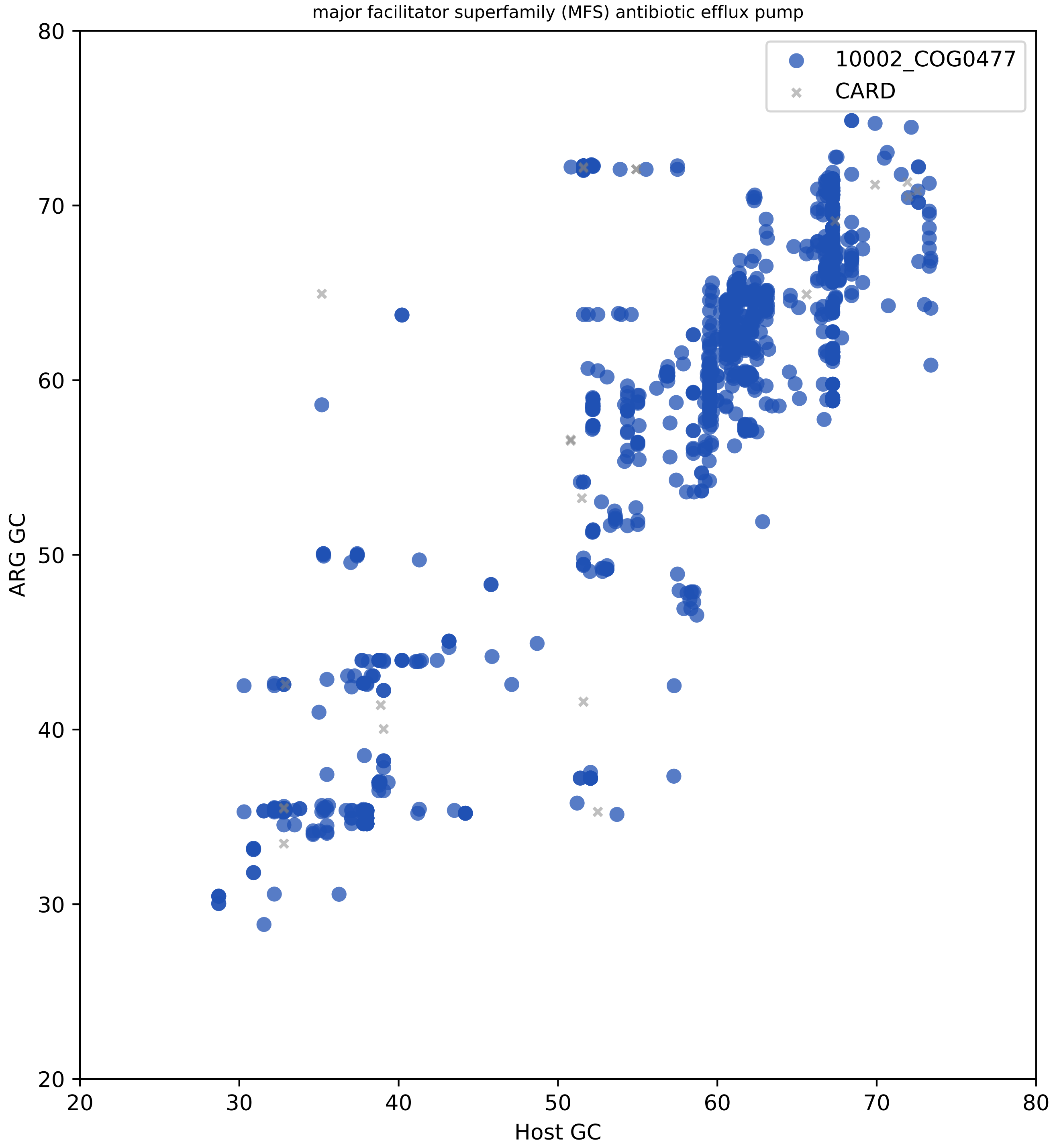

Supplementary Figure S1: (continued).

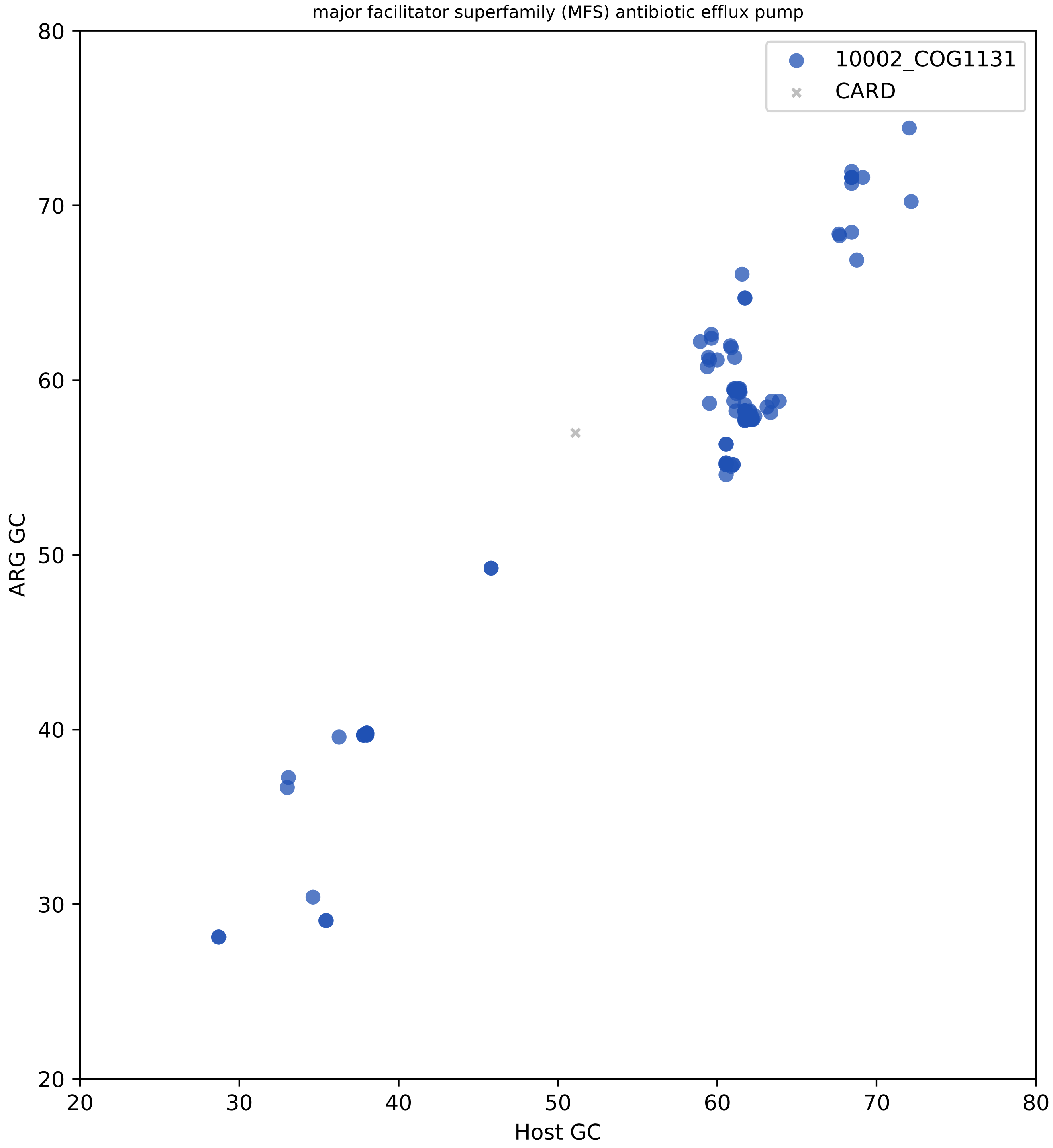

Supplementary Figure S1: (continued).

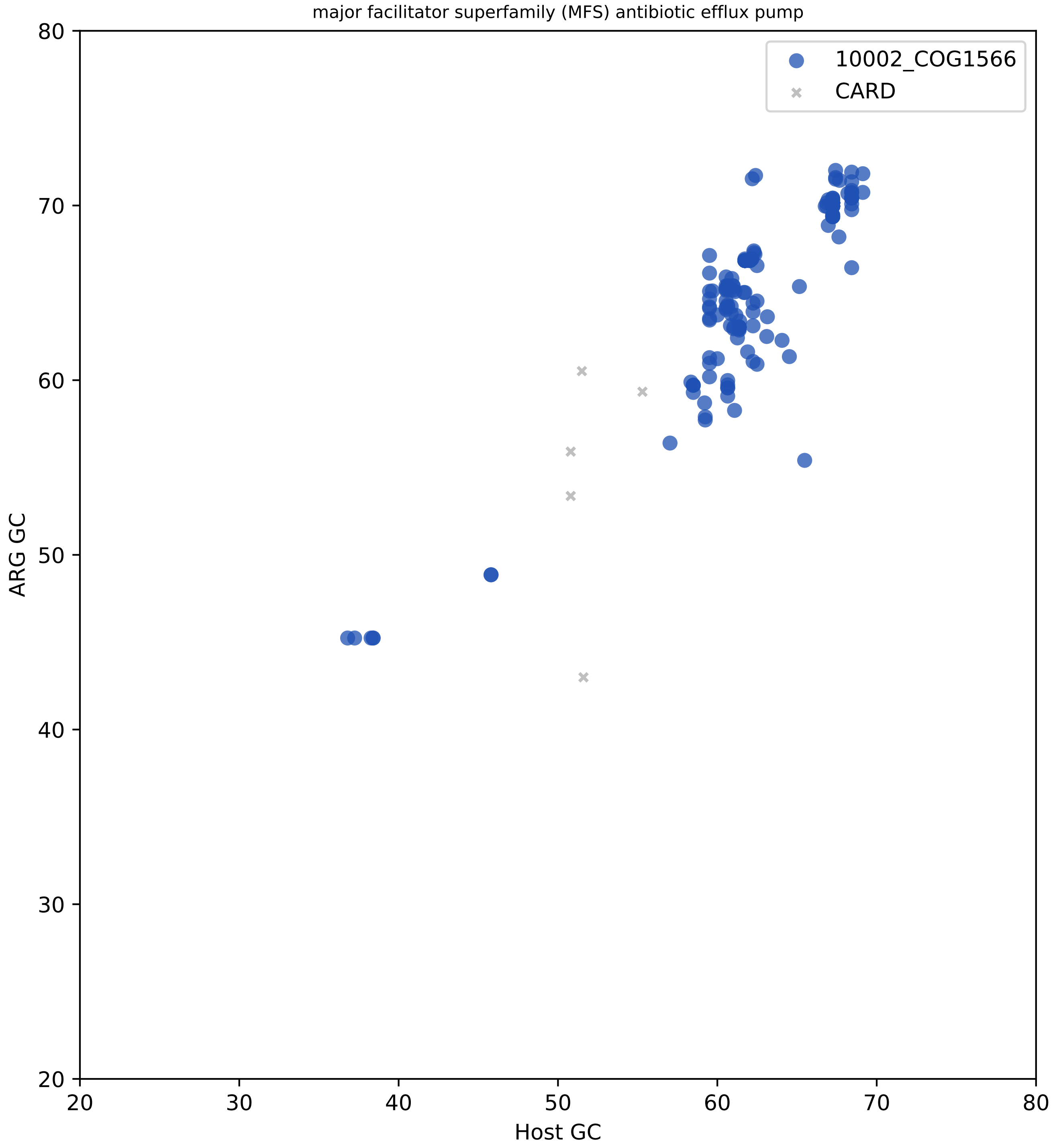

Supplementary Figure S1: (continued).

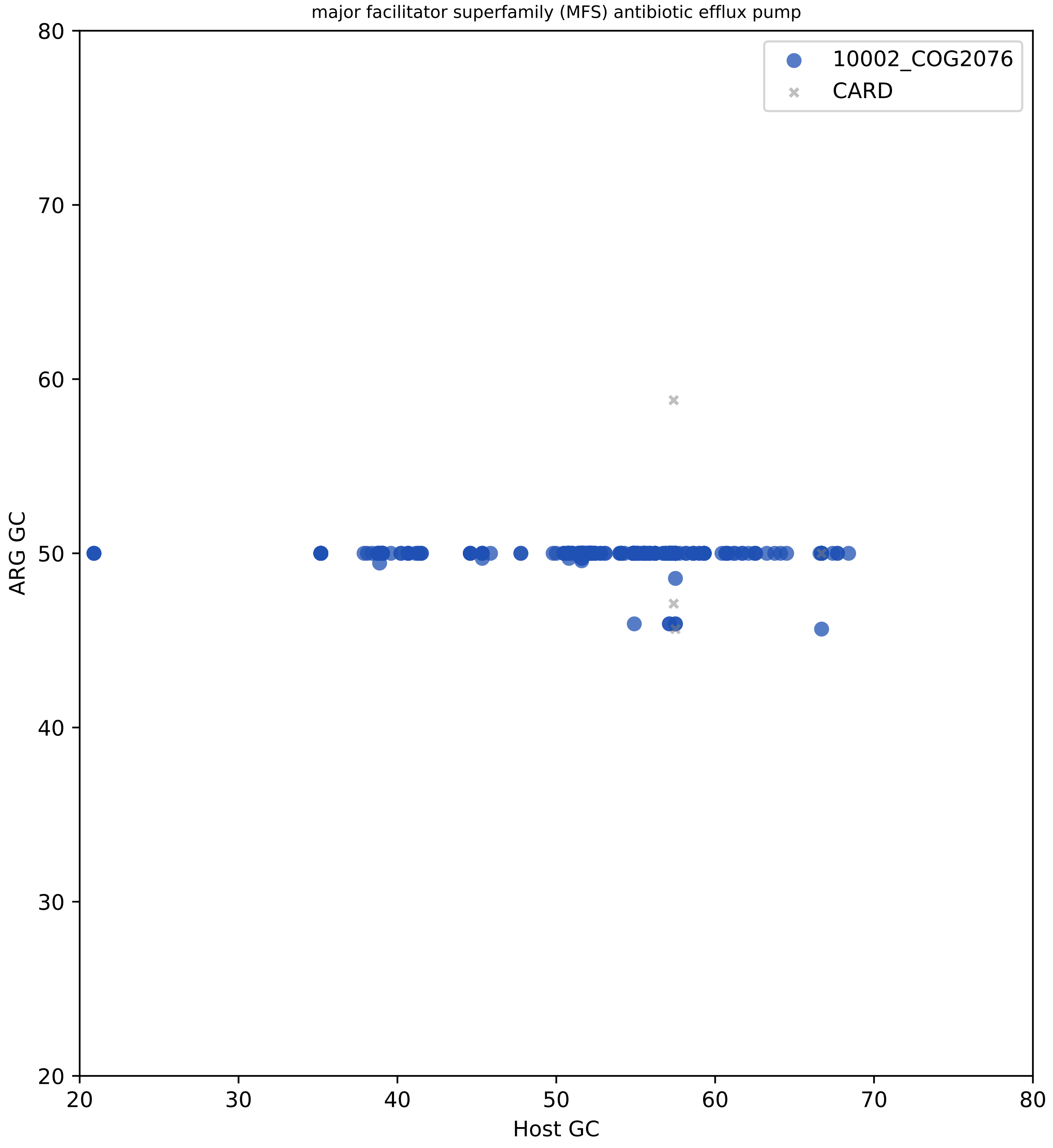

Supplementary Figure S1: (continued).

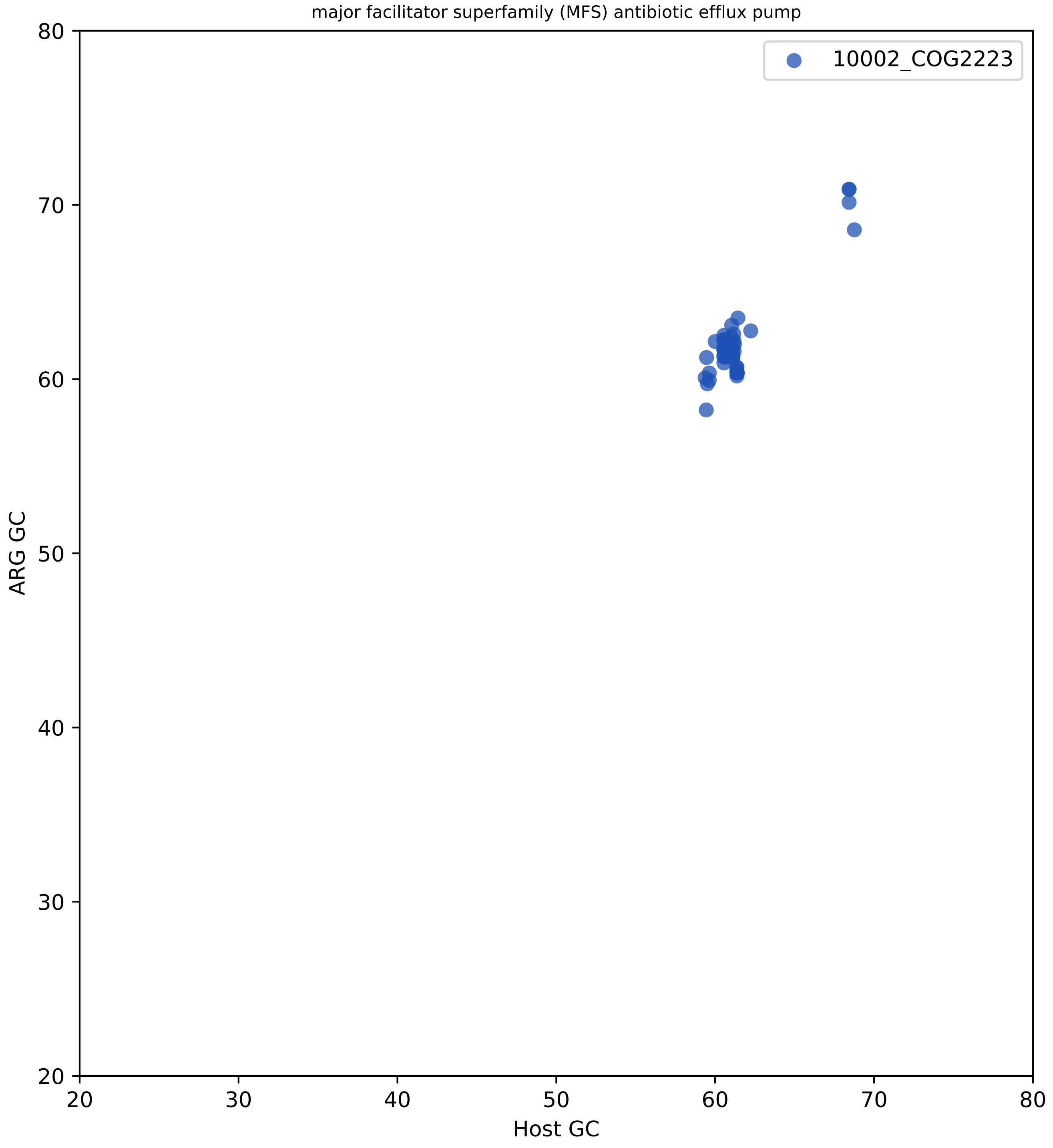

Supplementary Figure S1: (continued).

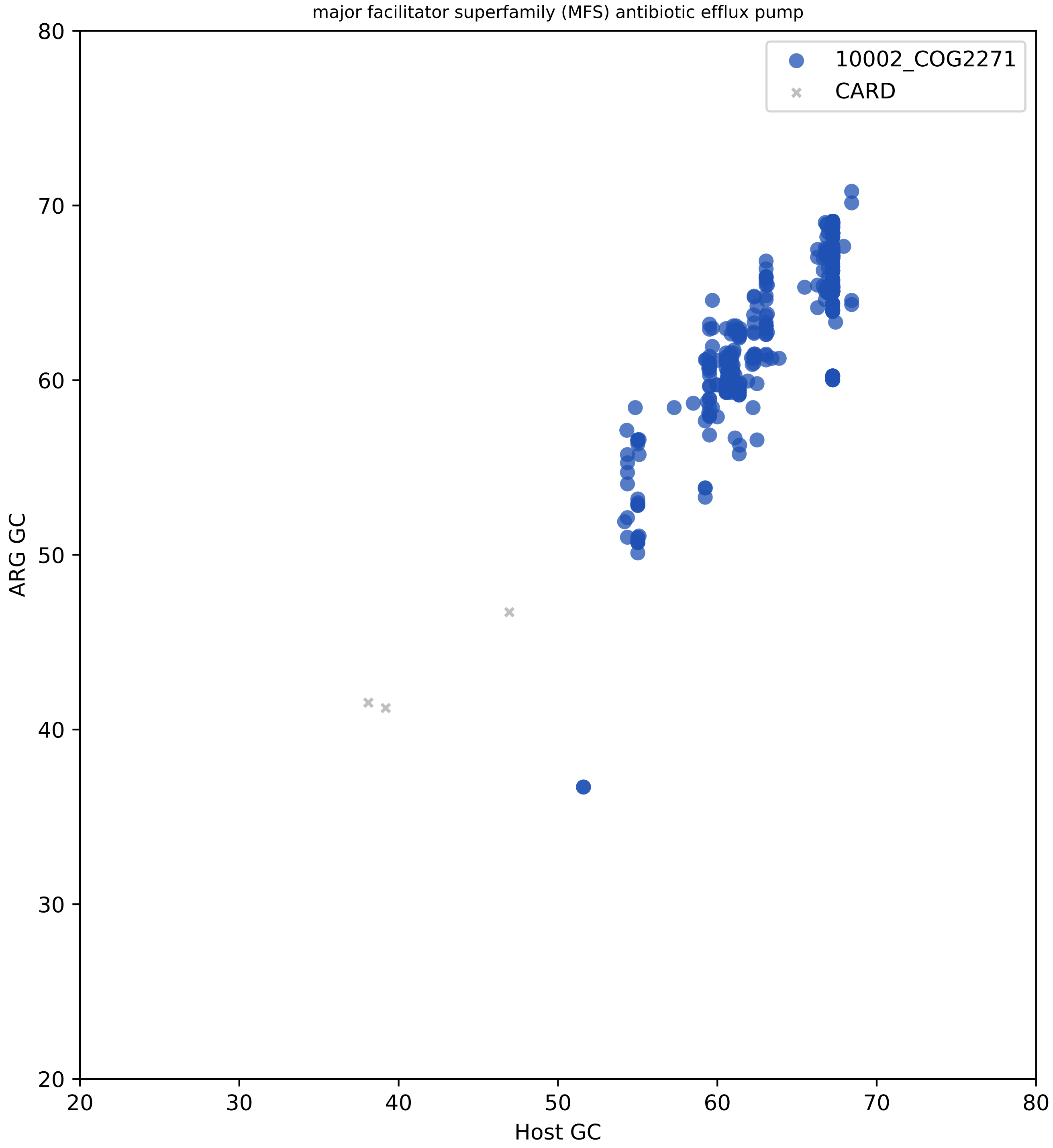

Supplementary Figure S1: (continued).

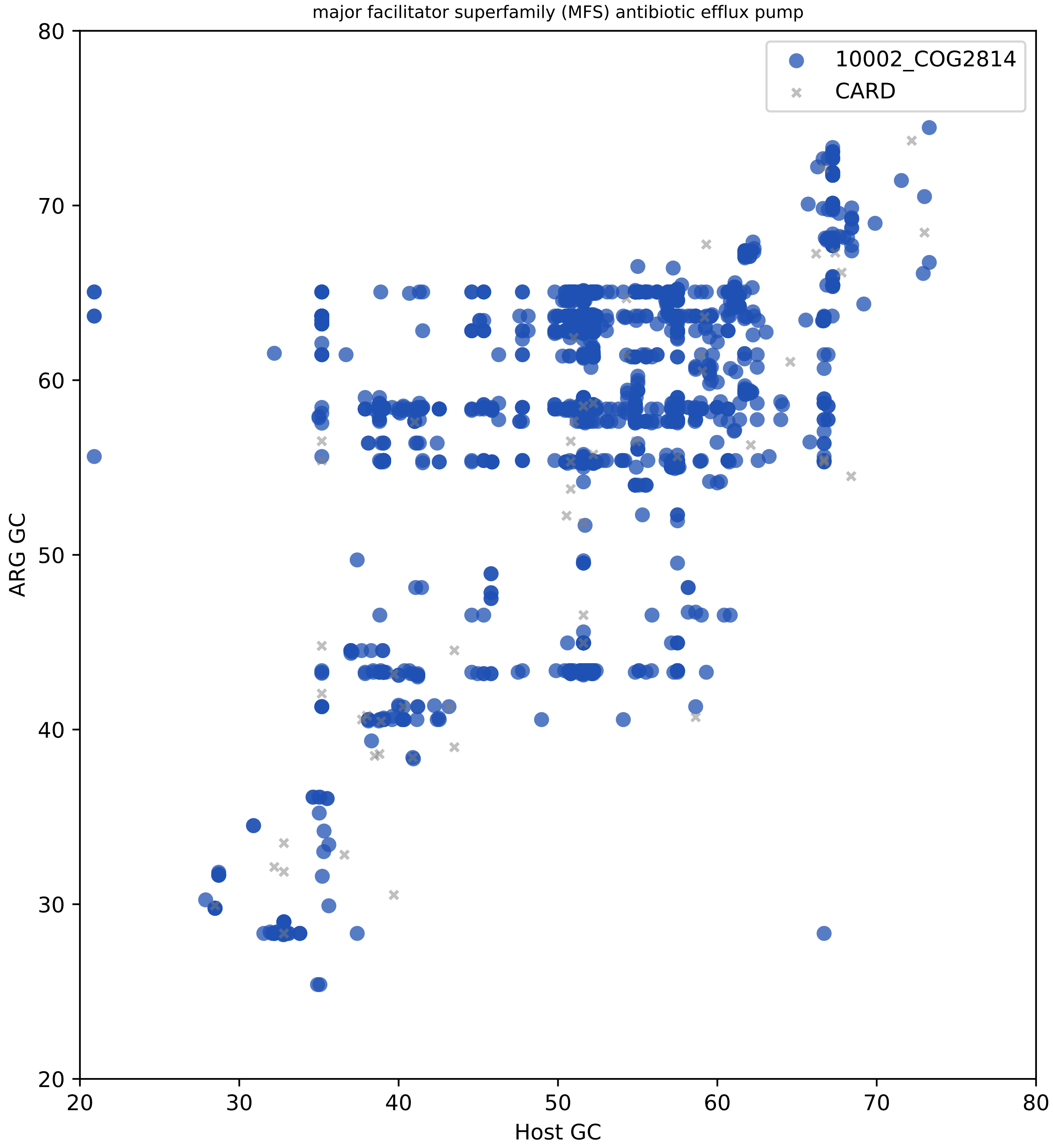

Supplementary Figure S1: (continued).

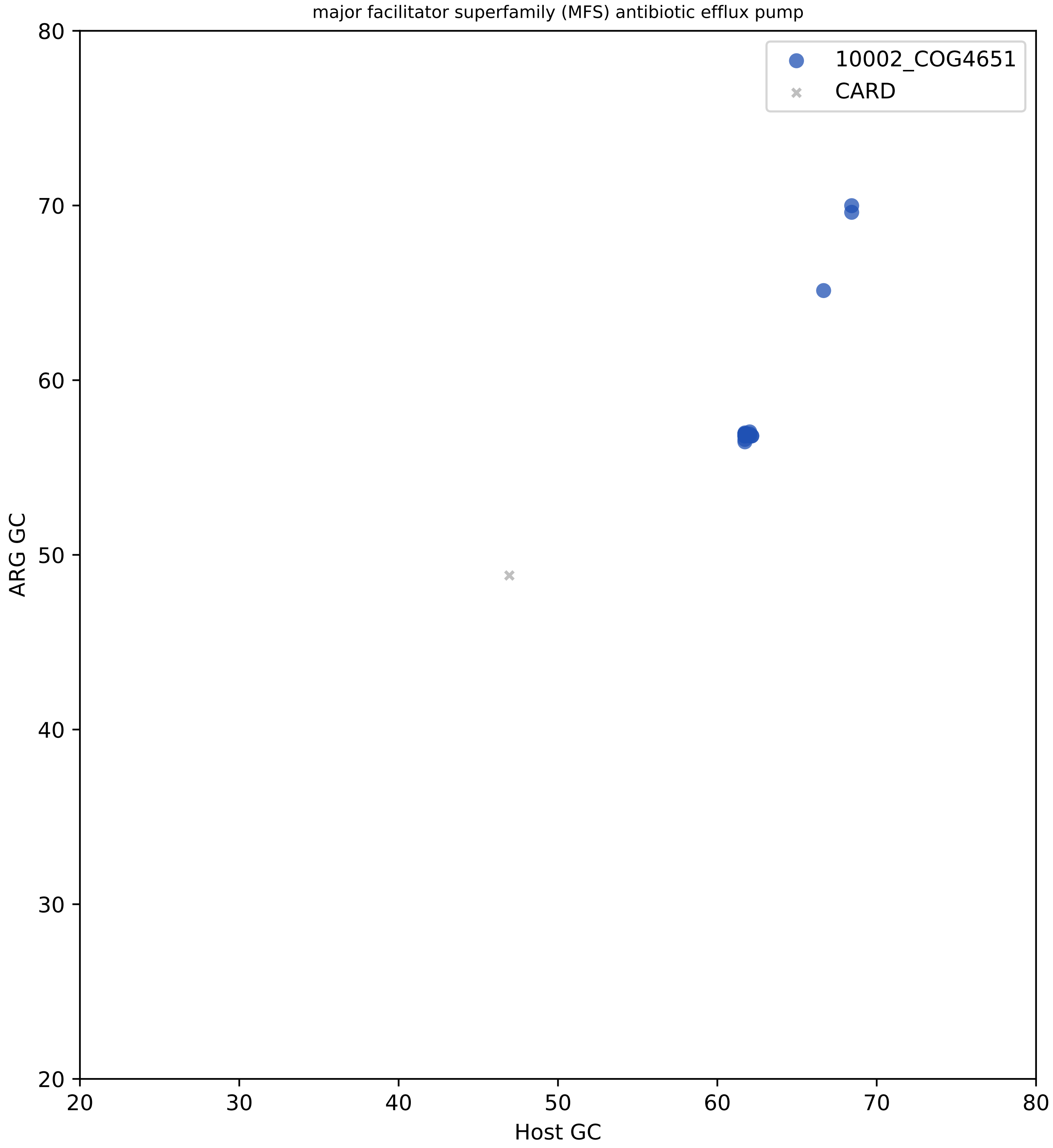

Supplementary Figure S1: (continued).

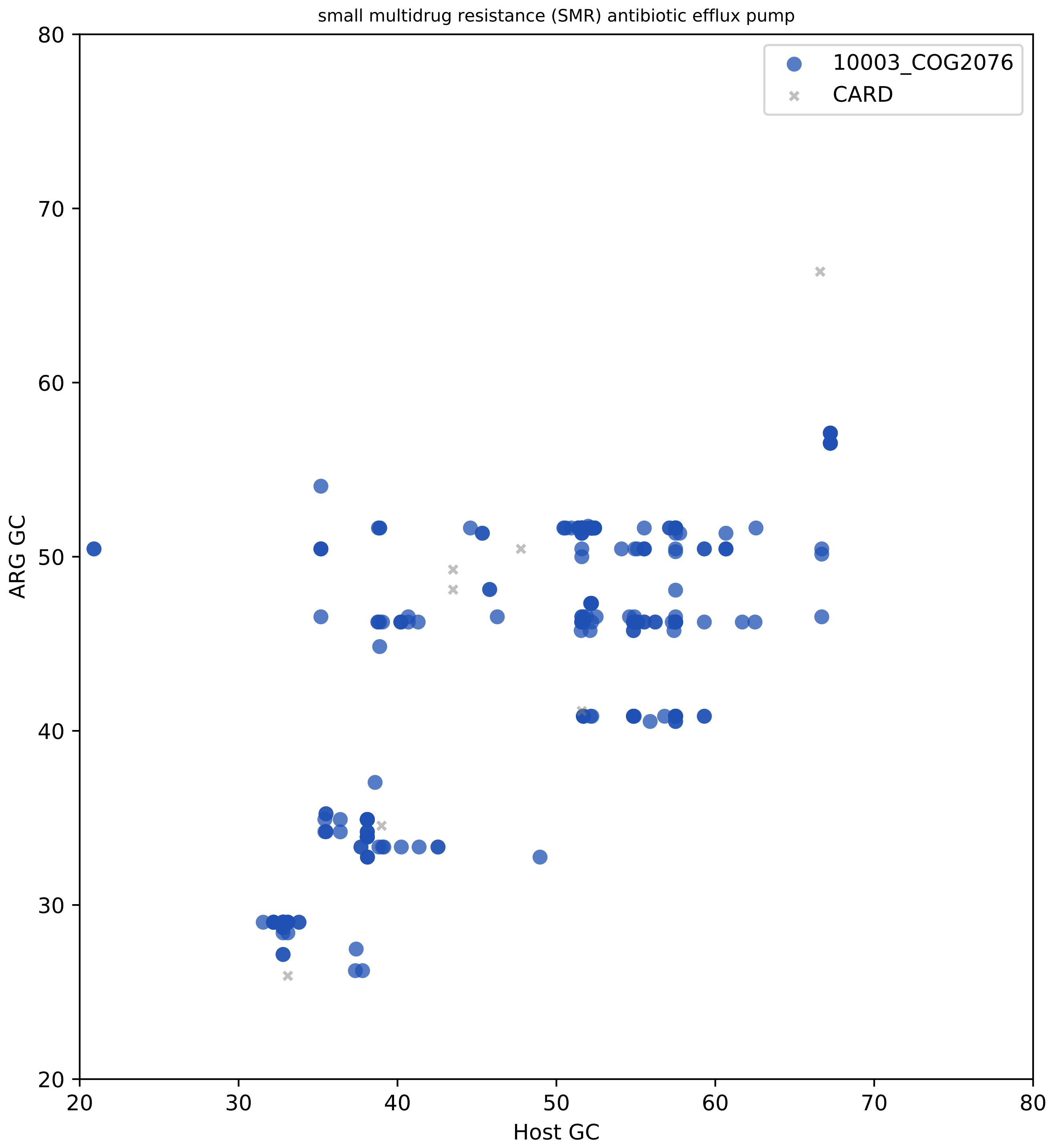

Supplementary Figure S1: (continued).

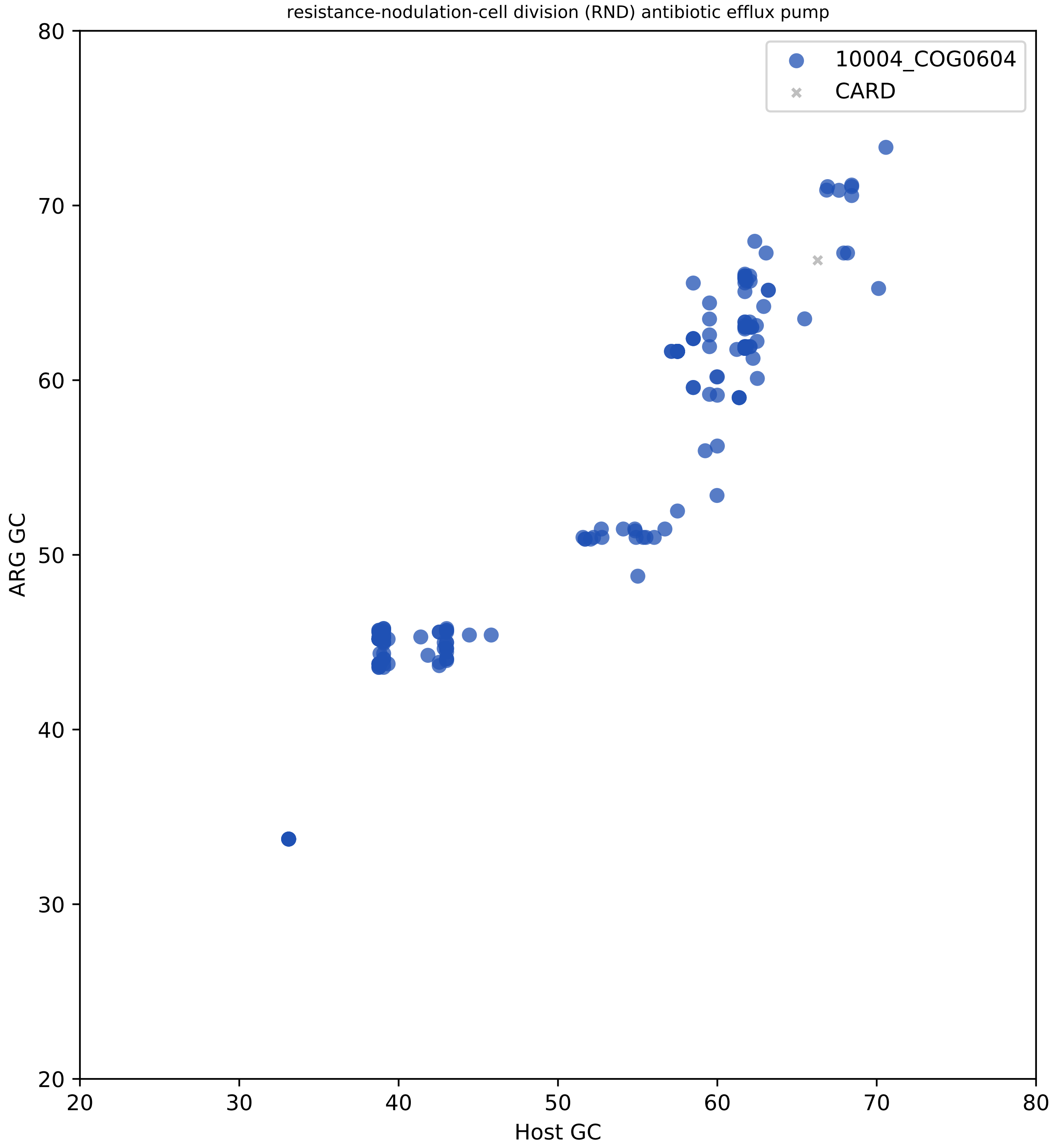

Supplementary Figure S1: (continued).

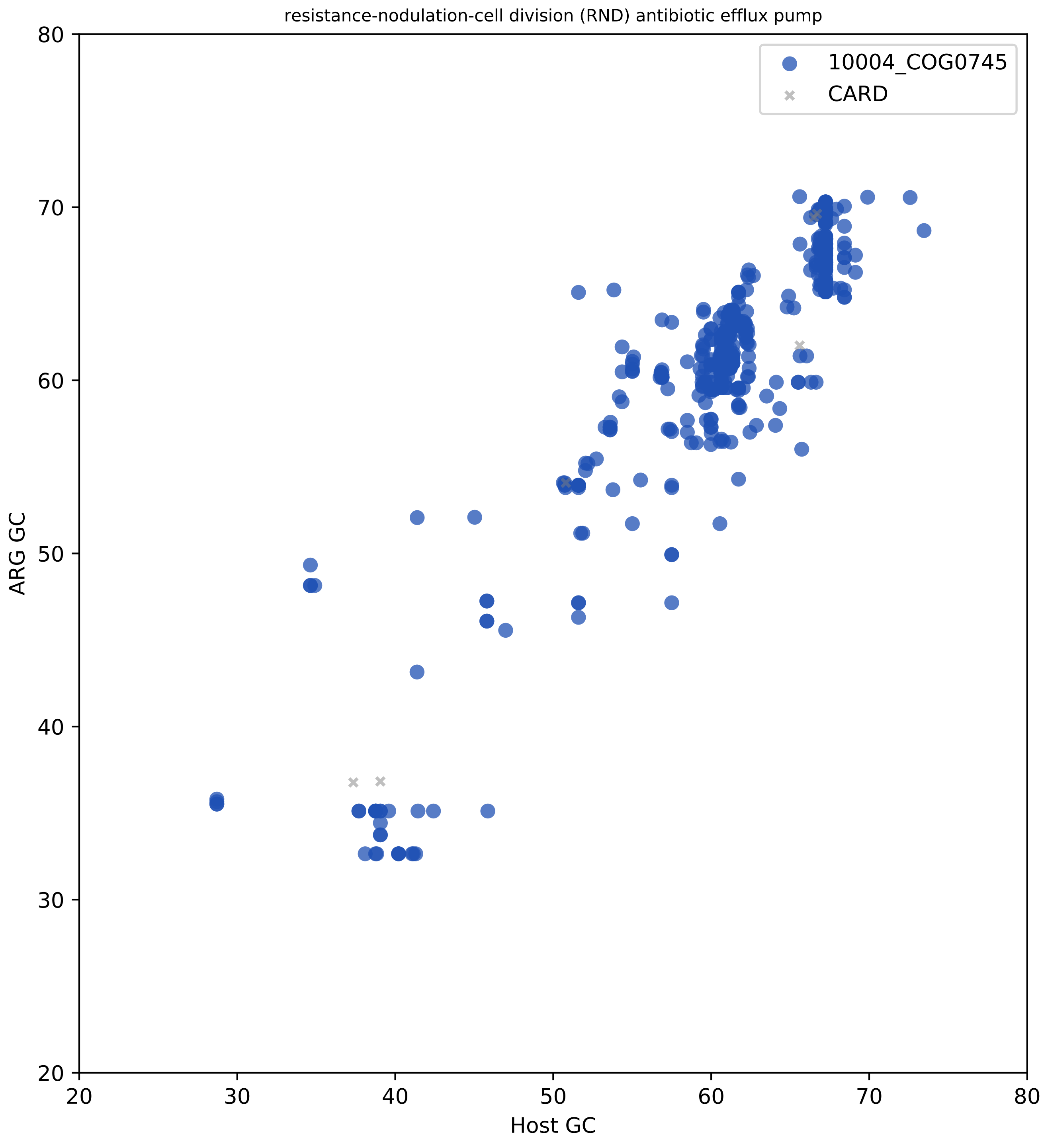

Supplementary Figure S1: (continued).

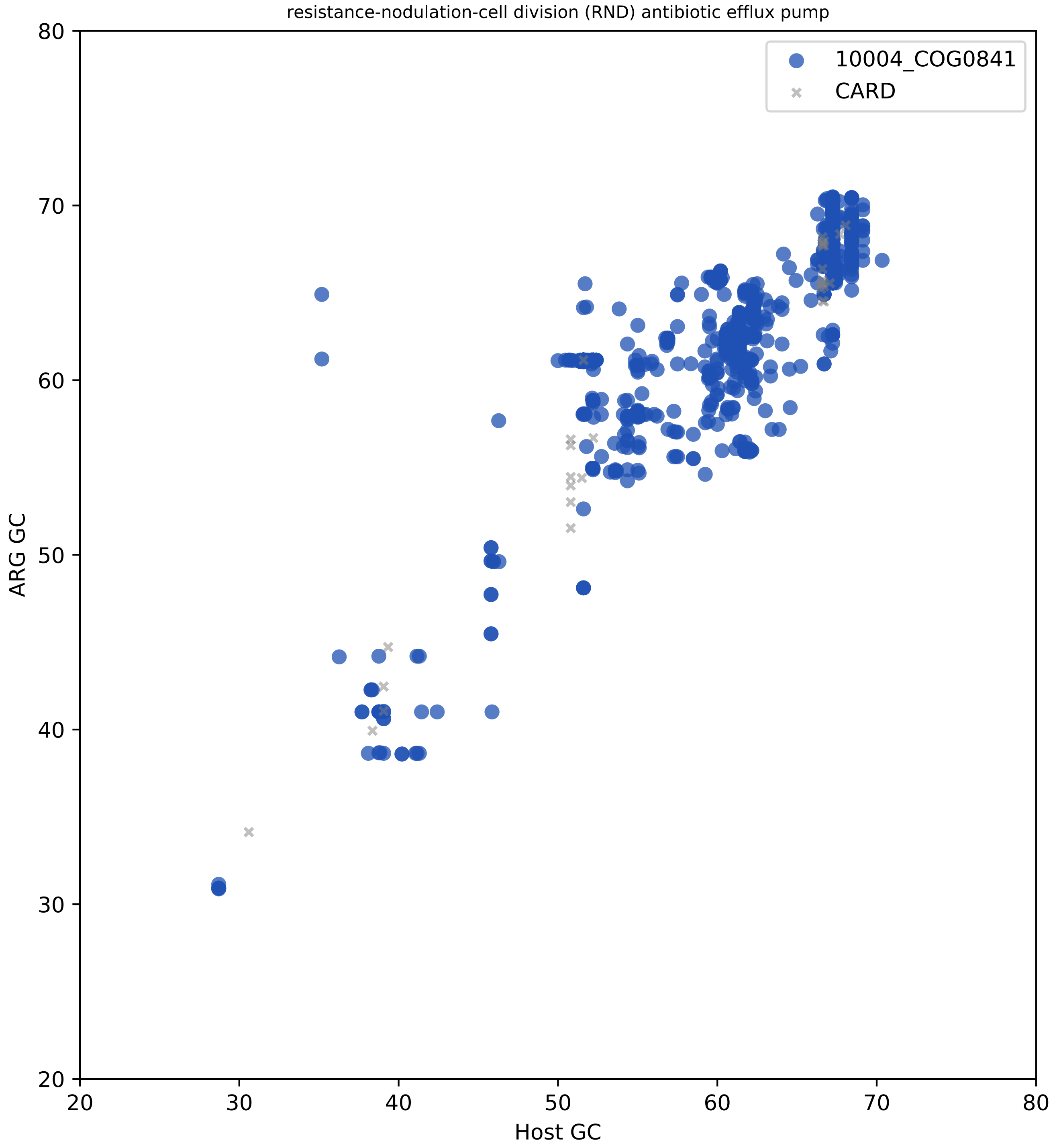

Supplementary Figure S1: (continued).

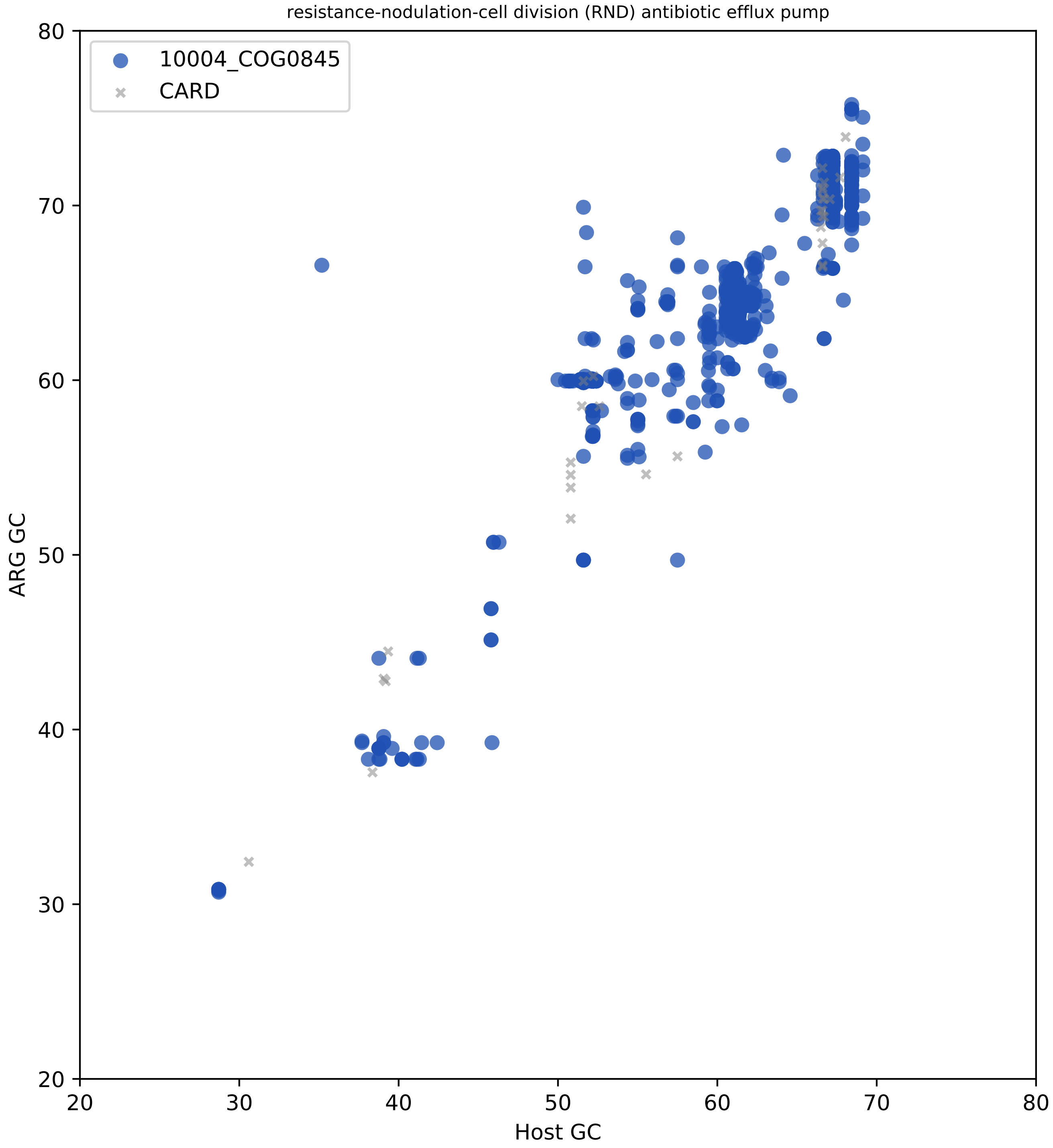

Supplementary Figure S1: (continued).

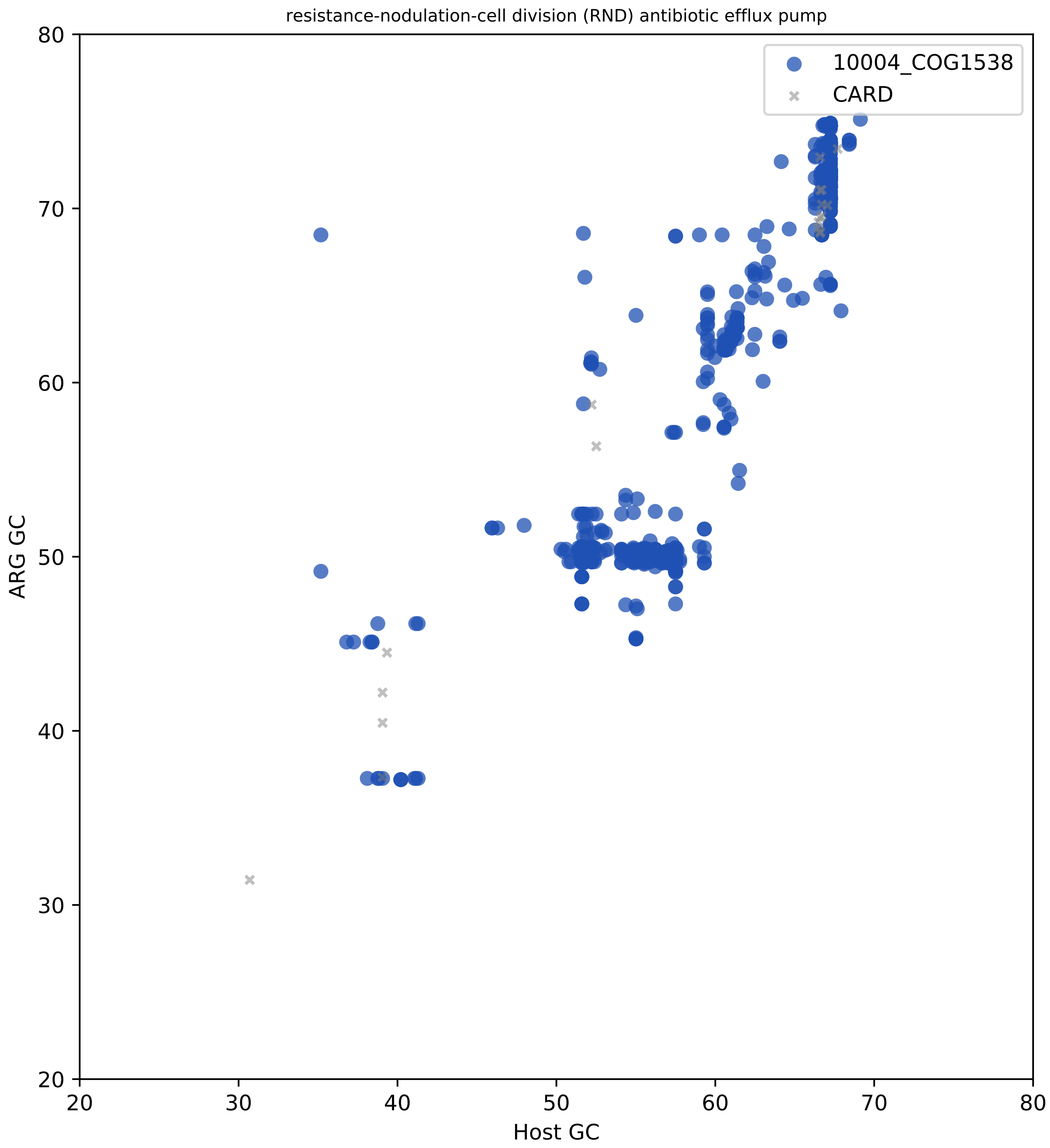

Supplementary Figure S1: (continued).

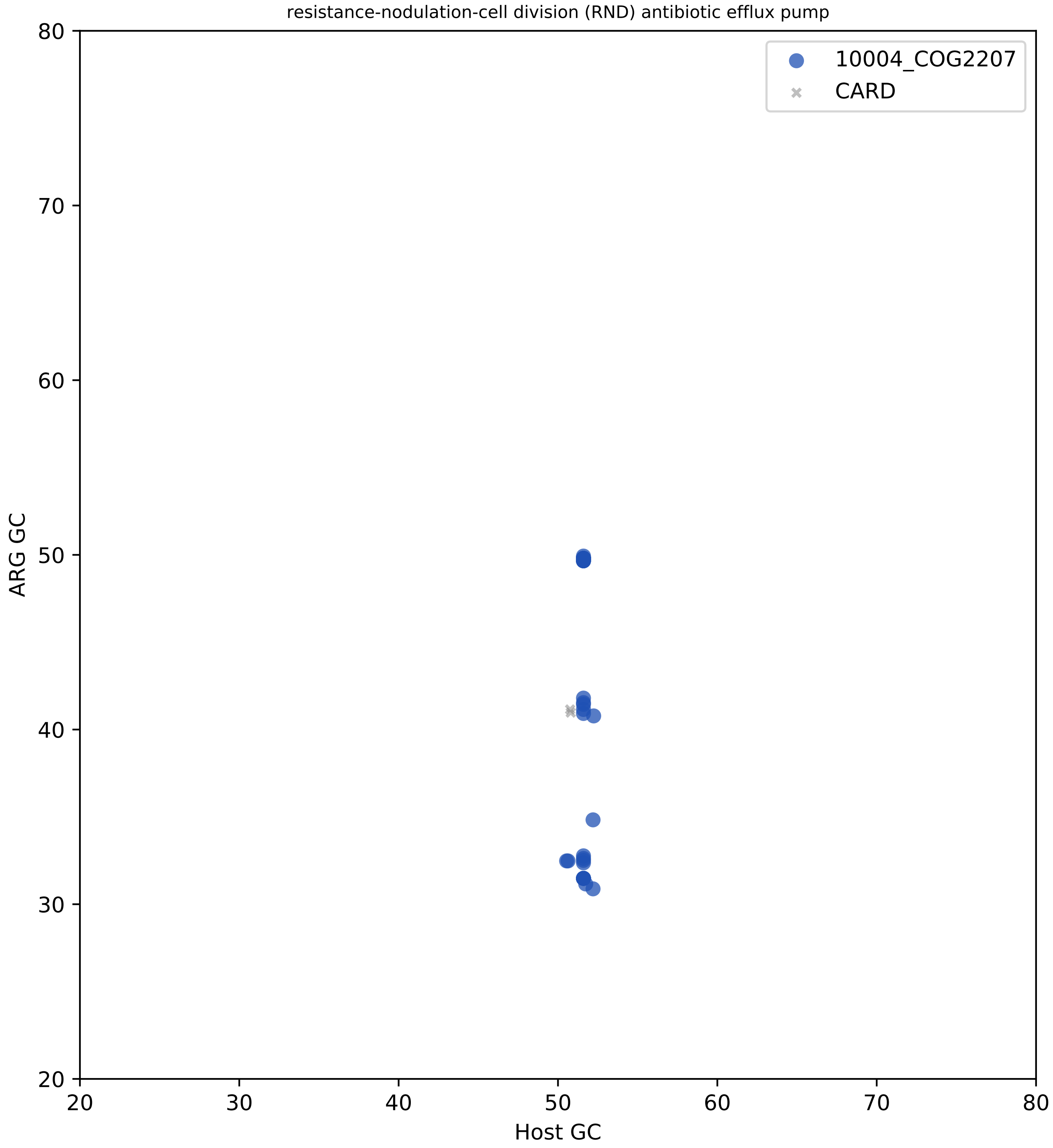

Supplementary Figure S1: (continued).

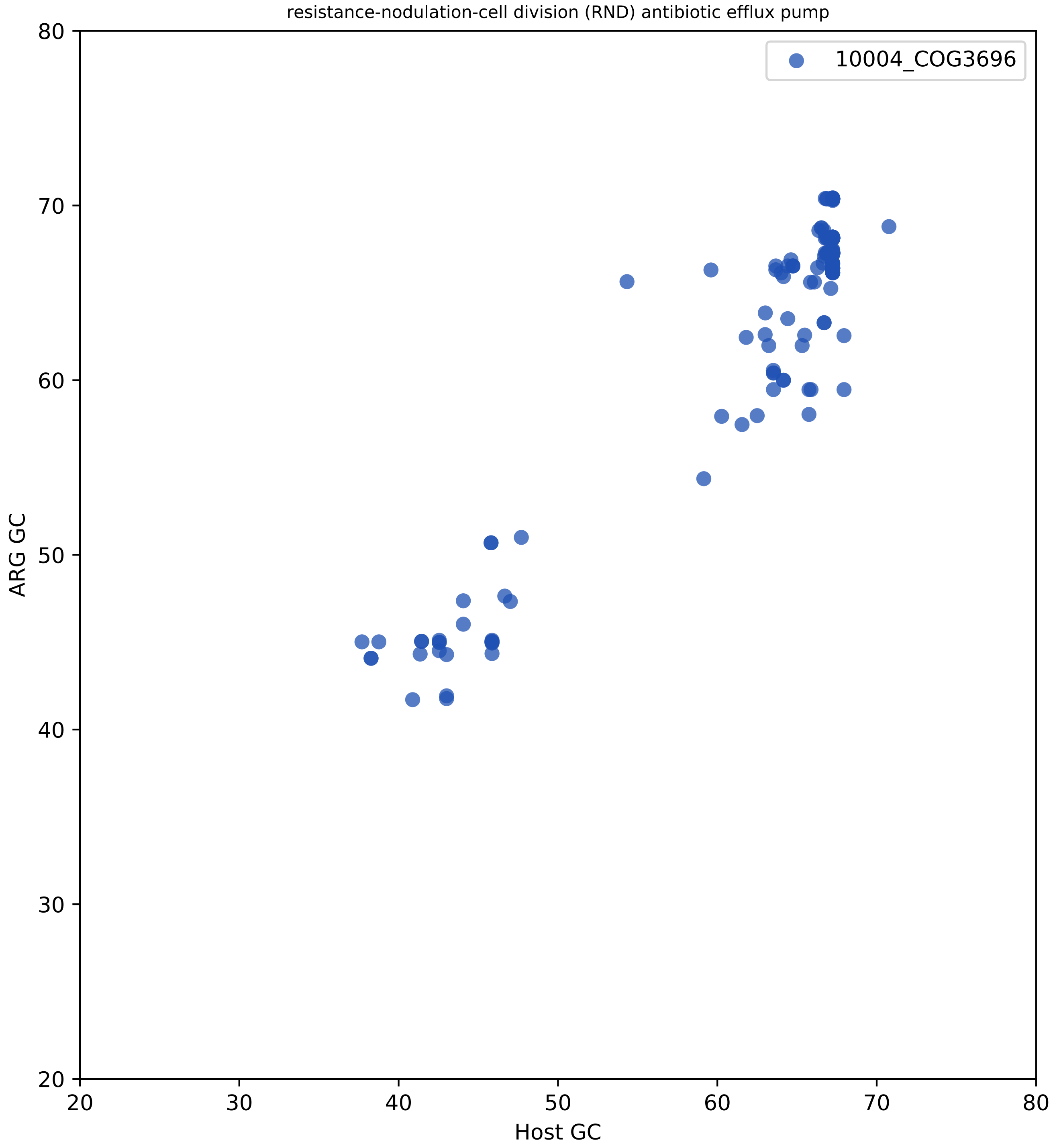

Supplementary Figure S1: (continued).

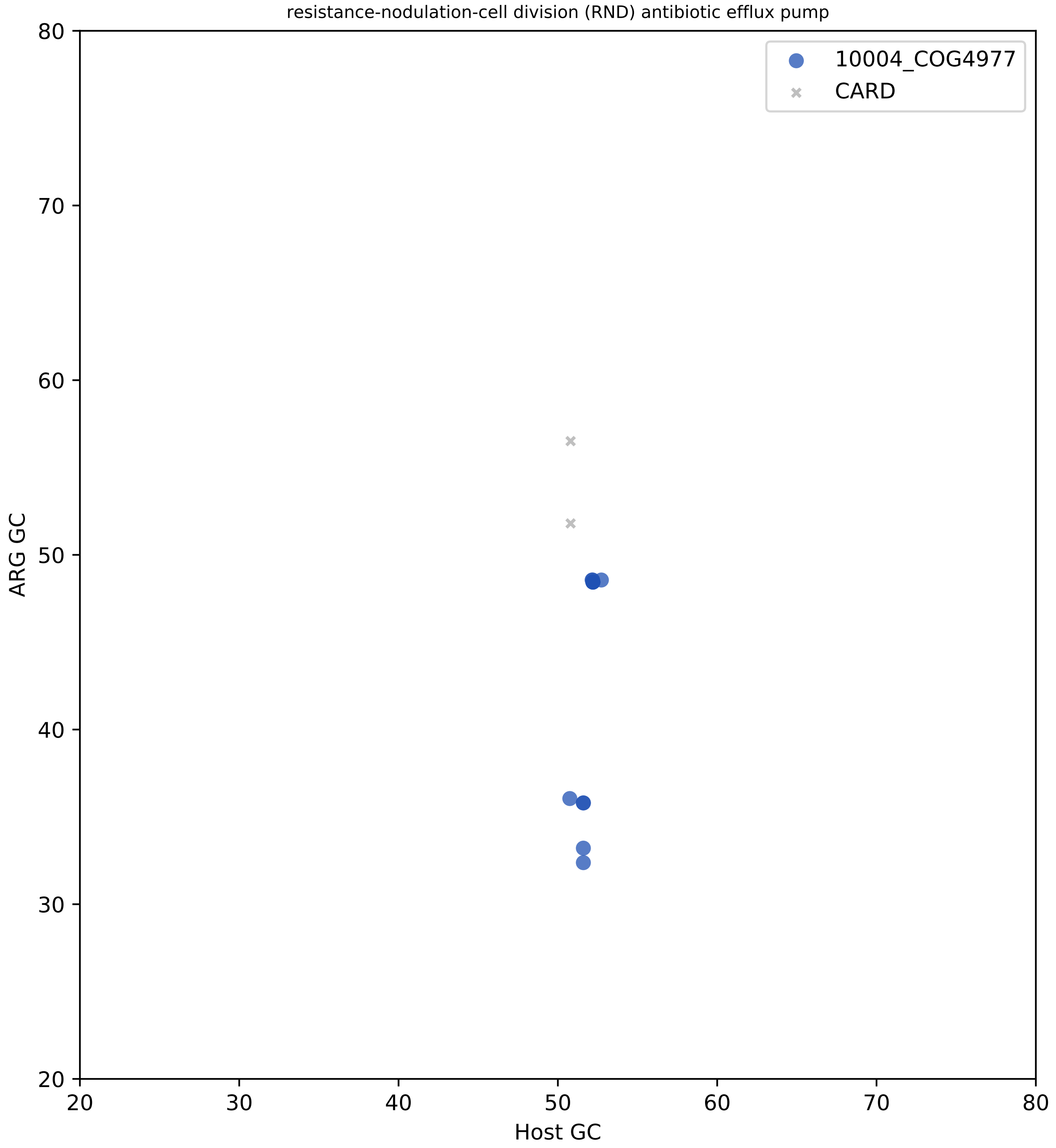

Supplementary Figure S1: (continued).

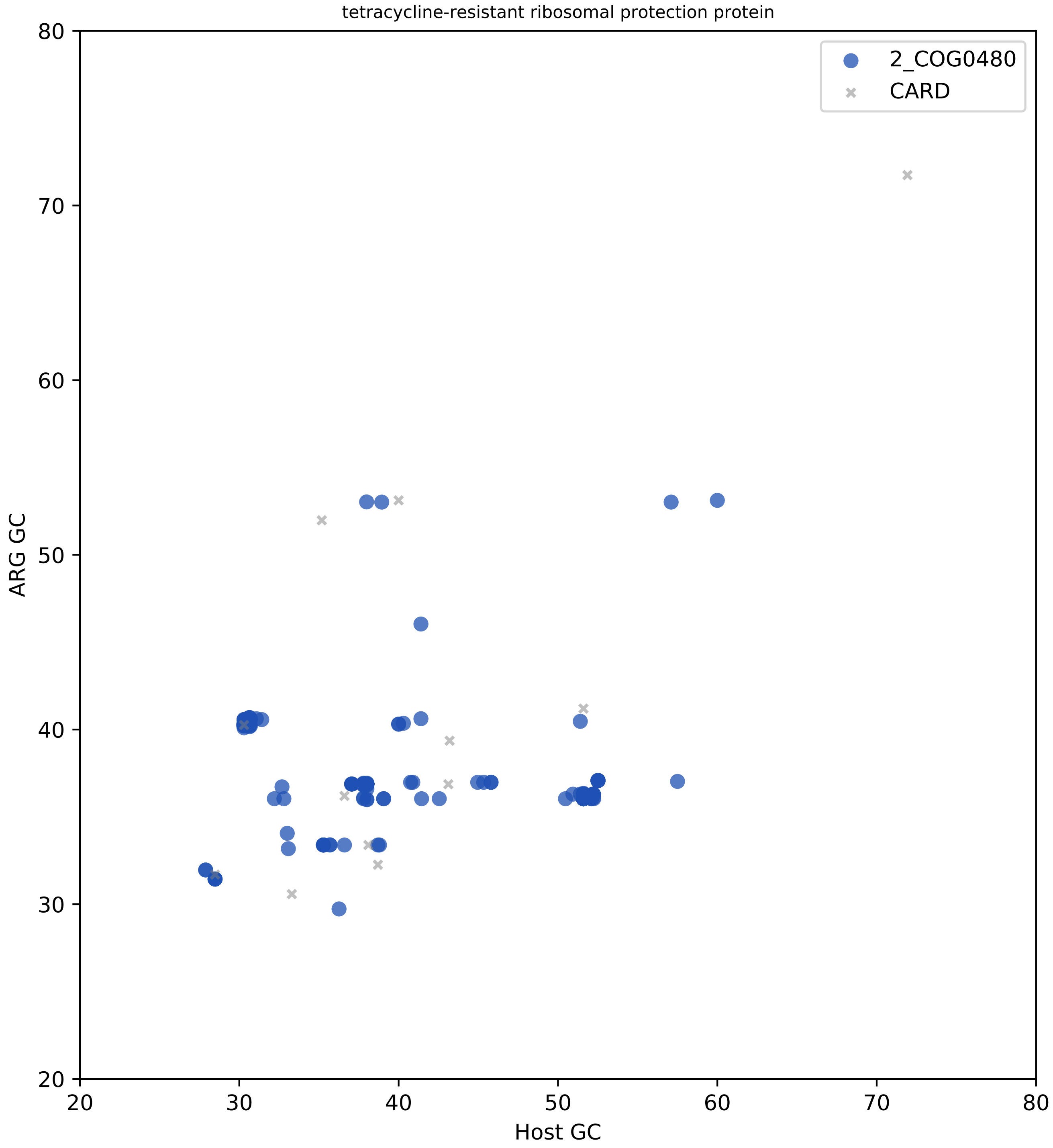

Supplementary Figure S1: (continued).

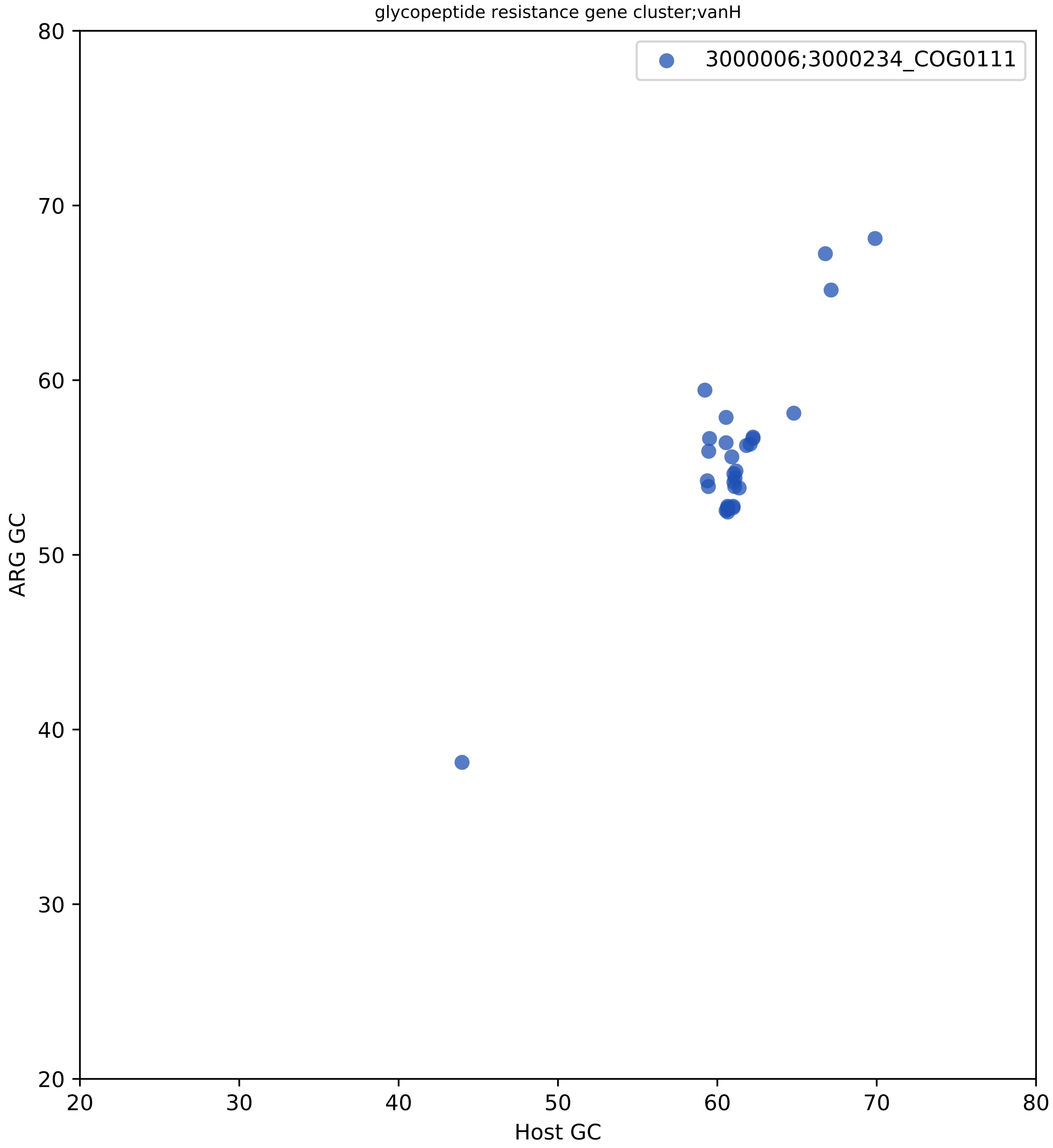

Supplementary Figure S1: (continued).

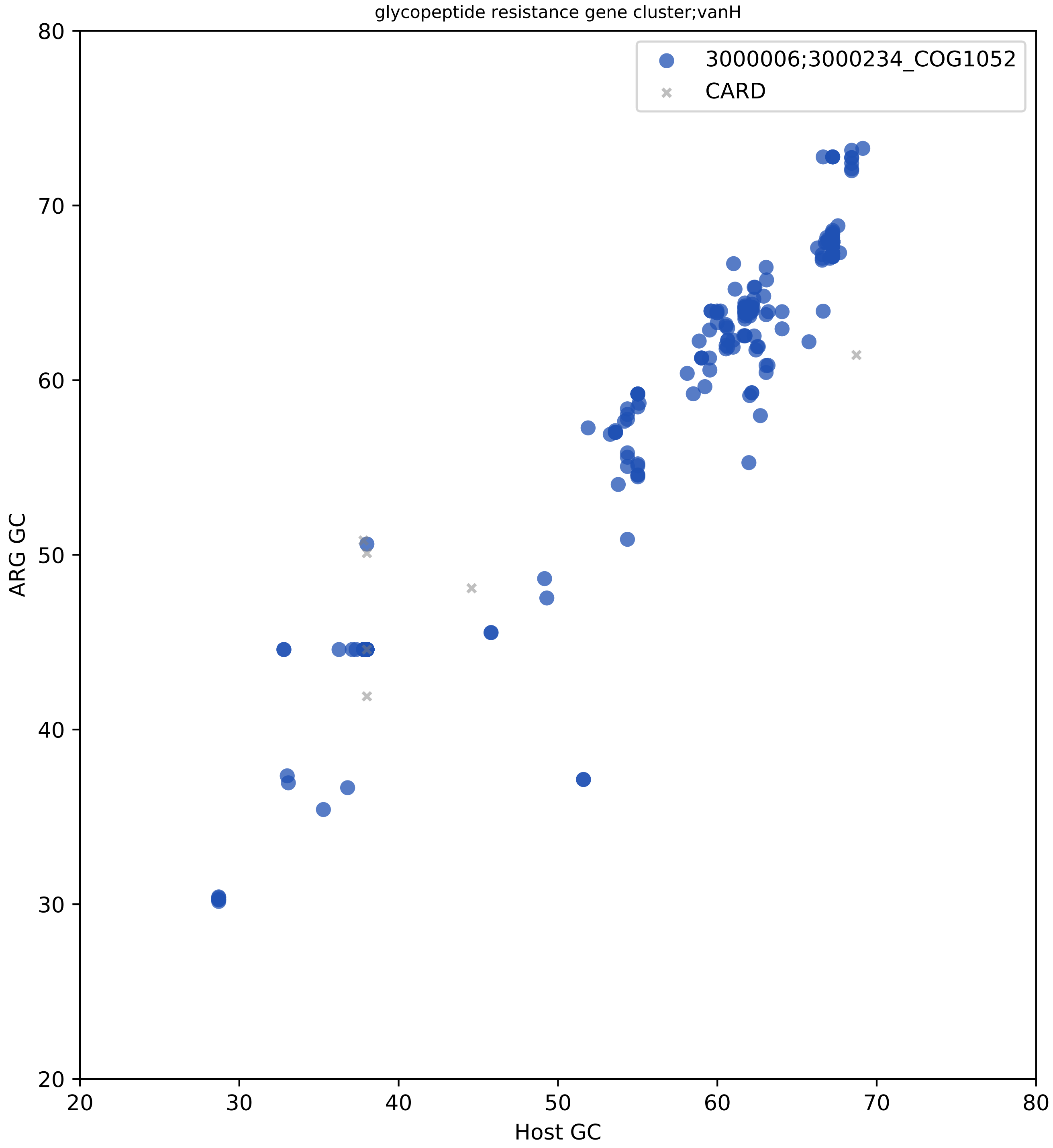

Supplementary Figure S1: (continued).

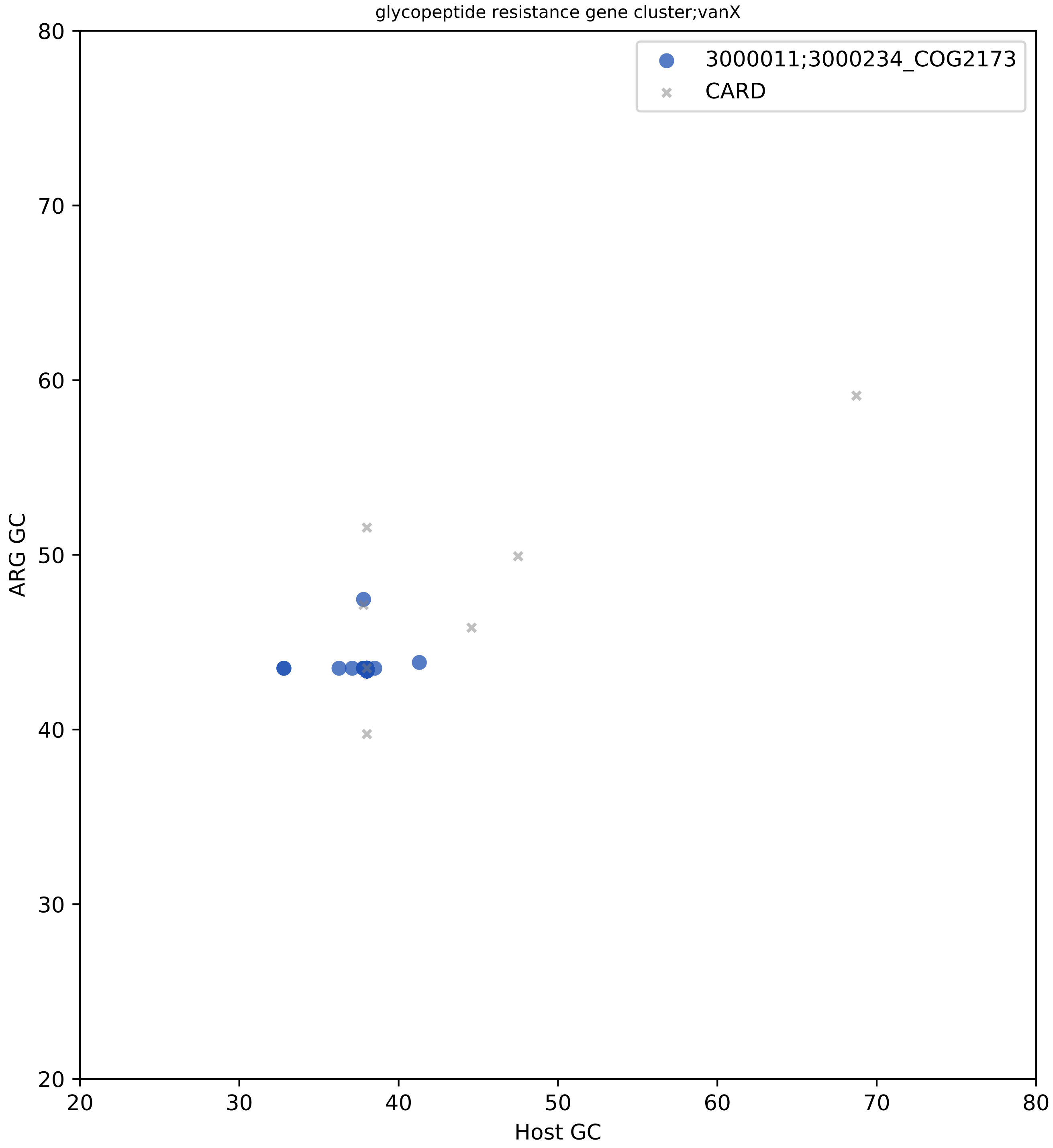

Supplementary Figure S1: (continued).

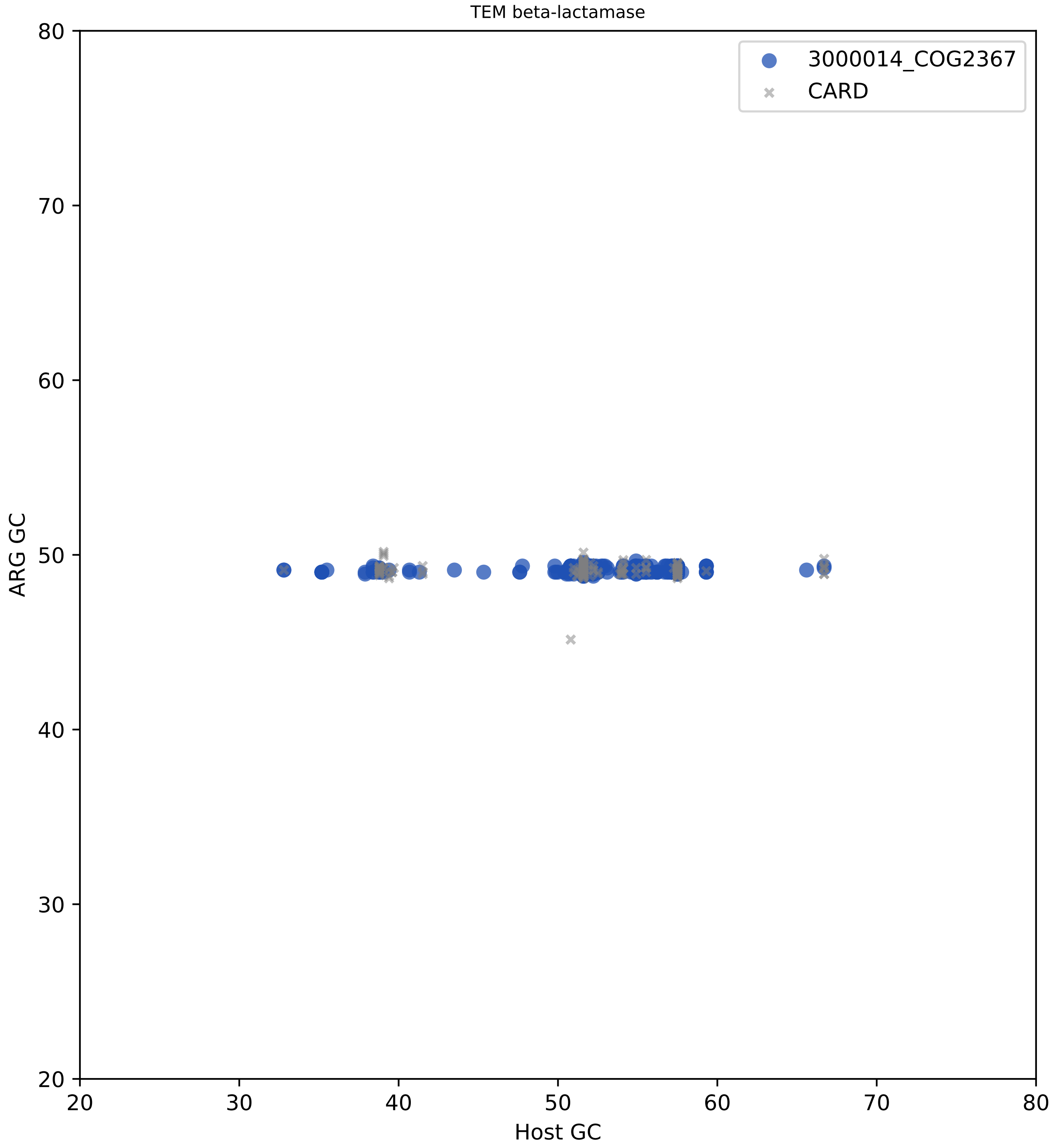

Supplementary Figure S1: (continued).

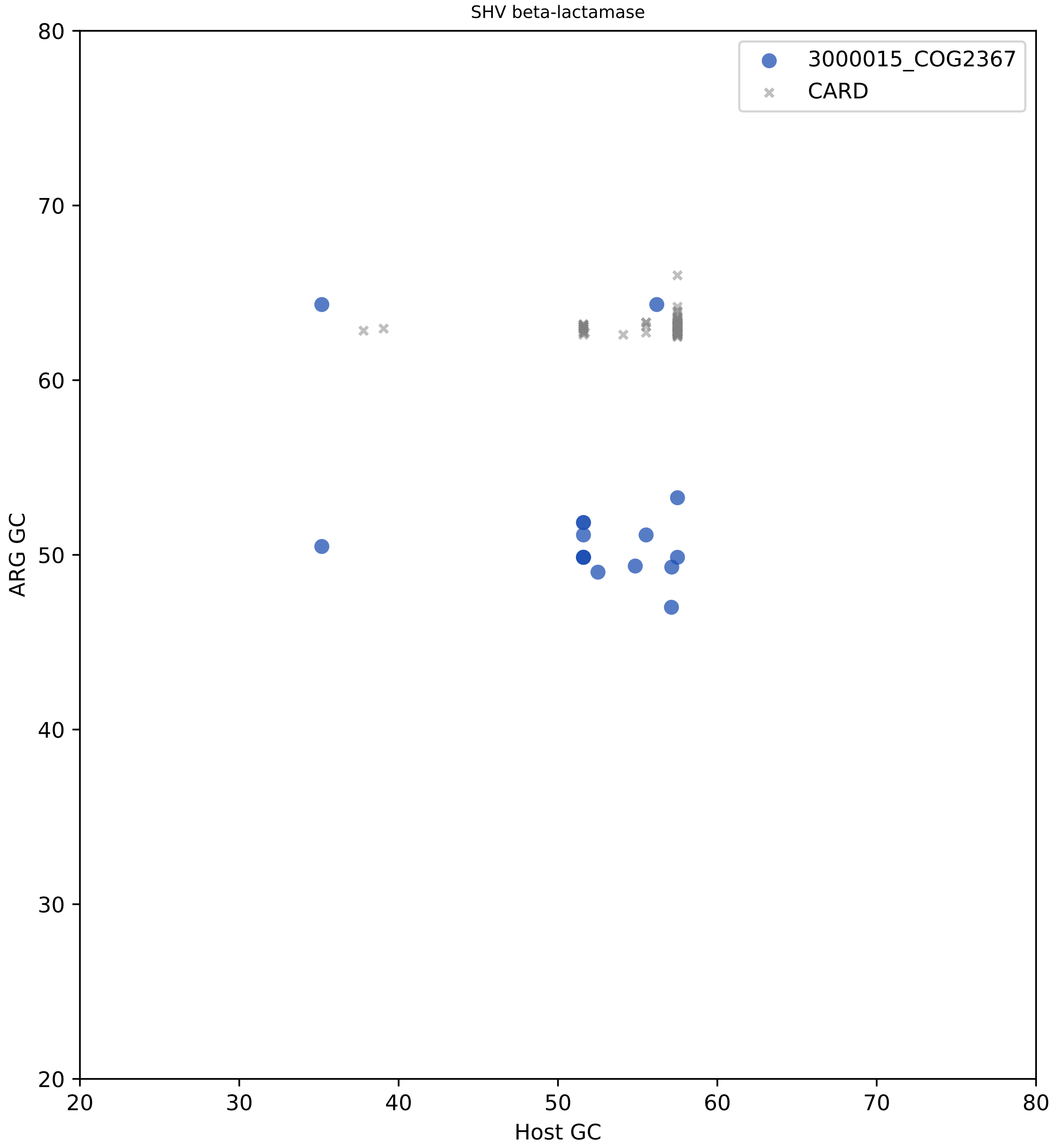

Supplementary Figure S1: (continued).

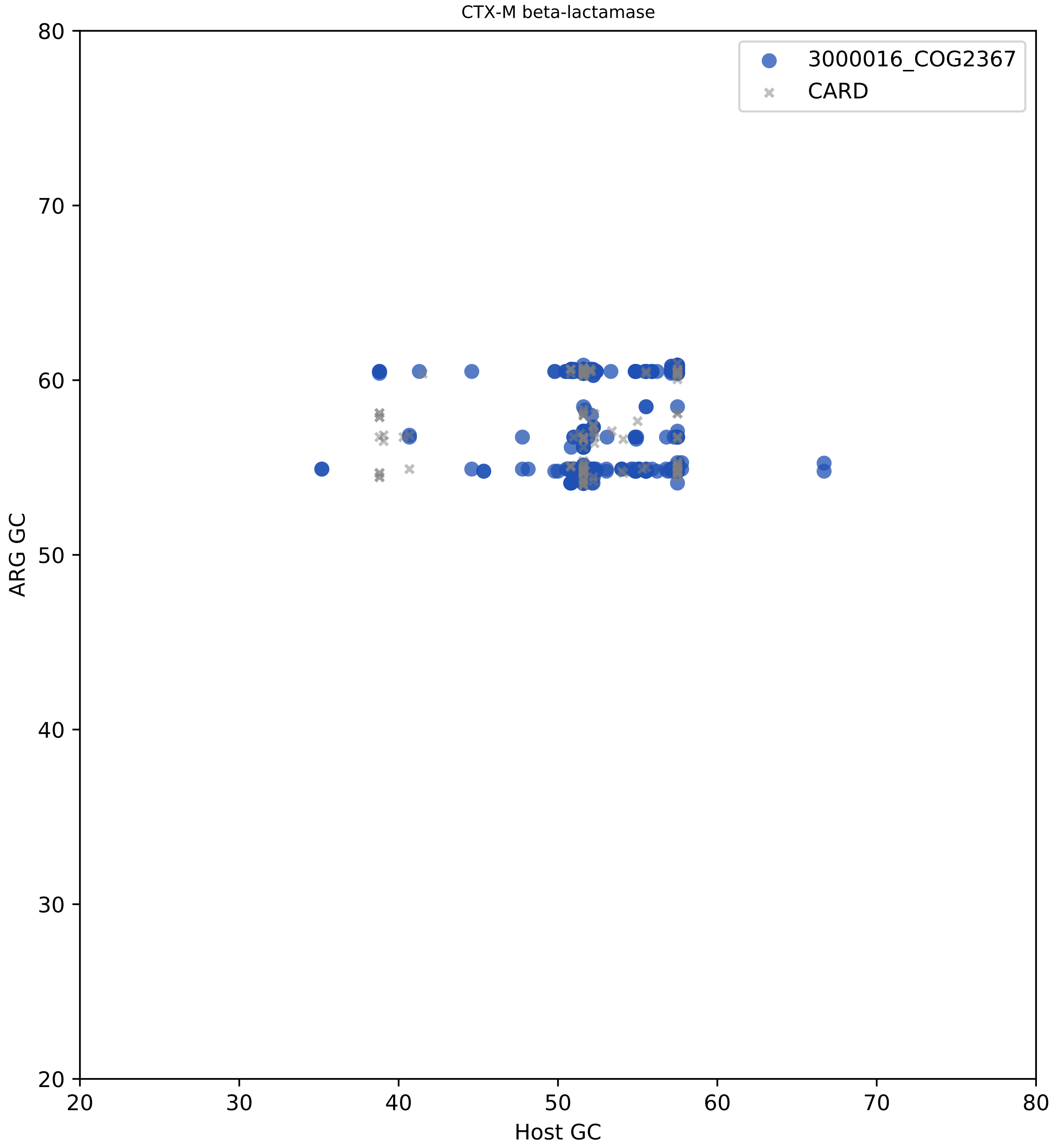

Supplementary Figure S1: (continued).

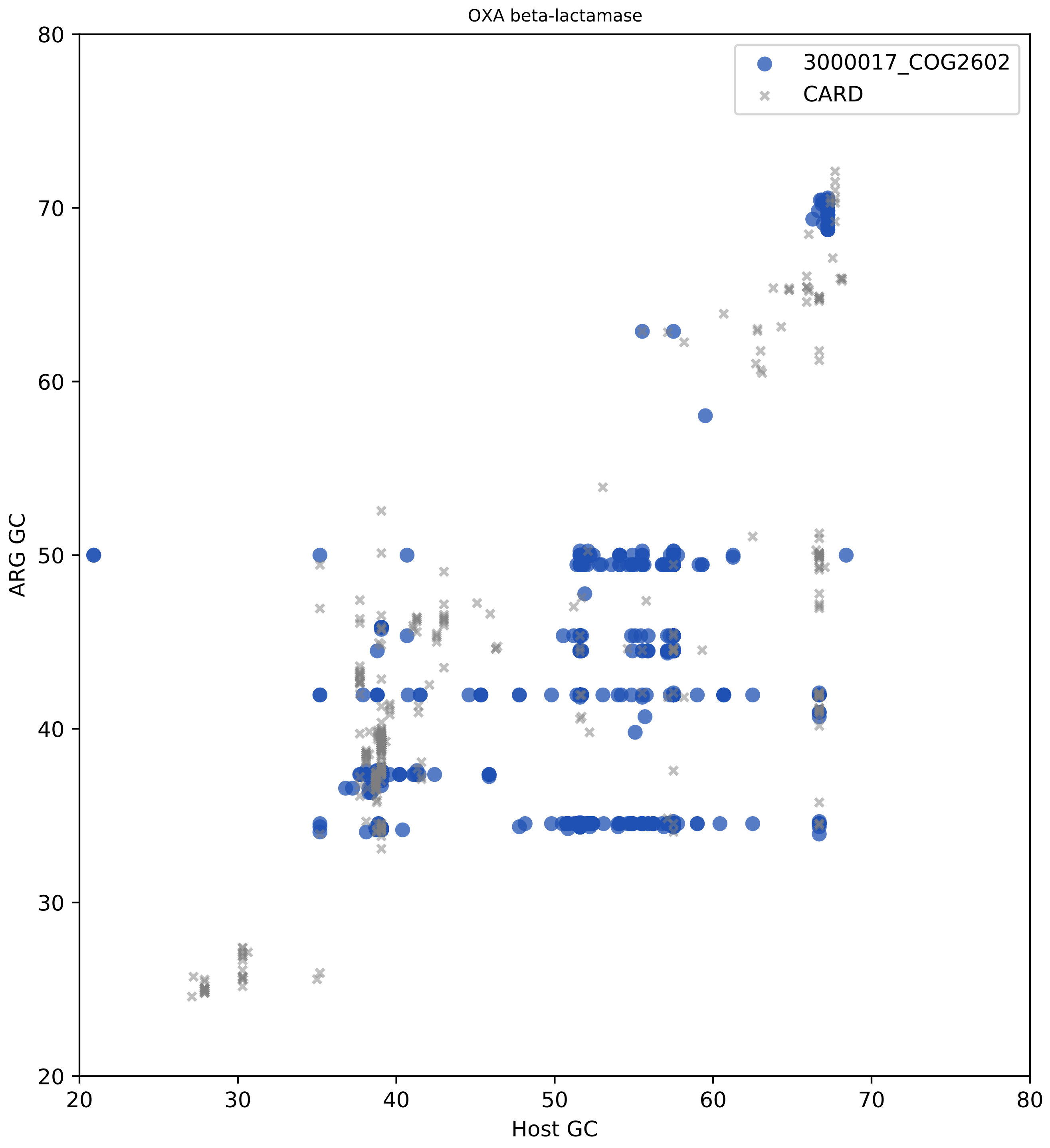

Supplementary Figure S1: (continued).

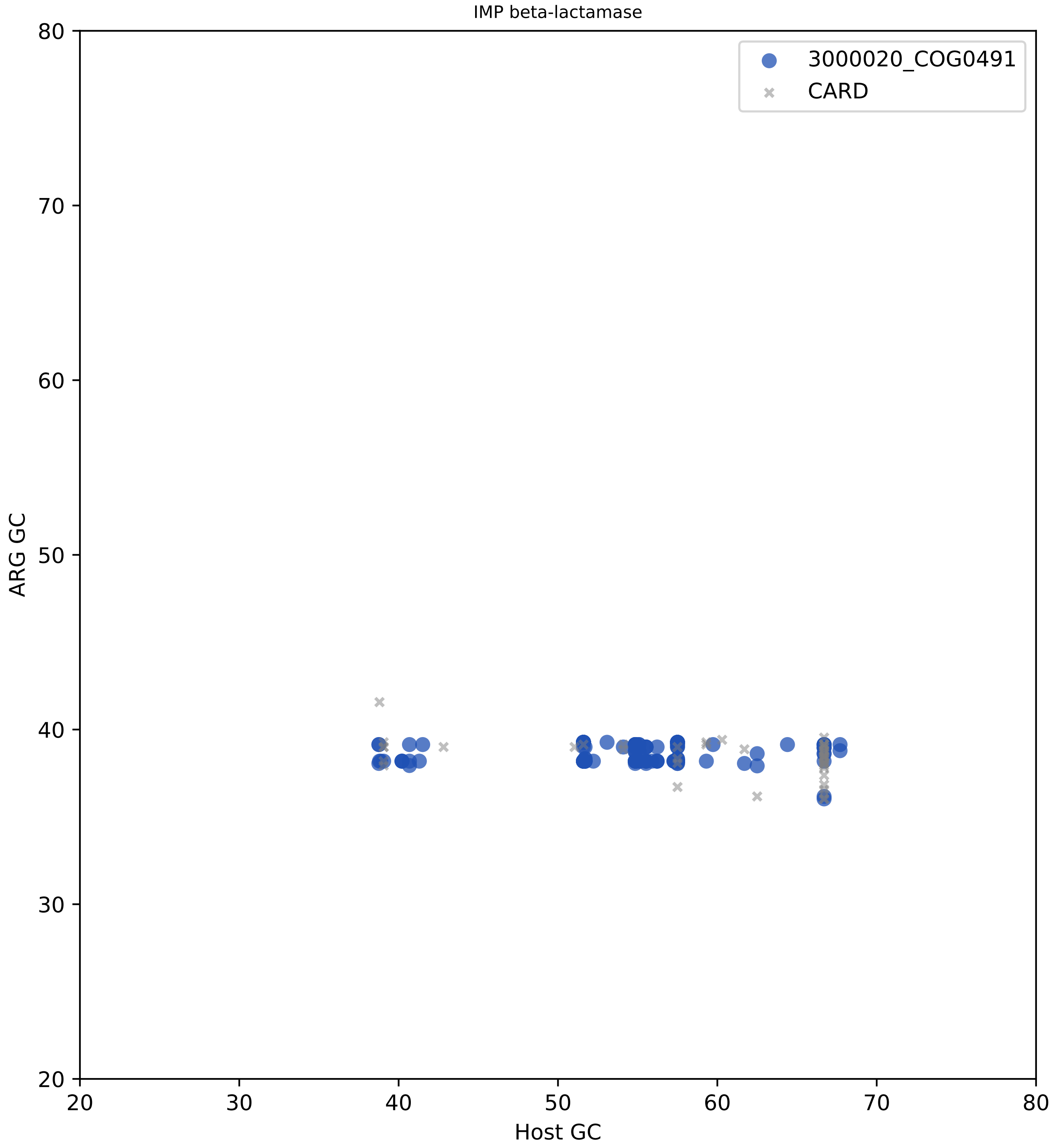

Supplementary Figure S1: (continued).

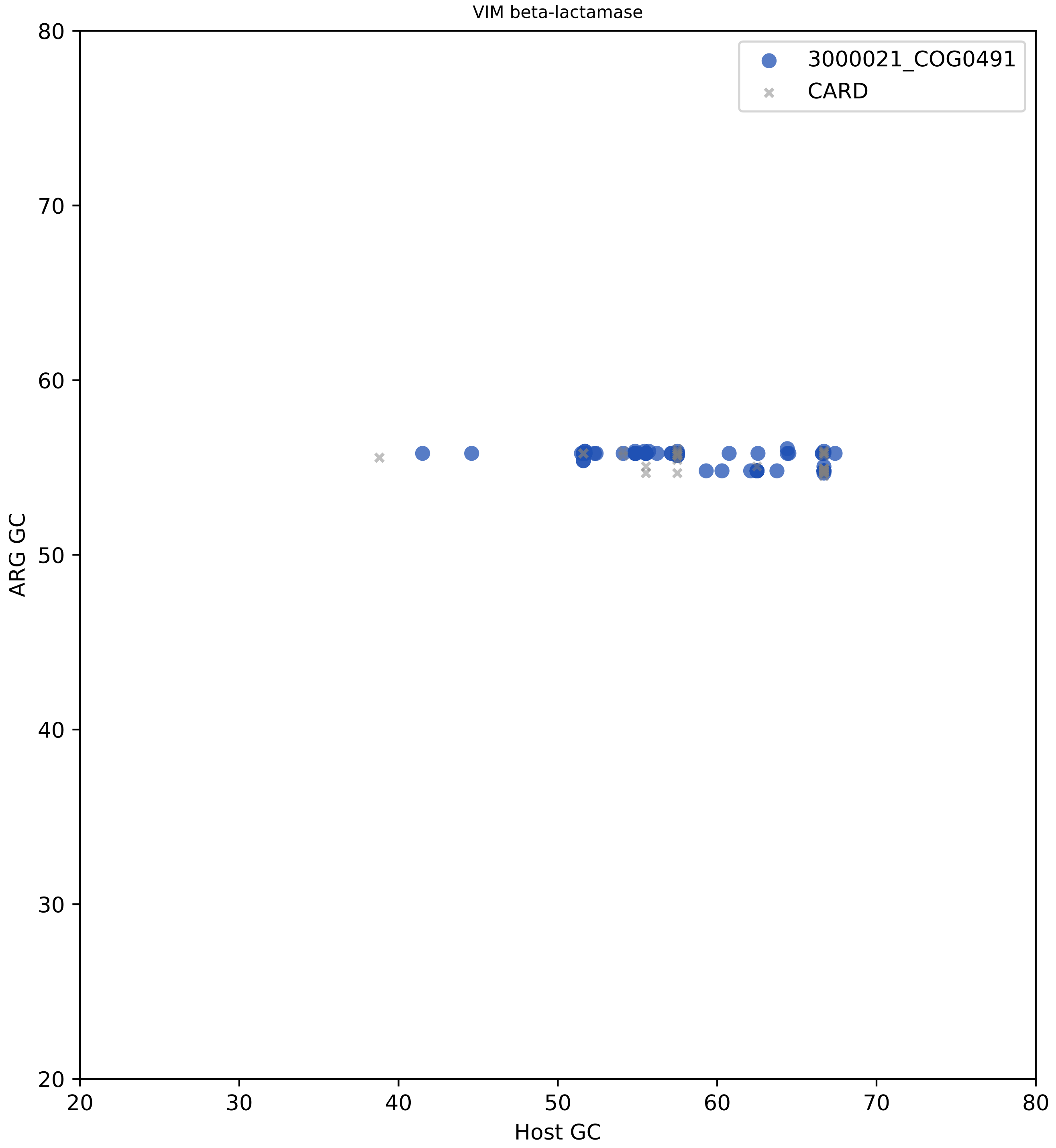

Supplementary Figure S1: (continued).

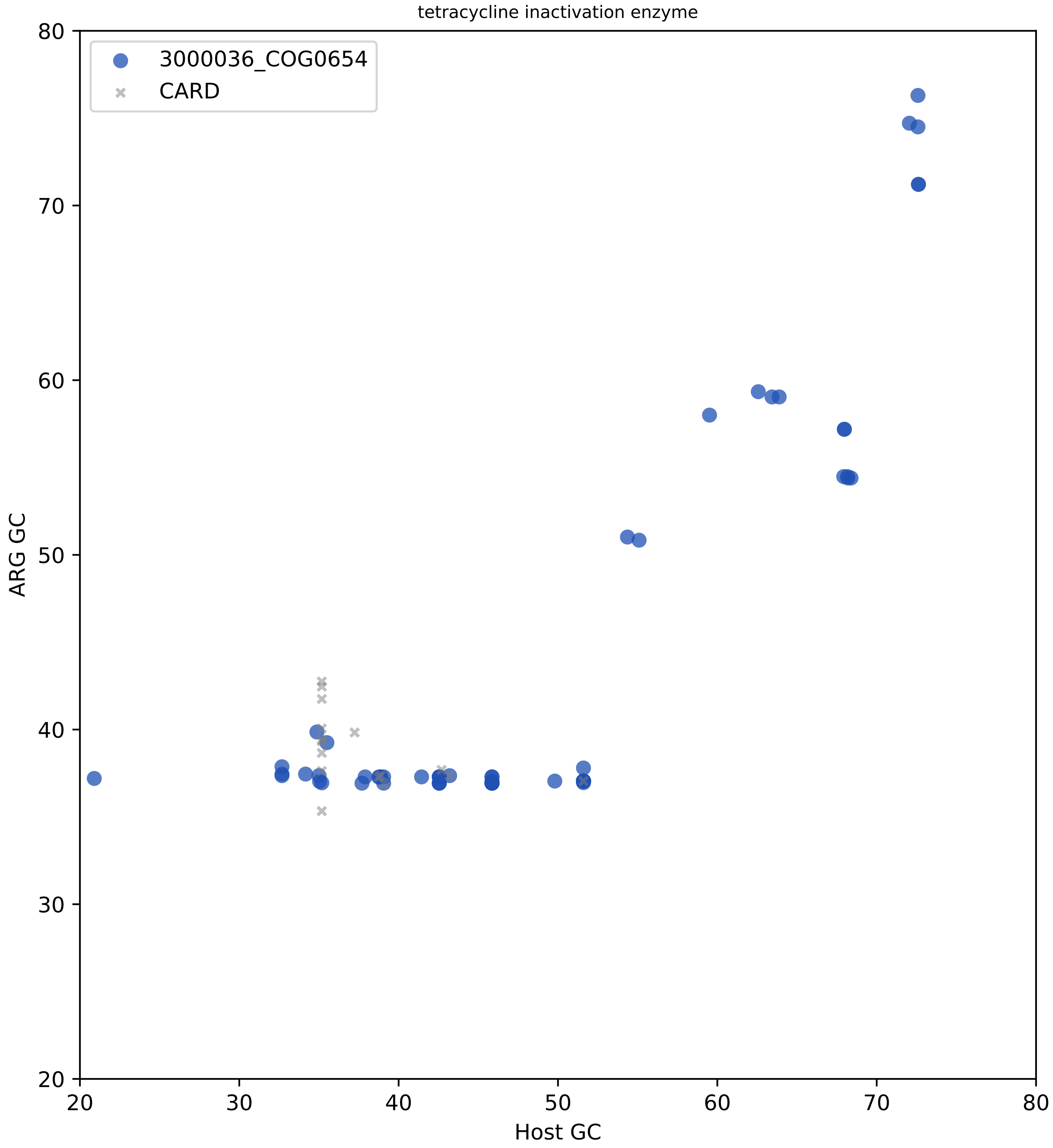

Supplementary Figure S1: (continued).

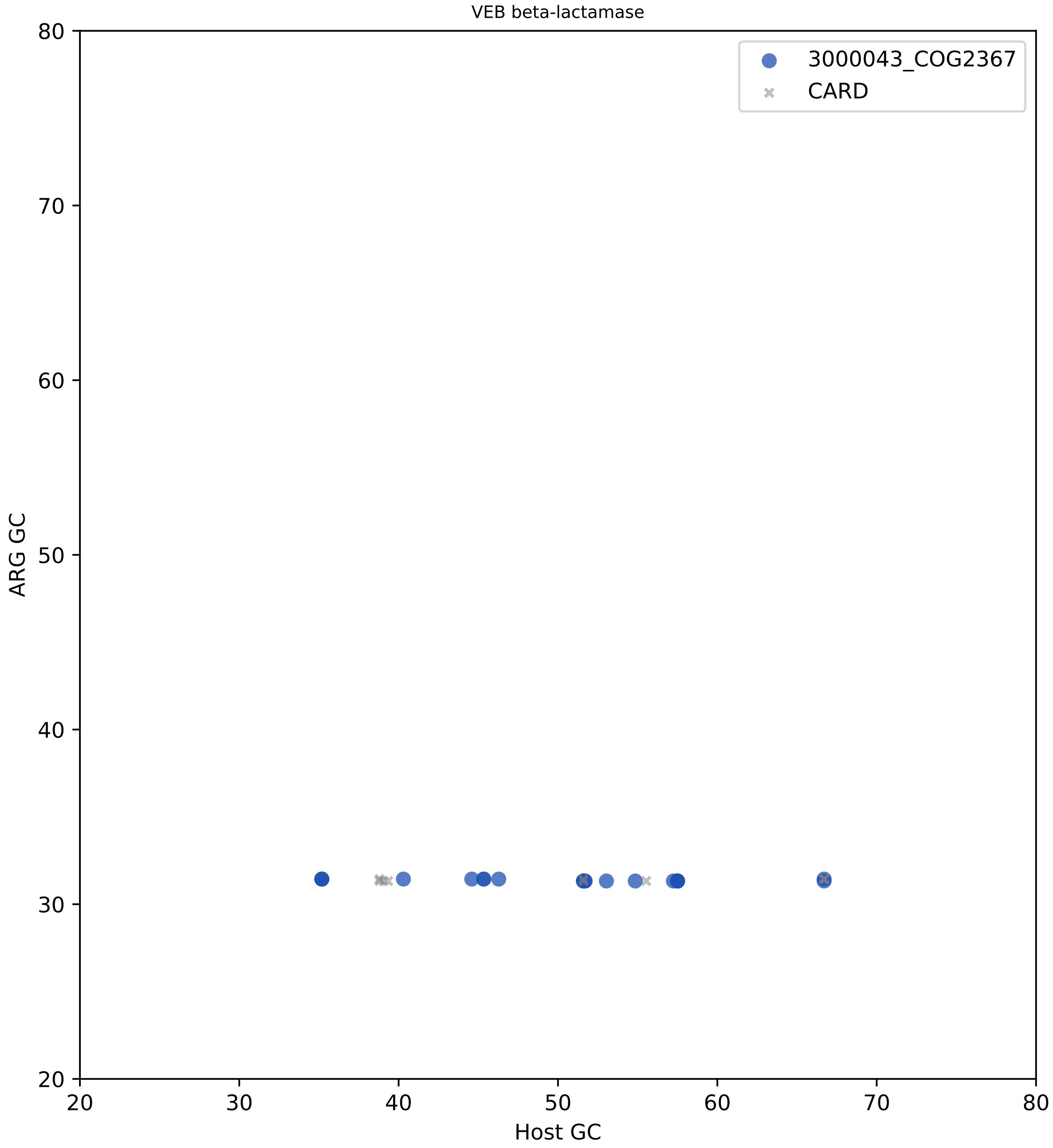

Supplementary Figure S1: (continued).

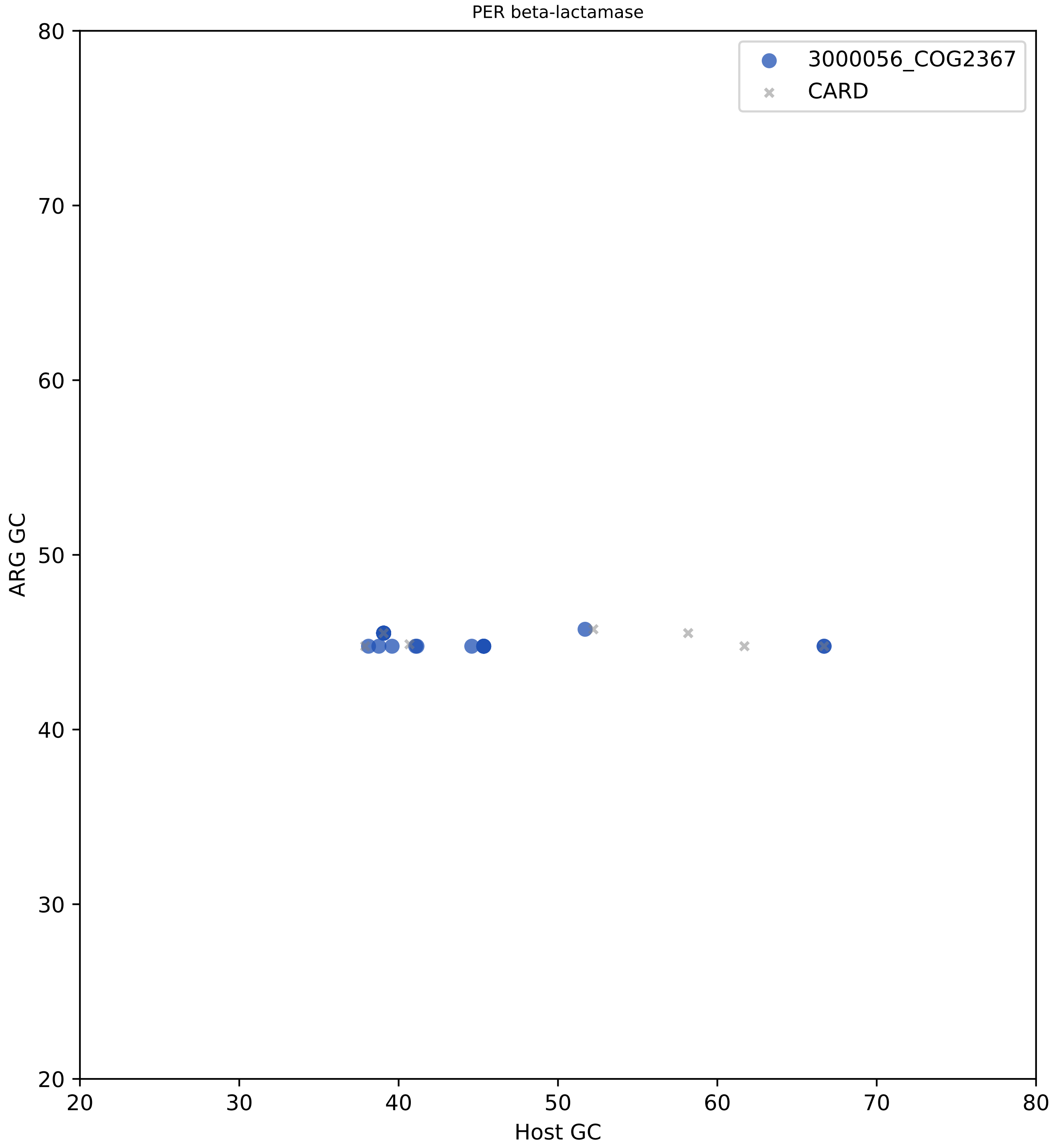

Supplementary Figure S1: (continued).

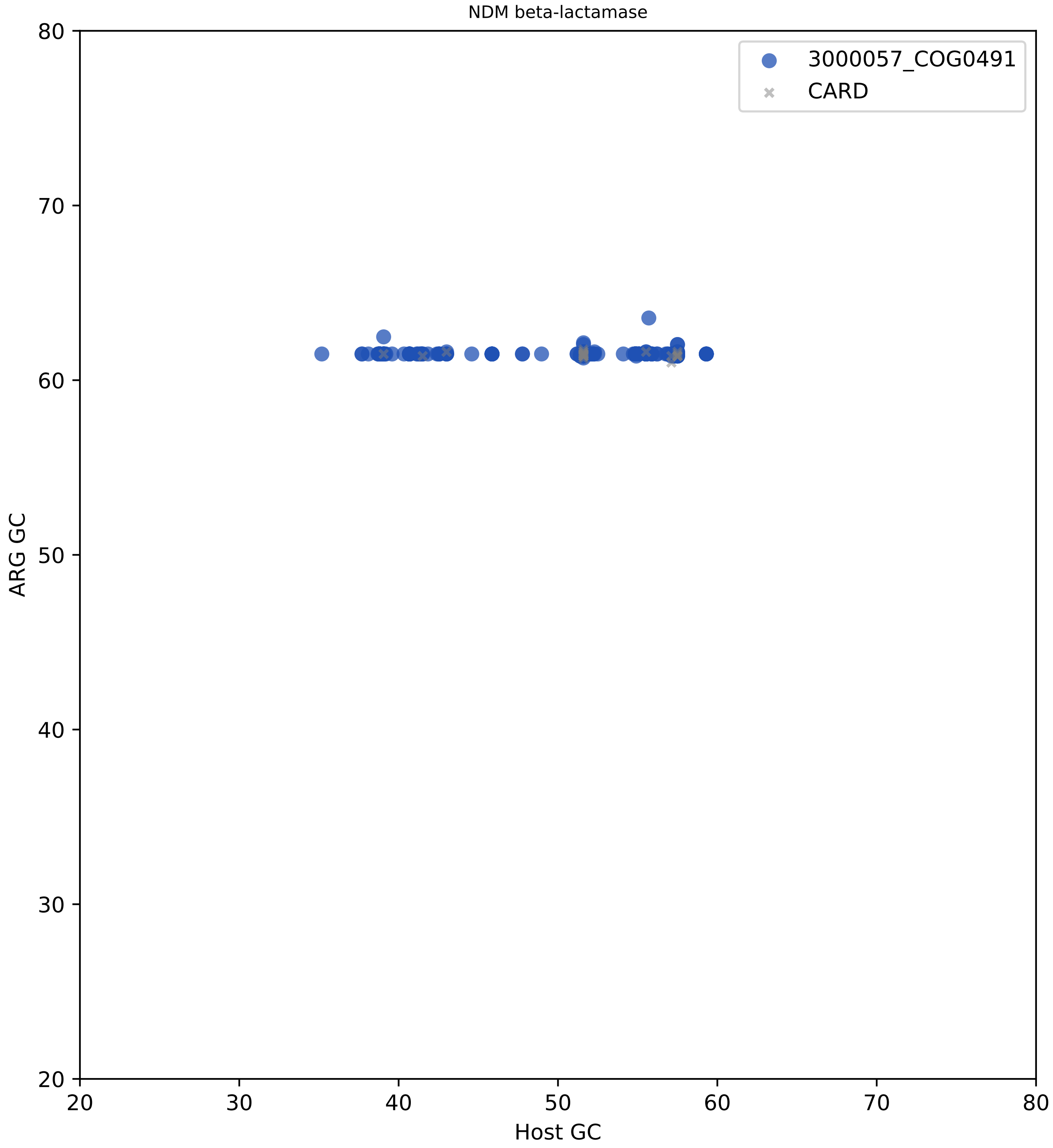

Supplementary Figure S1: (continued).

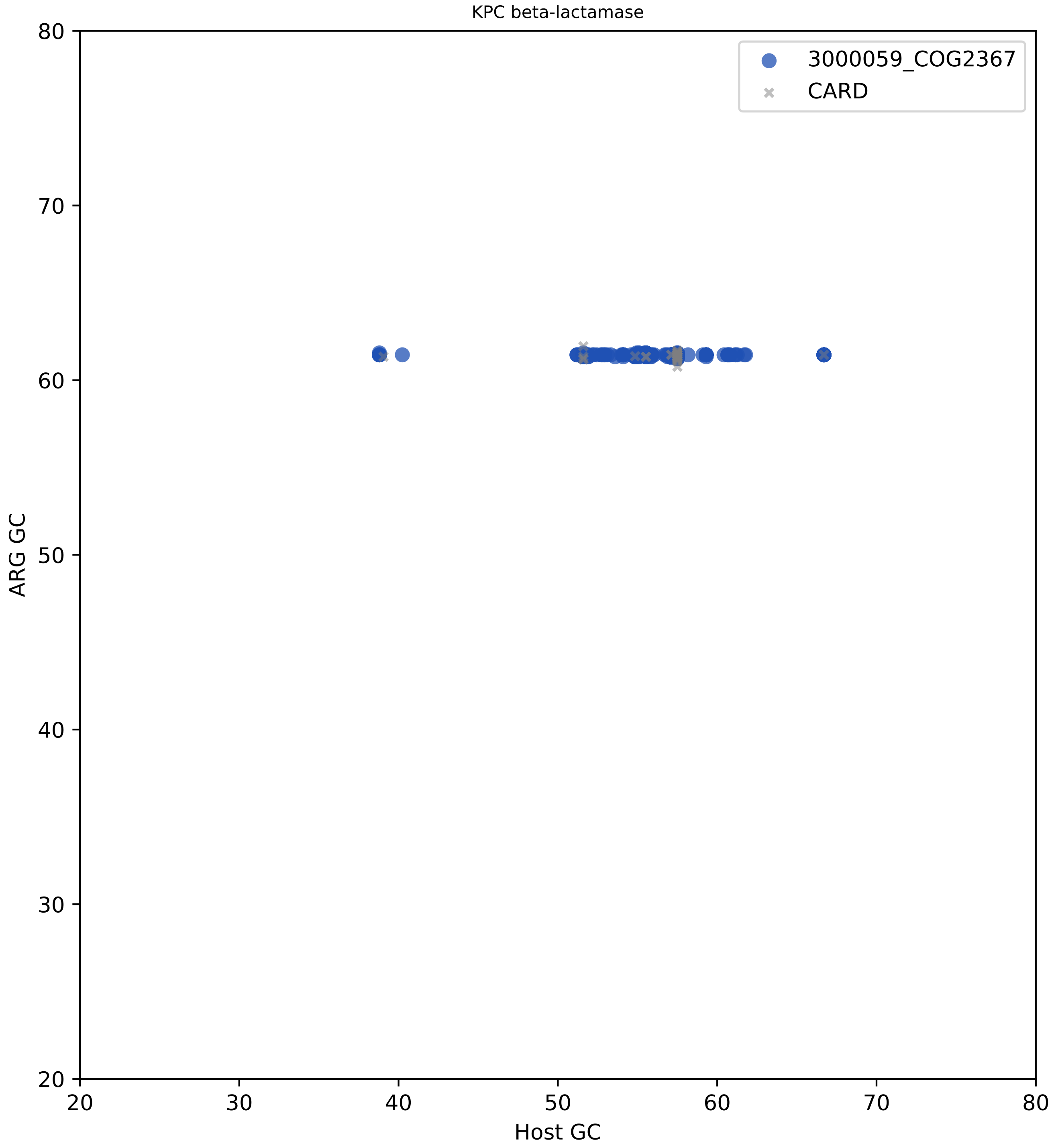

Supplementary Figure S1: (continued).

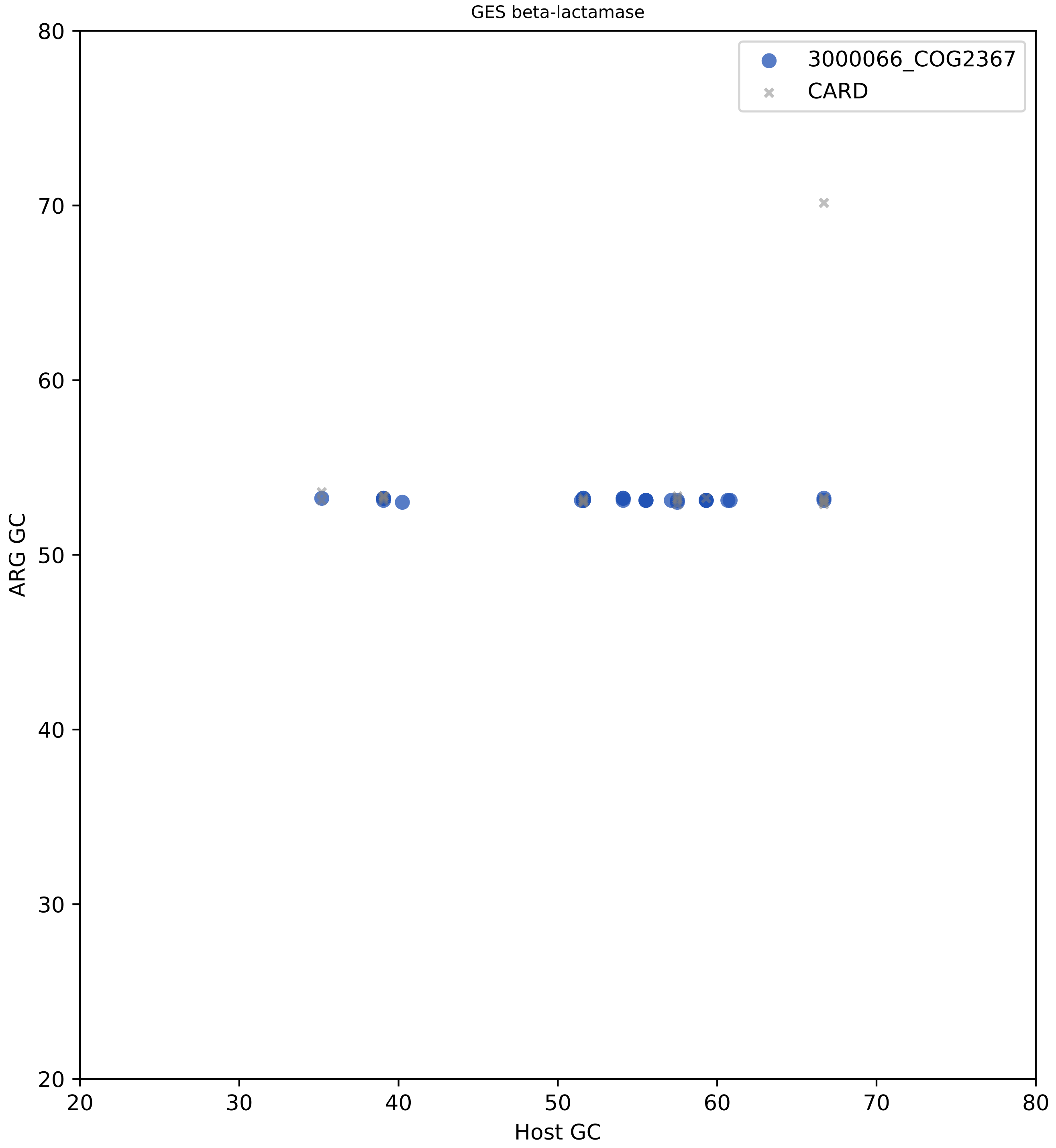

Supplementary Figure S1: (continued).

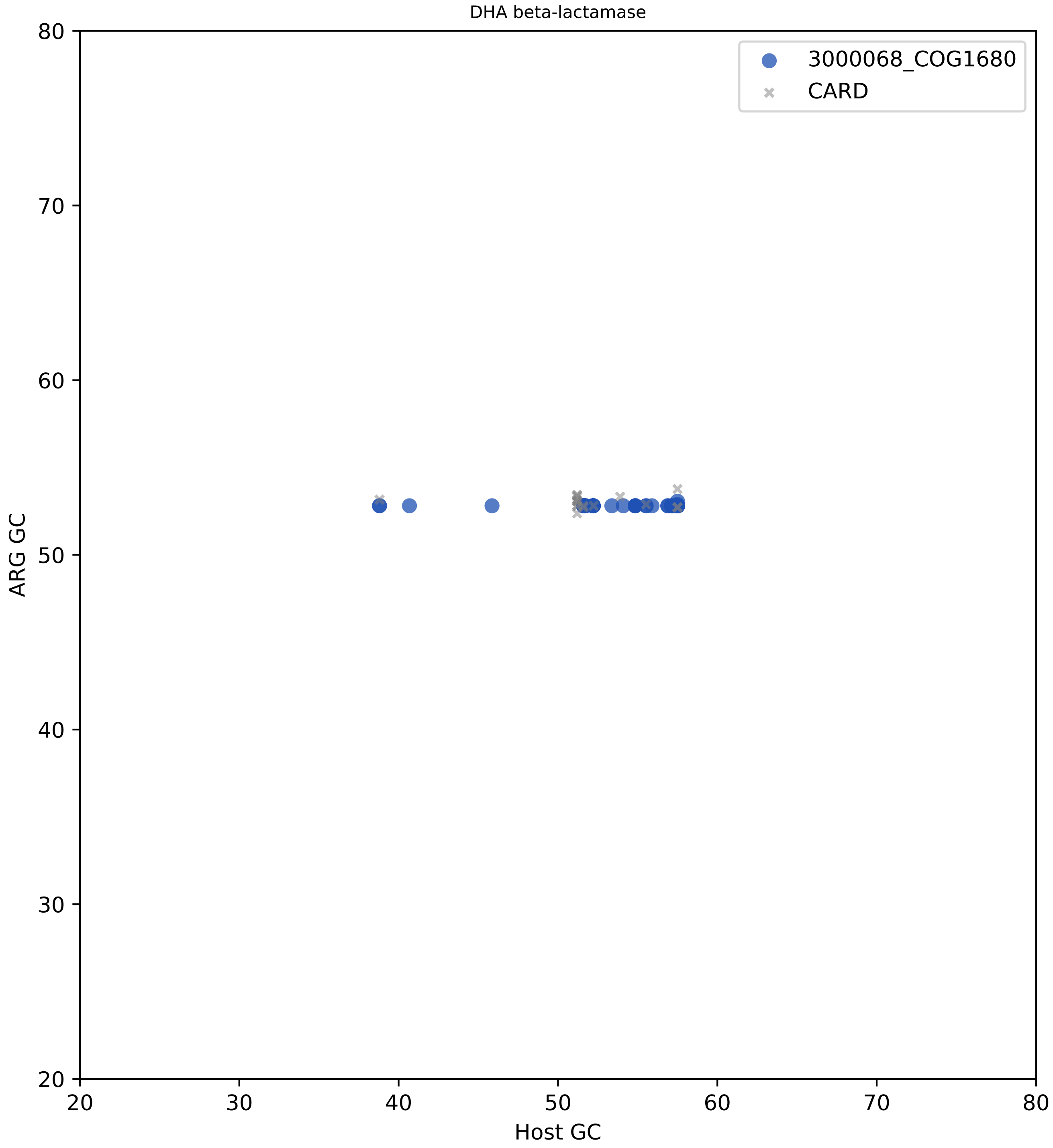

Supplementary Figure S1: (continued).

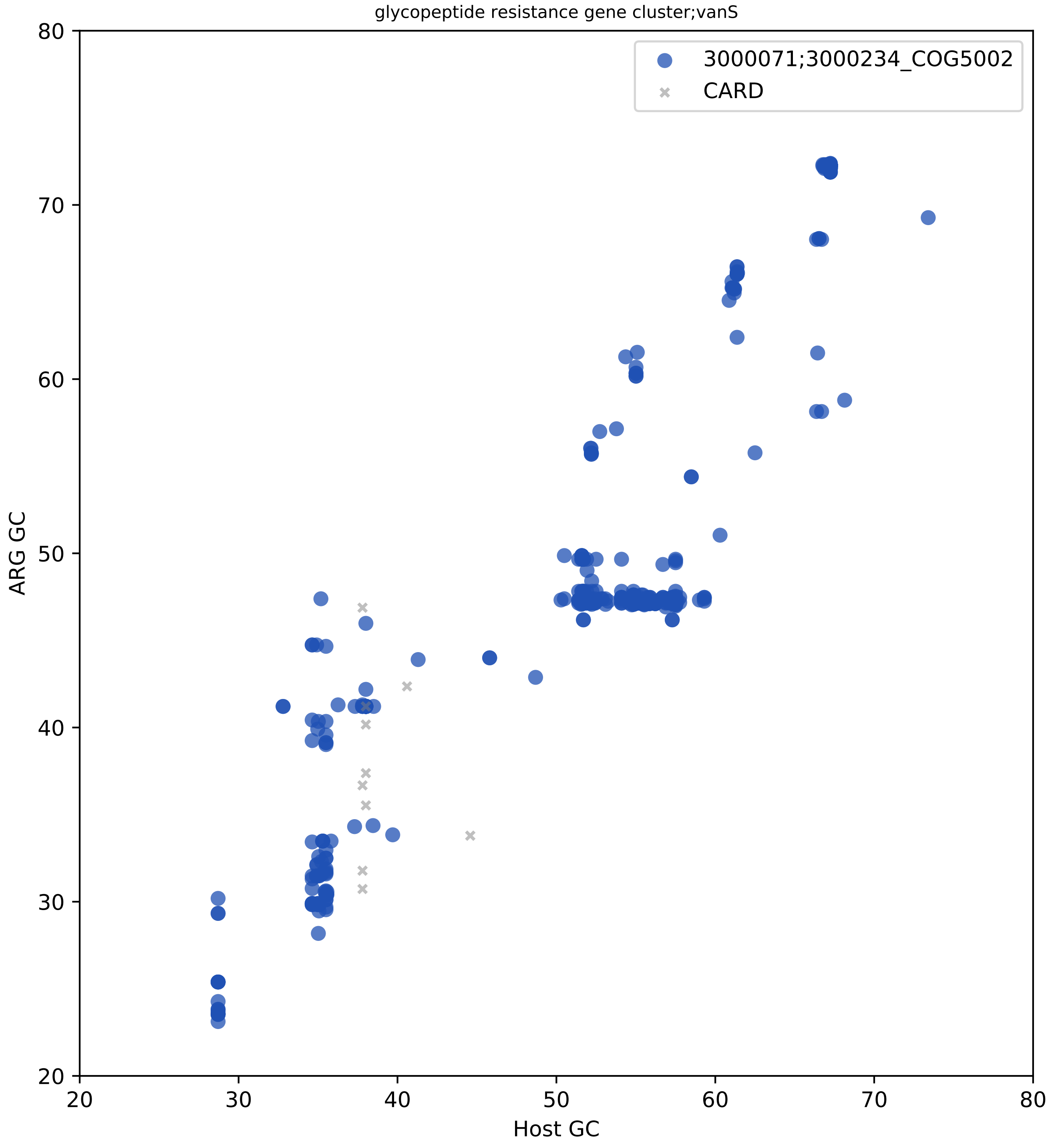

Supplementary Figure S1: (continued).

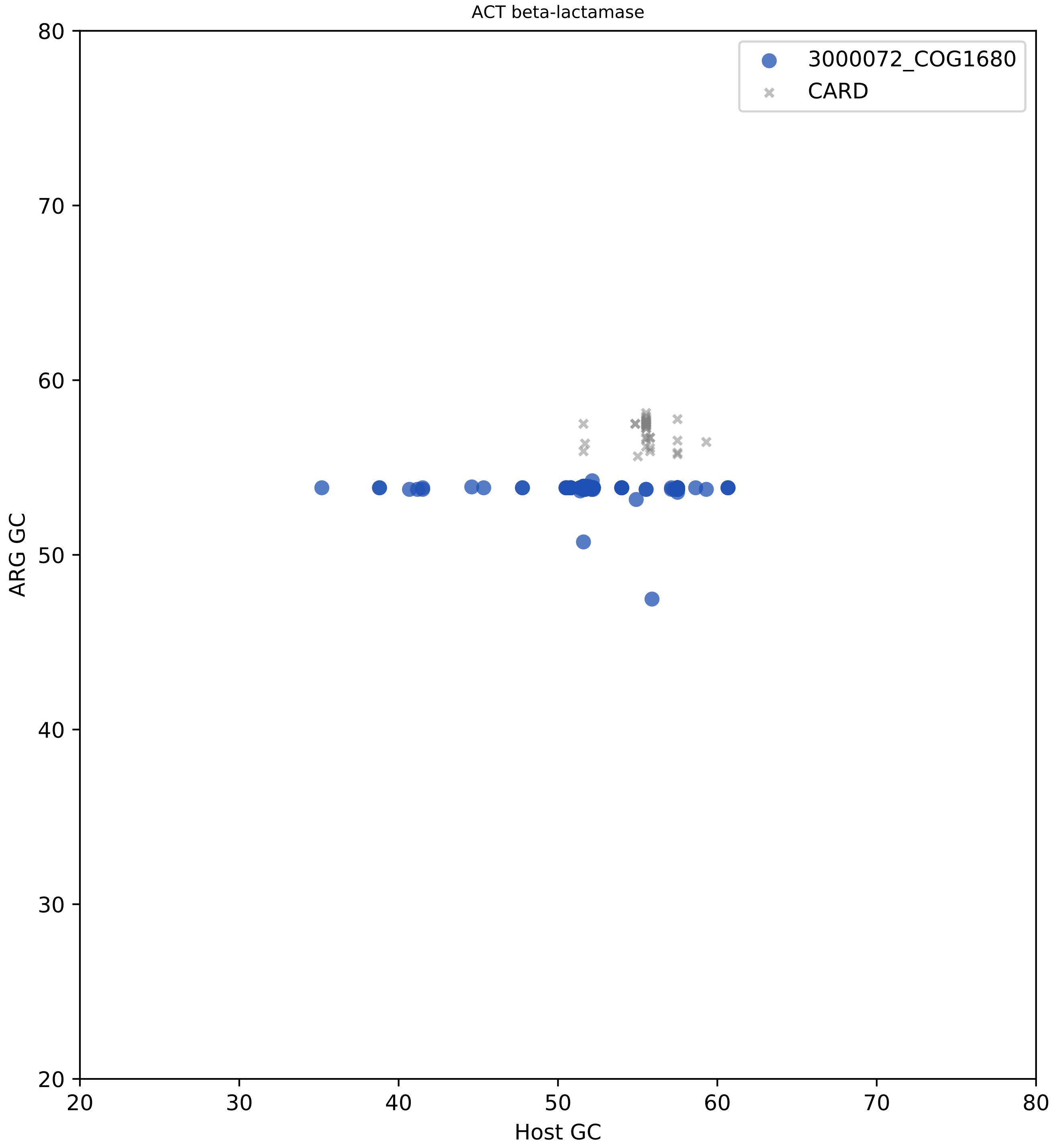

Supplementary Figure S1: (continued).

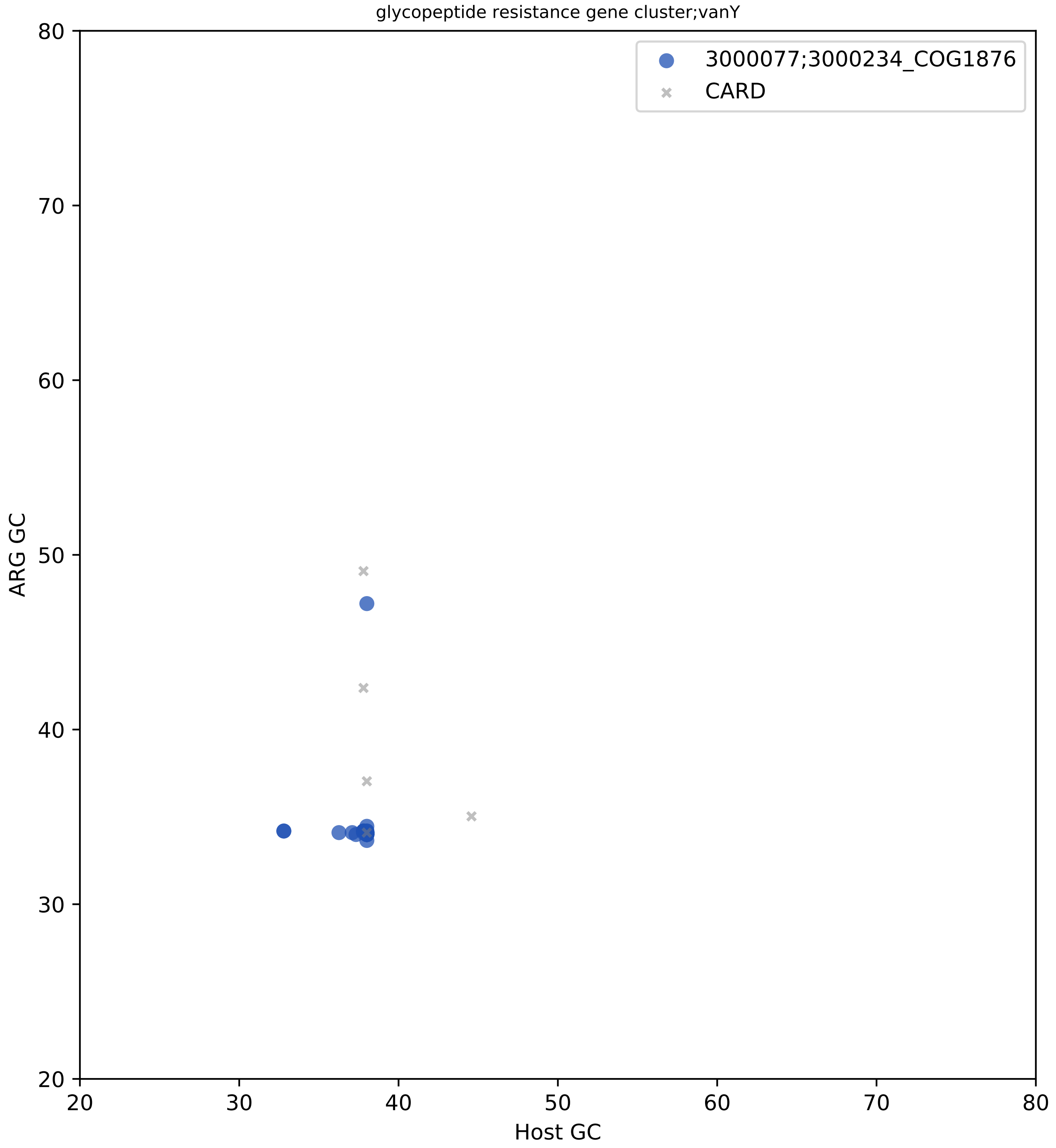

Supplementary Figure S1: (continued).

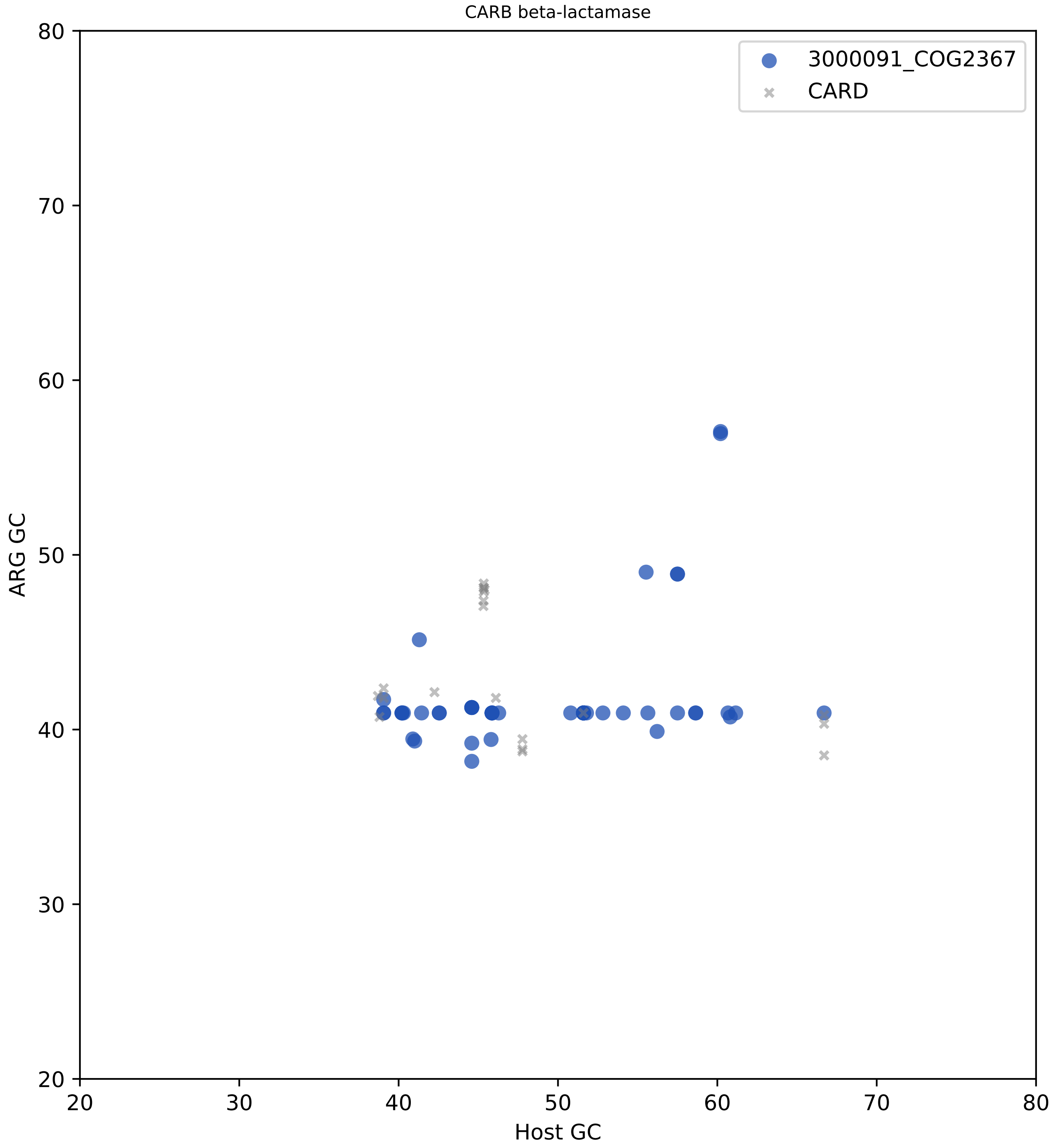

Supplementary Figure S1: (continued).

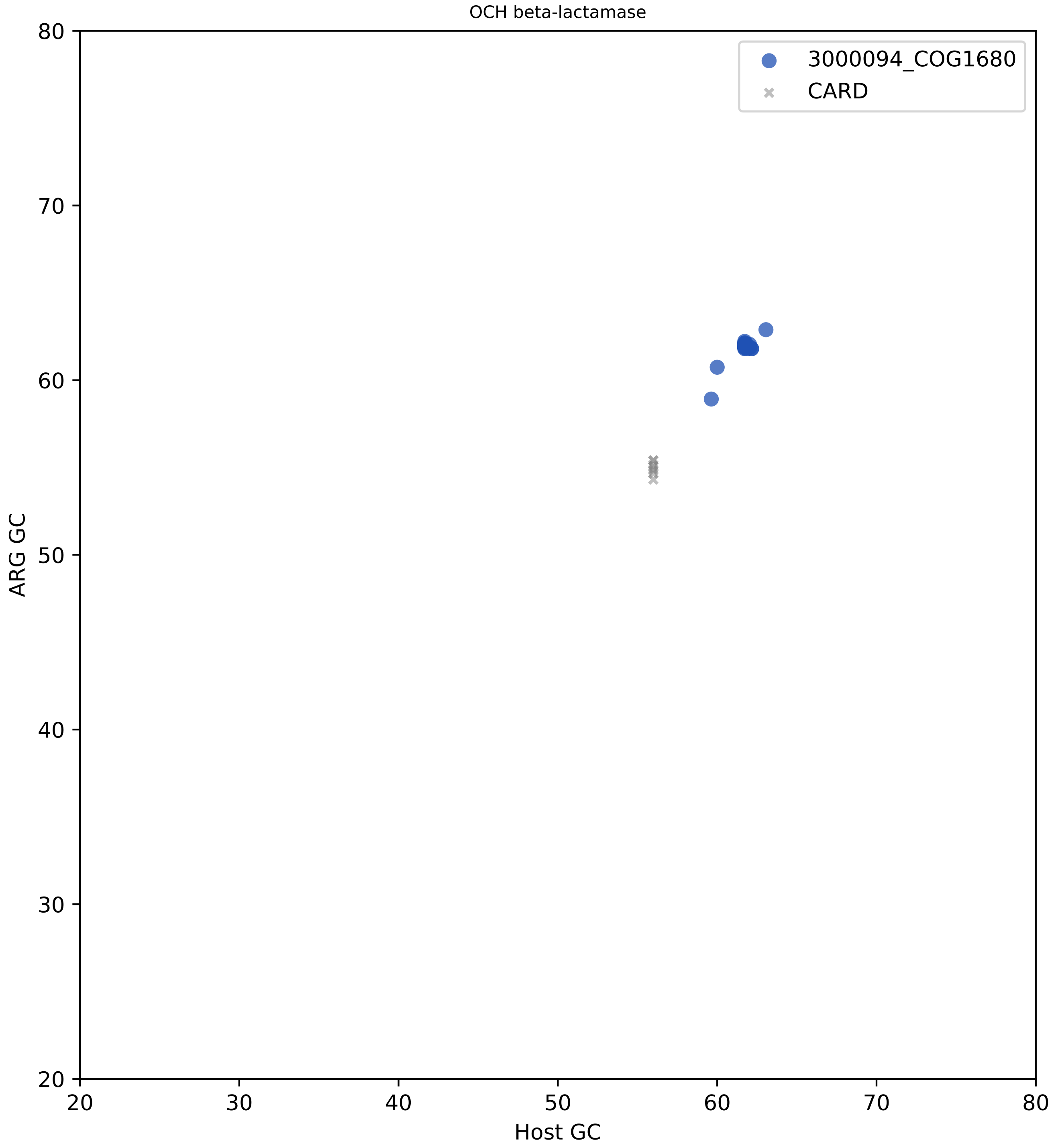

Supplementary Figure S1: (continued).

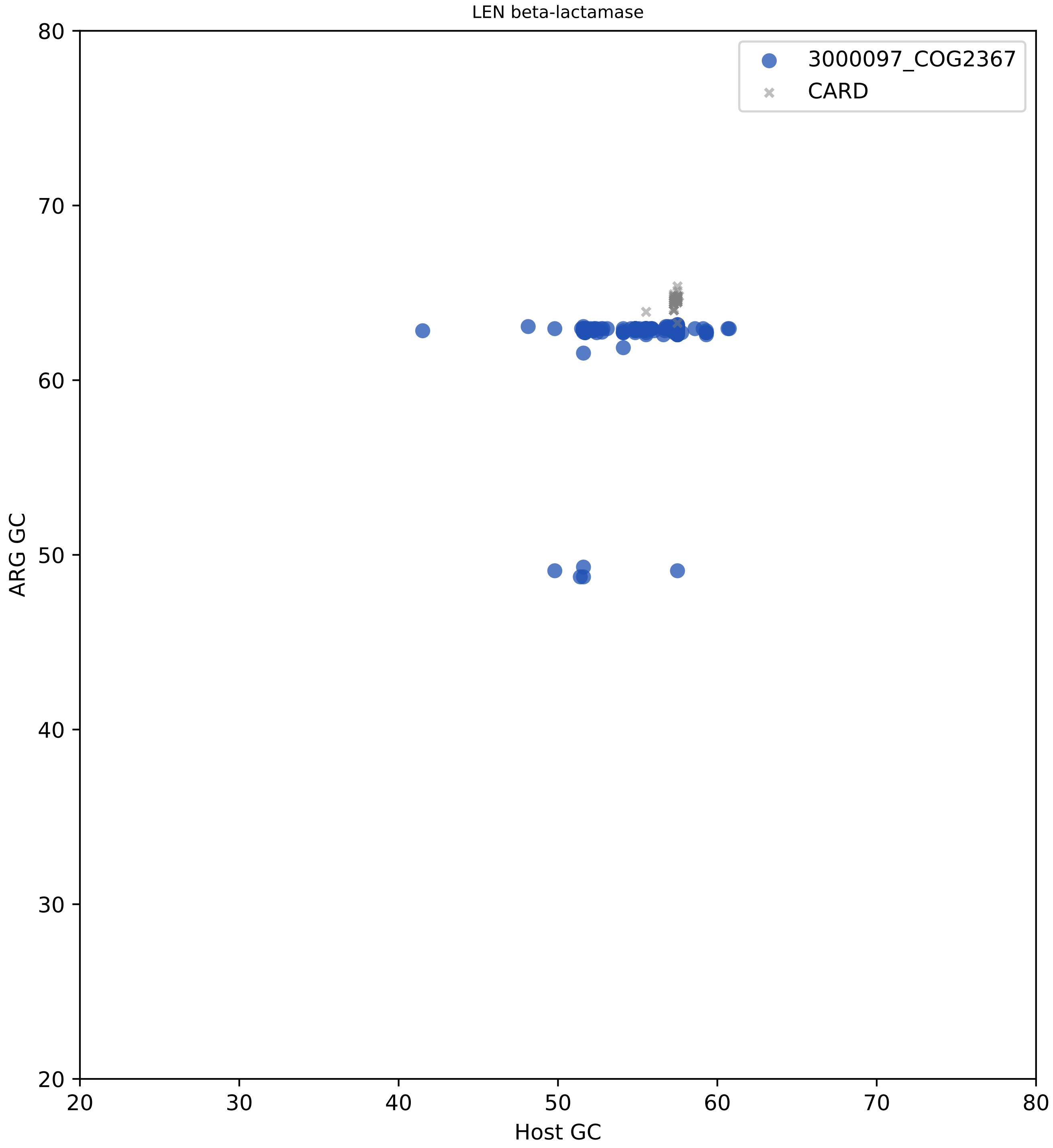

Supplementary Figure S1: (continued).

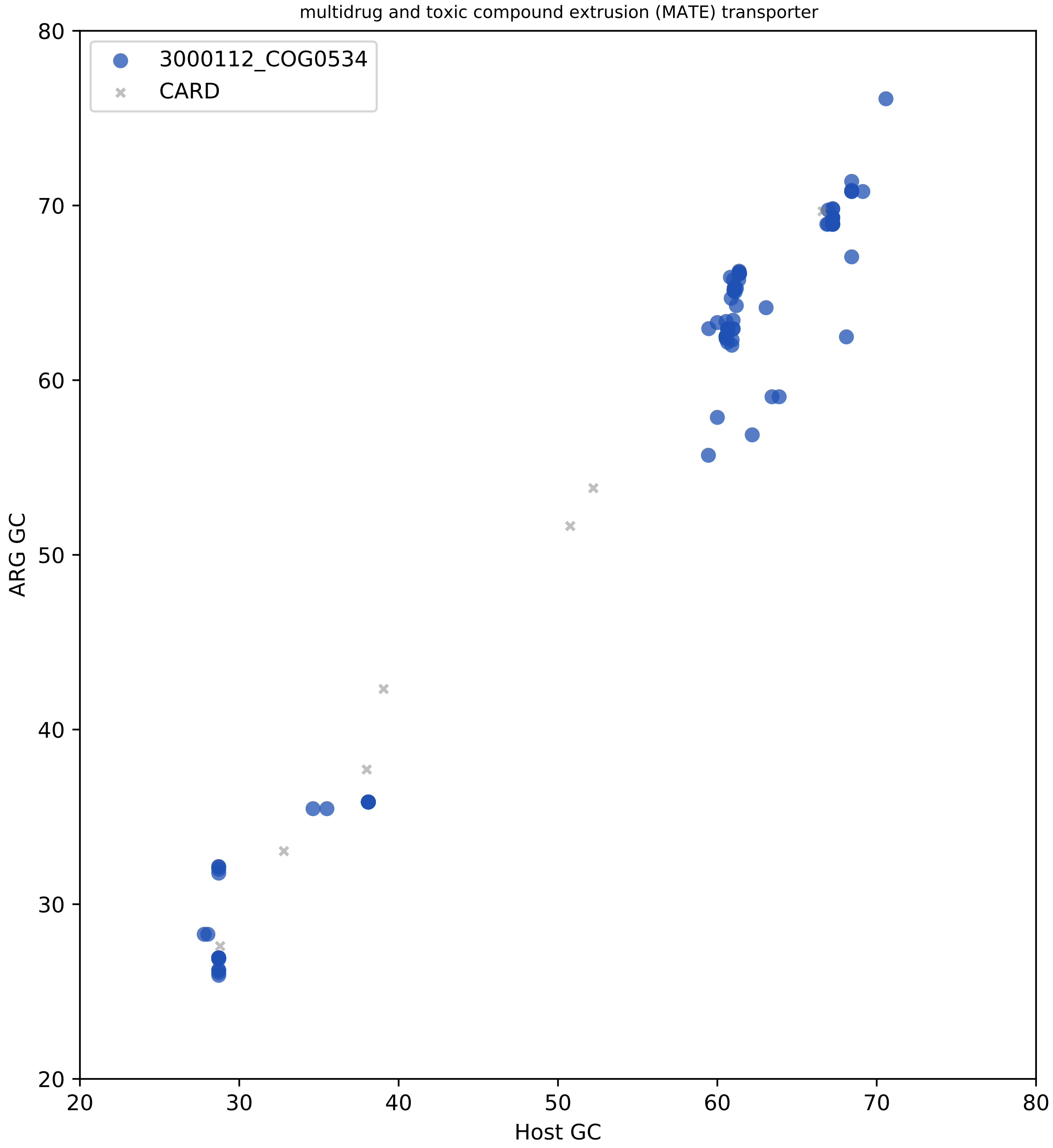

Supplementary Figure S1: (continued).

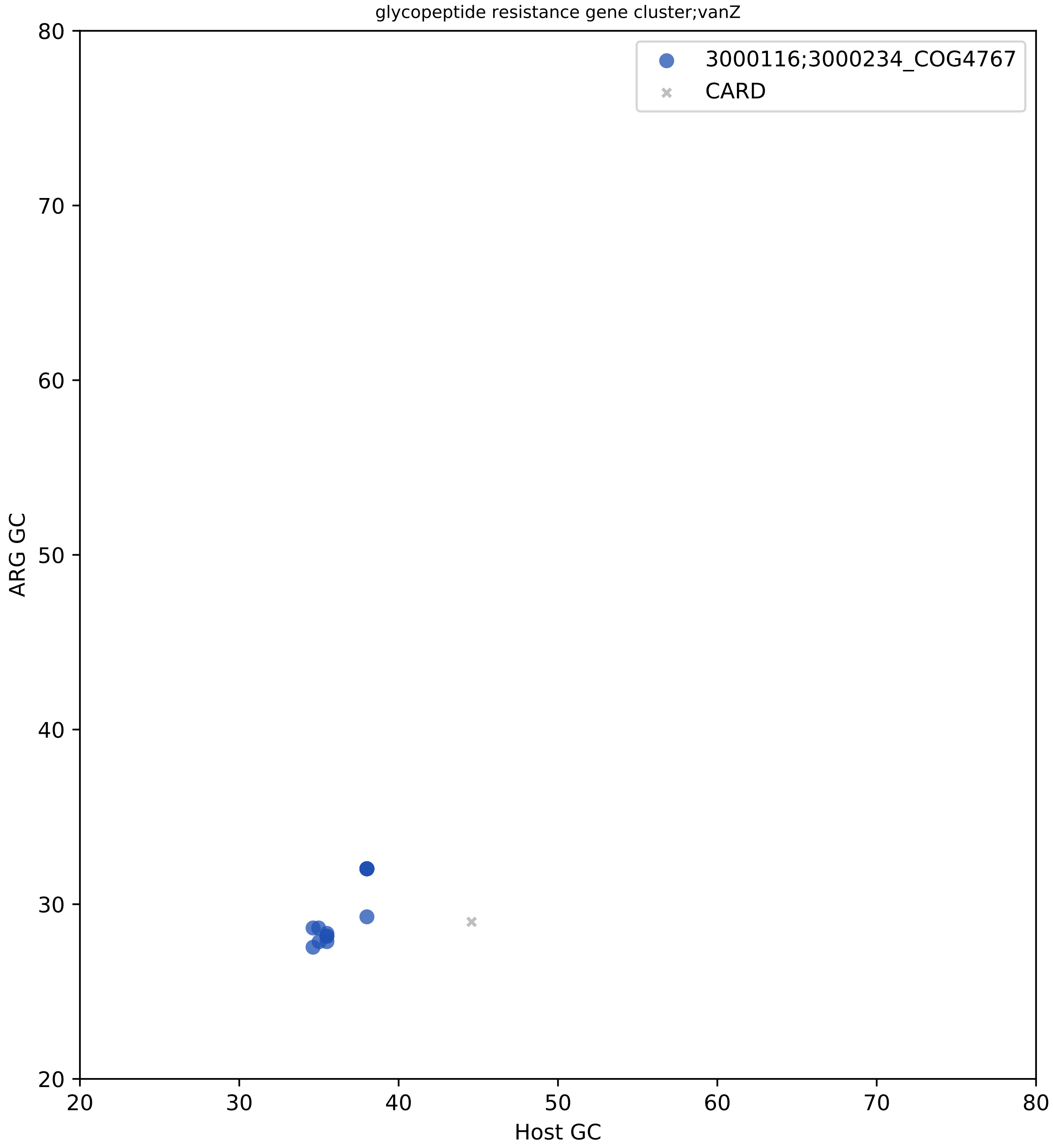

Supplementary Figure S1: (continued).

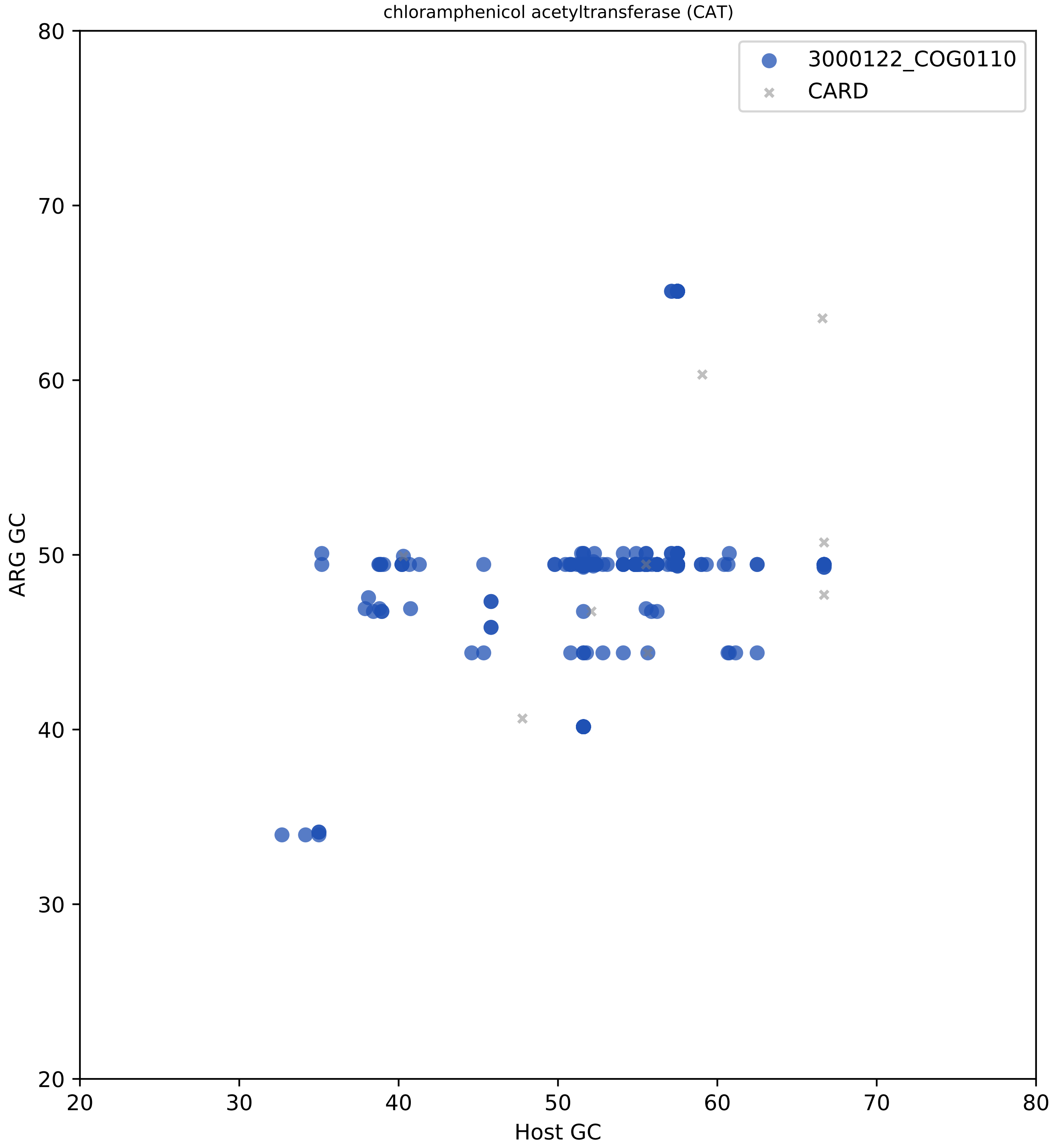

Supplementary Figure S1: (continued).

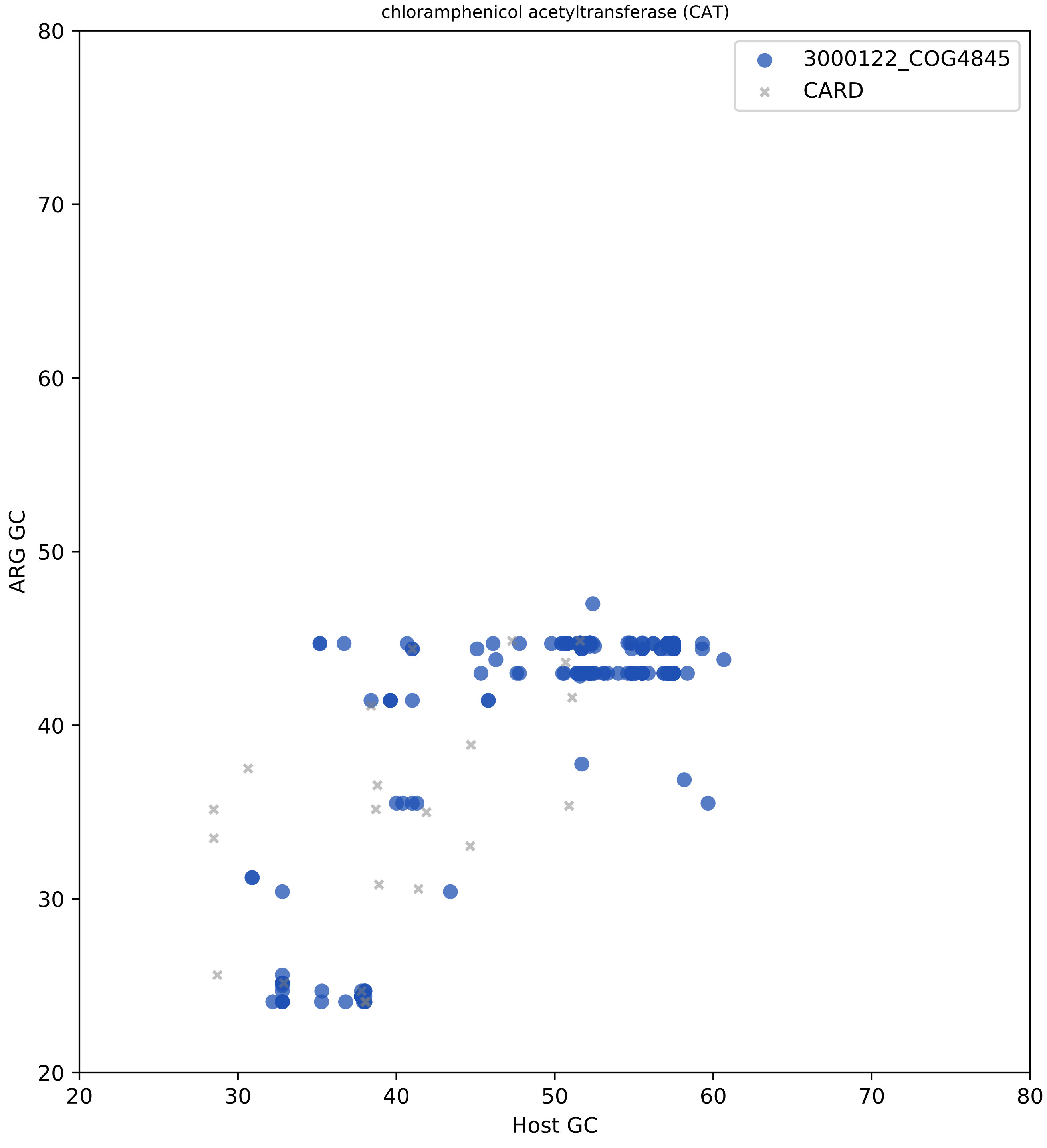

Supplementary Figure S1: (continued).

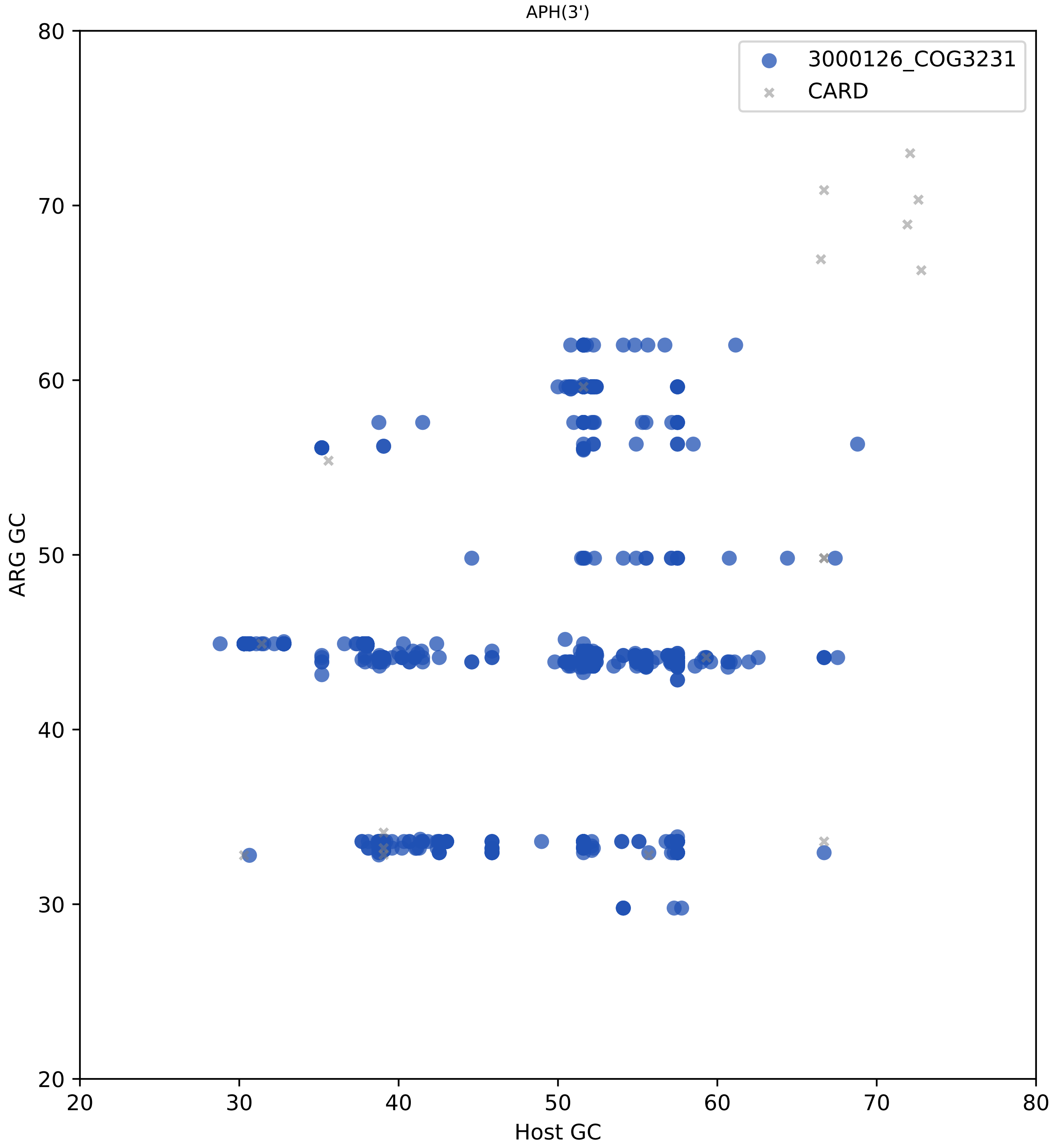

Supplementary Figure S1: (continued).

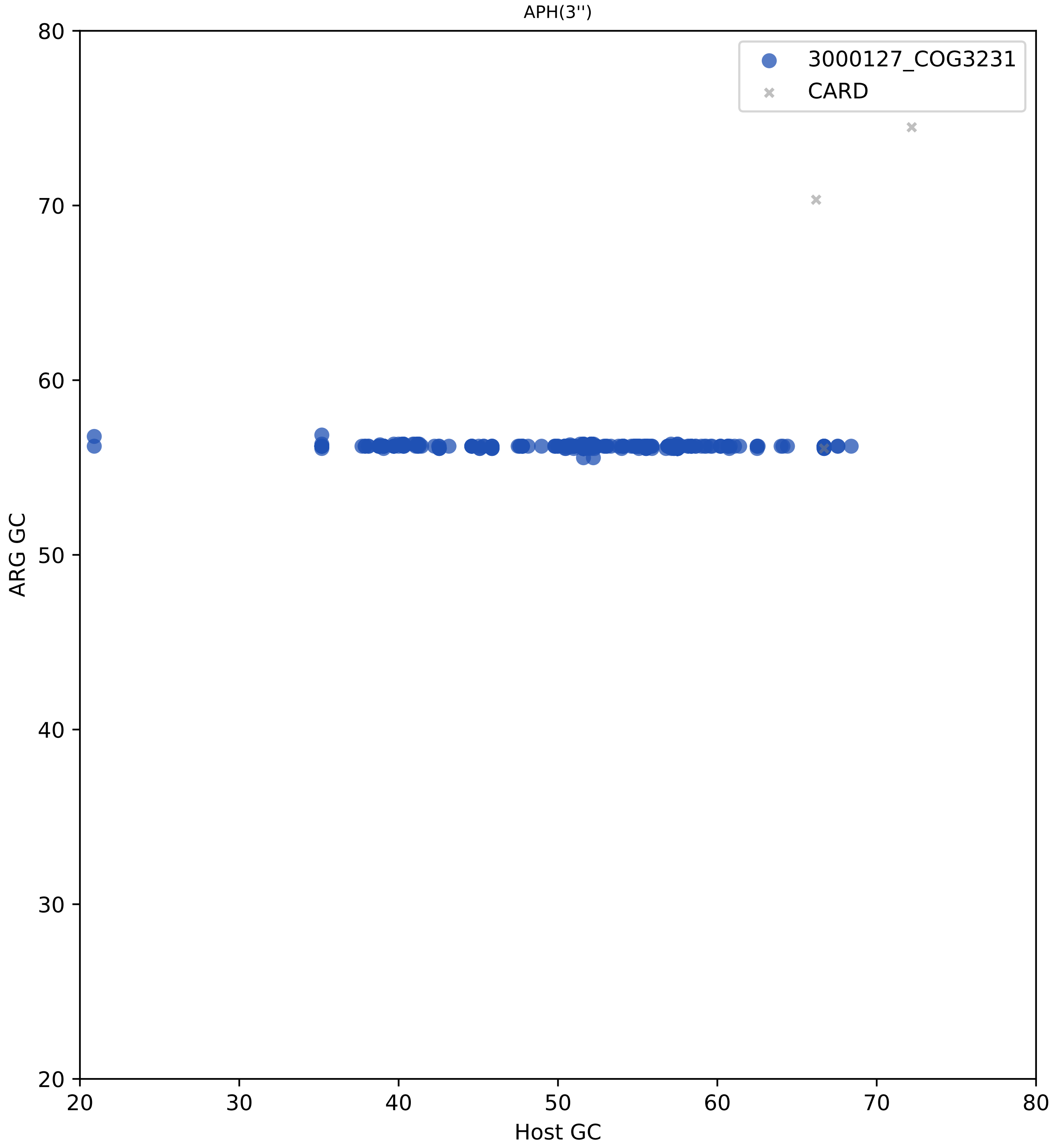

Supplementary Figure S1: (continued).

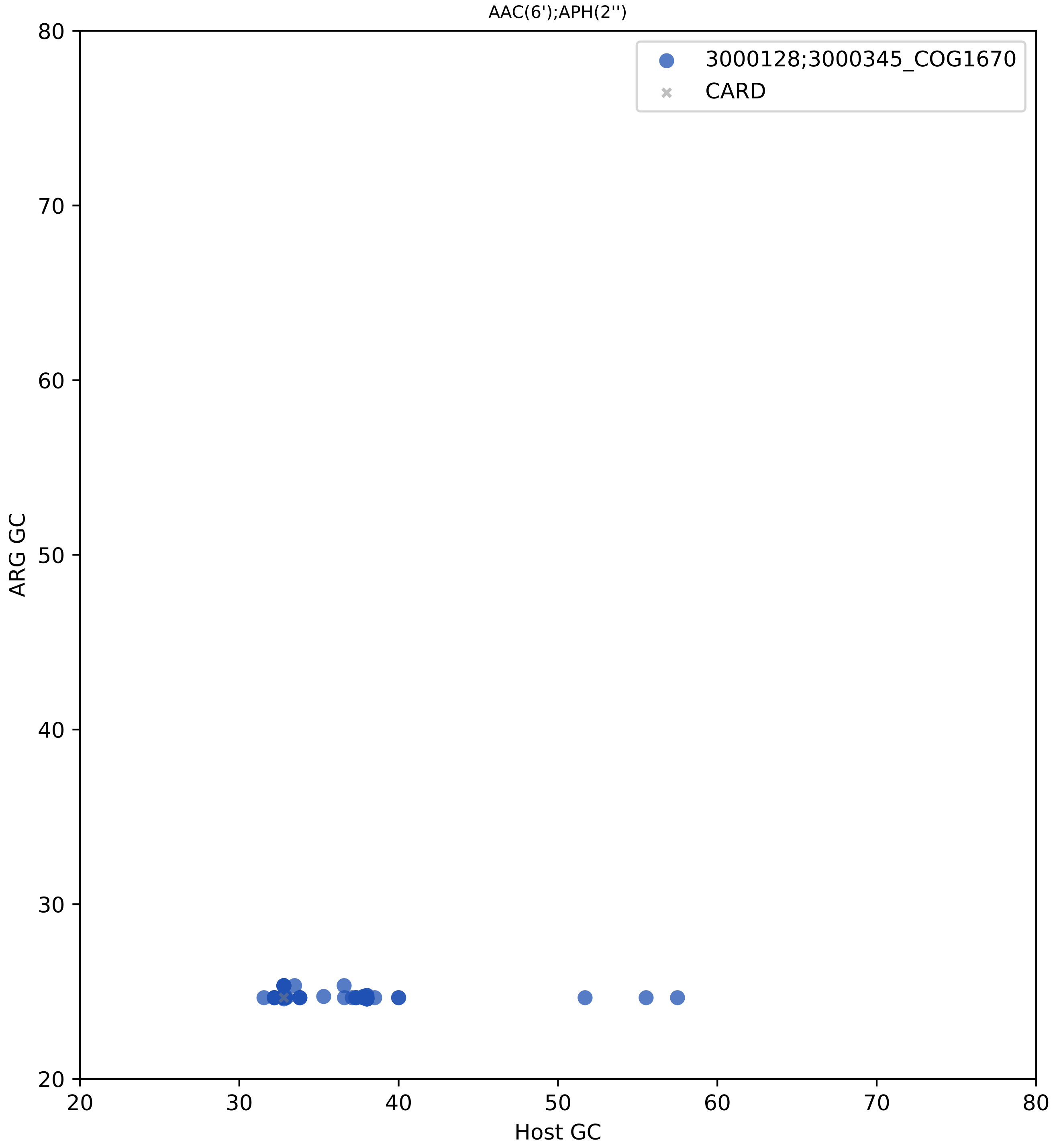

Supplementary Figure S1: (continued).

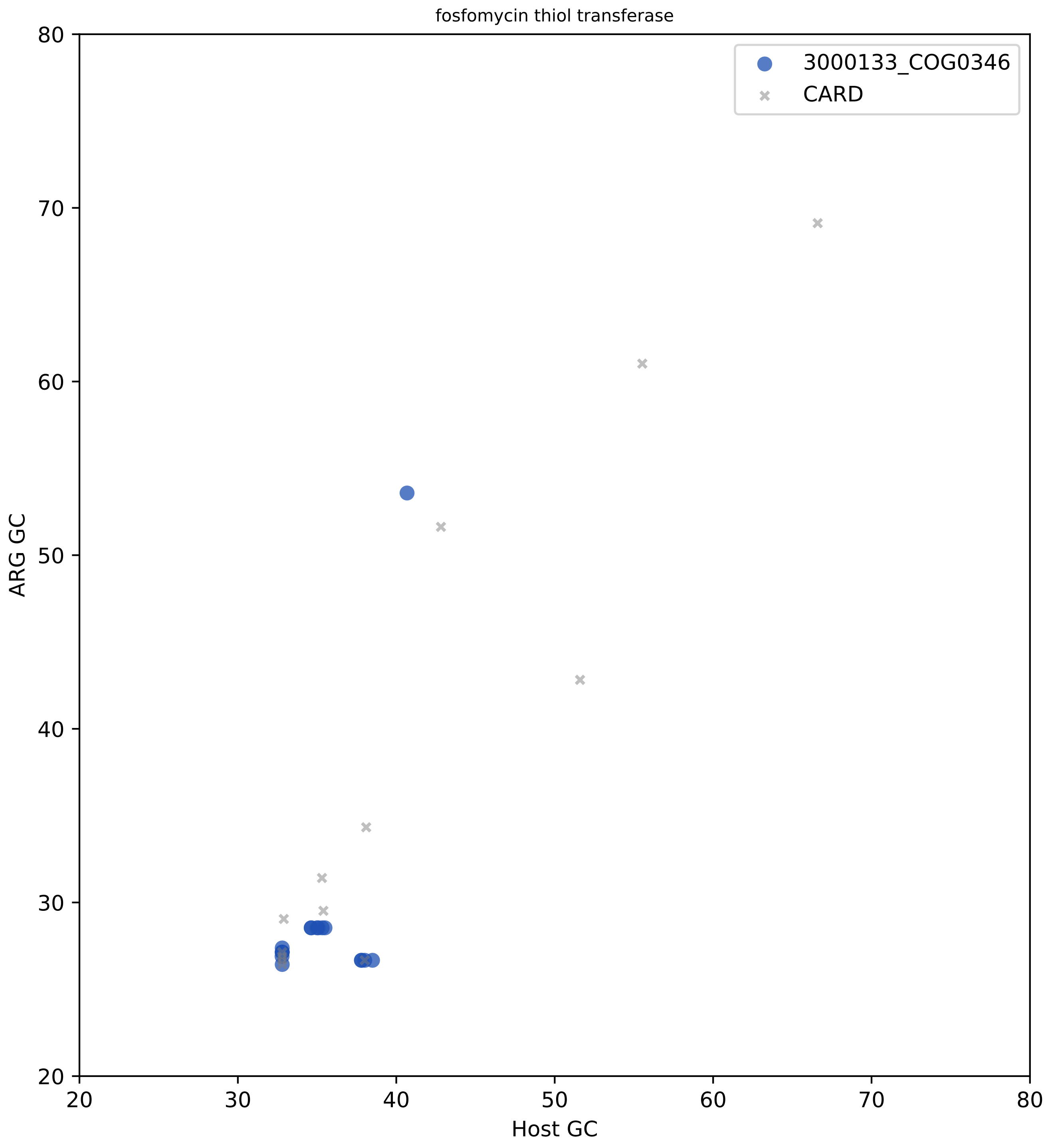

Supplementary Figure S1: (continued).

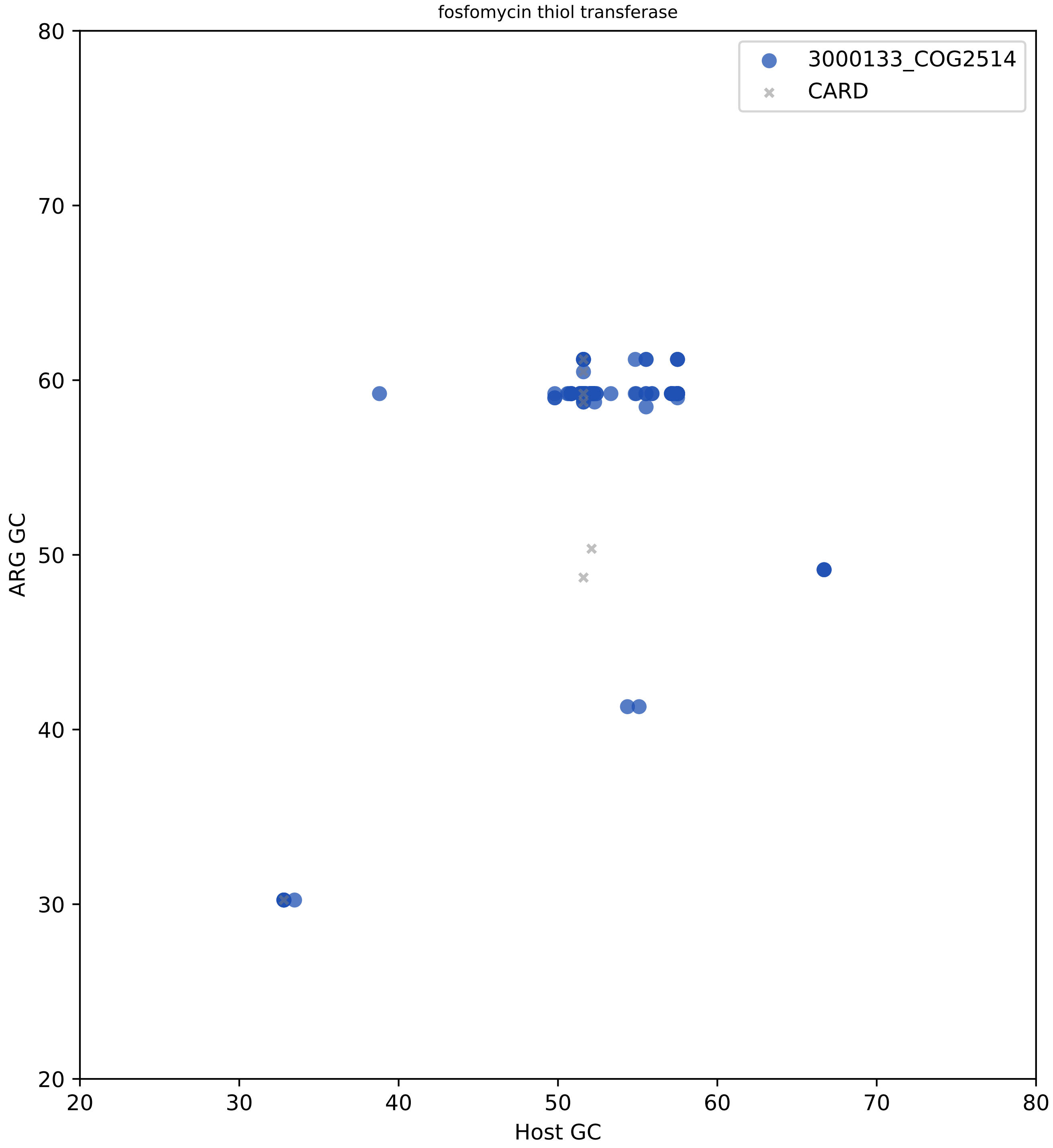

Supplementary Figure S1: (continued).

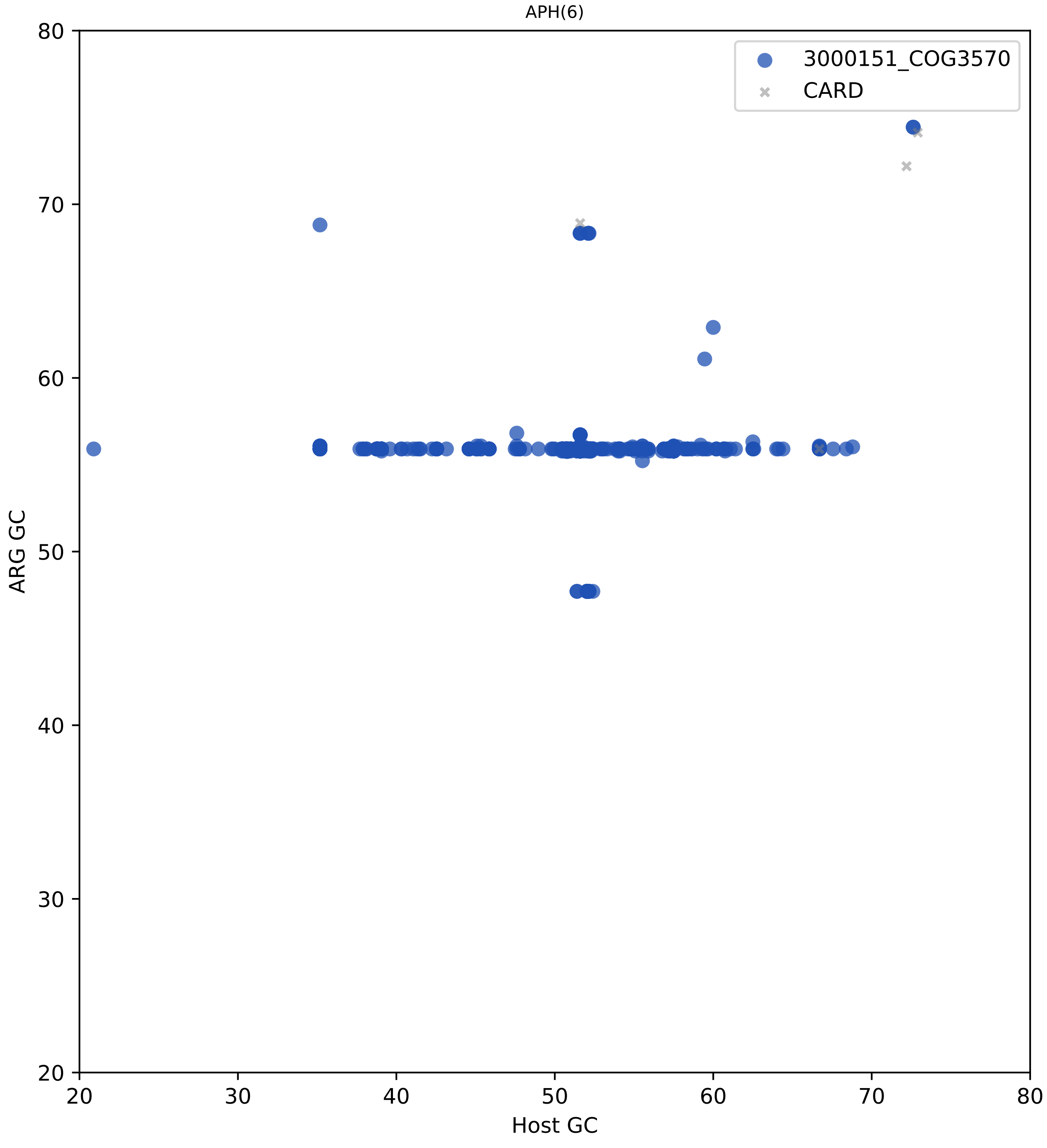

Supplementary Figure S1: (continued).

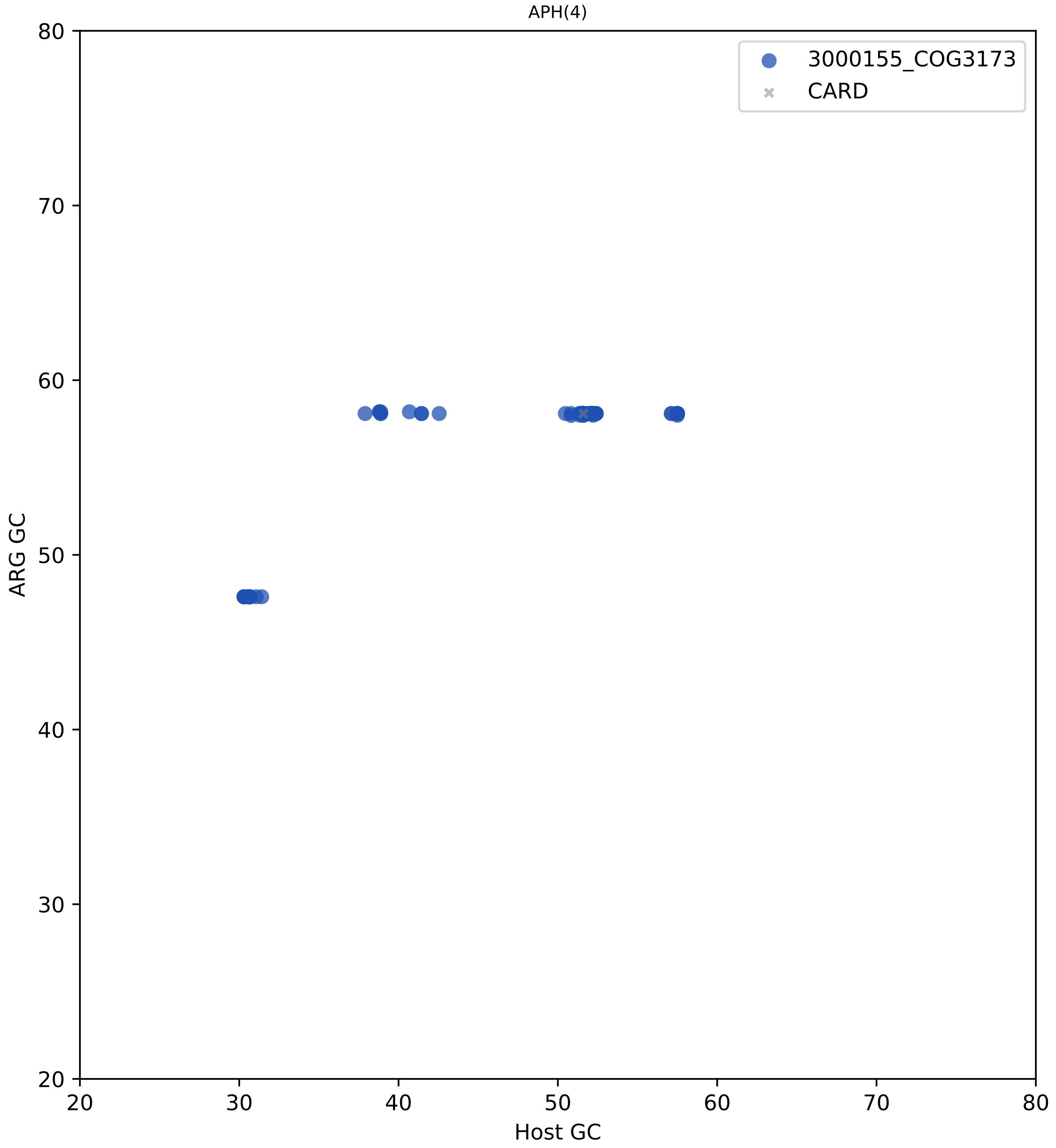

Supplementary Figure S1: (continued).

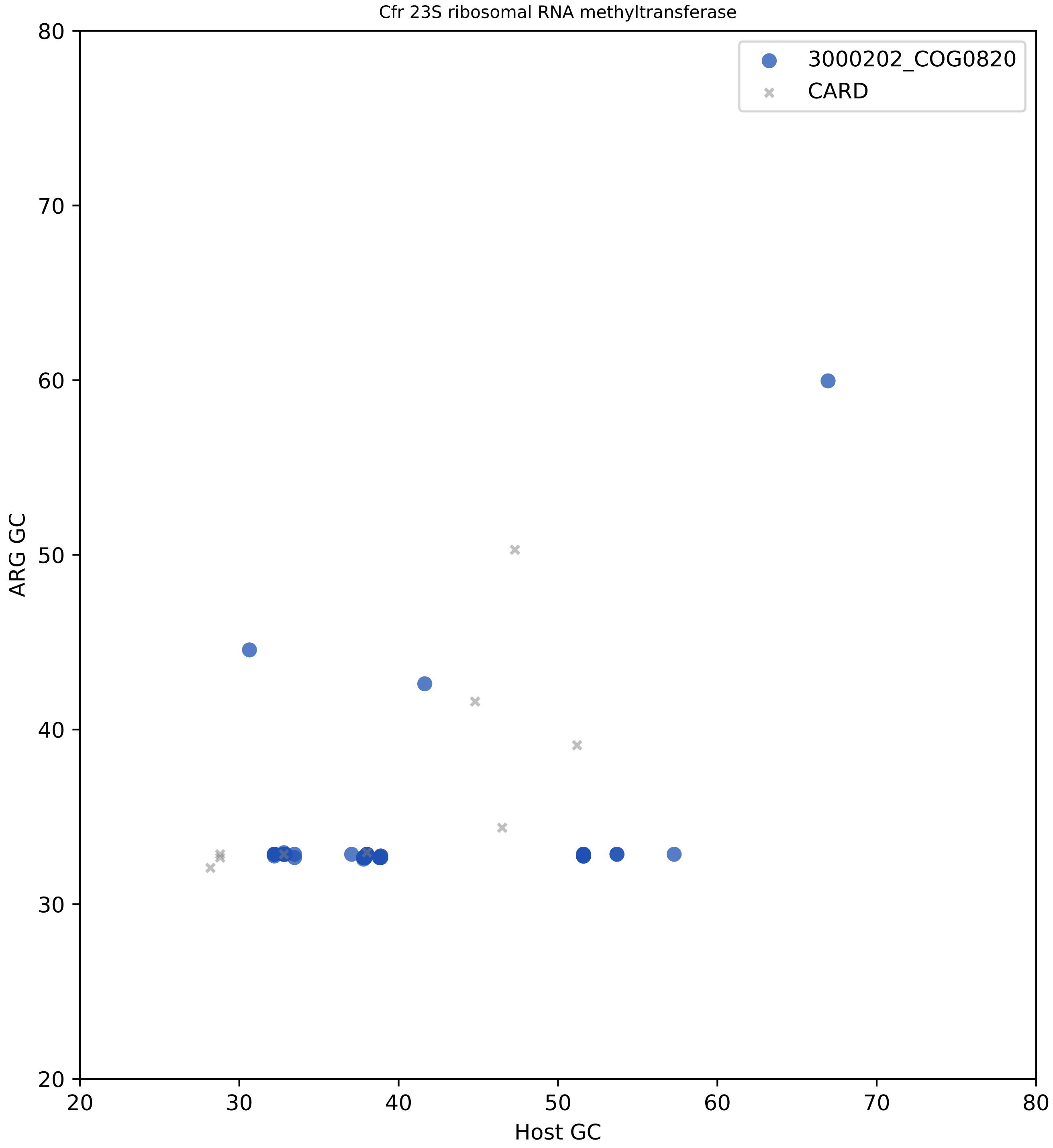

Supplementary Figure S1: (continued).

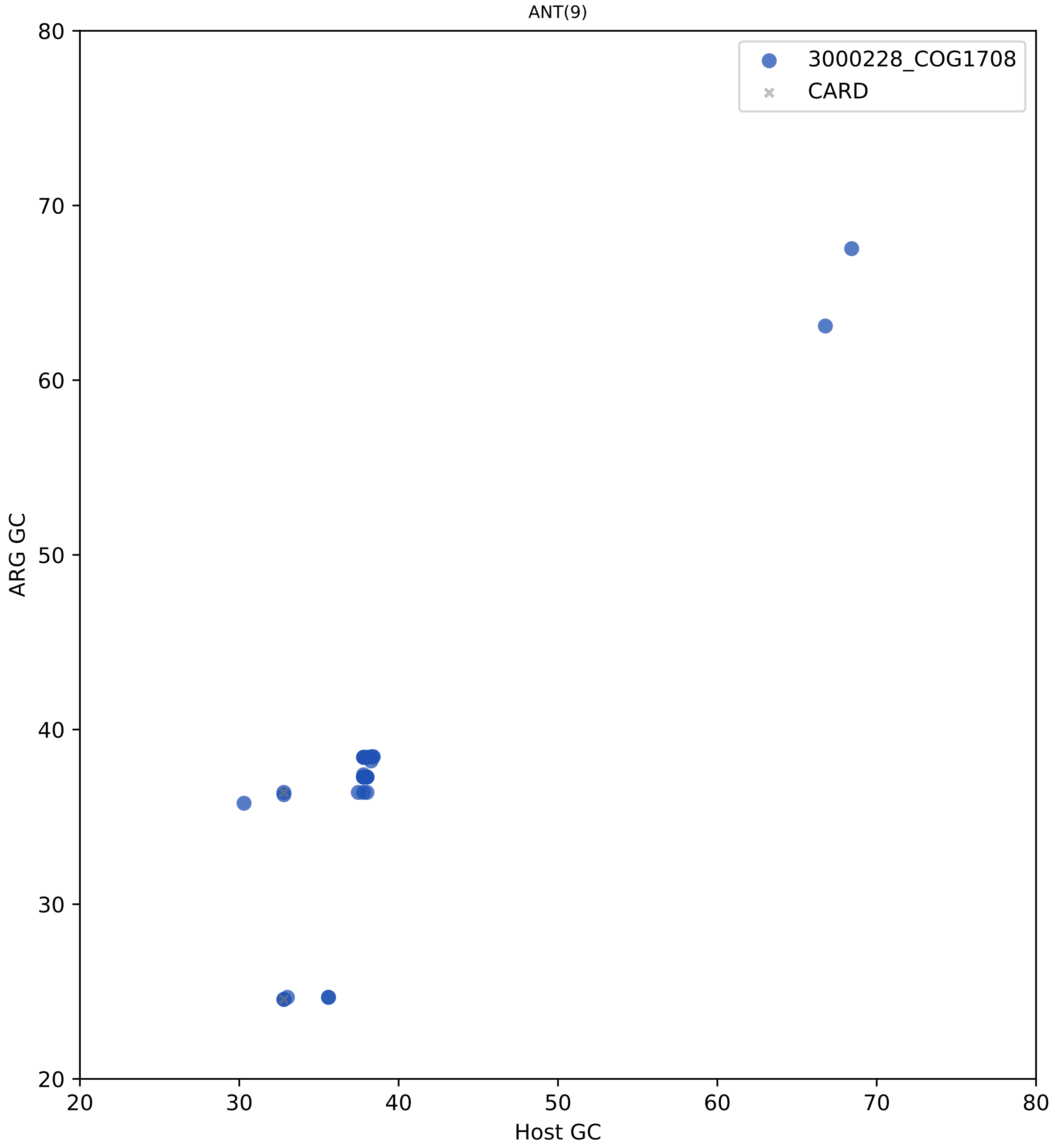

Supplementary Figure S1: (continued).

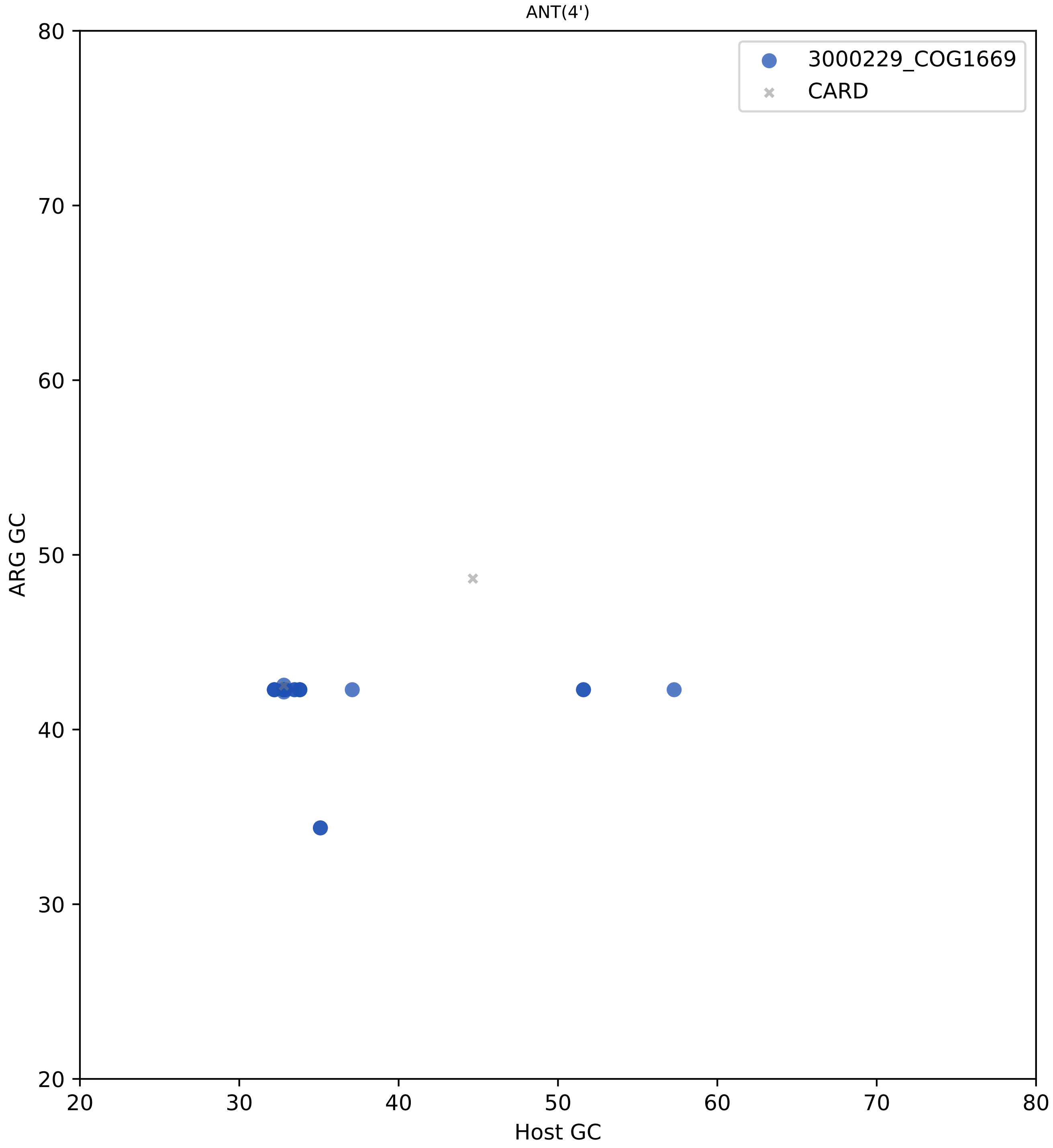

Supplementary Figure S1: (continued).

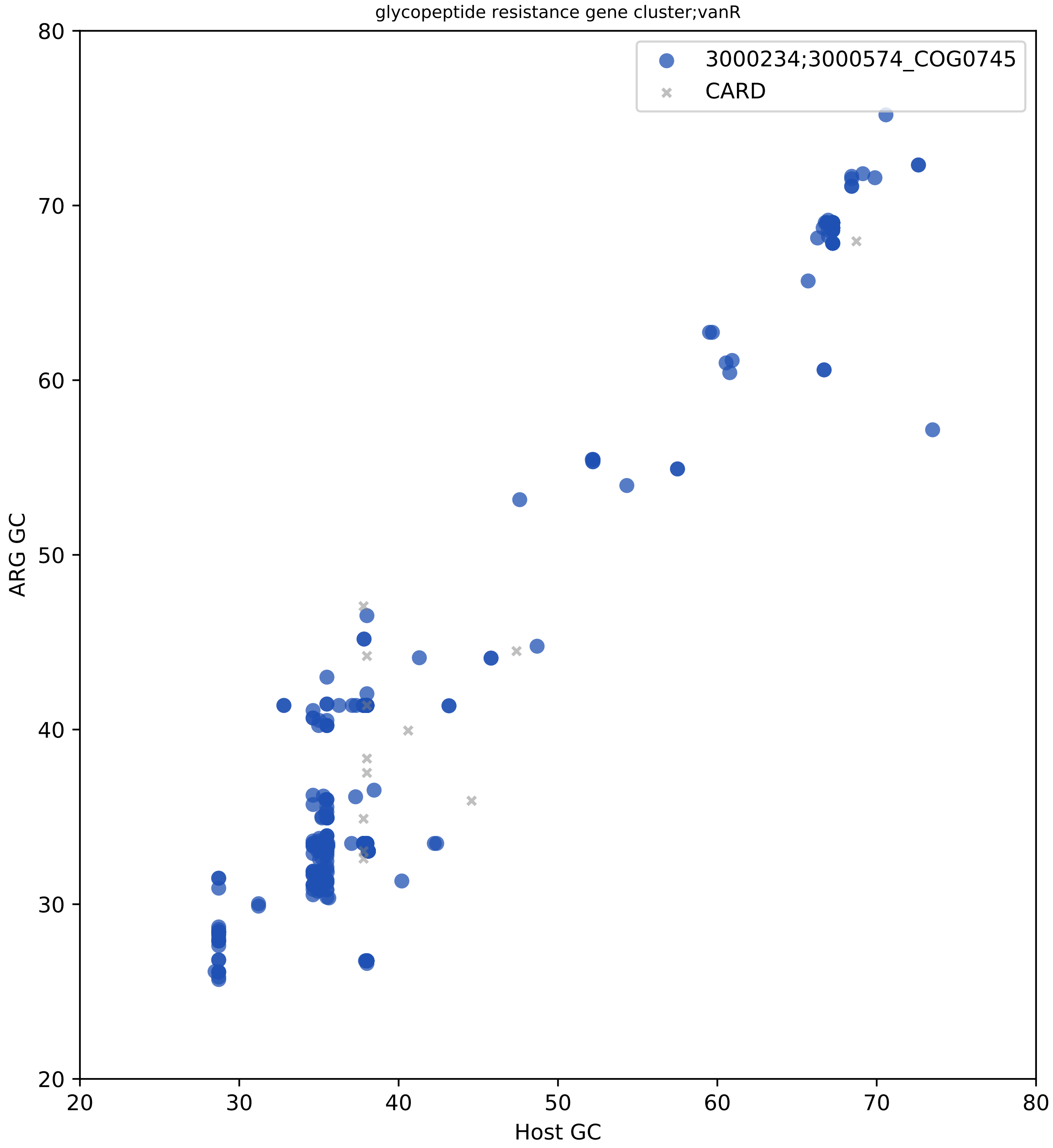

Supplementary Figure S1: (continued).

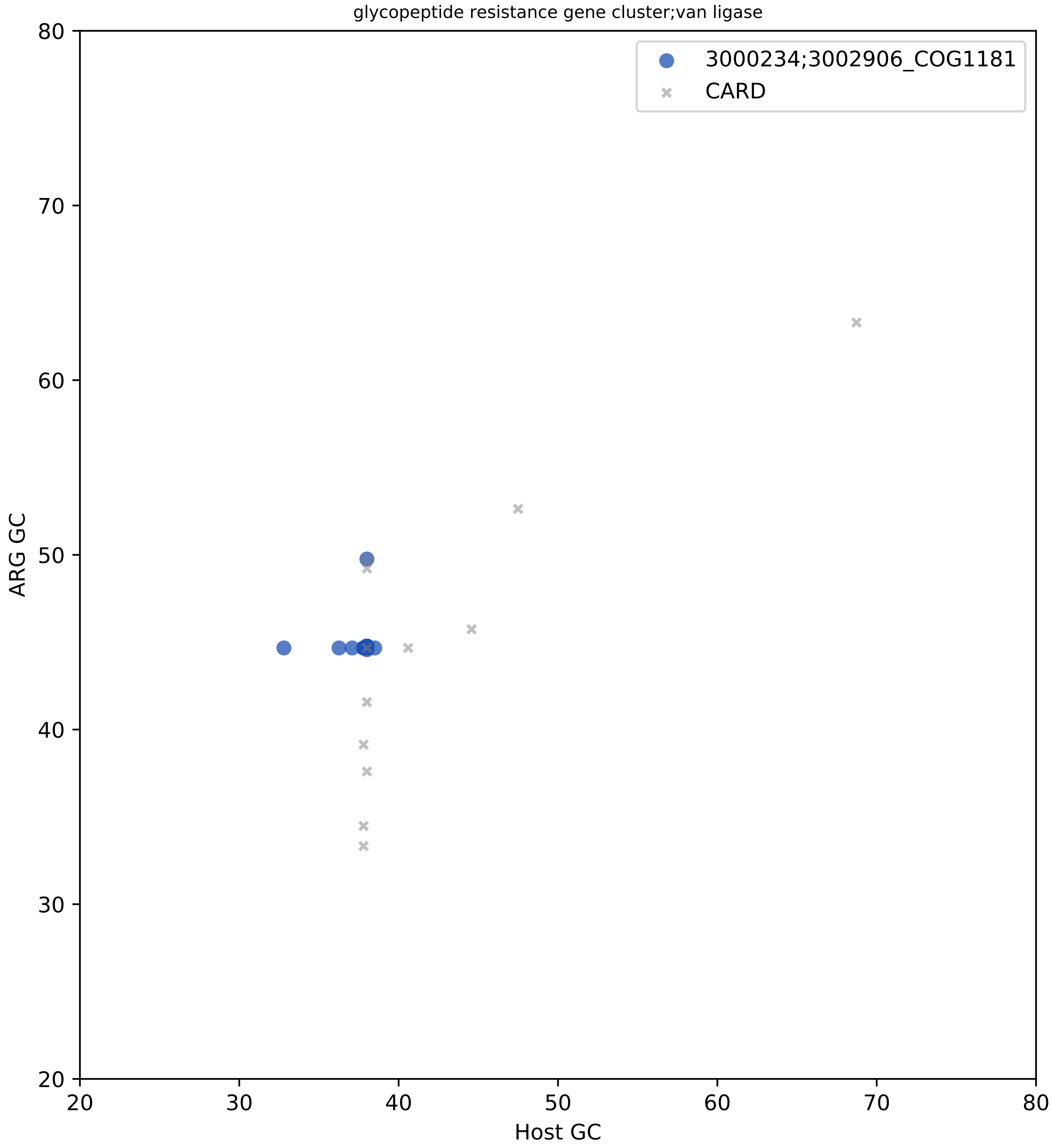

Supplementary Figure S1: (continued).

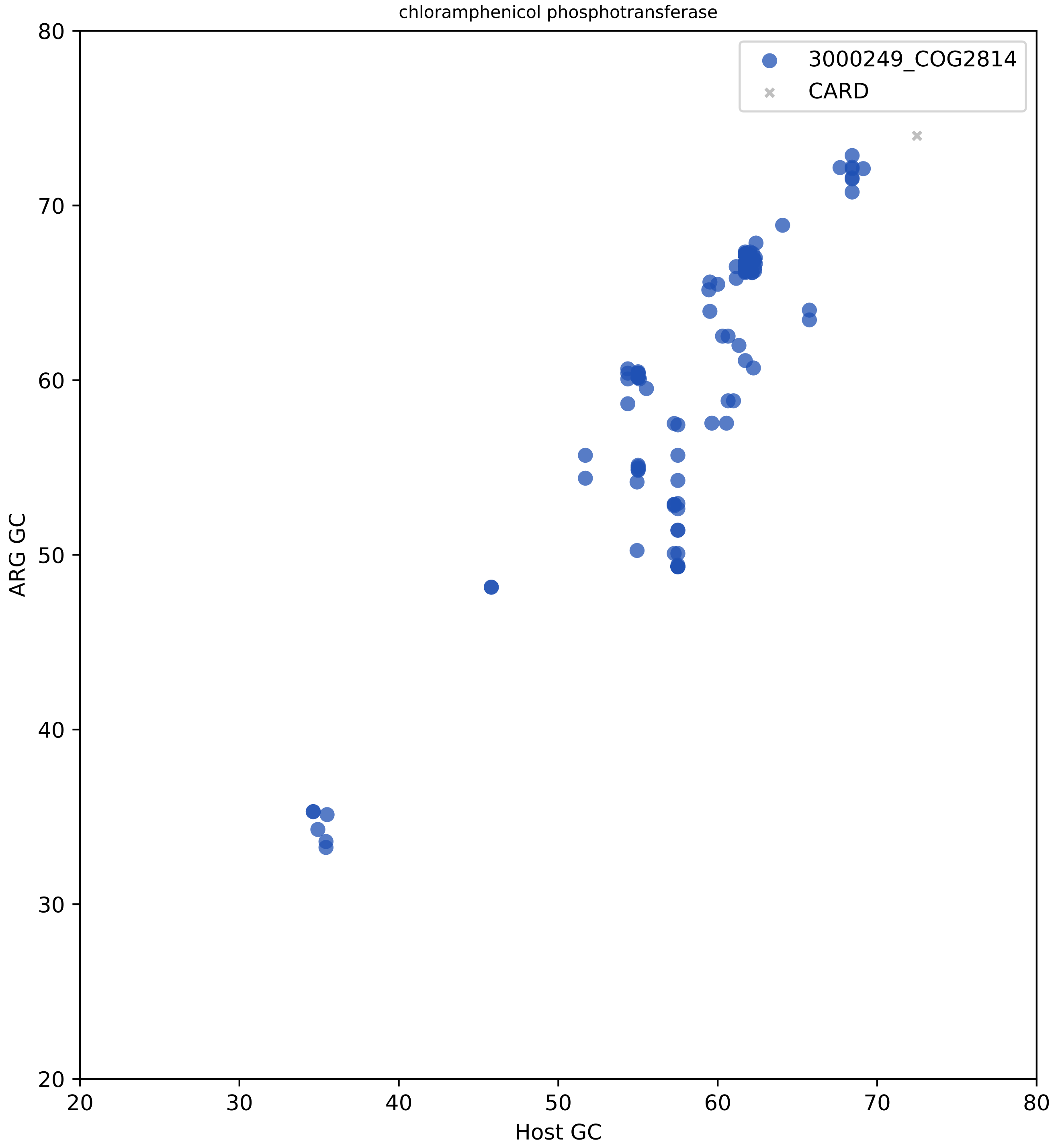

Supplementary Figure S1: (continued).

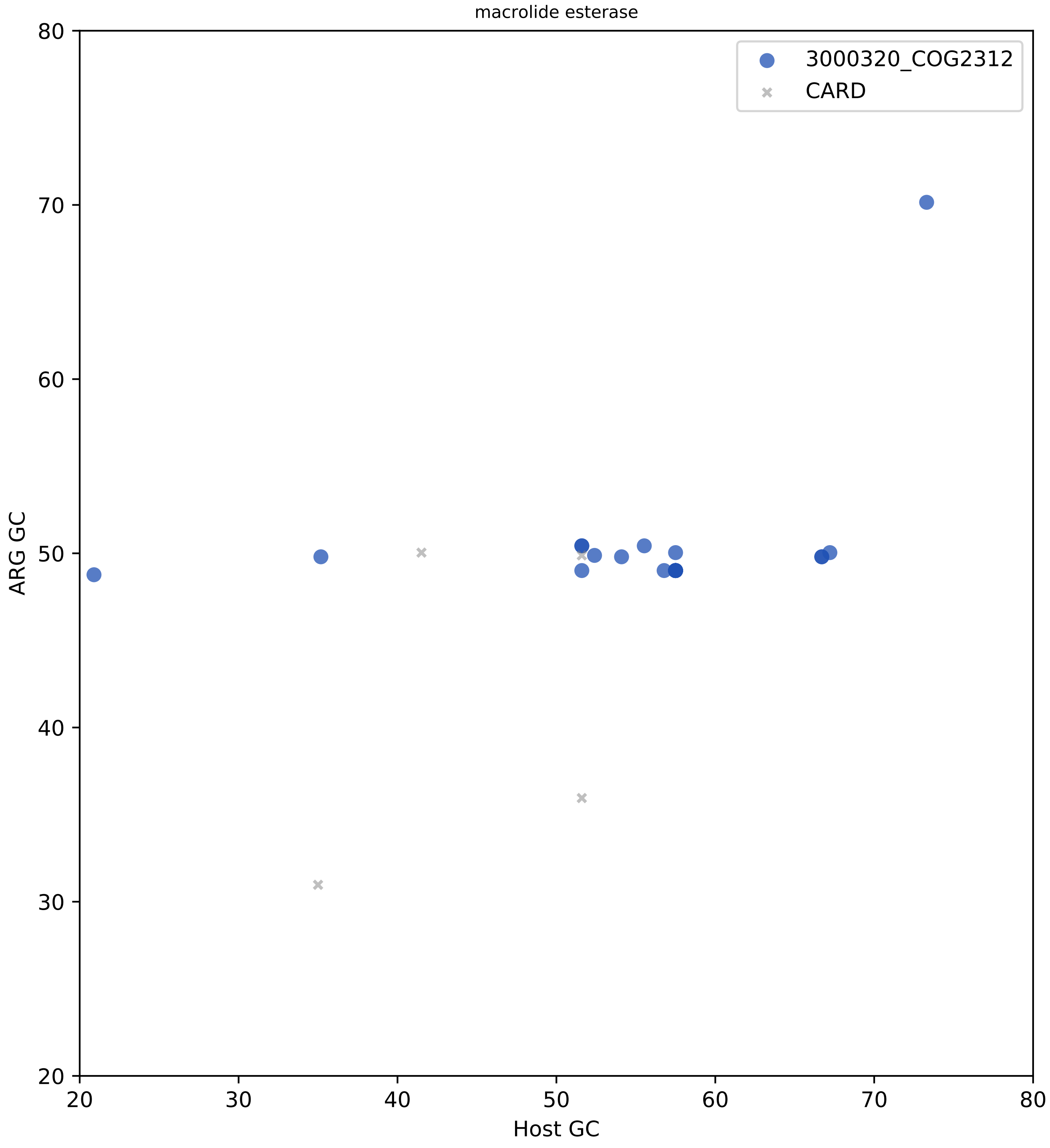

Supplementary Figure S1: (continued).

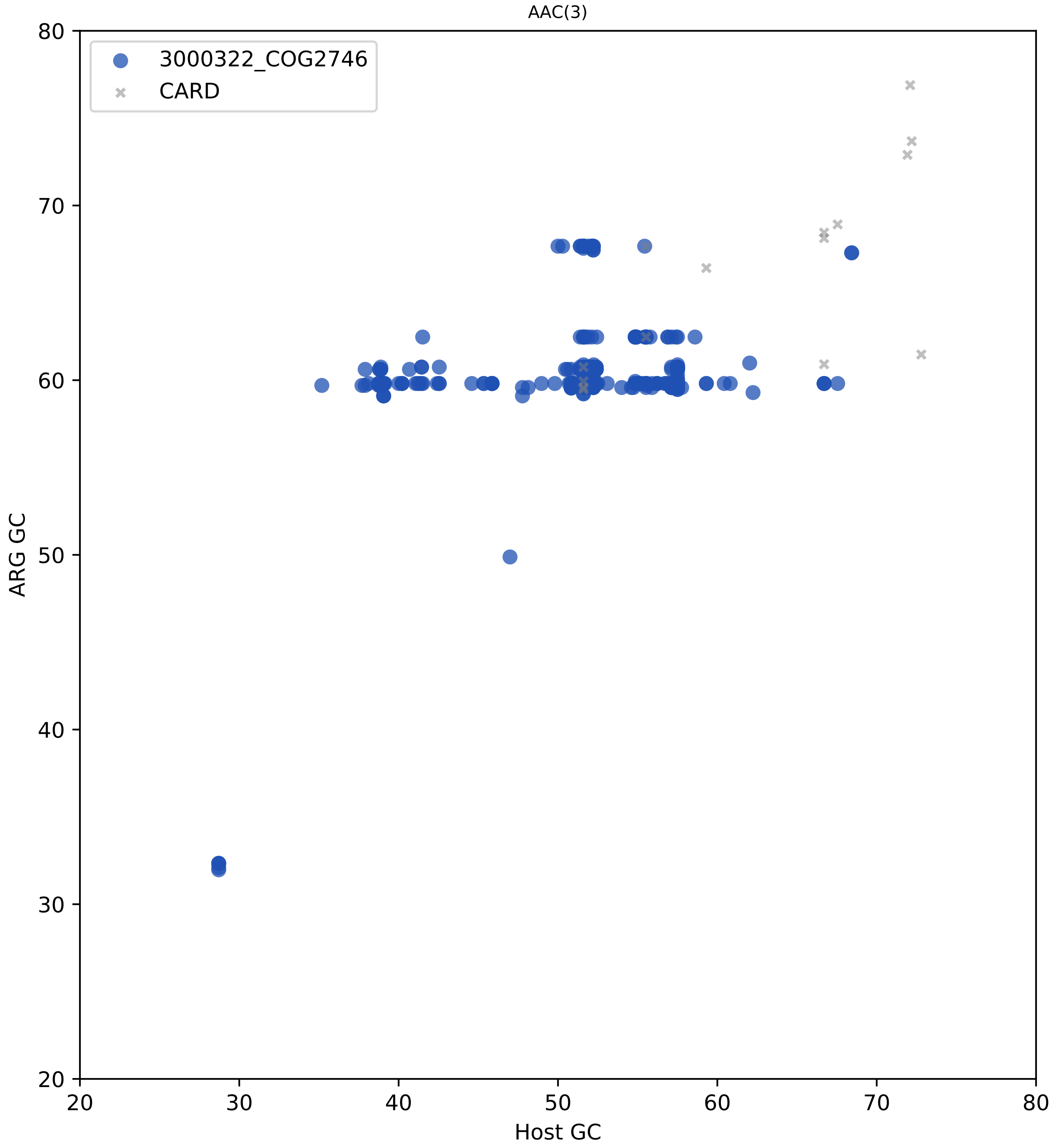

Supplementary Figure S1: (continued).

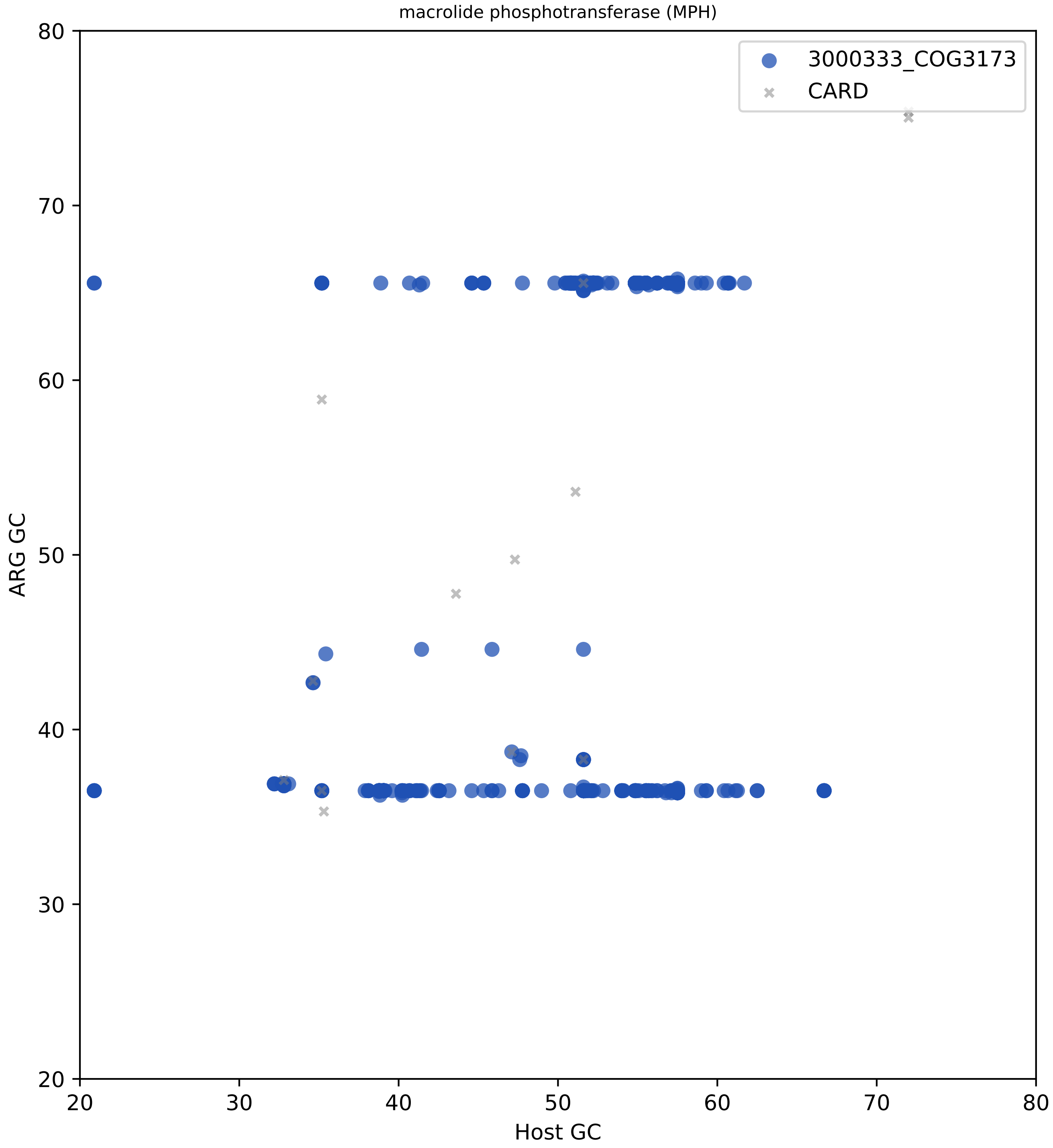

Supplementary Figure S1: (continued).

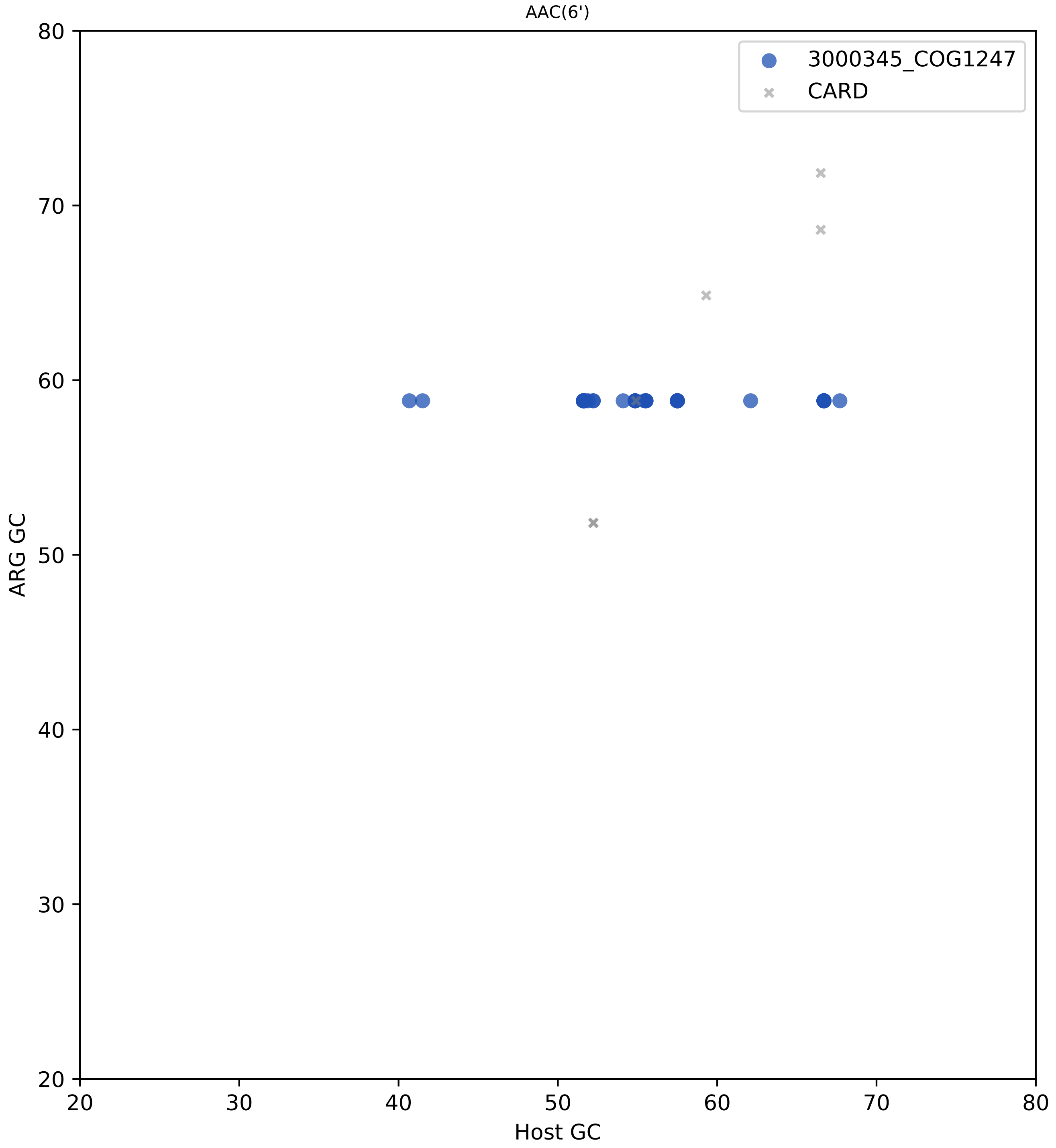

Supplementary Figure S1: (continued).

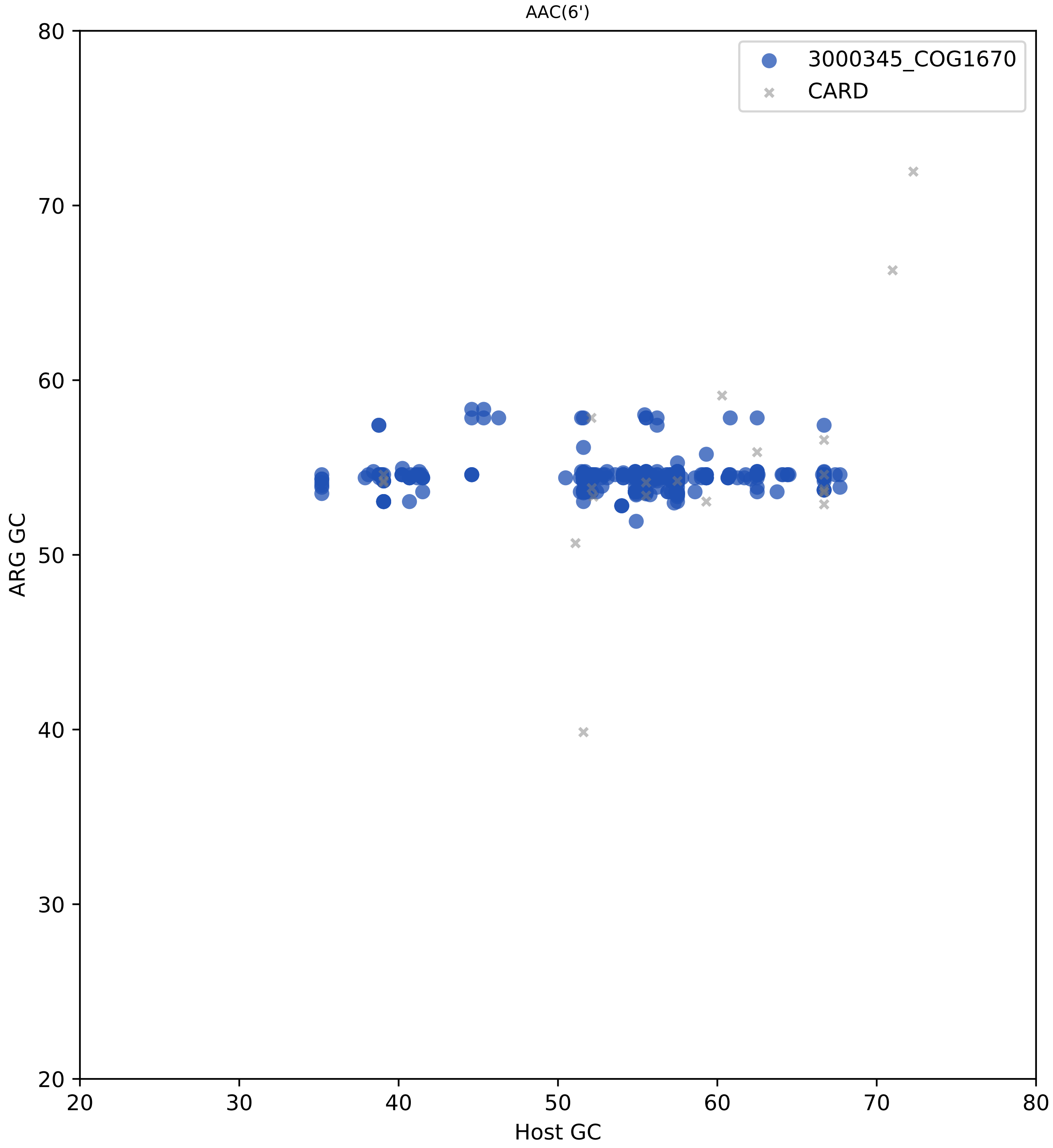

Supplementary Figure S1: (continued).

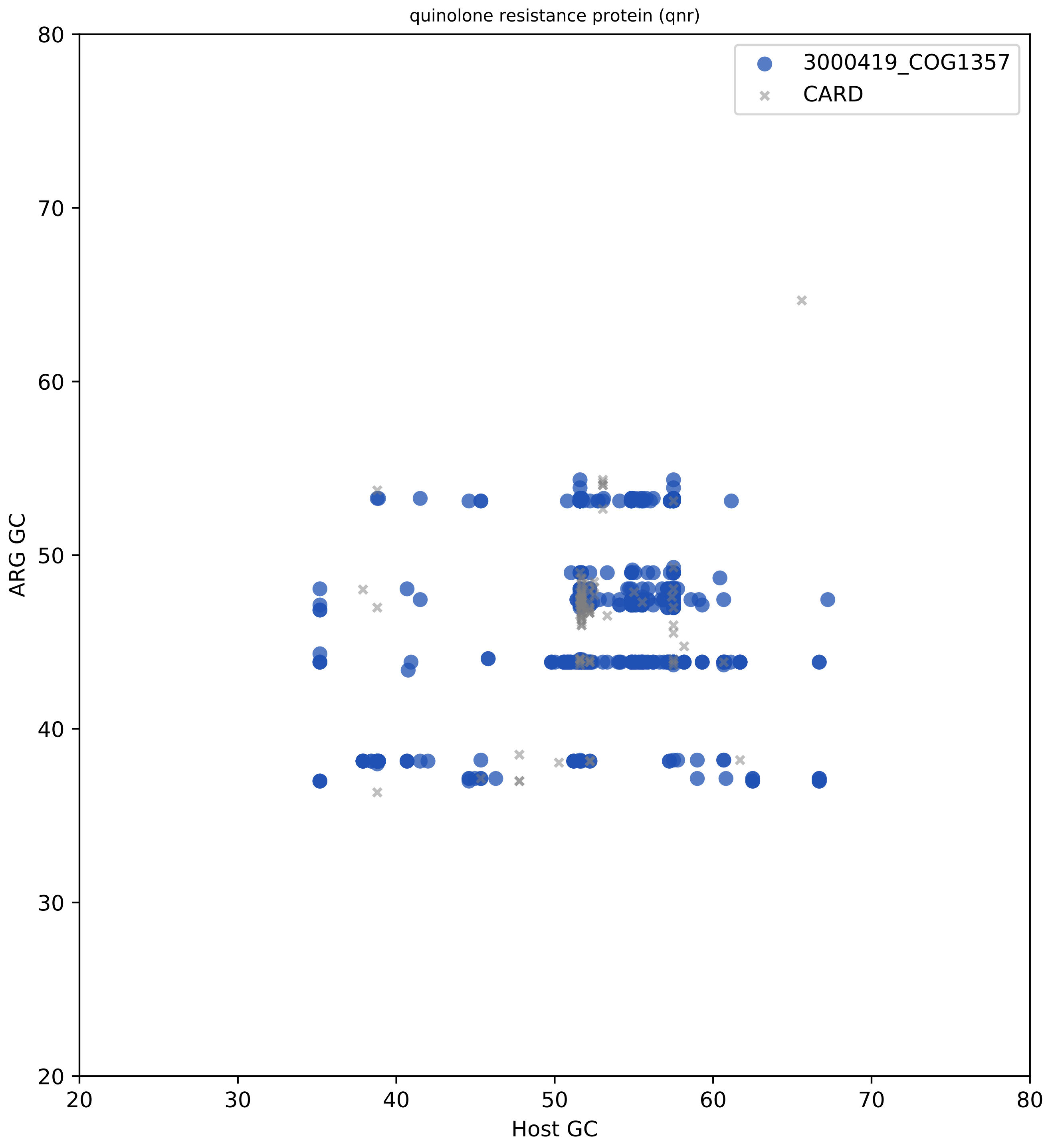

Supplementary Figure S1: (continued).

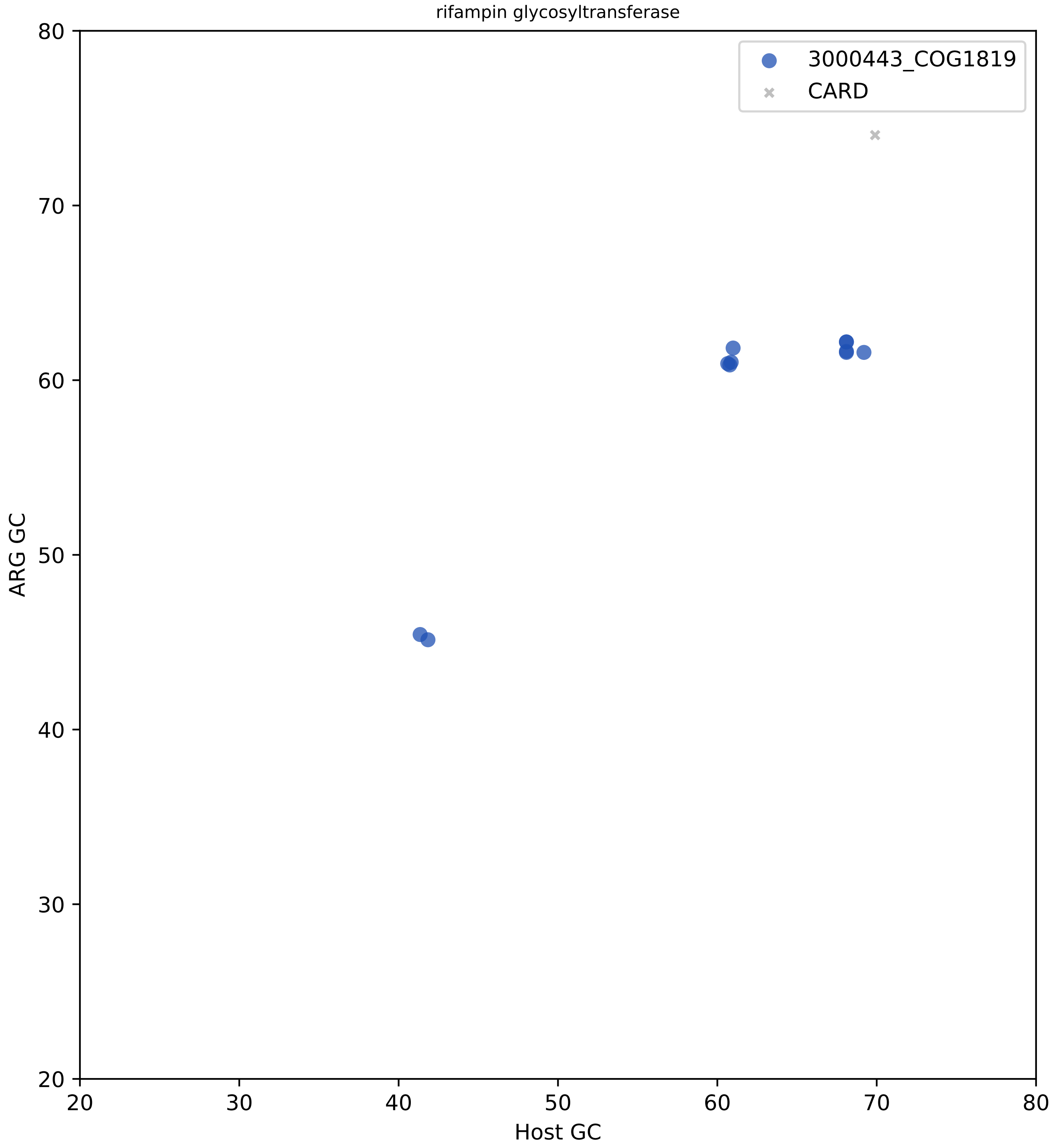

Supplementary Figure S1: (continued).

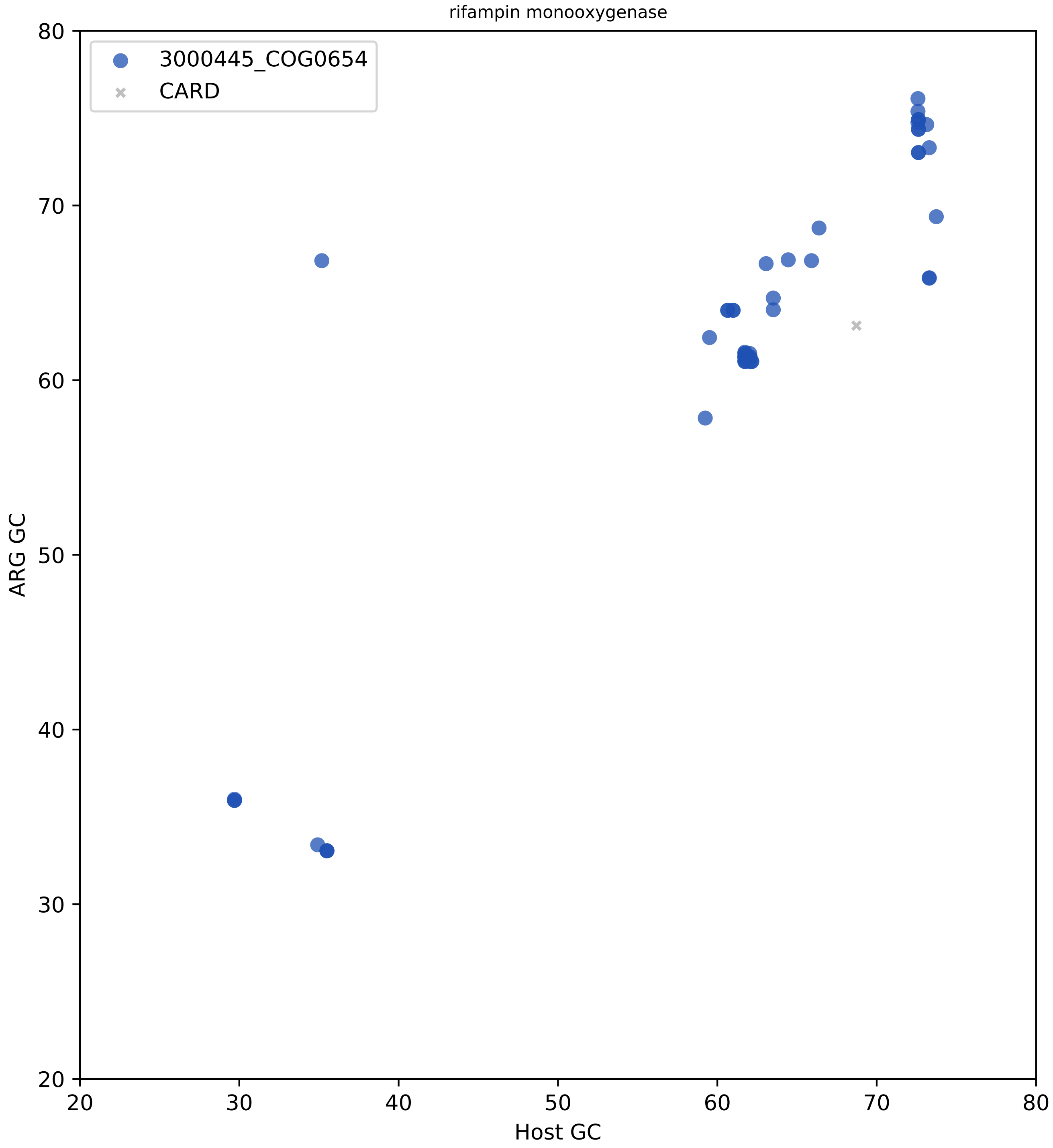

Supplementary Figure S1: (continued).

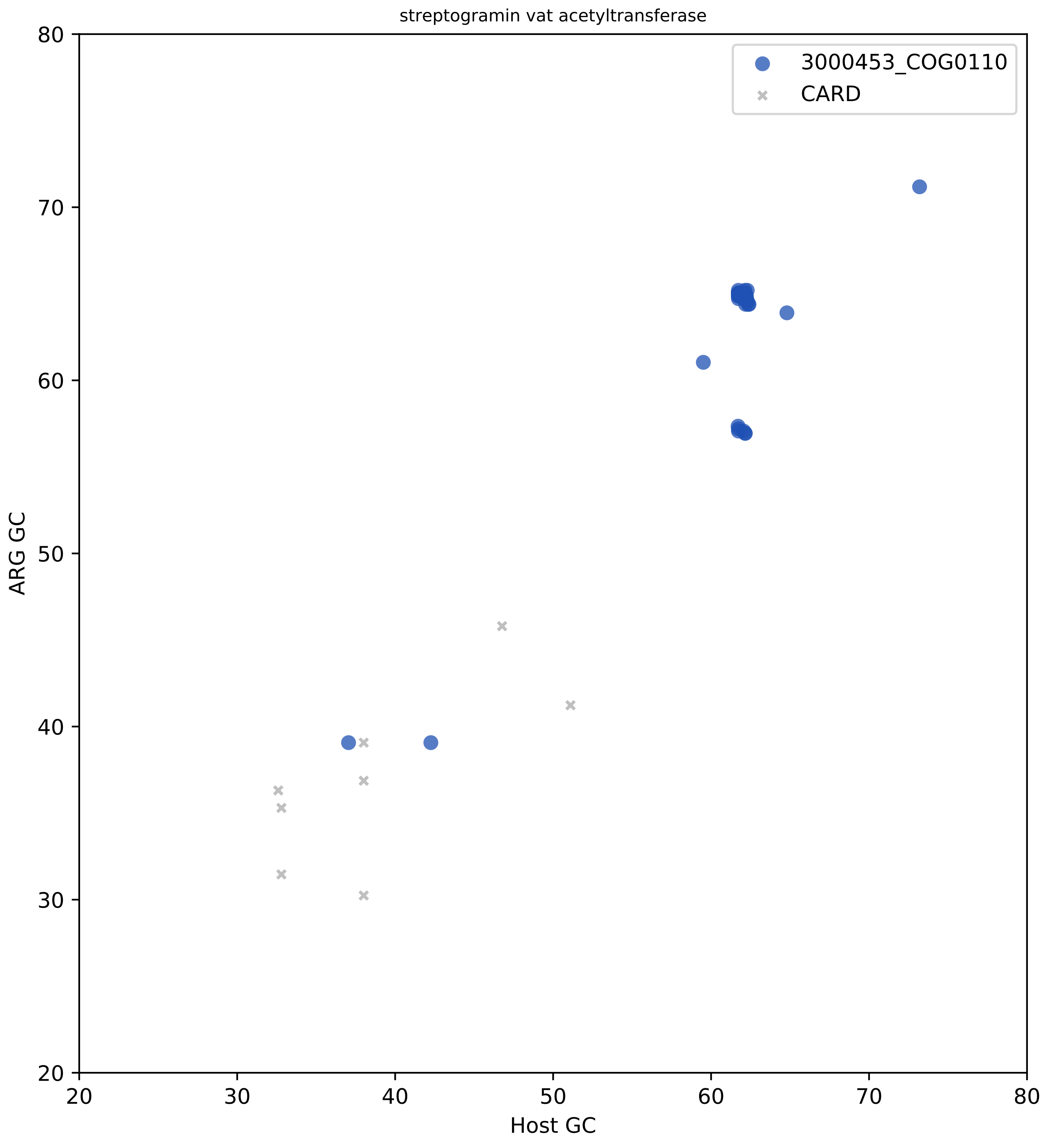

Supplementary Figure S1: (continued).

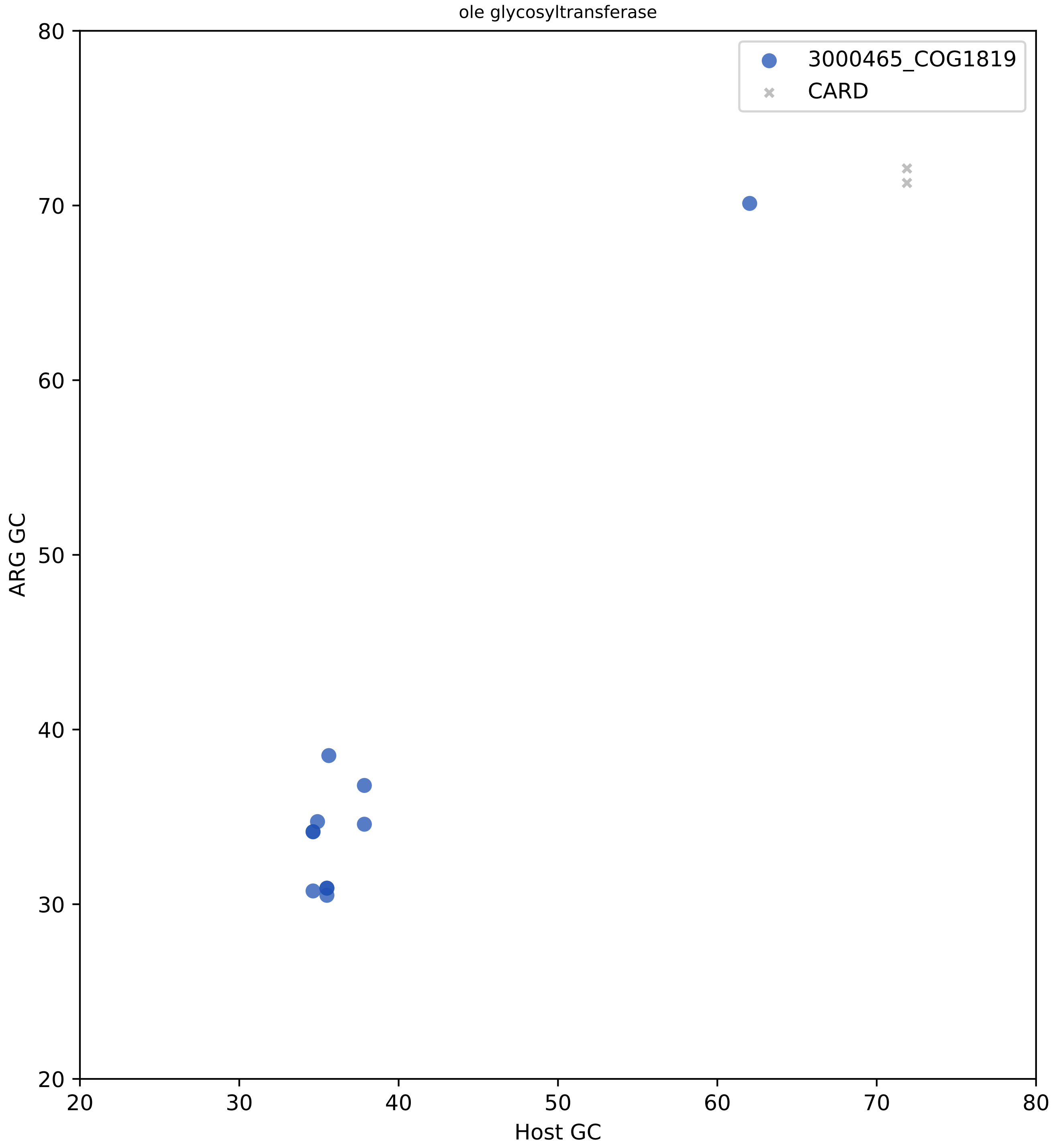

Supplementary Figure S1: (continued).

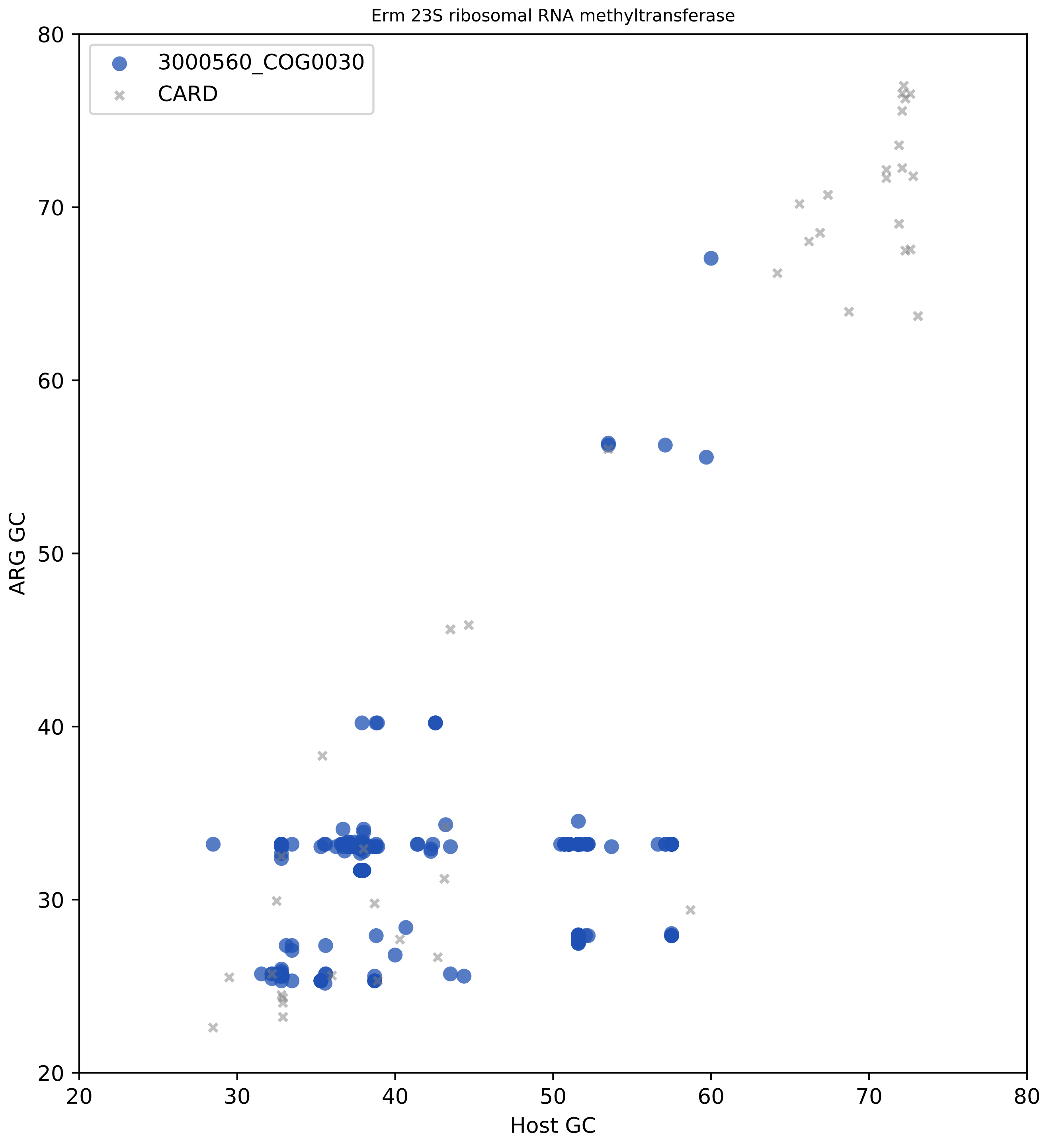

Supplementary Figure S1: (continued).

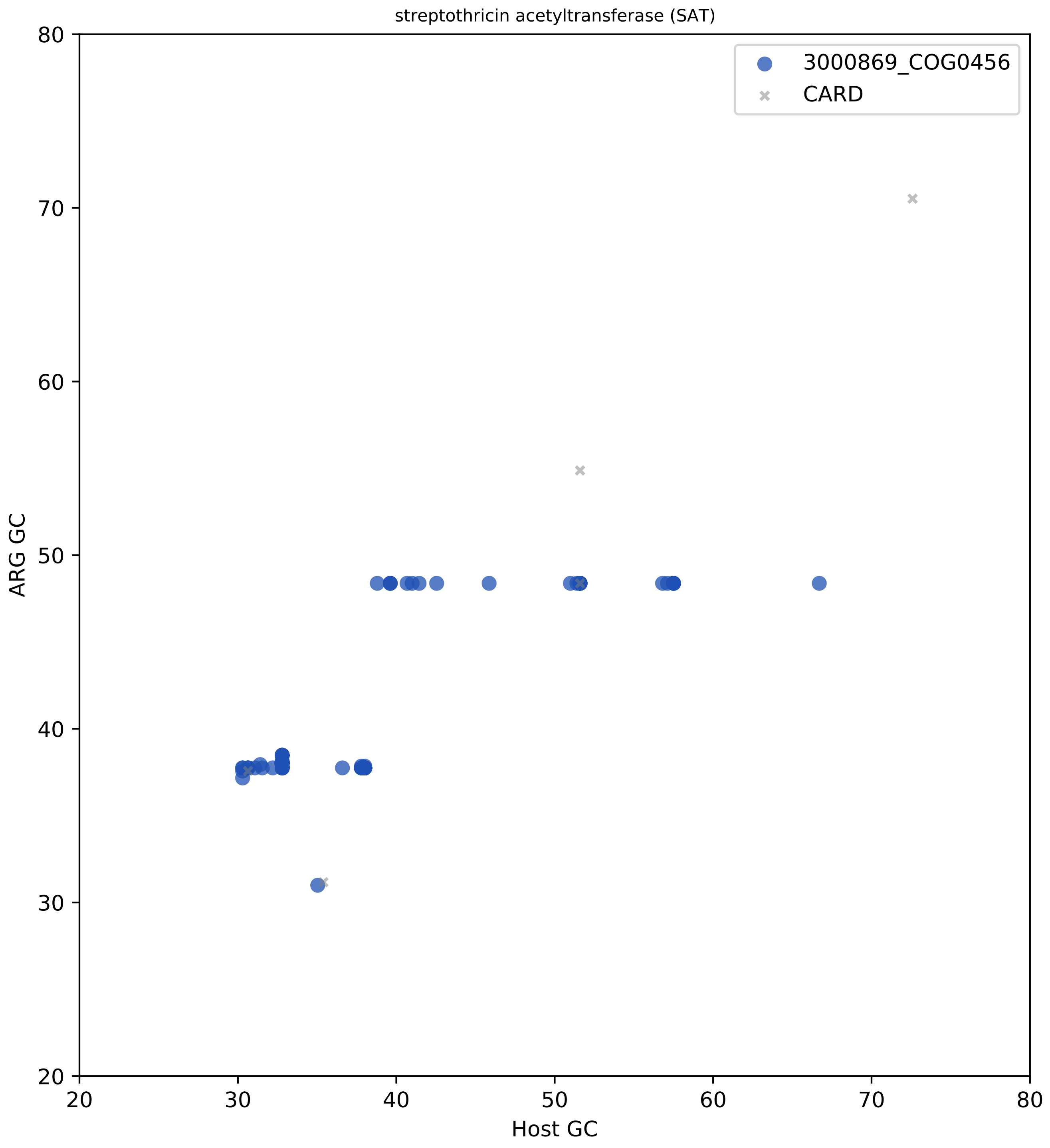

Supplementary Figure S1: (continued).

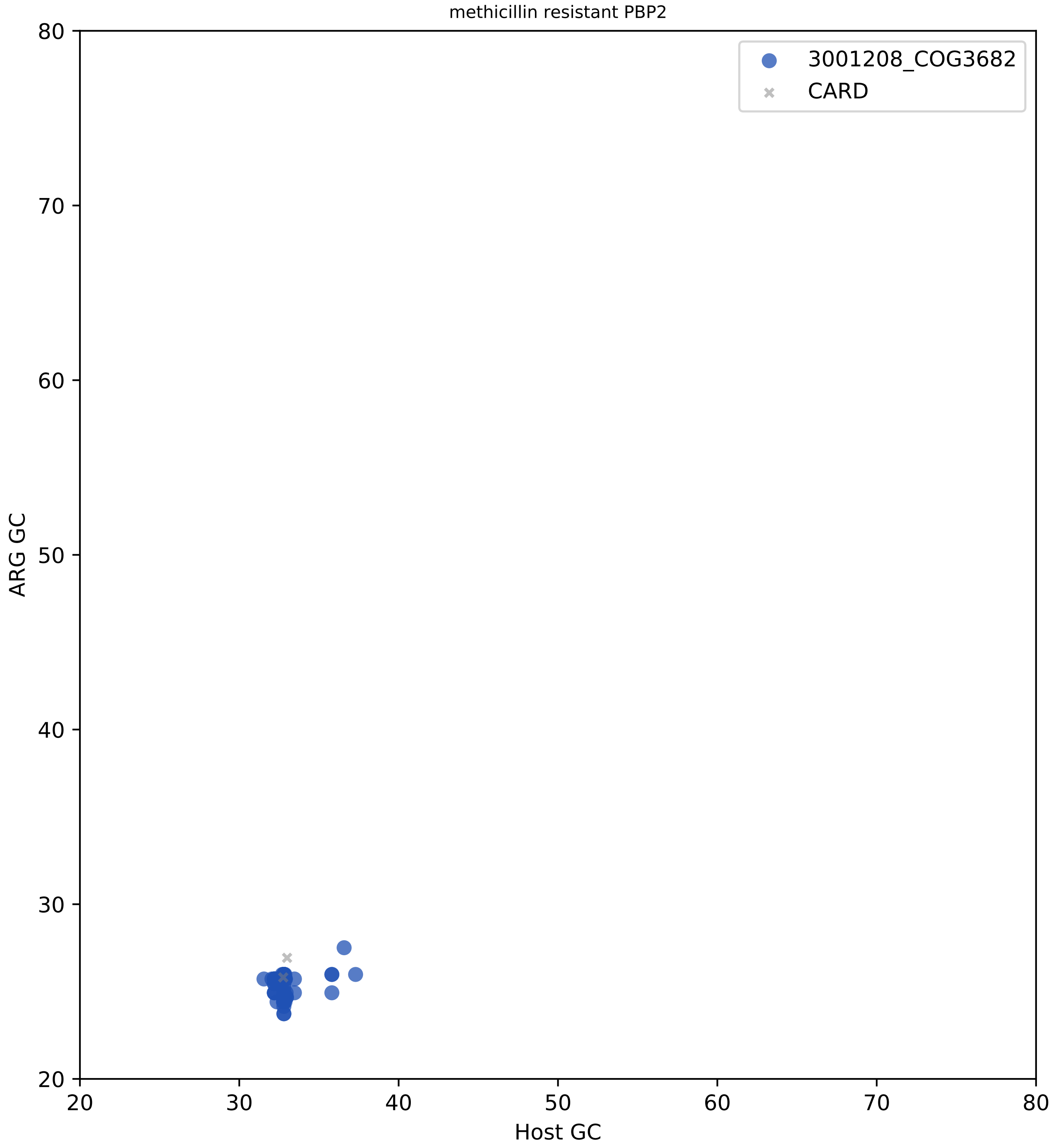

Supplementary Figure S1: (continued).

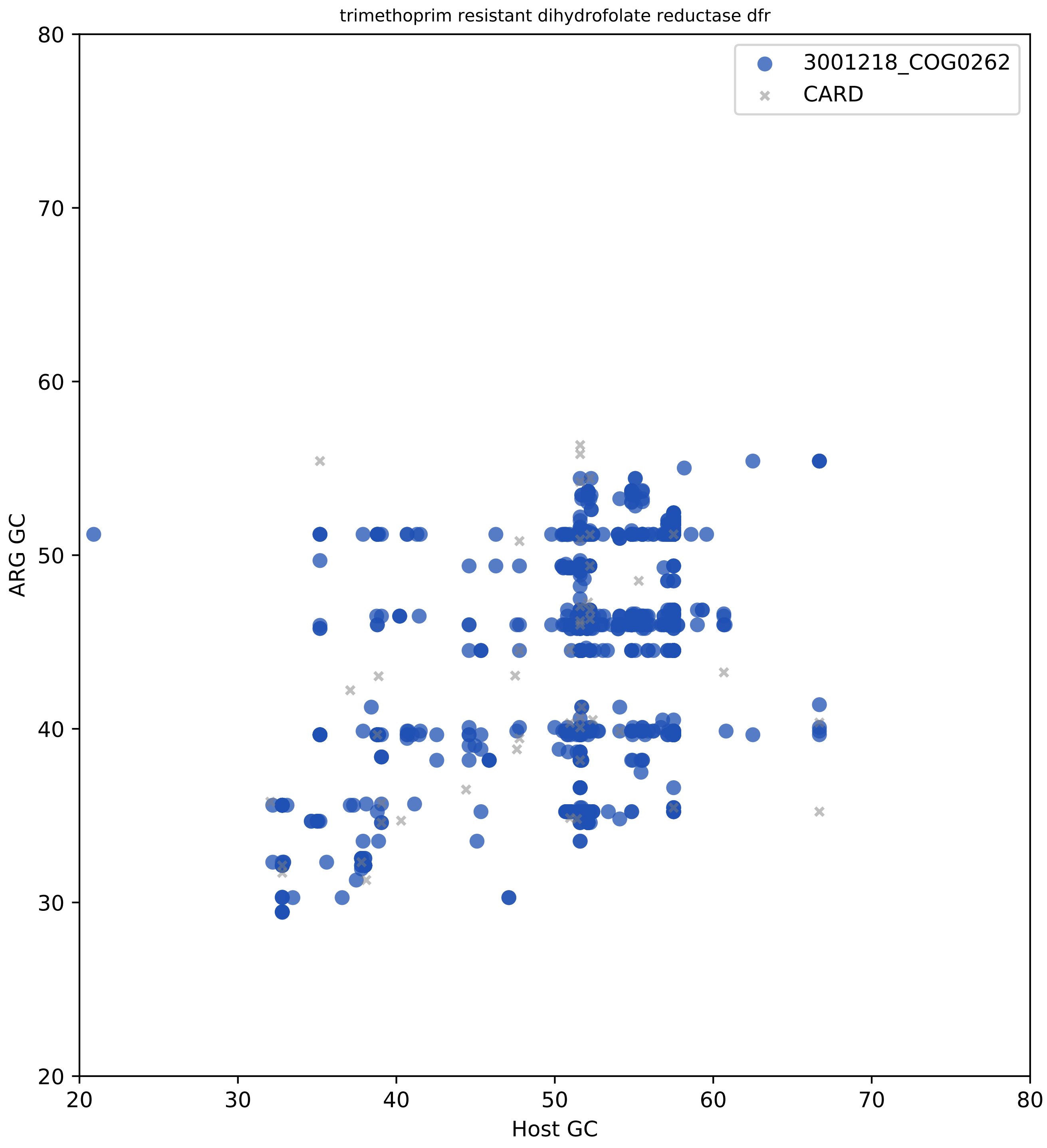

Supplementary Figure S1: (continued).

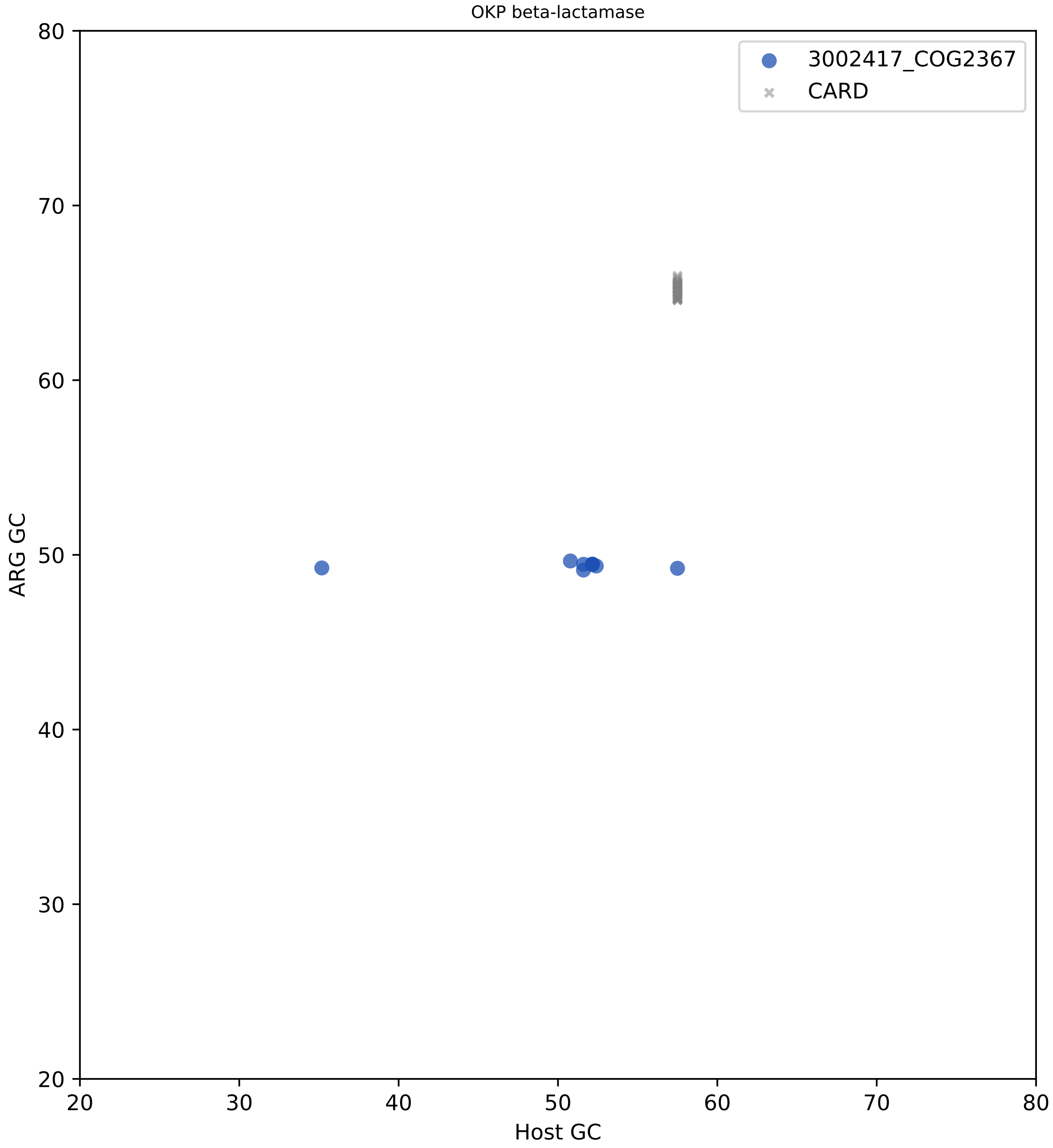

Supplementary Figure S1: (continued).

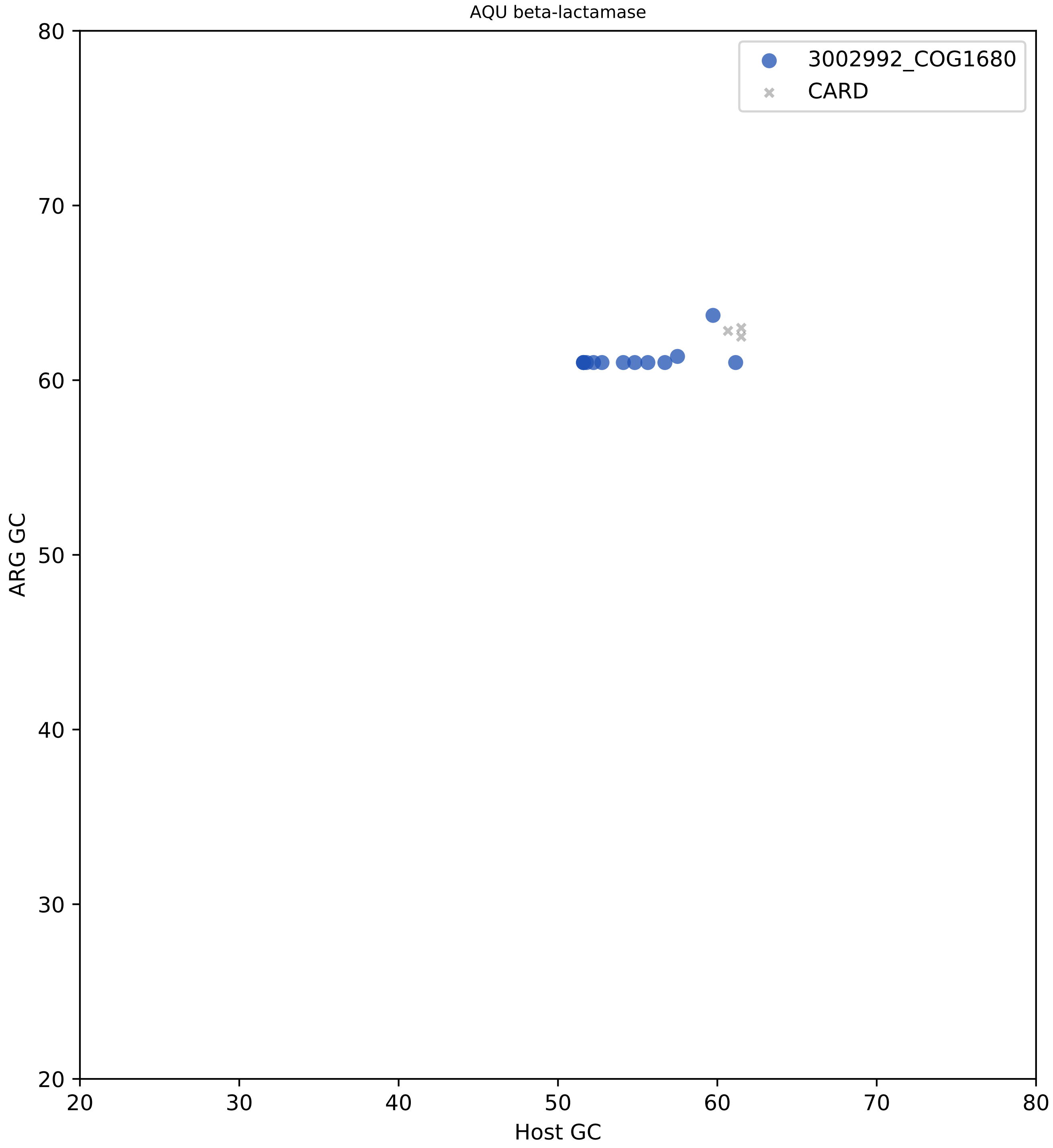

Supplementary Figure S1: (continued).

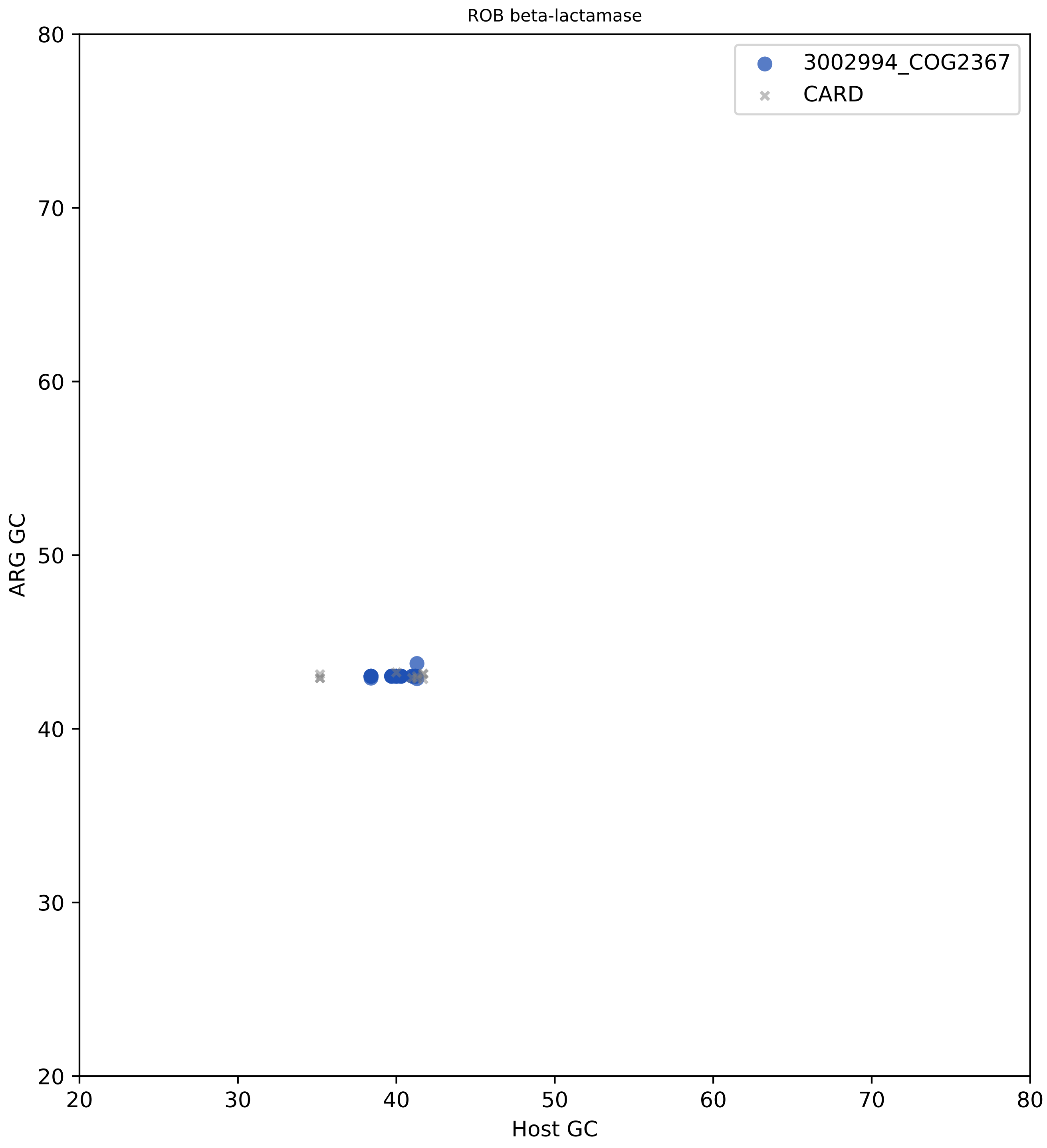

Supplementary Figure S1: (continued).

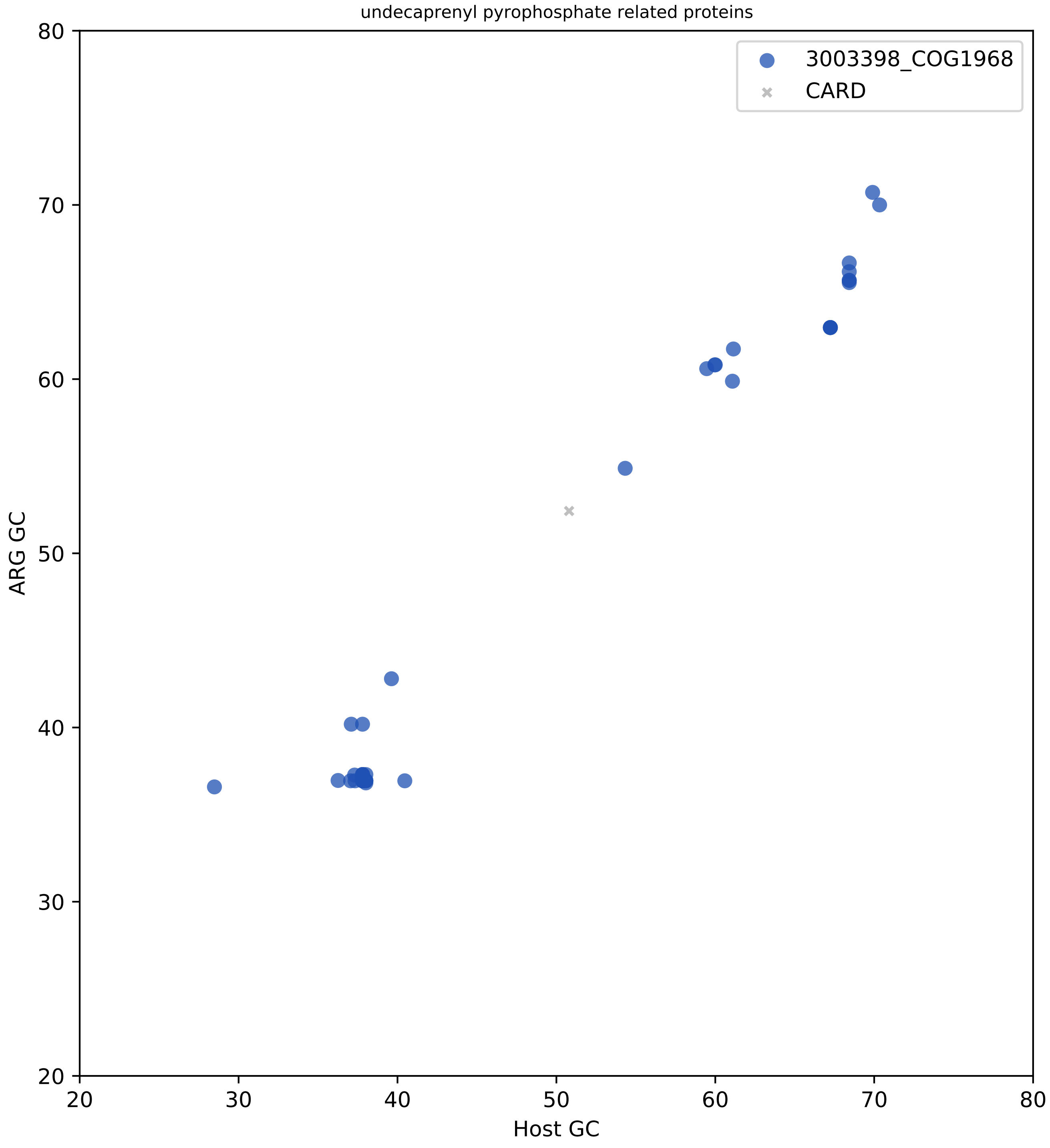

Supplementary Figure S1: (continued).

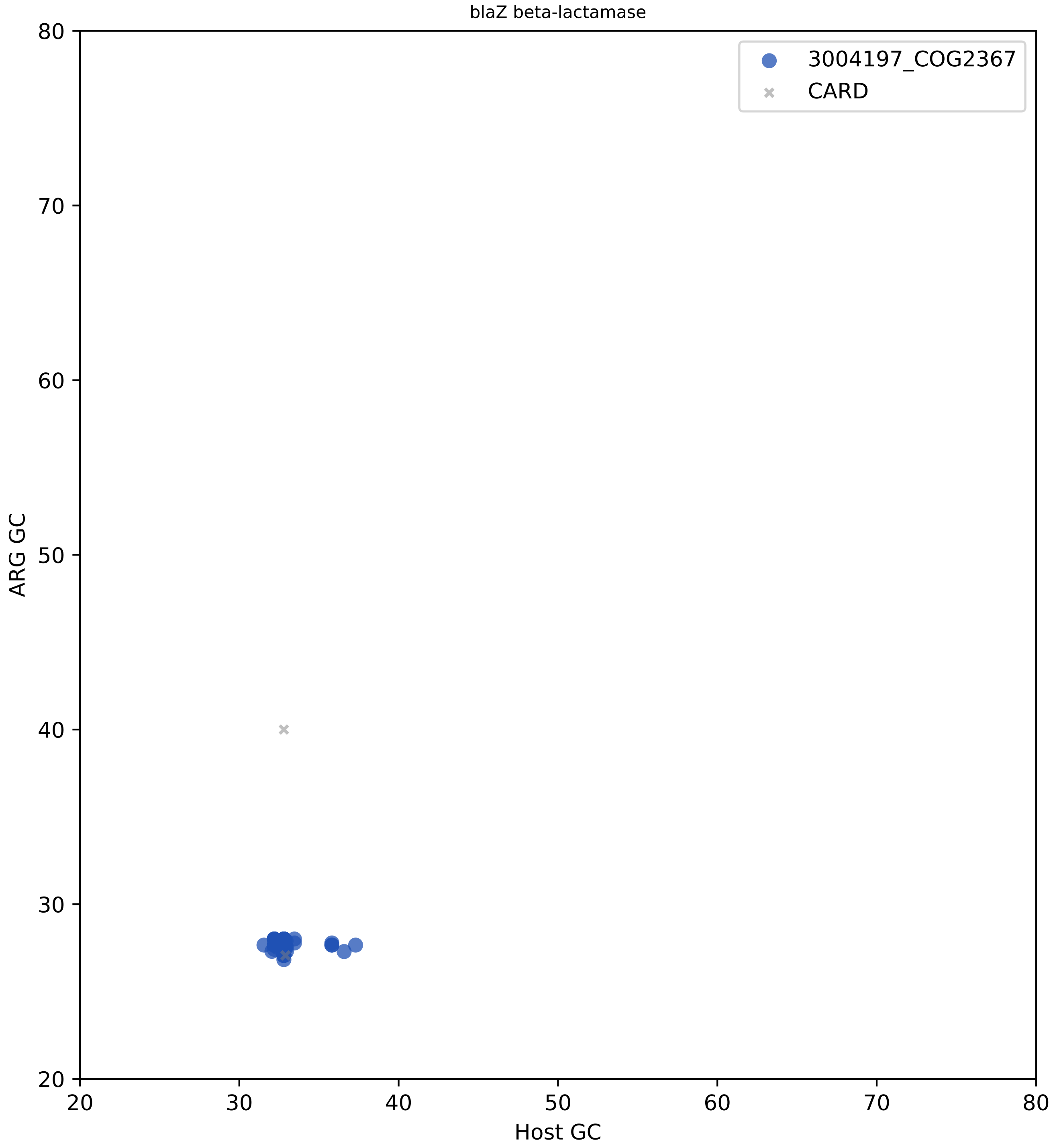

Supplementary Figure S1: (continued).

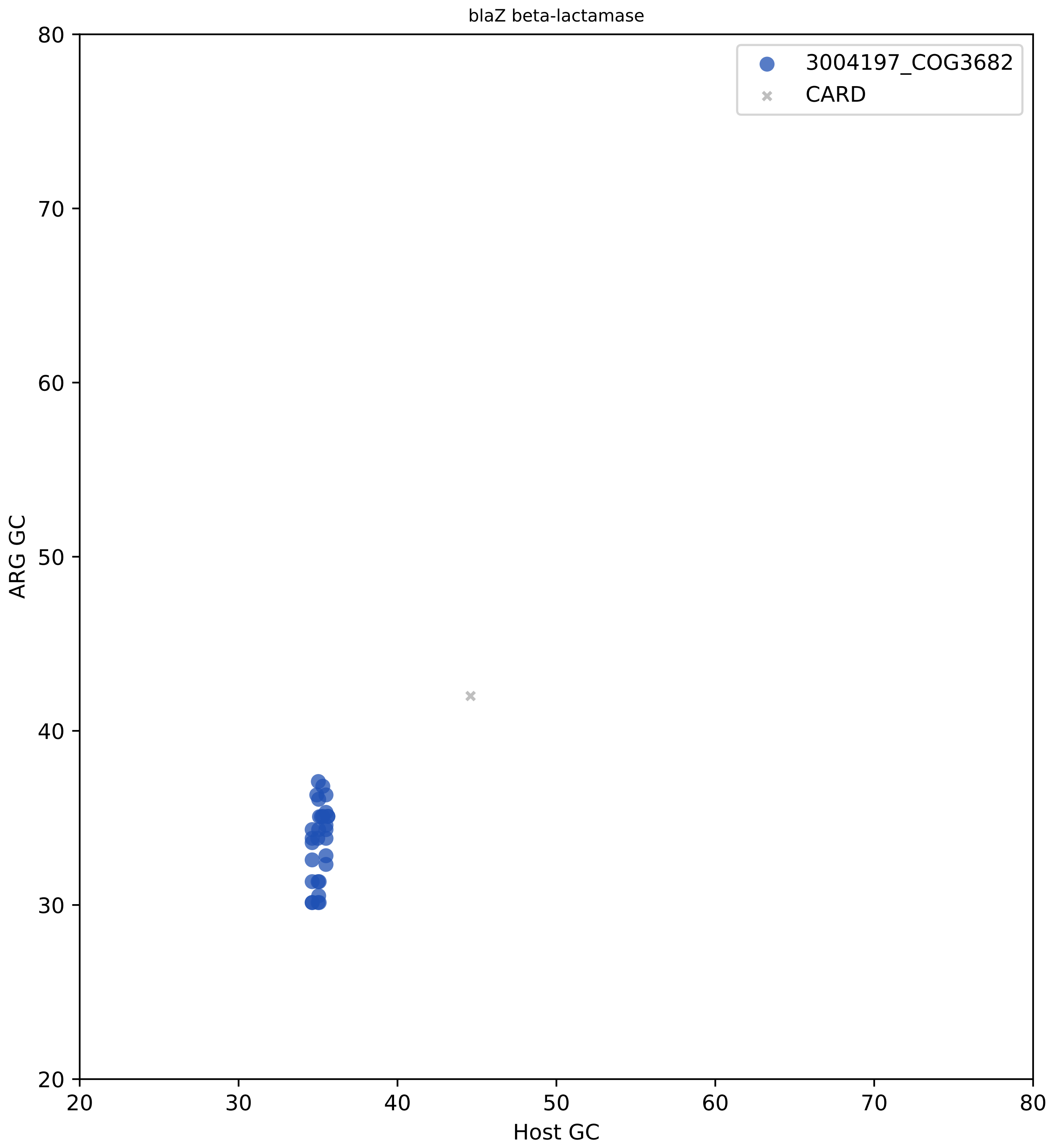

Supplementary Figure S1: (continued).

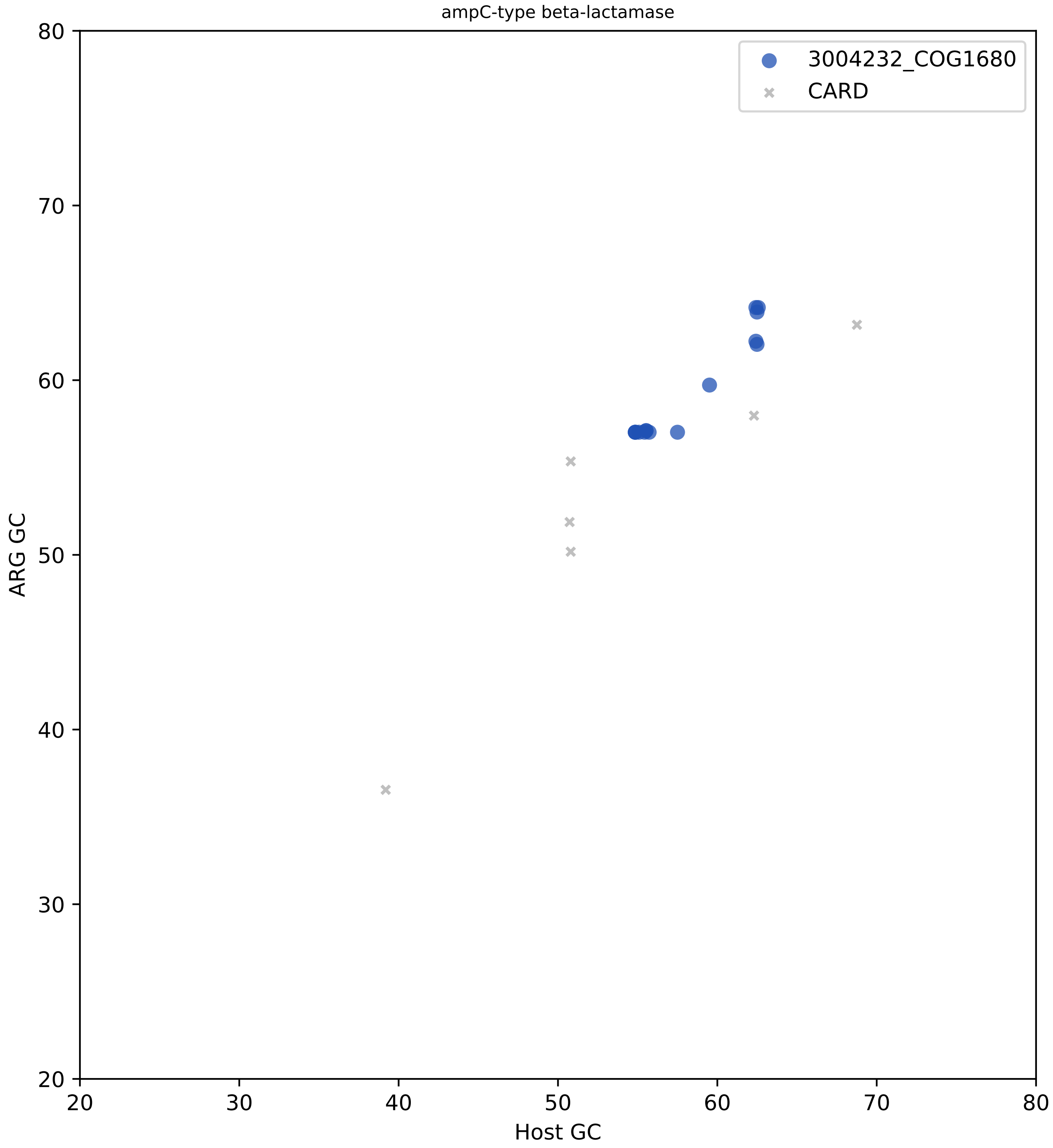

Supplementary Figure S1: (continued).

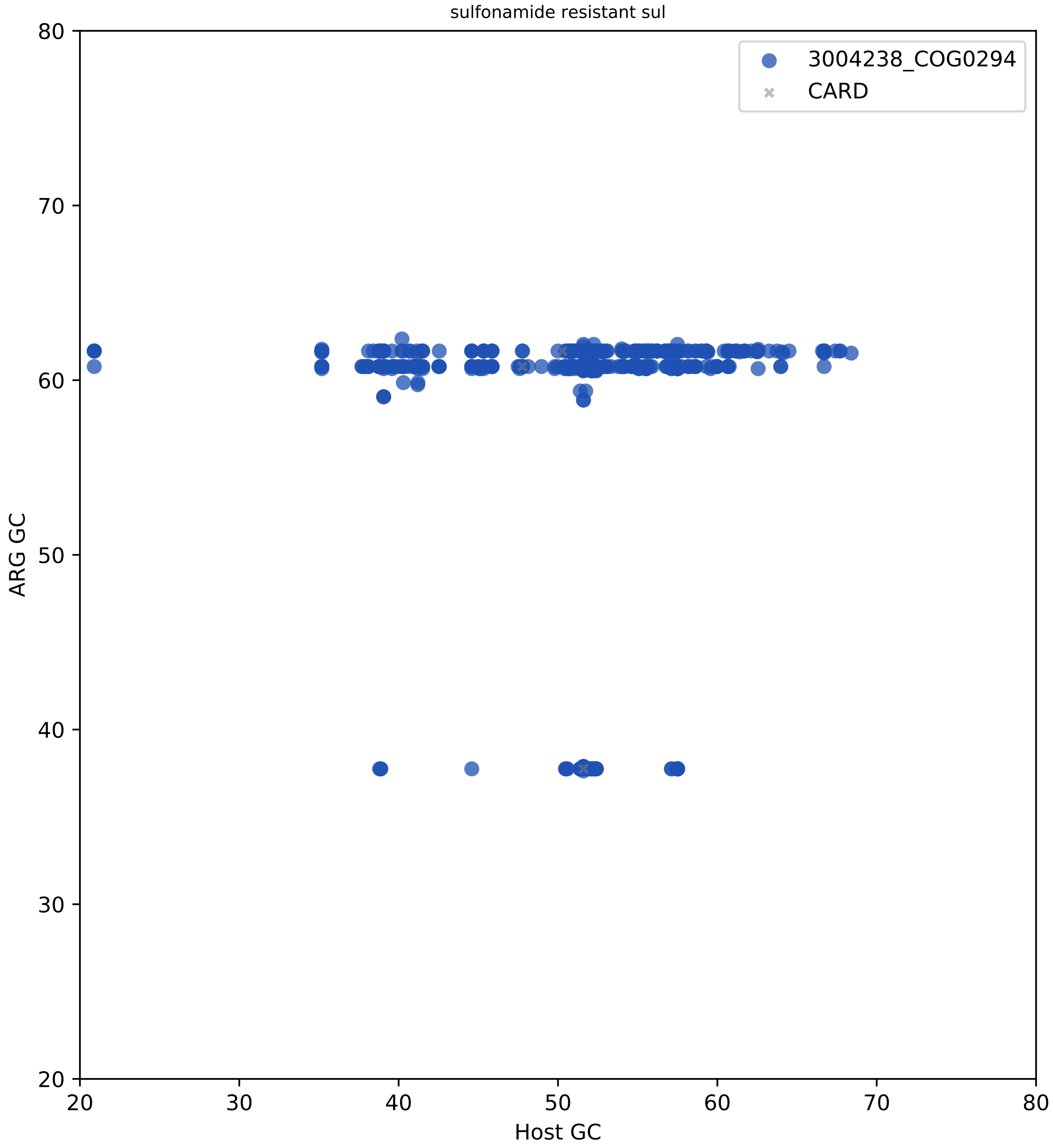

Supplementary Figure S1: (continued).

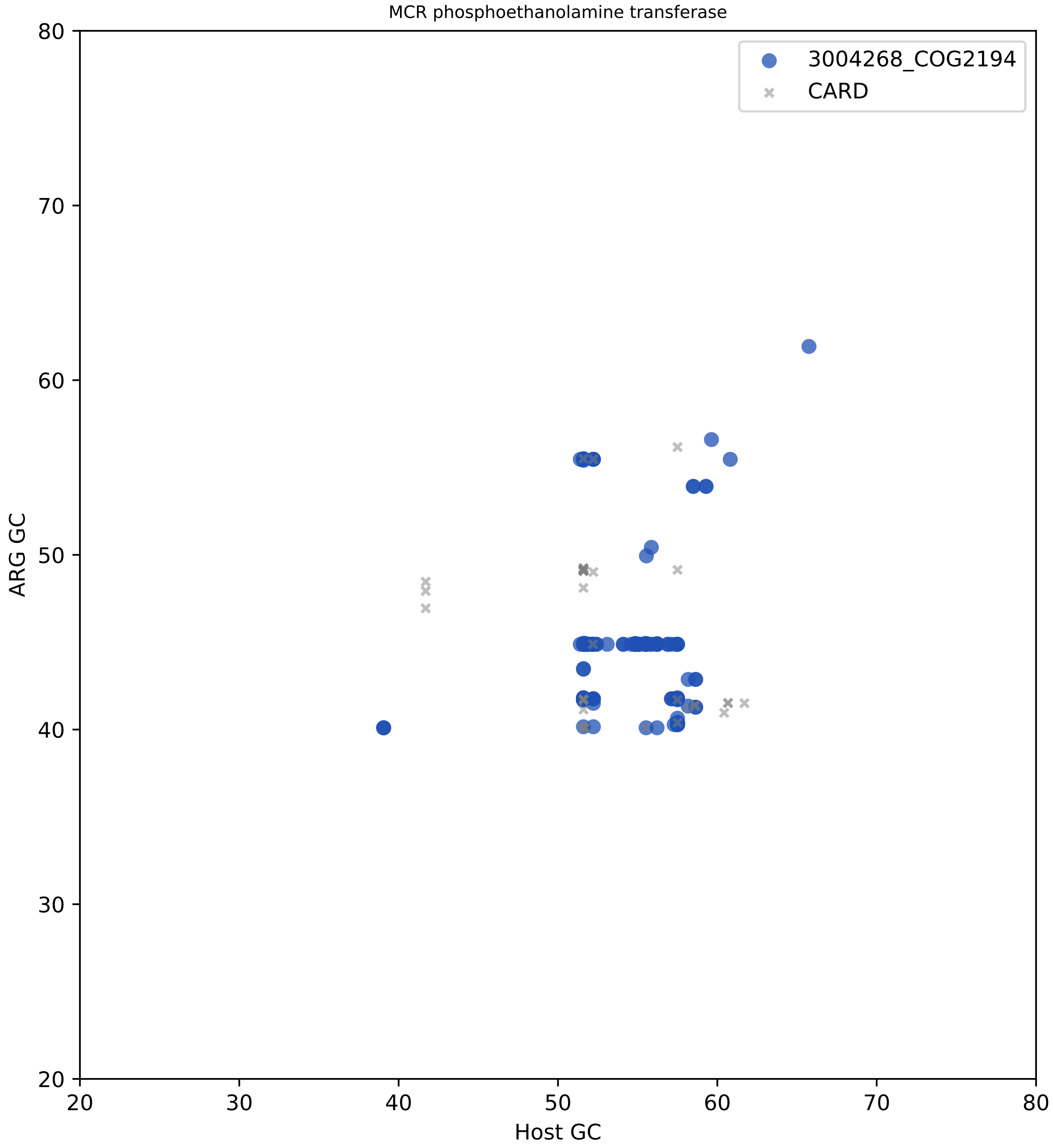

Supplementary Figure S1: (continued).

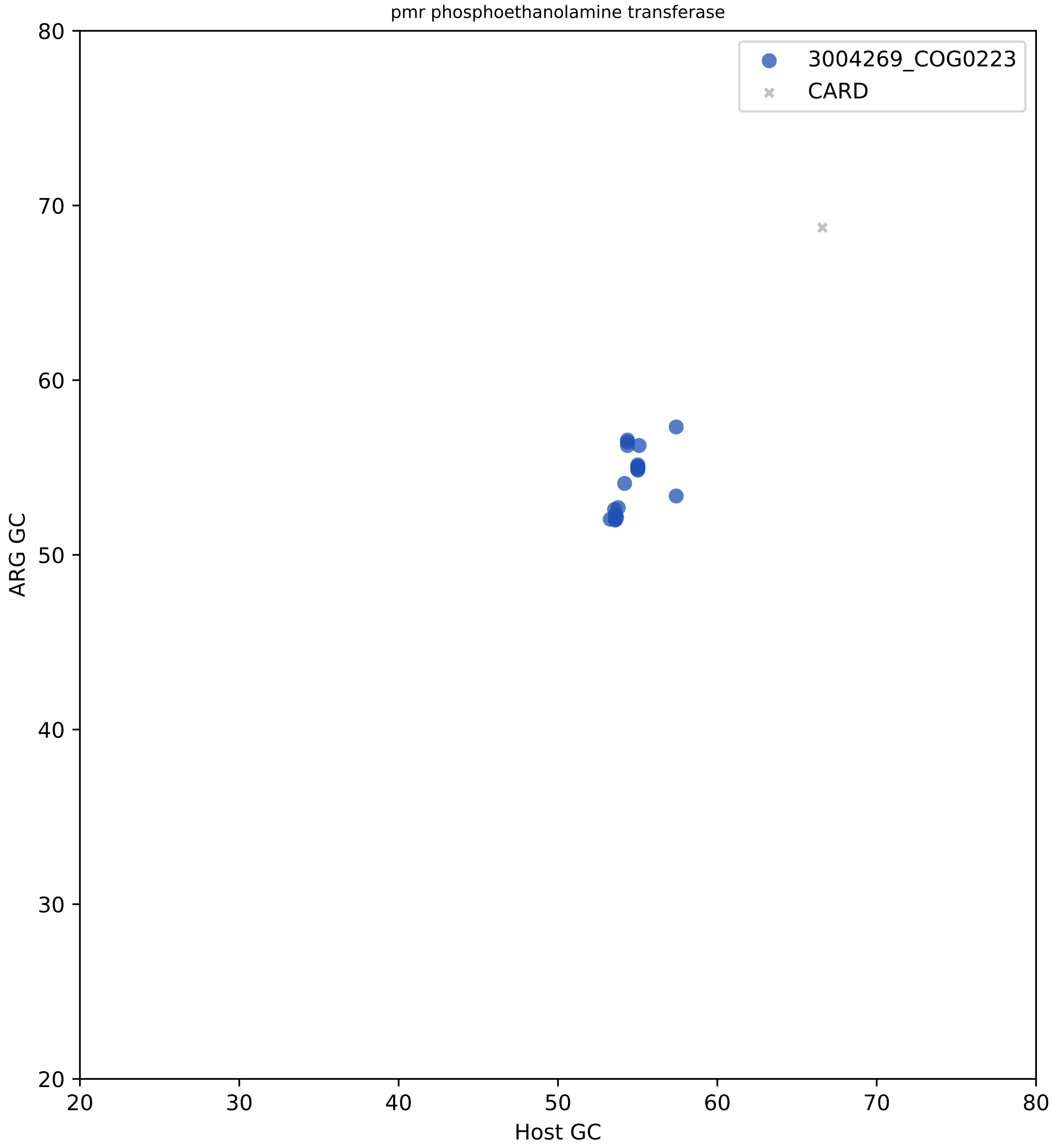

Supplementary Figure S1: (continued).

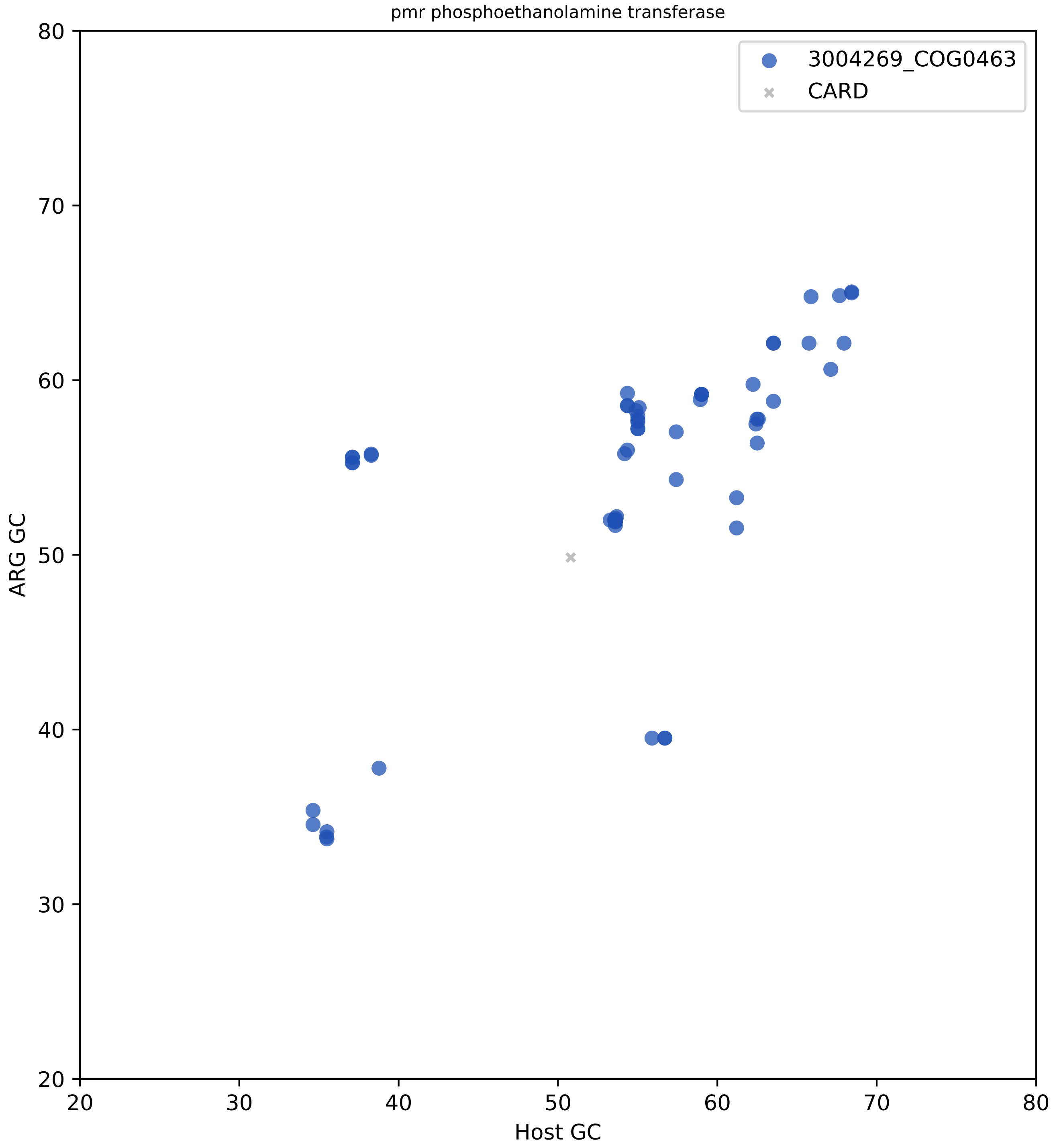

Supplementary Figure S1: (continued).

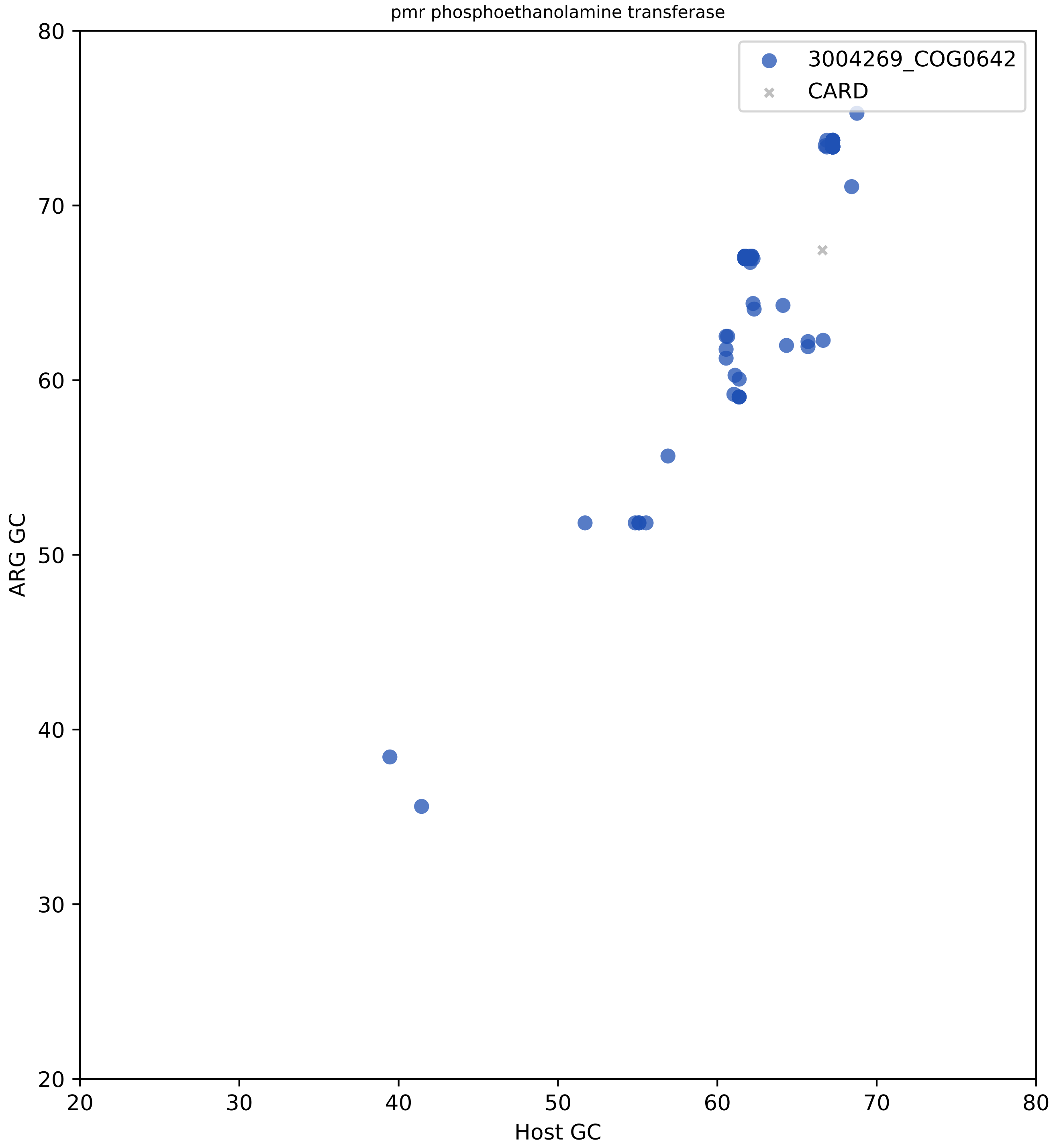

Supplementary Figure S1: (continued).

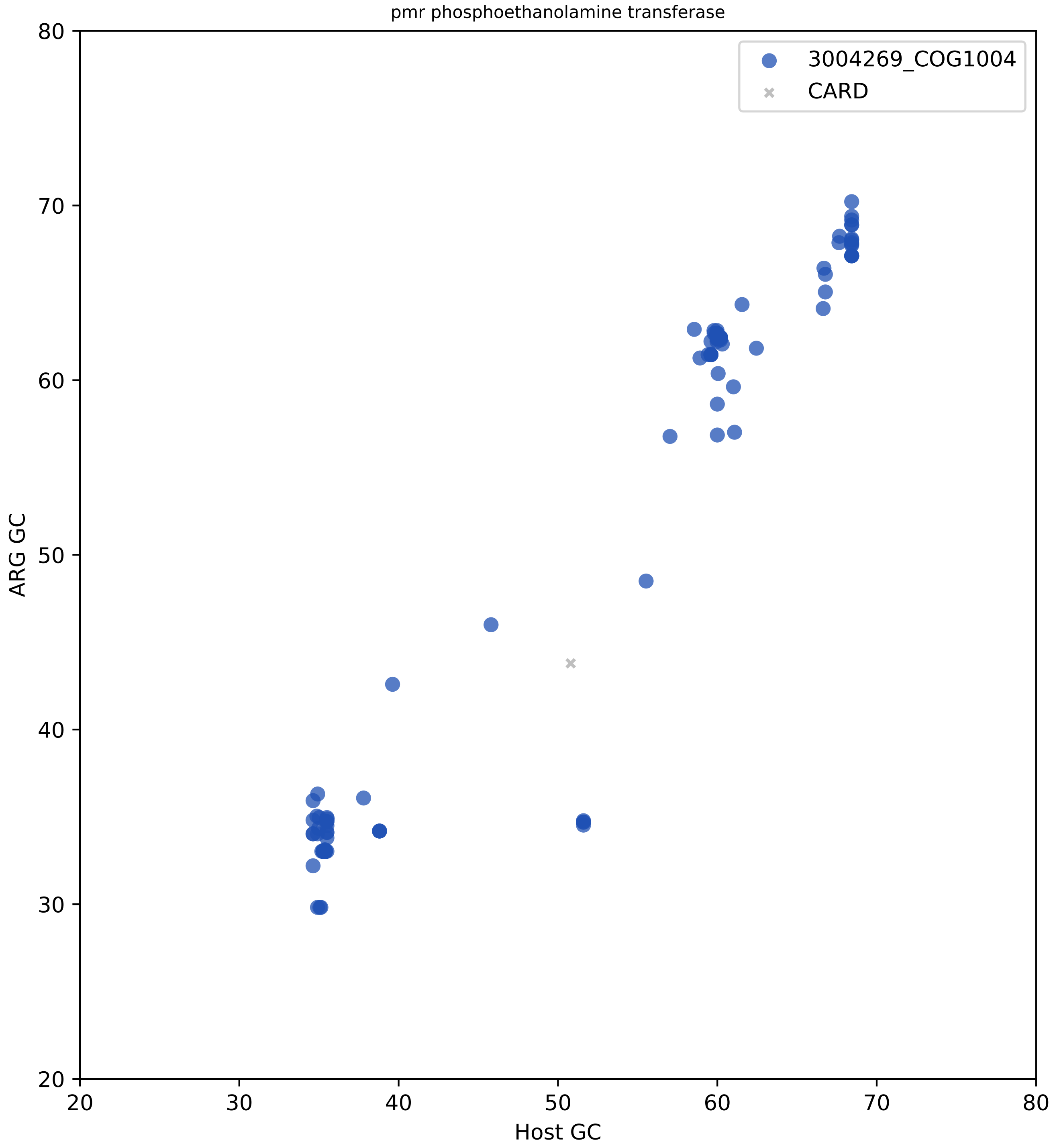

Supplementary Figure S1: (continued).

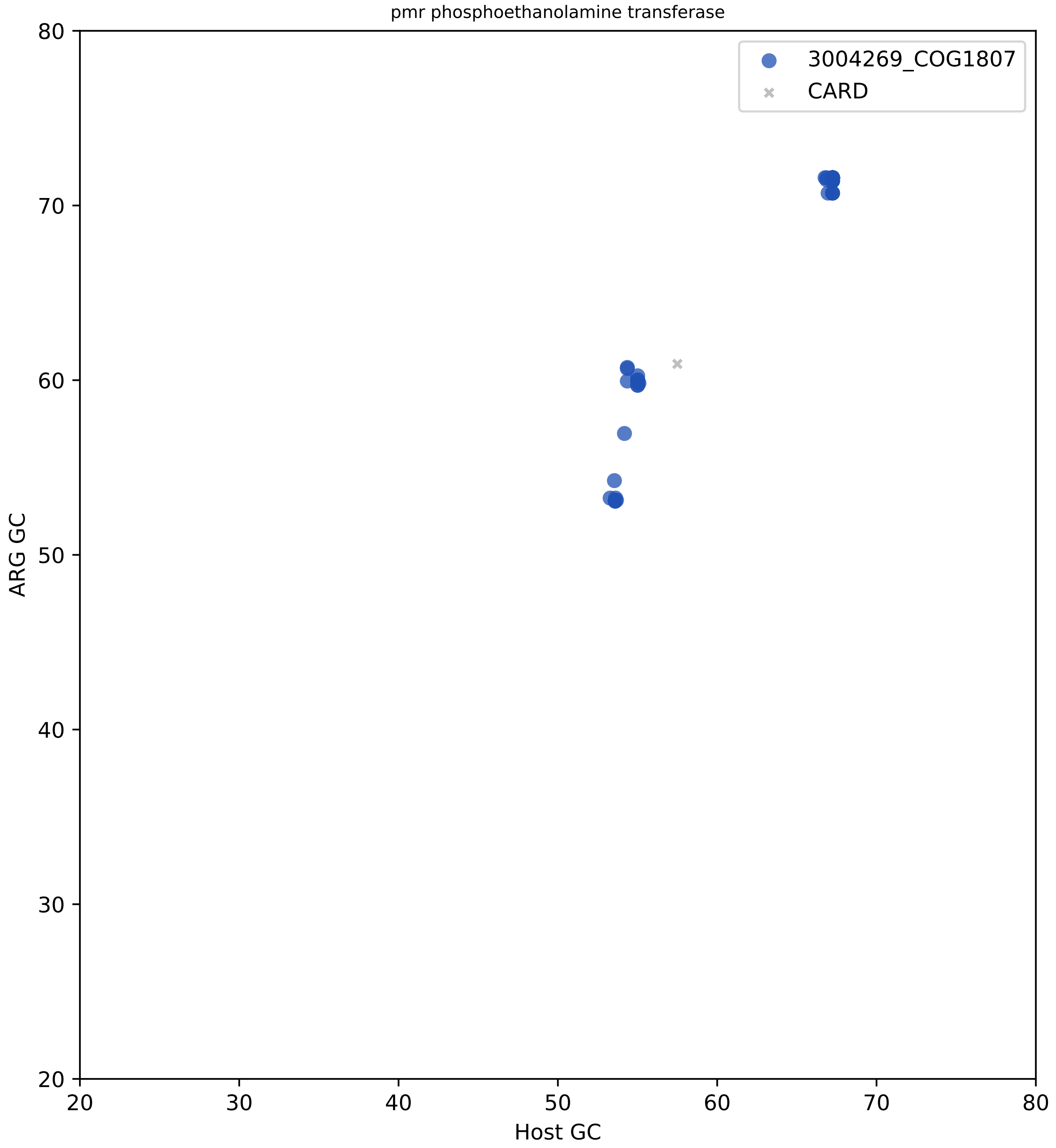

Supplementary Figure S1: (continued).

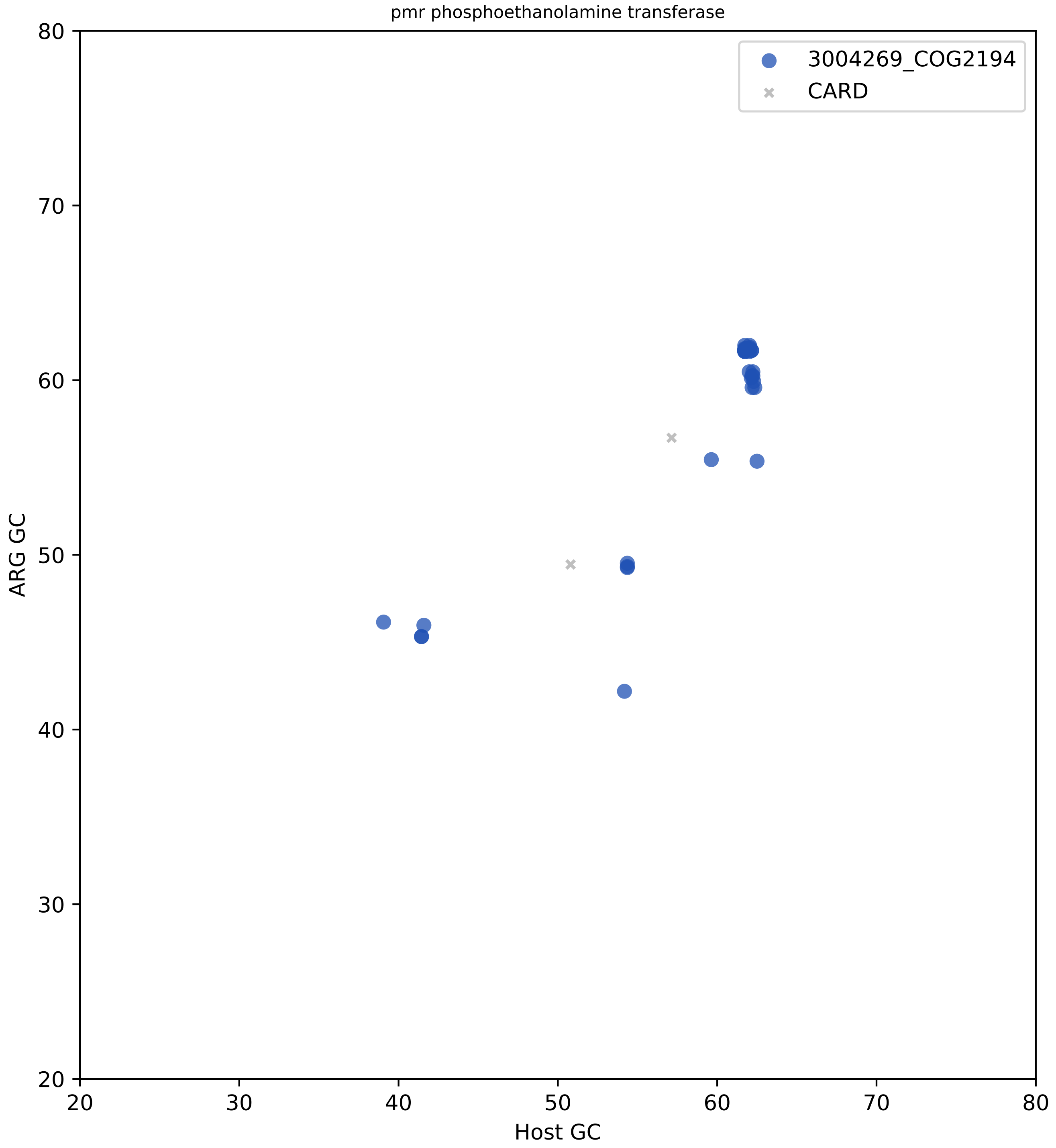

Supplementary Figure S1: (continued).

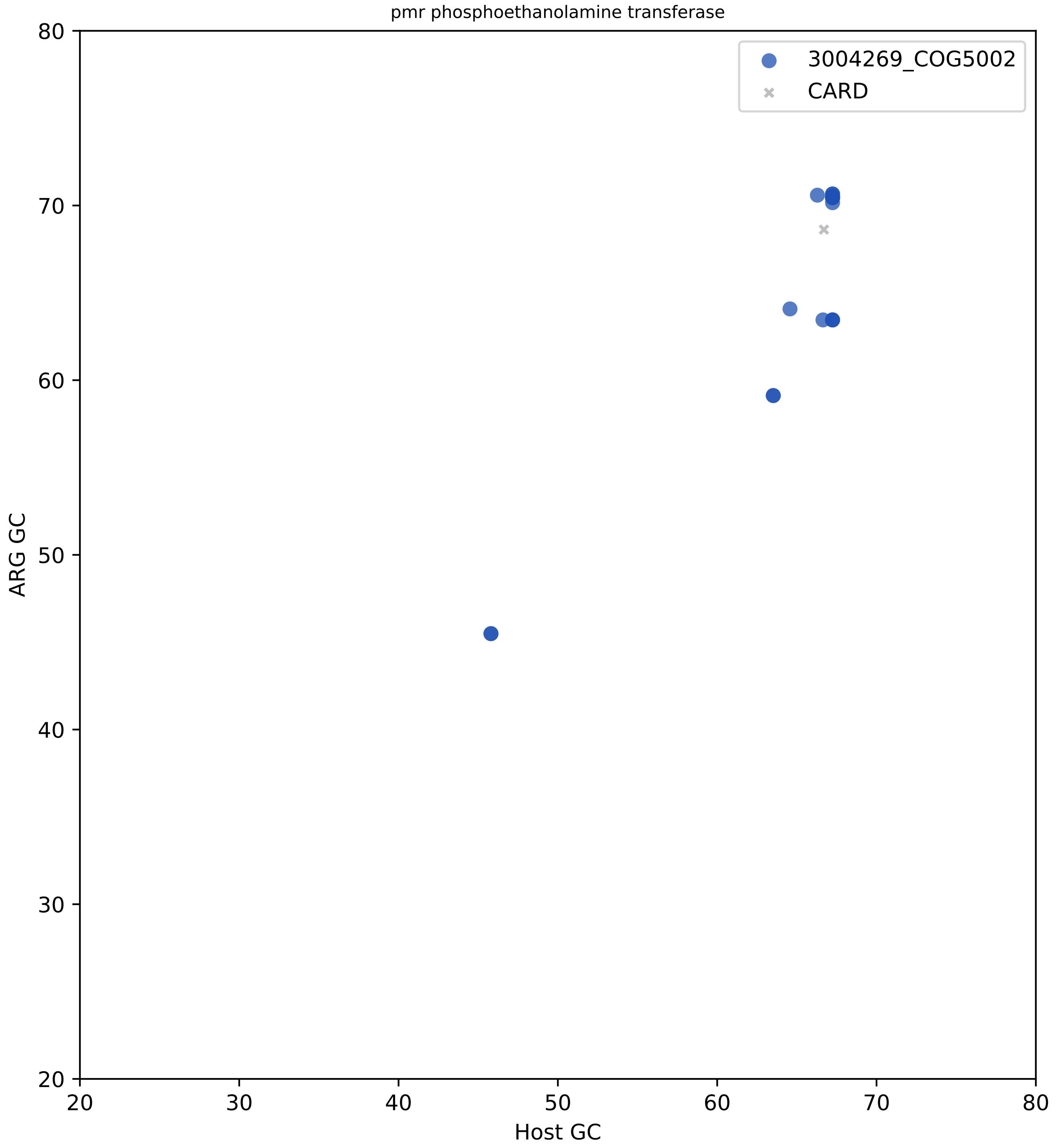

Supplementary Figure S1: (continued).

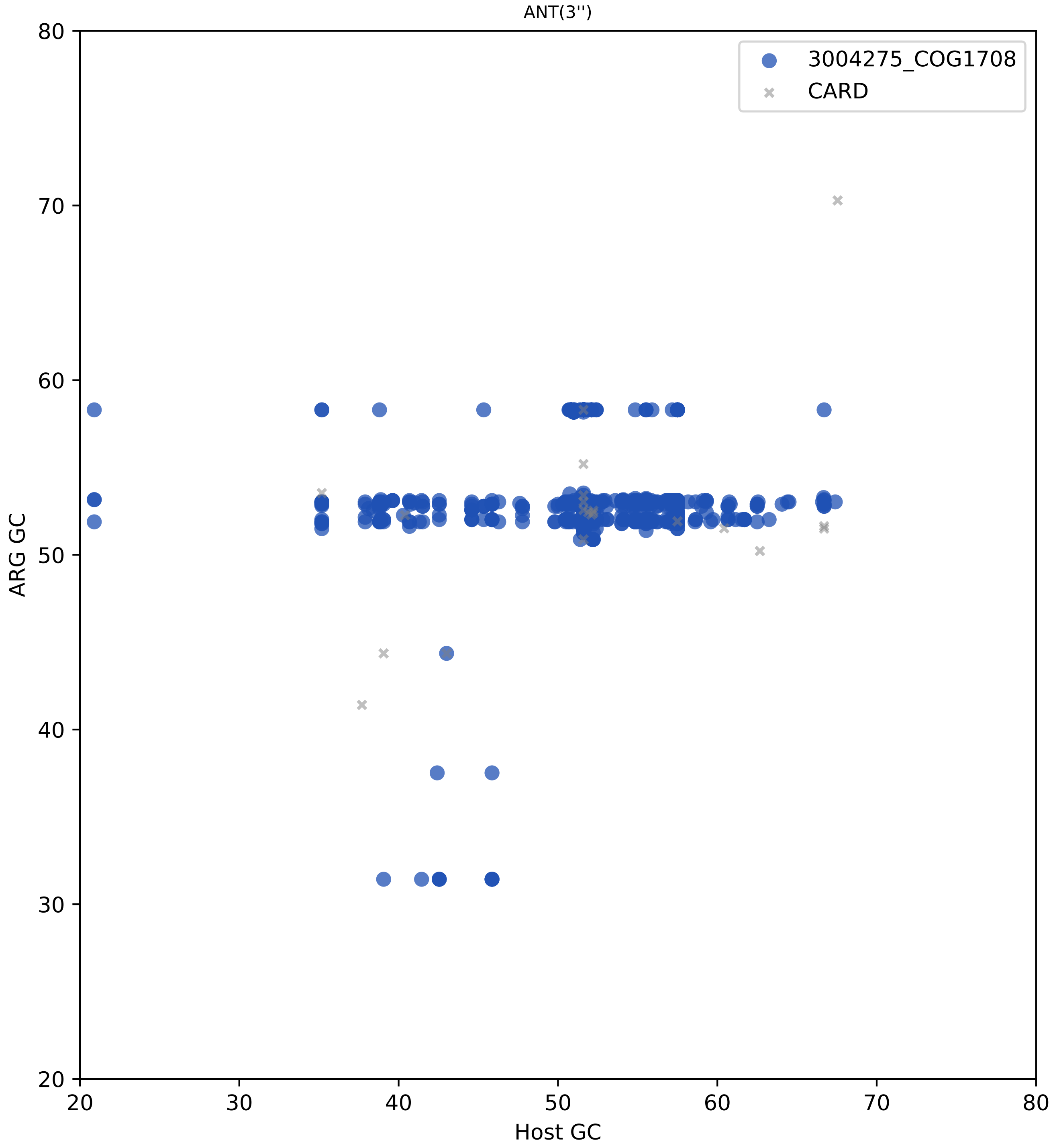

Supplementary Figure S1: (continued).

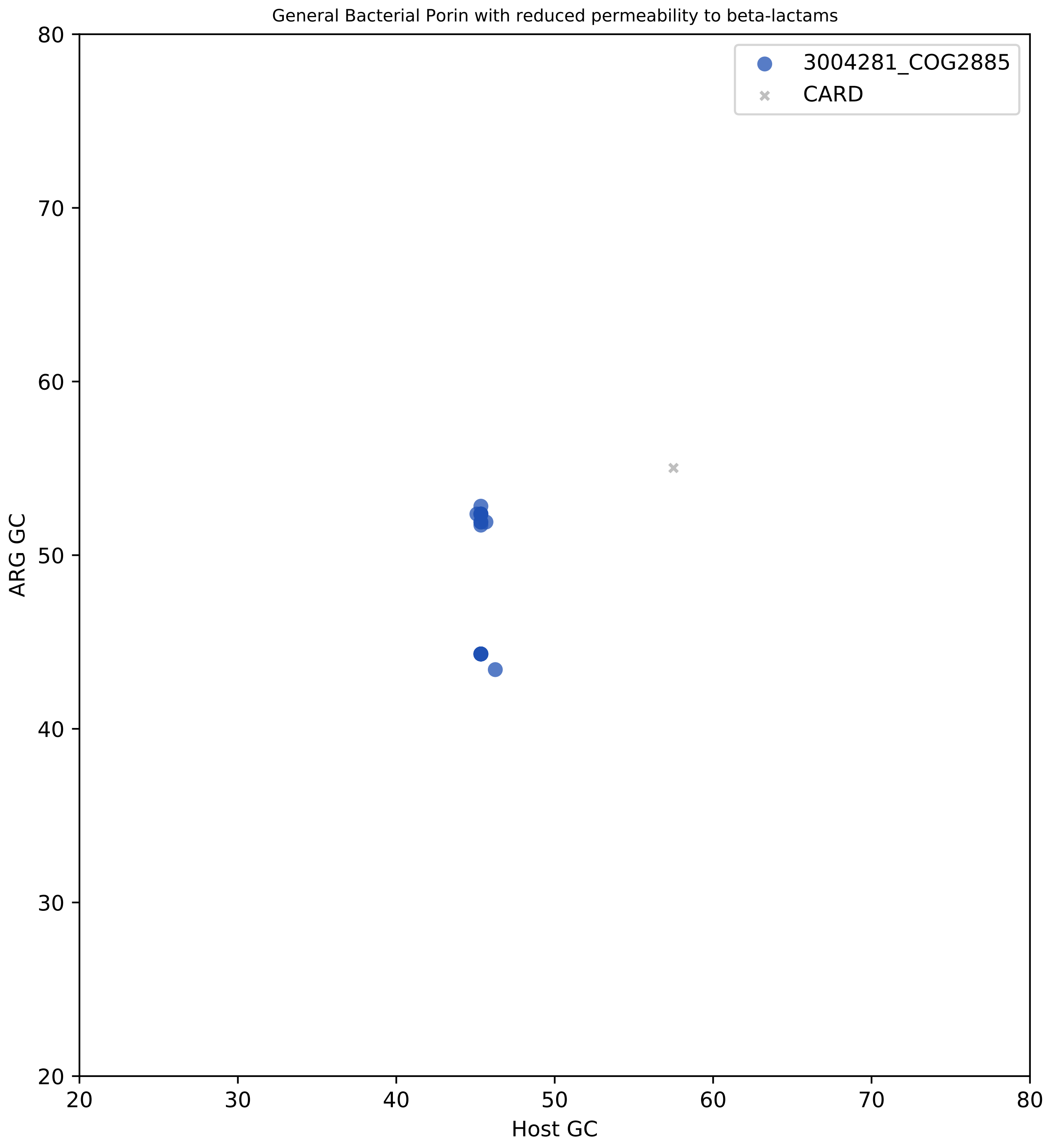

Supplementary Figure S1: (continued).

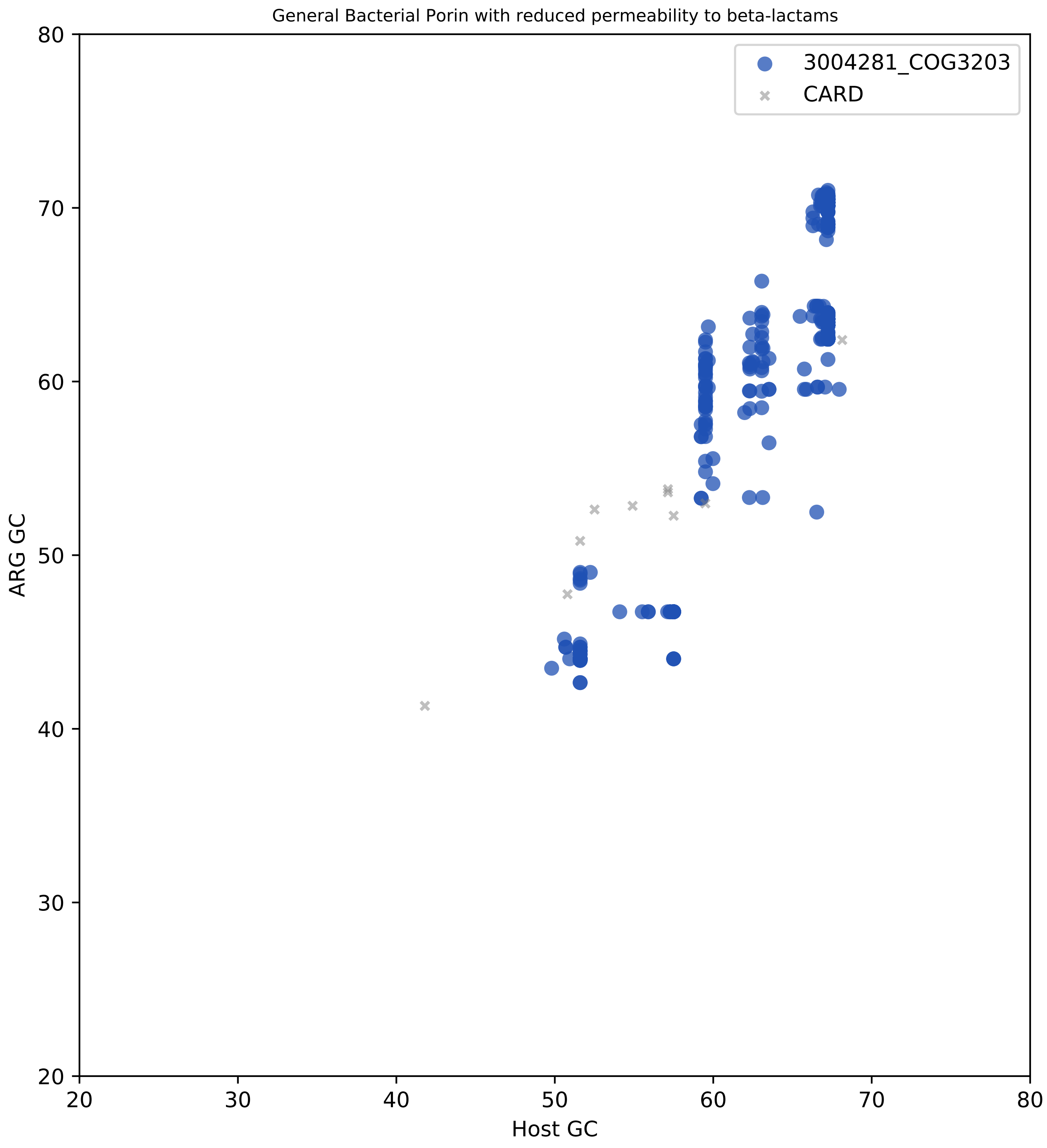

Supplementary Figure S1: (continued).

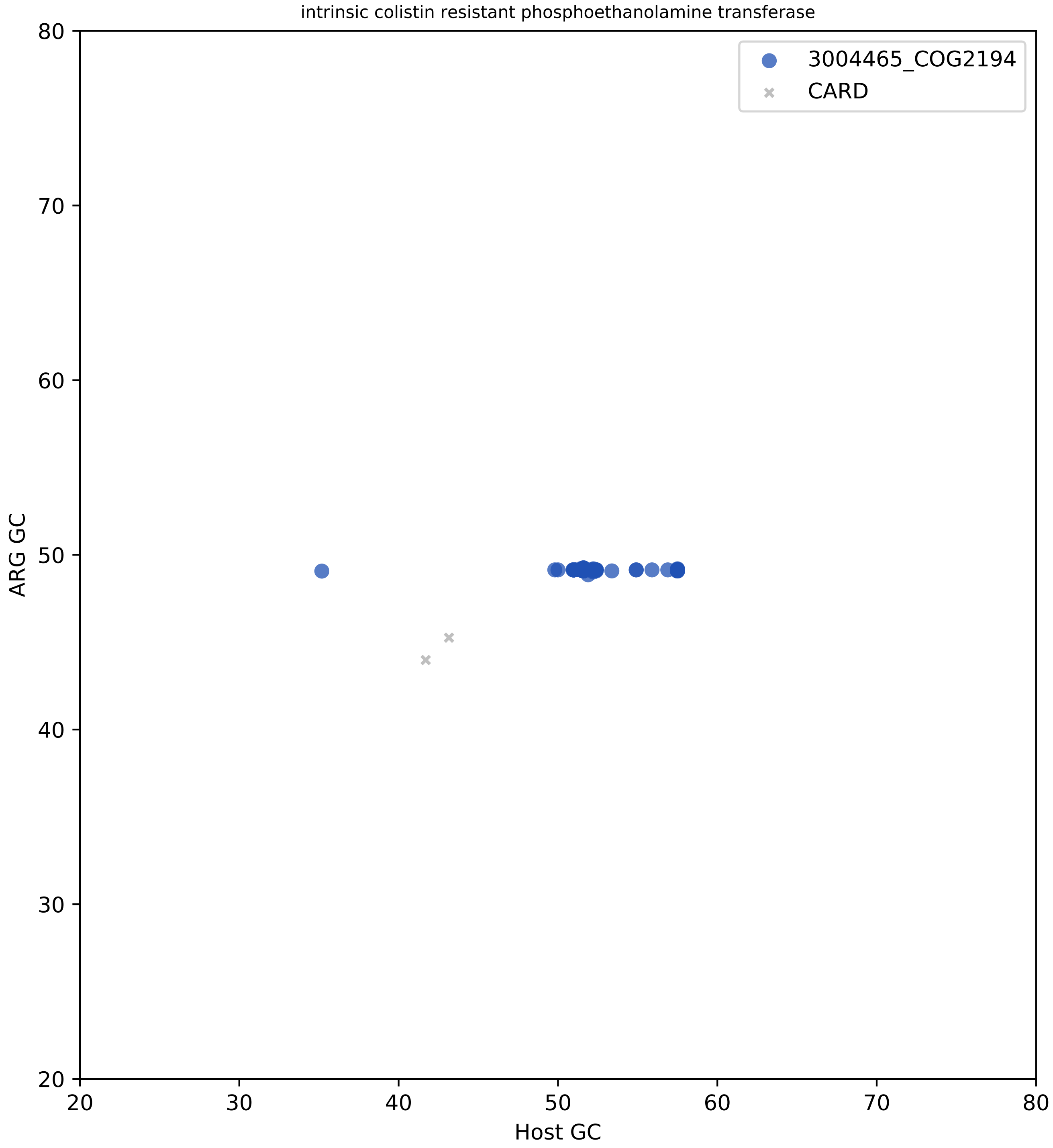

Supplementary Figure S1: (continued).

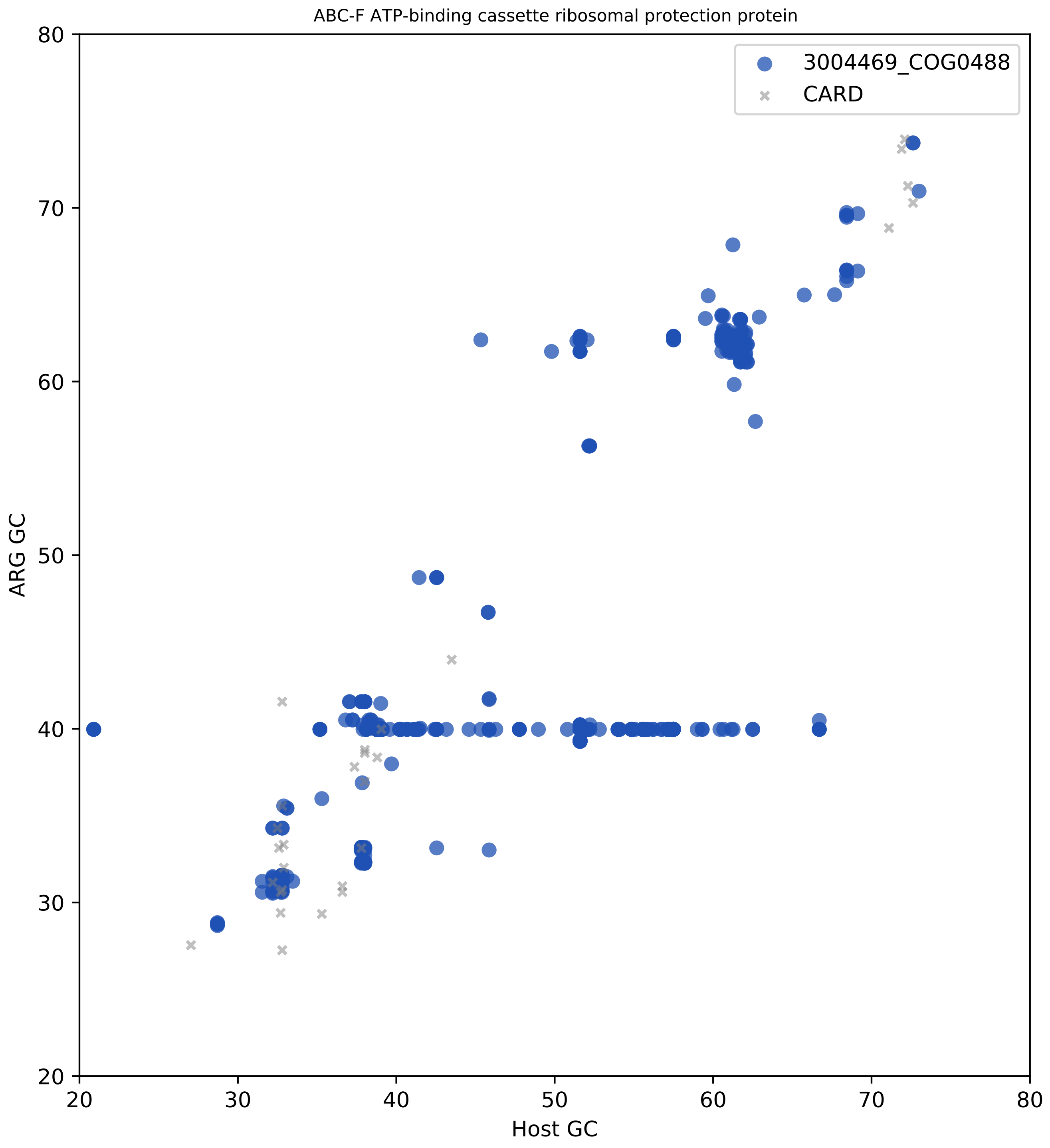

Supplementary Figure S1: (continued).

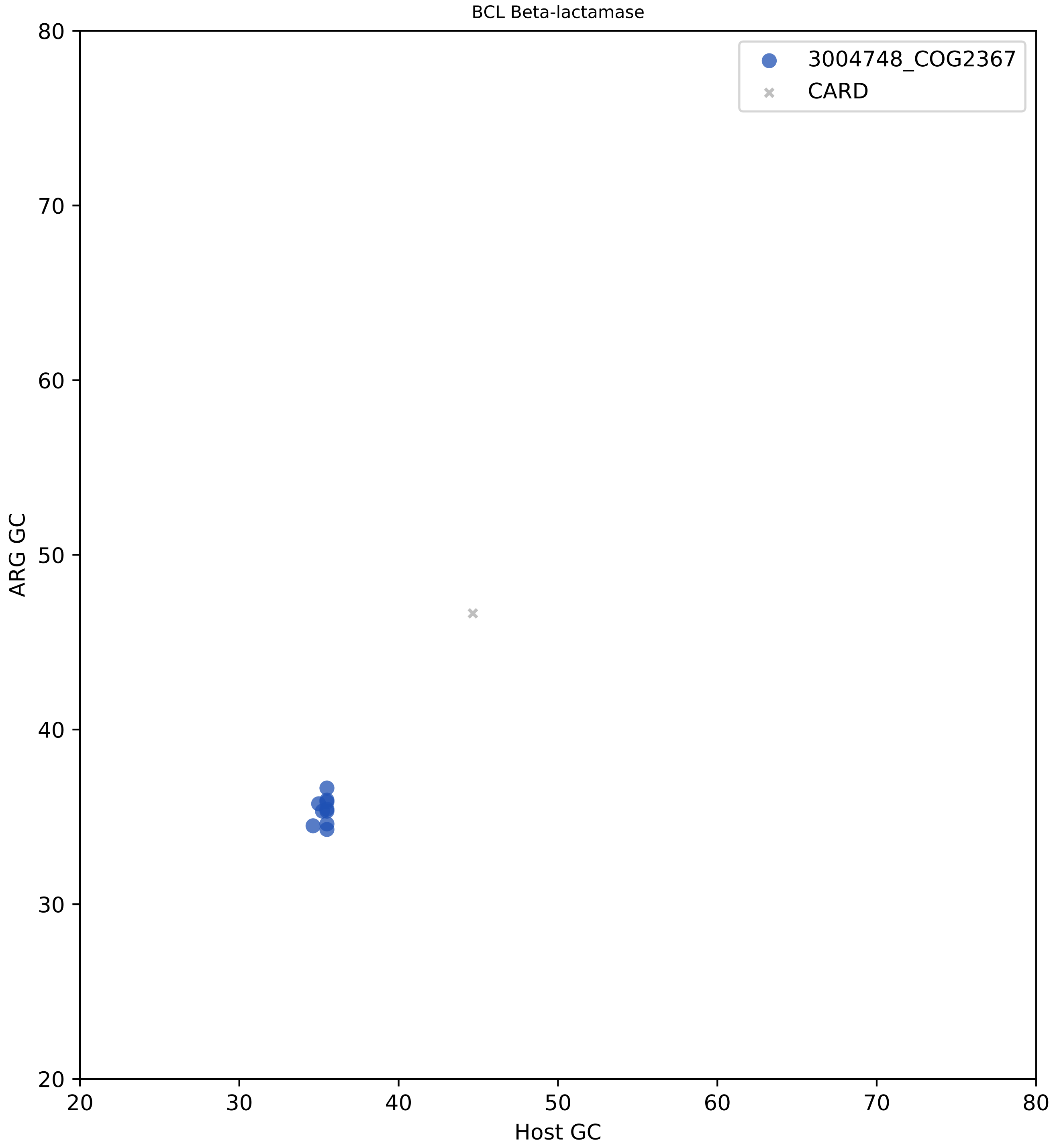

Supplementary Figure S1: (continued).

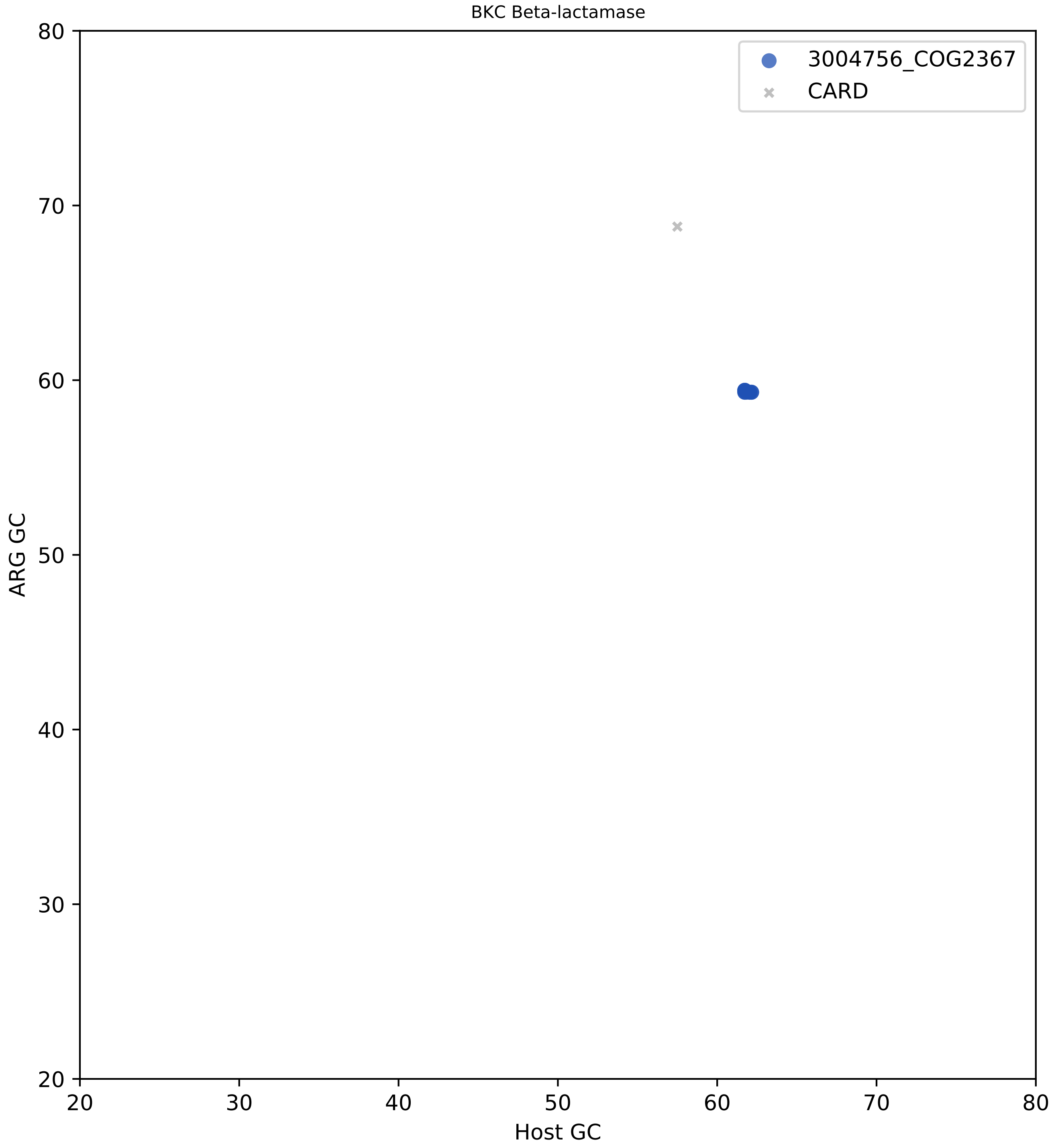

Supplementary Figure S1: (continued).

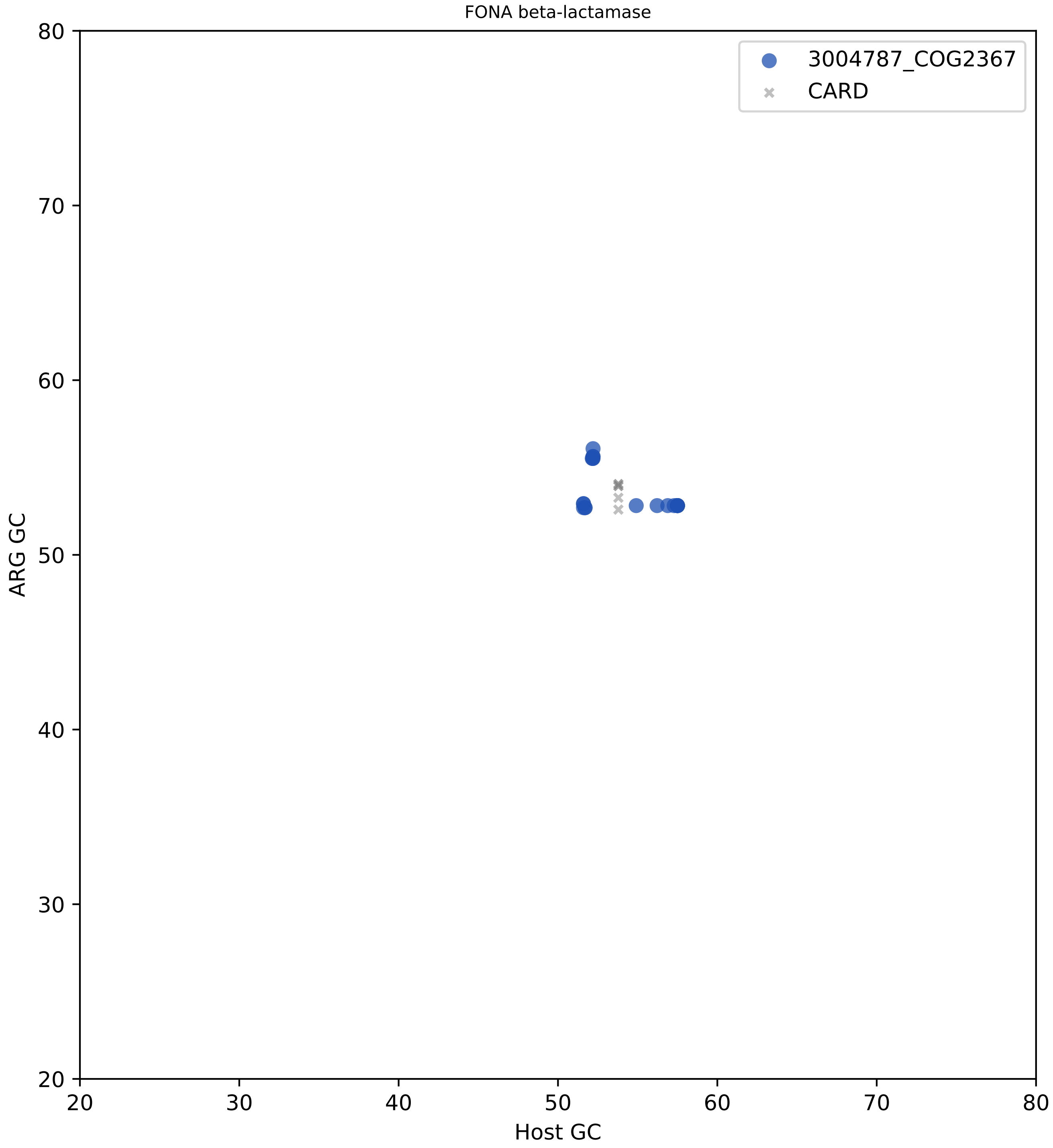

Supplementary Figure S1: (continued).

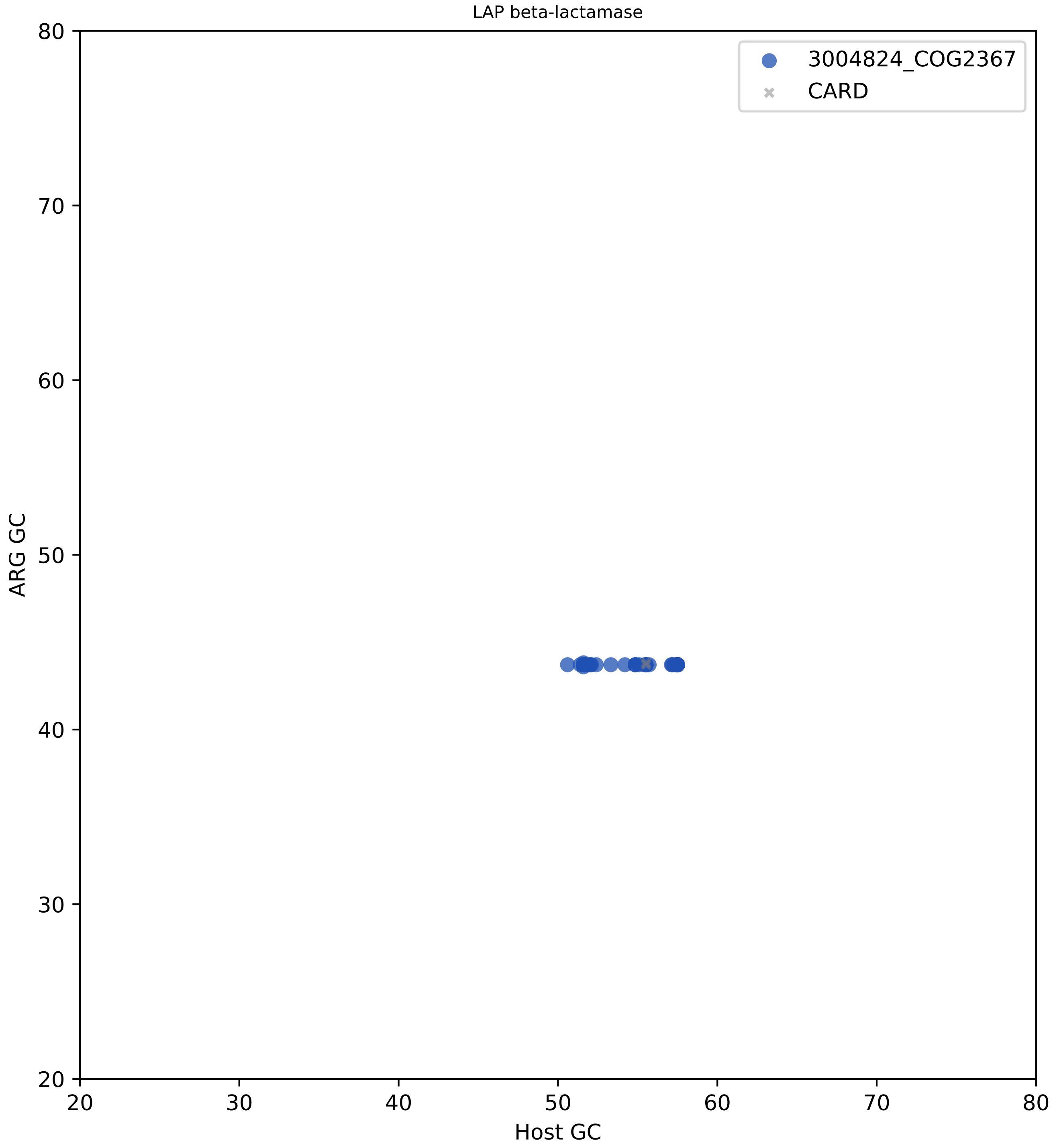

Supplementary Figure S1: (continued).

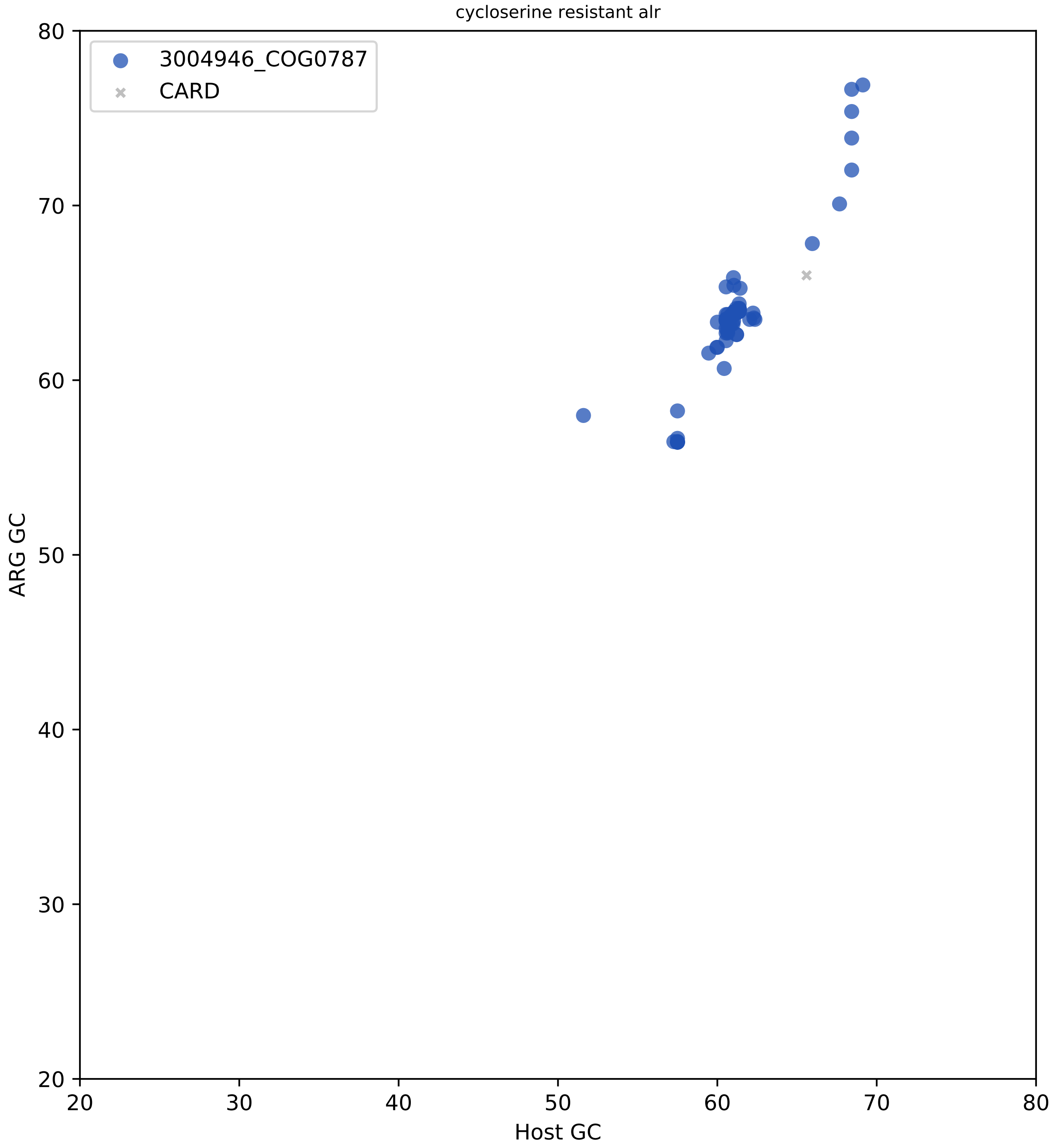

Supplementary Figure S1: (continued).

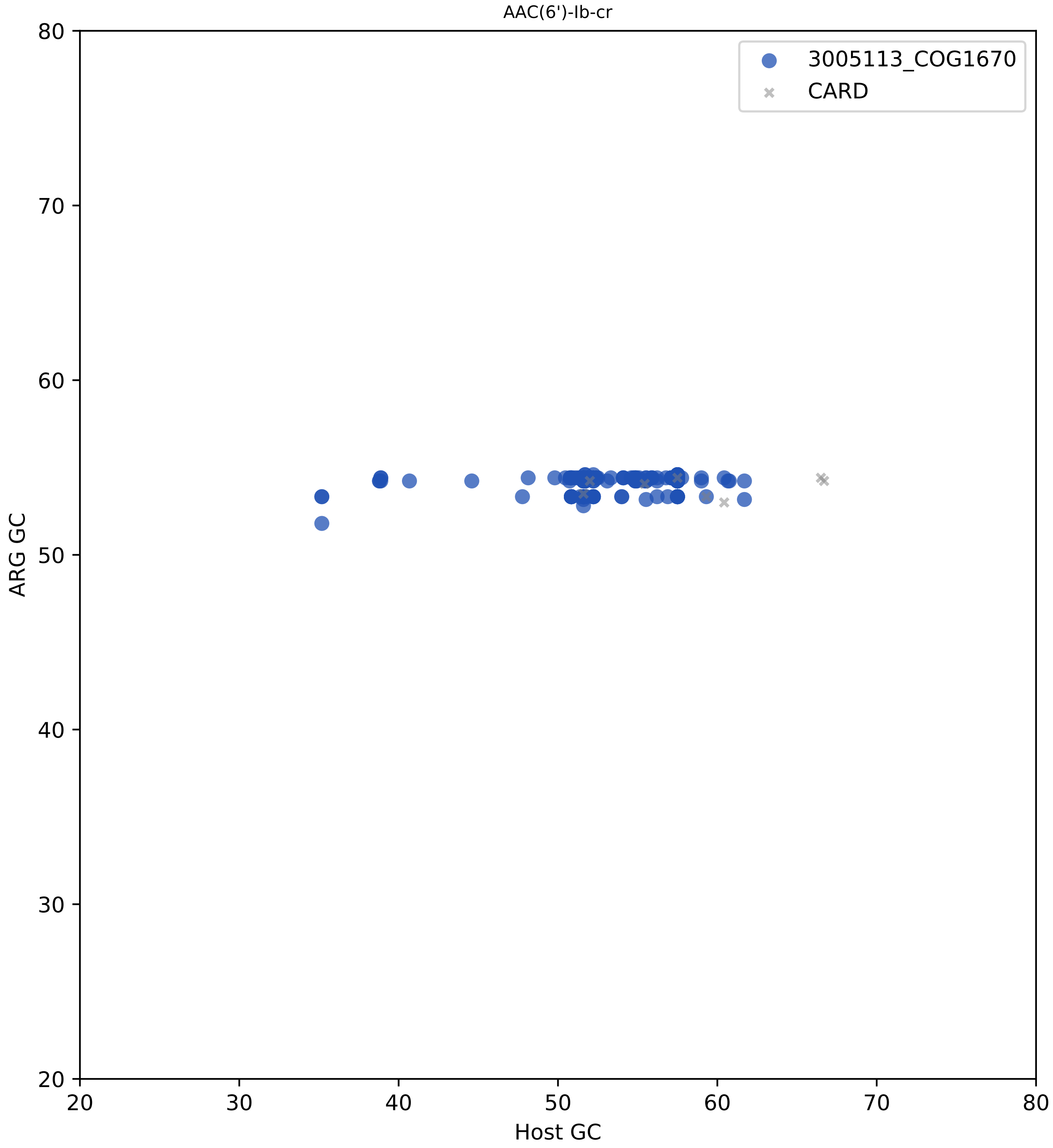

Supplementary Figure S1: (continued).

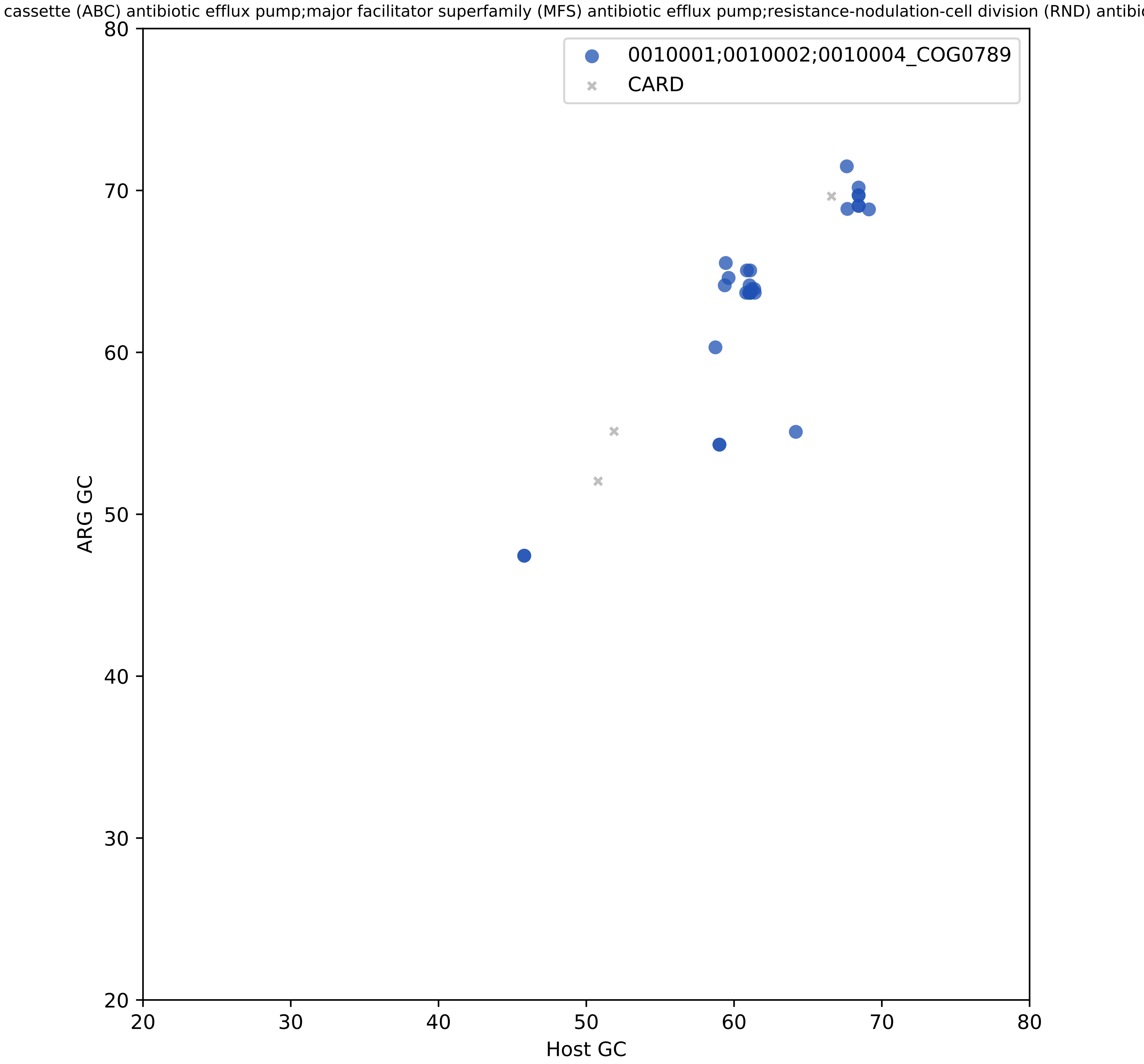

Supplementary Figure S1: (continued).

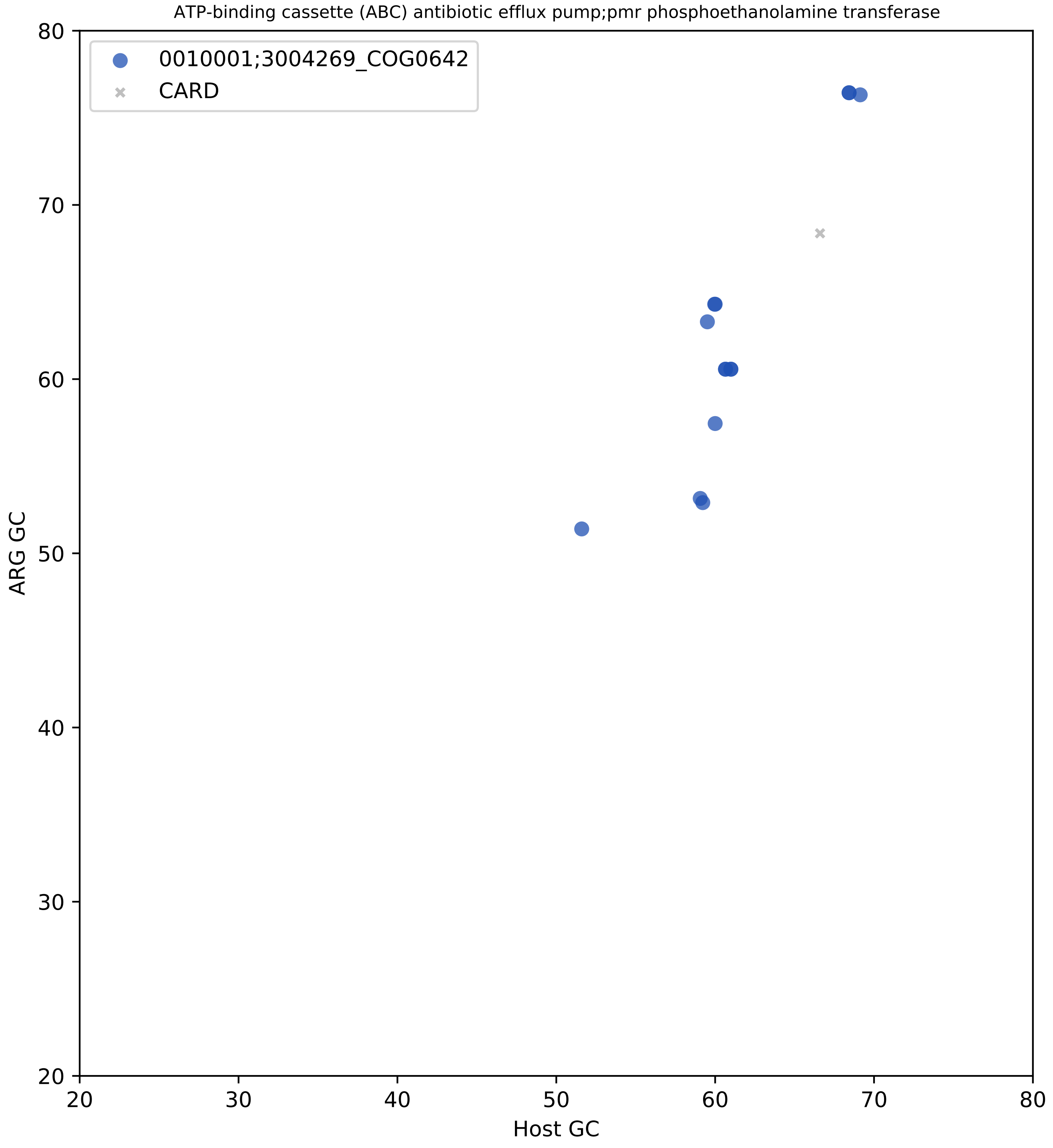

Supplementary Figure S1: (continued).

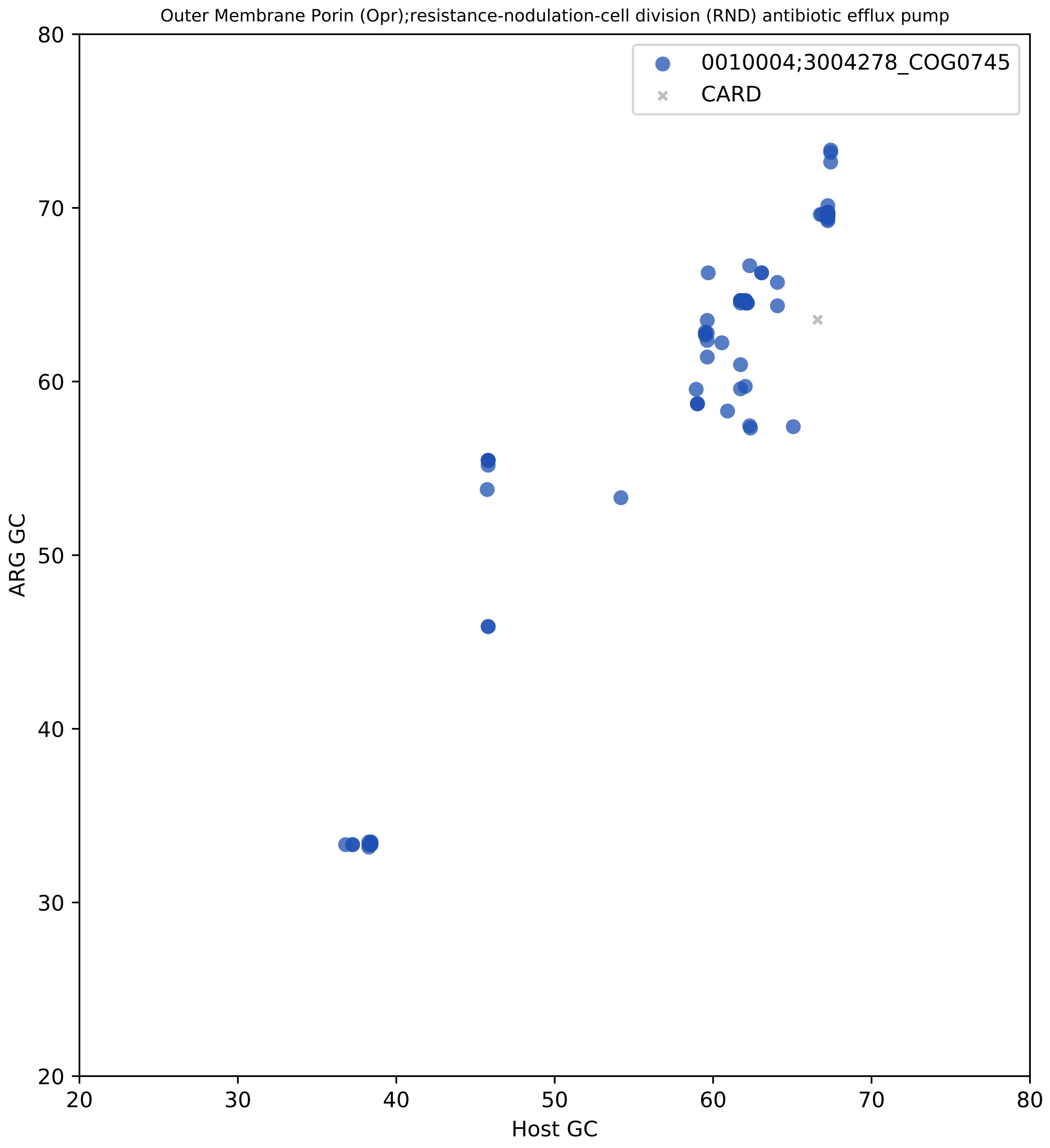

Supplementary Figure S1: (continued).

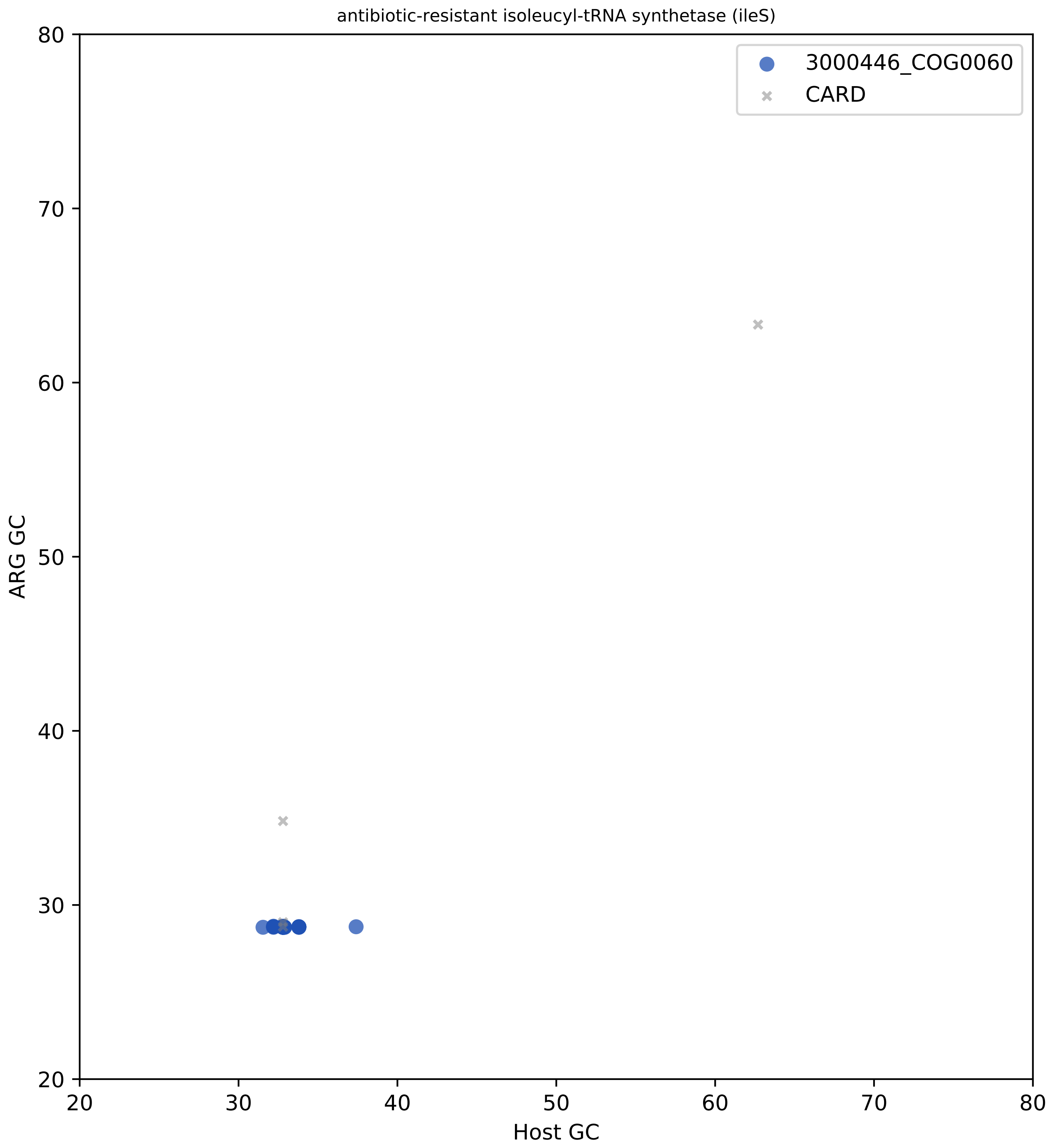

Supplementary Figure S1: (continued).

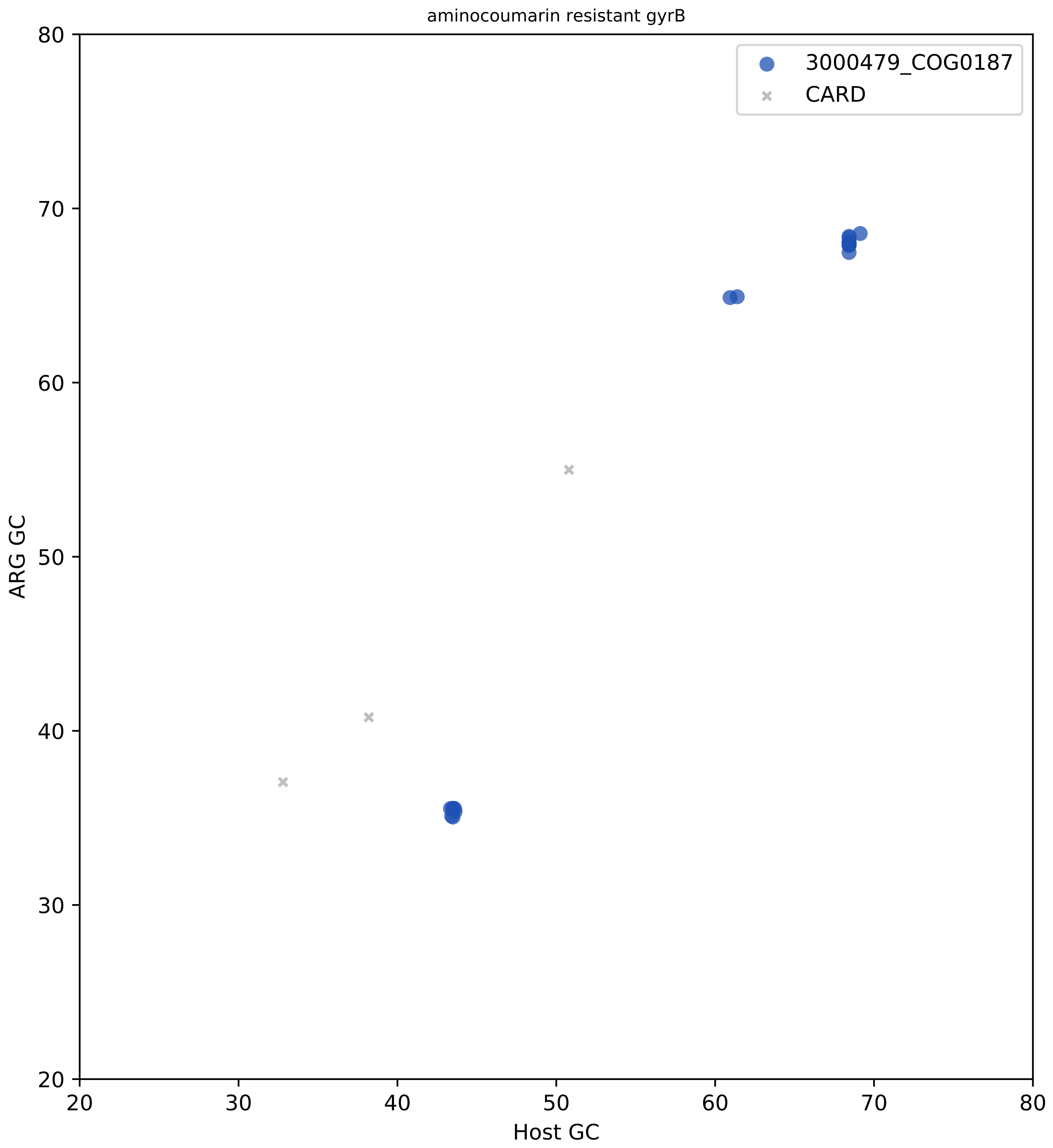

Supplementary Figure S1: (continued).

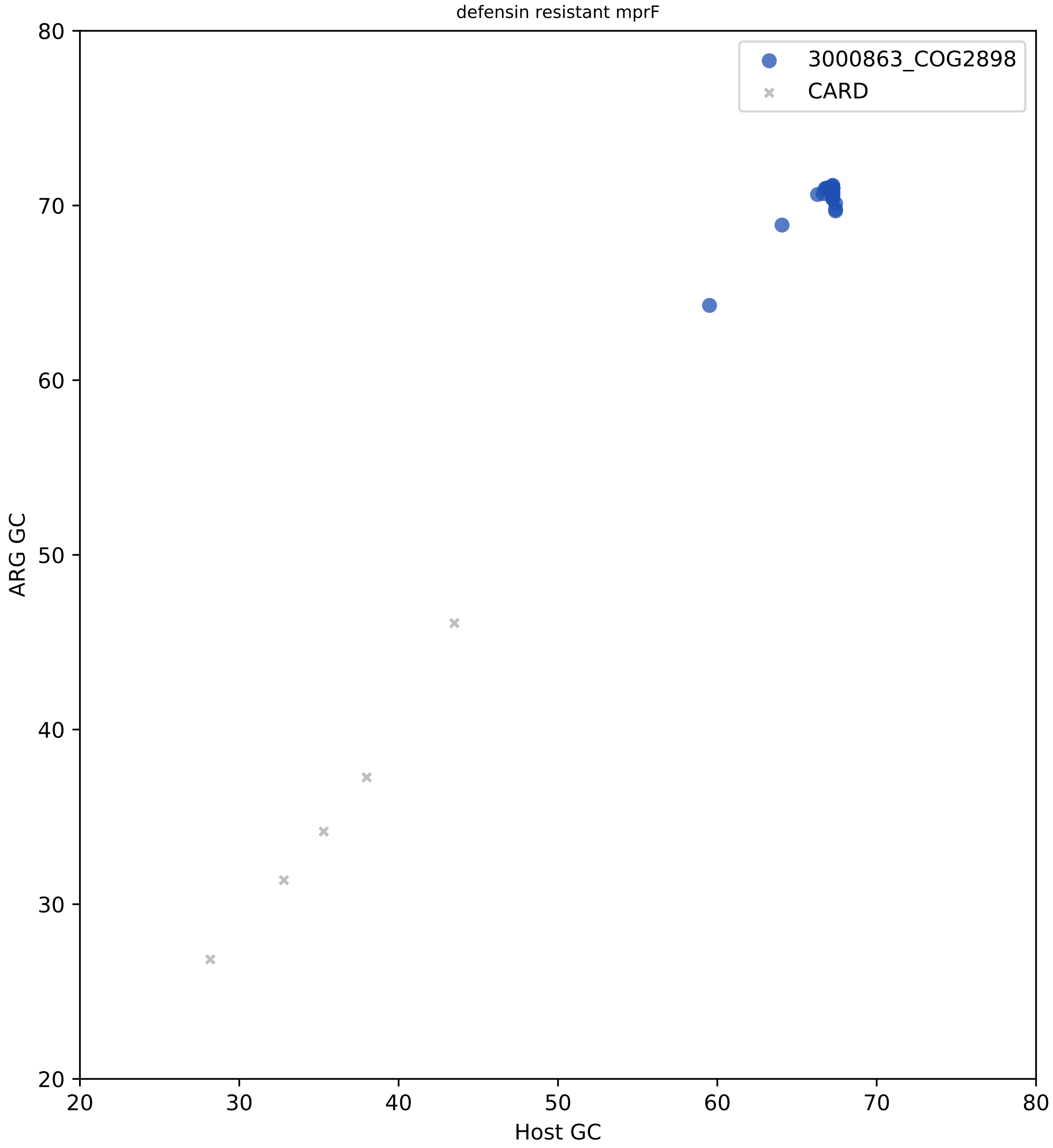

Supplementary Figure S1: (continued).

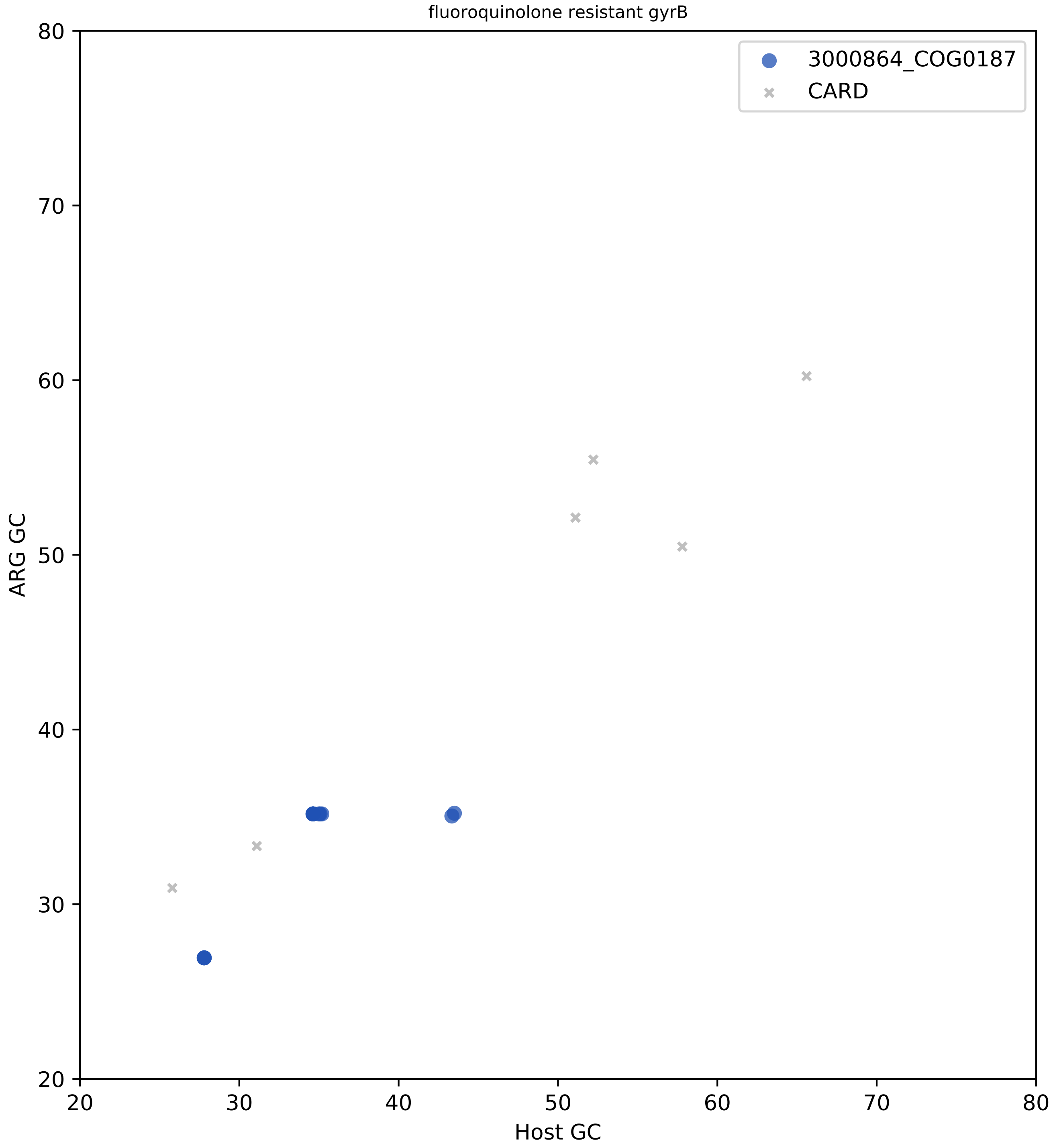

Supplementary Figure S1: (continued).

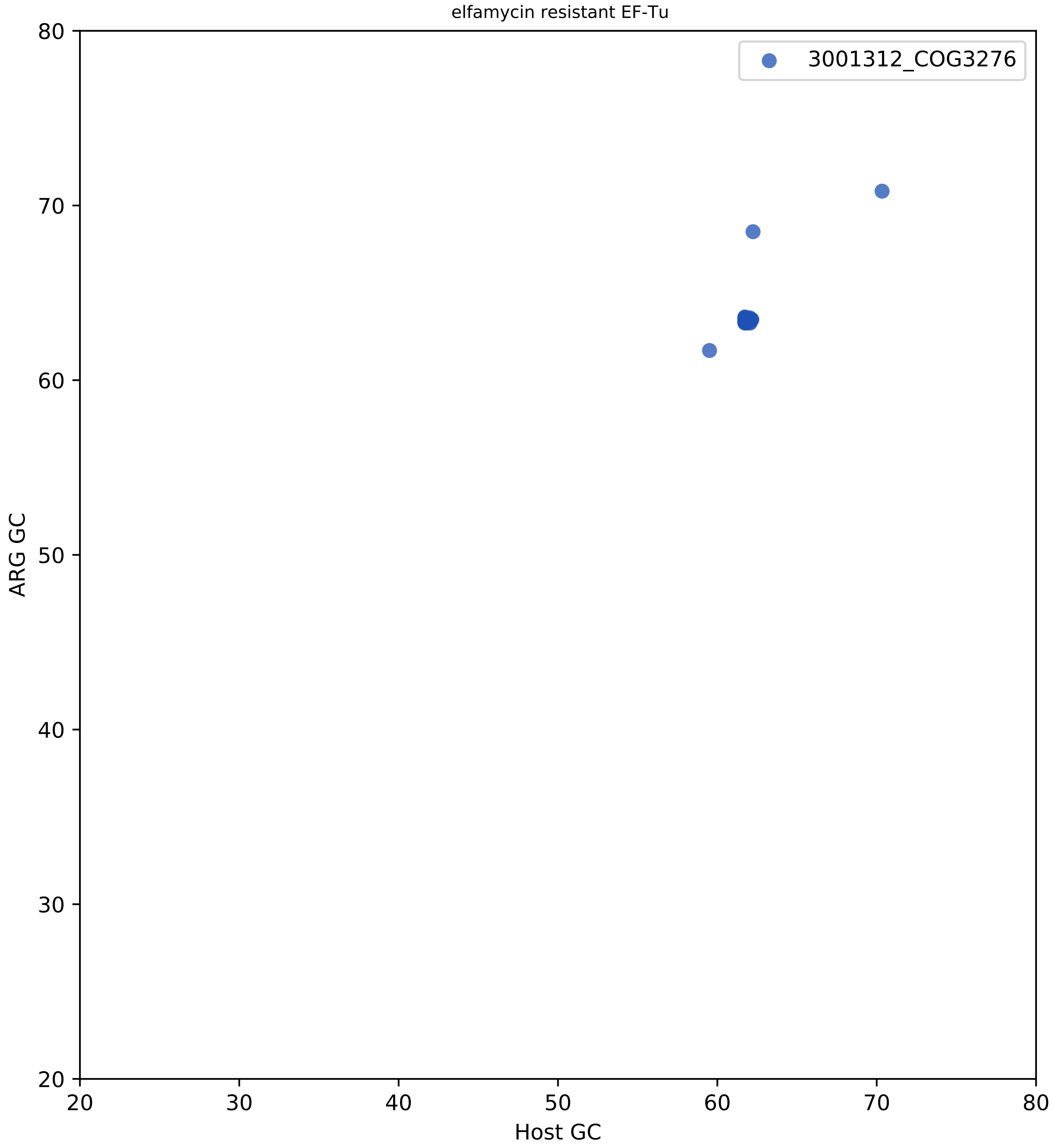

Supplementary Figure S1: (continued).

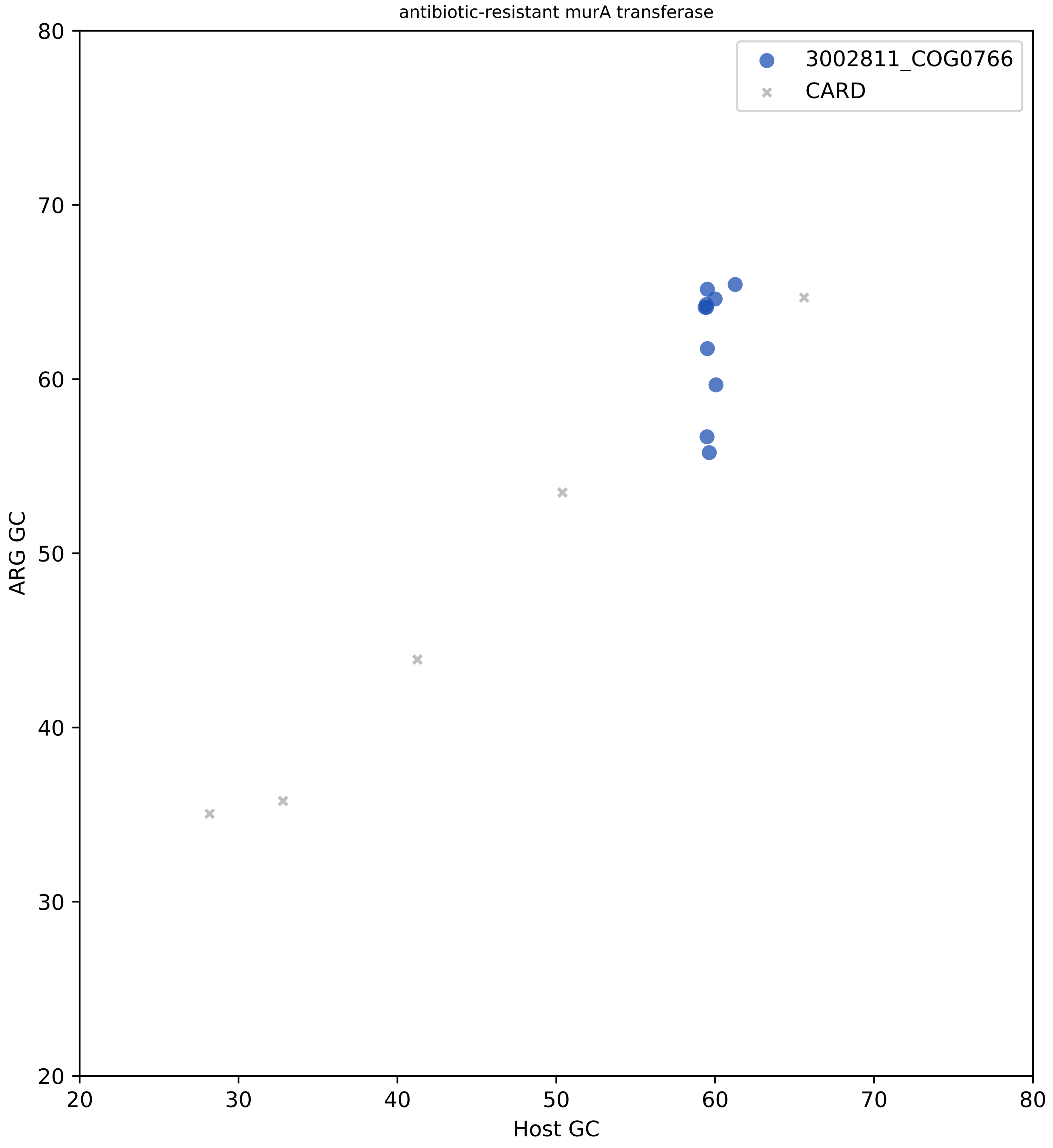

Supplementary Figure S1: (continued).

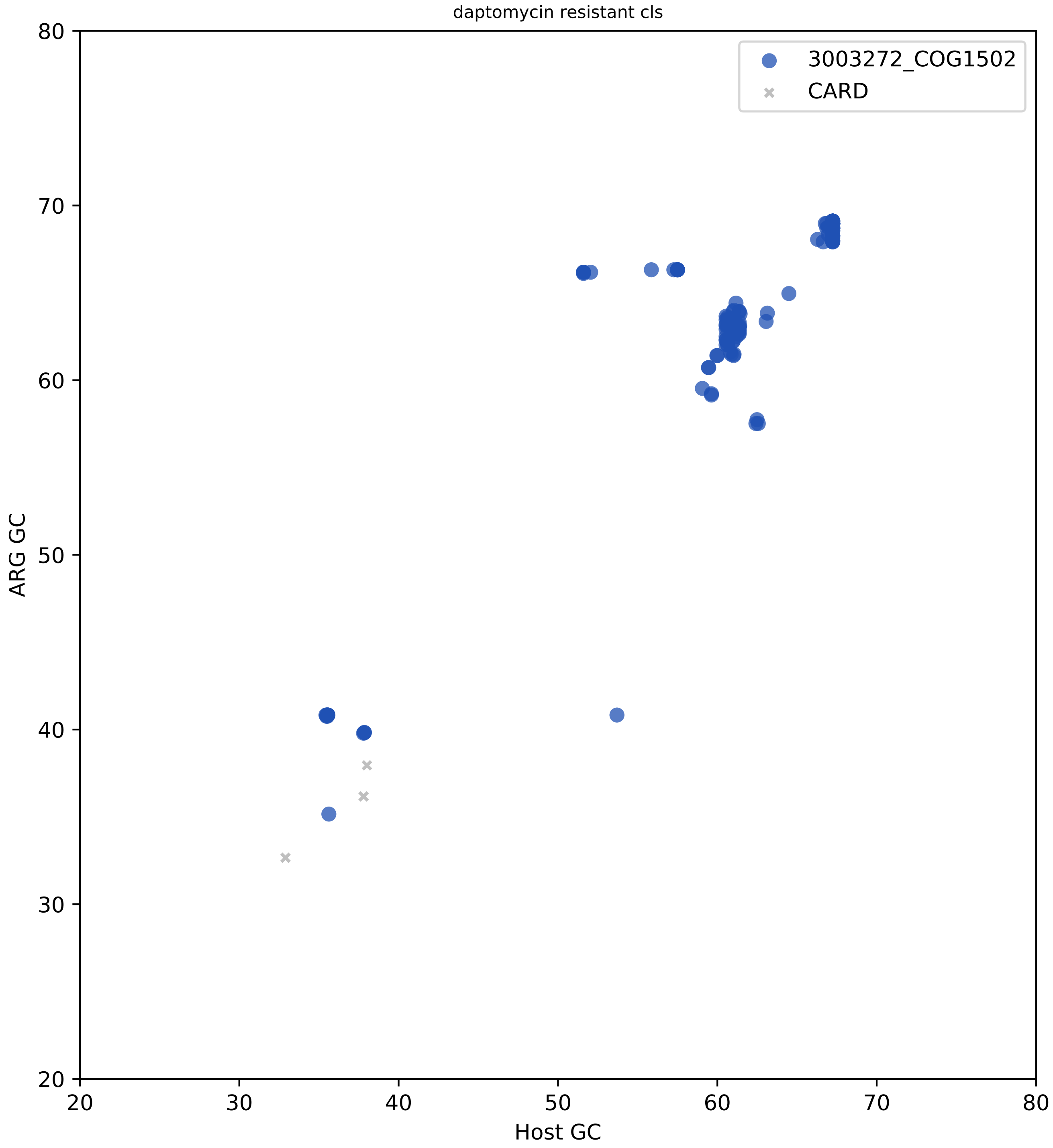

Supplementary Figure S1: (continued).

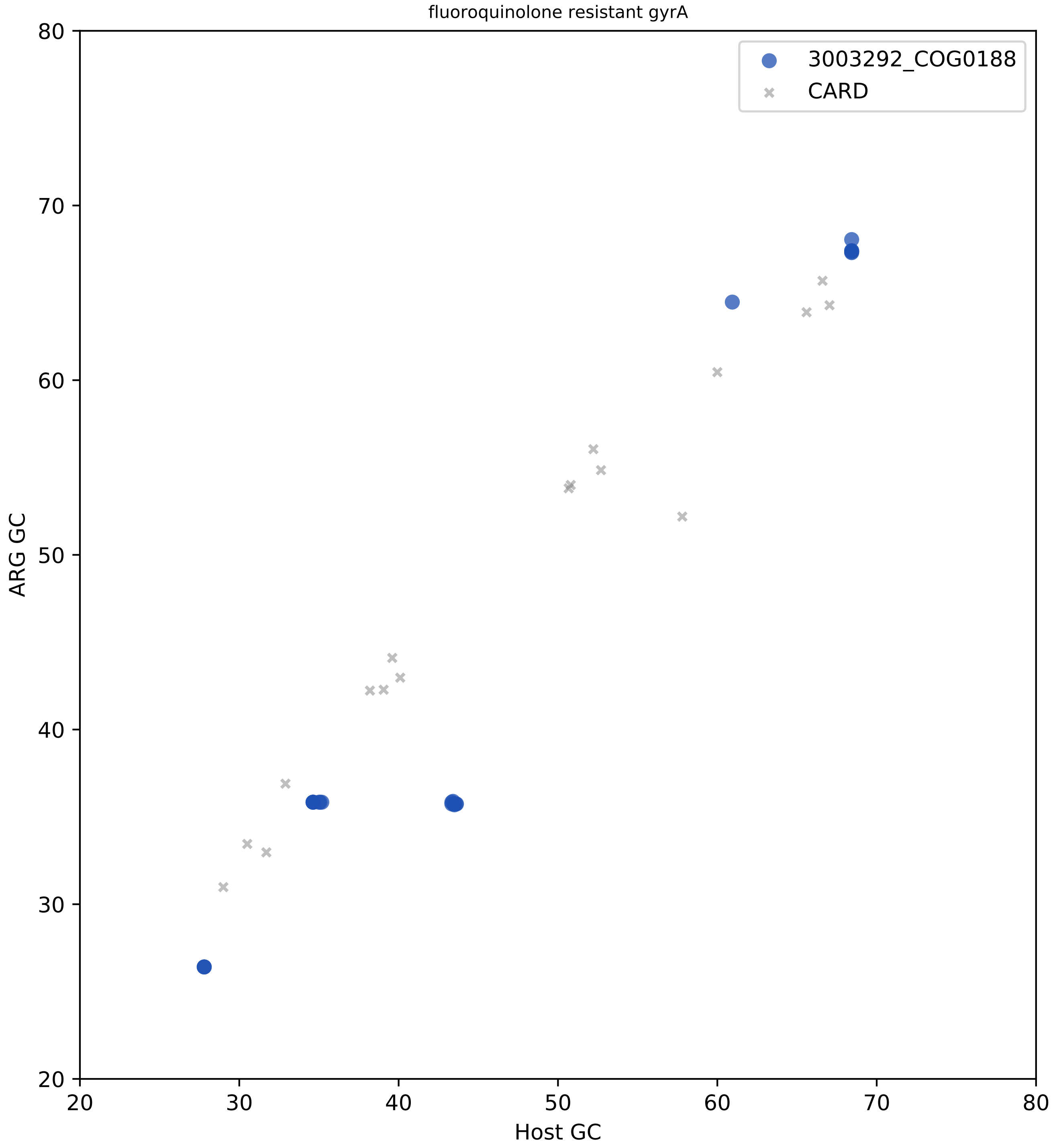

Supplementary Figure S1: (continued).

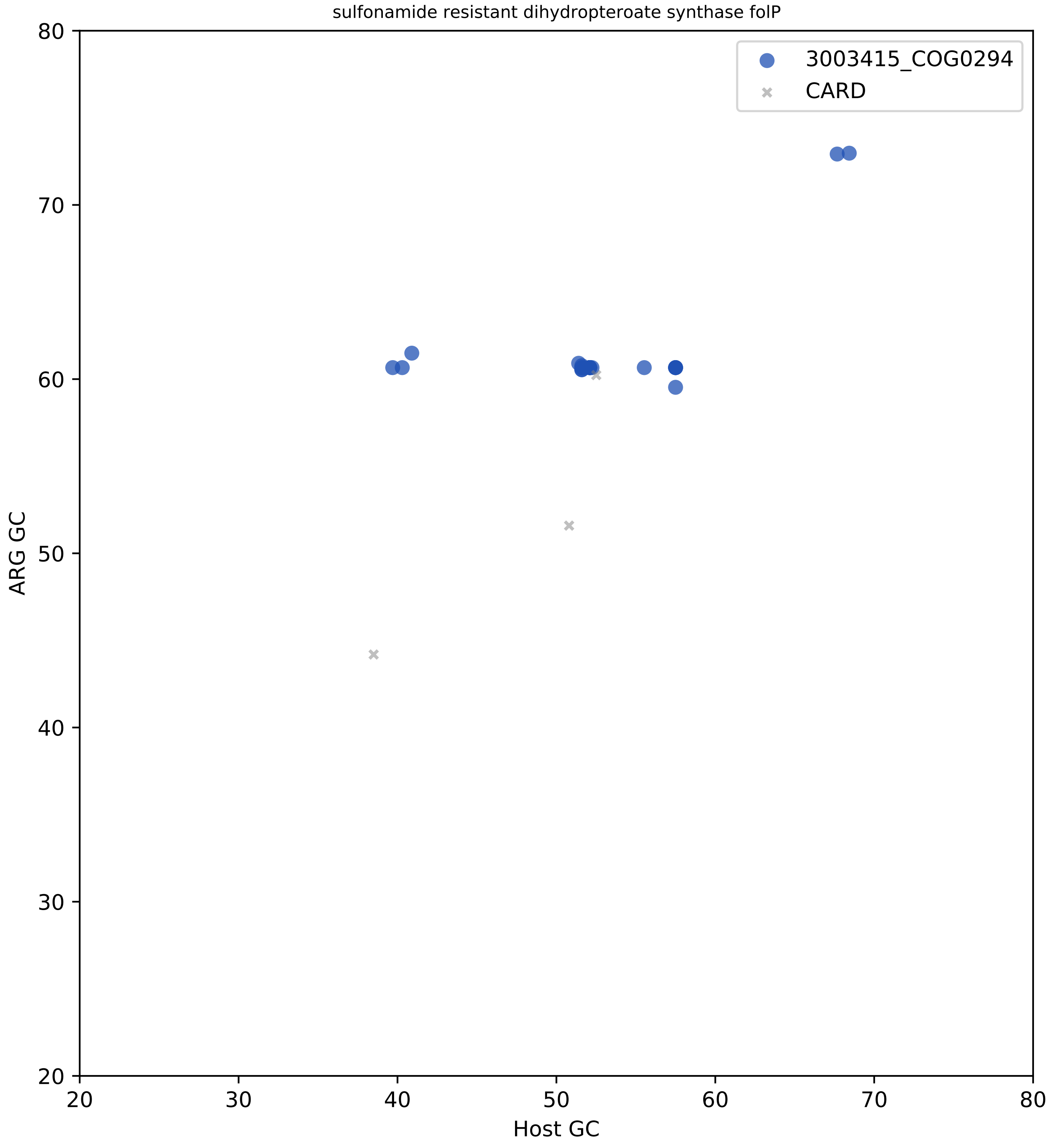

Supplementary Figure S1: (continued).

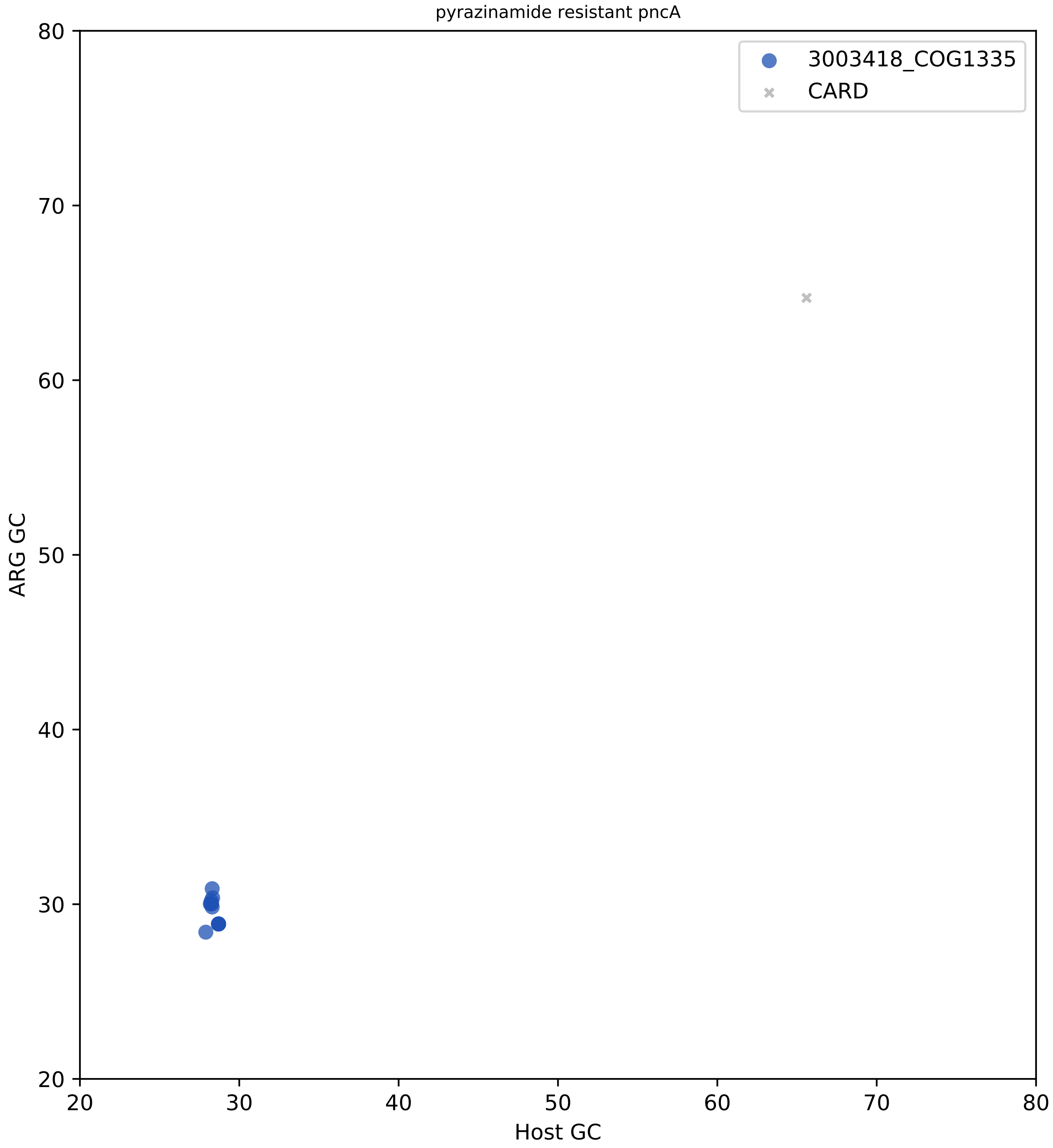

Supplementary Figure S1: (continued).

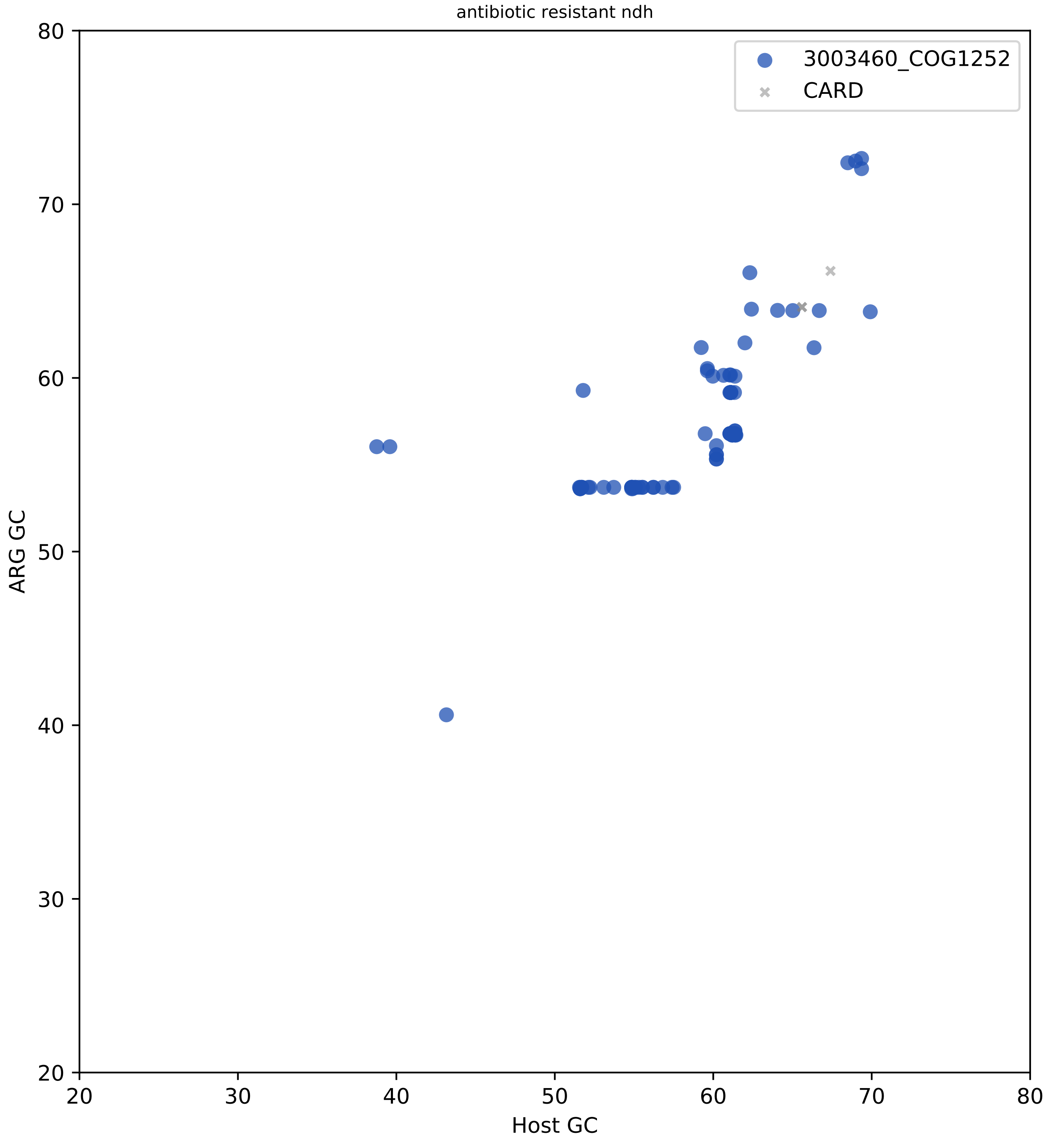

Supplementary Figure S1: (continued).

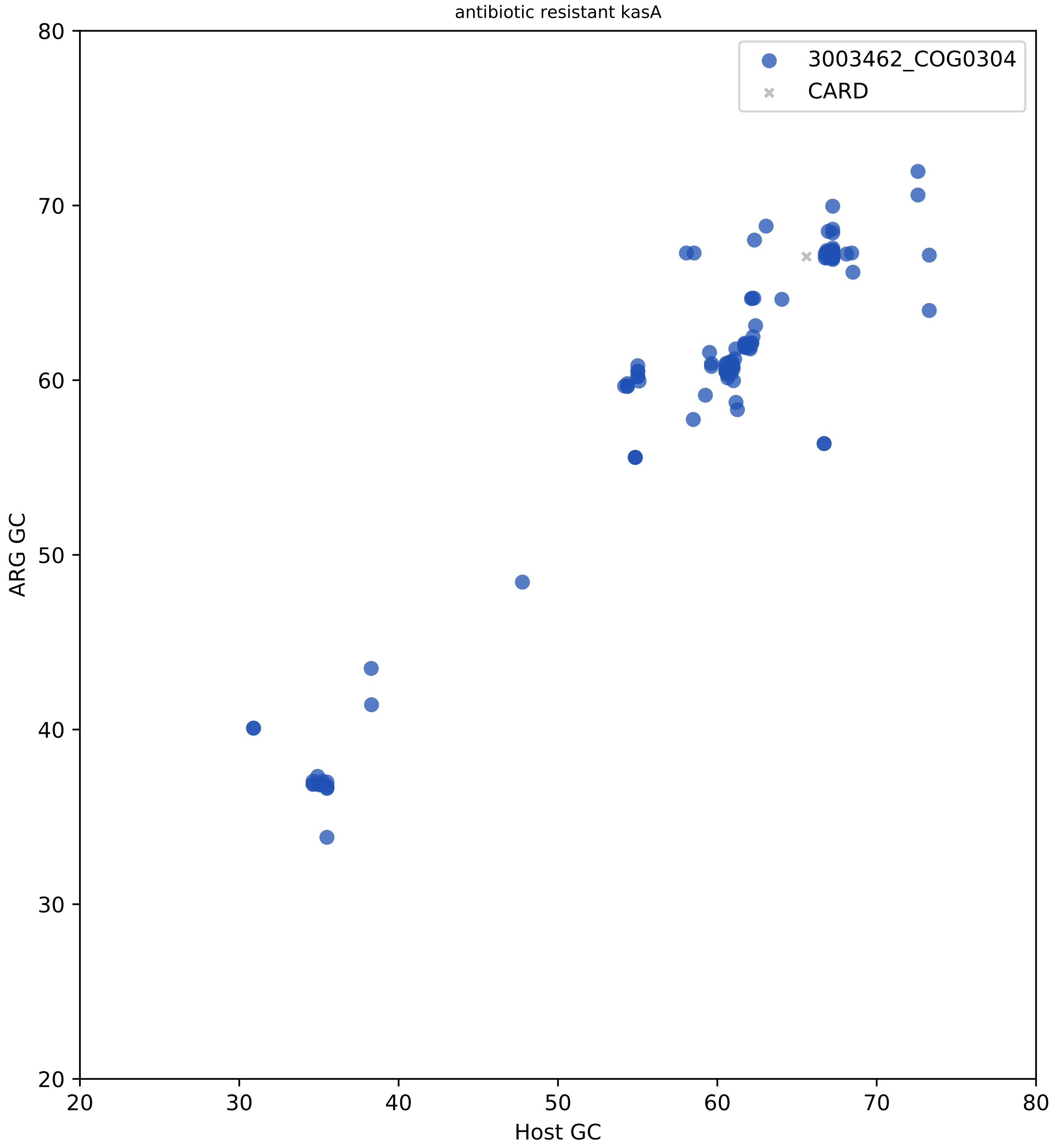

Supplementary Figure S1: (continued).

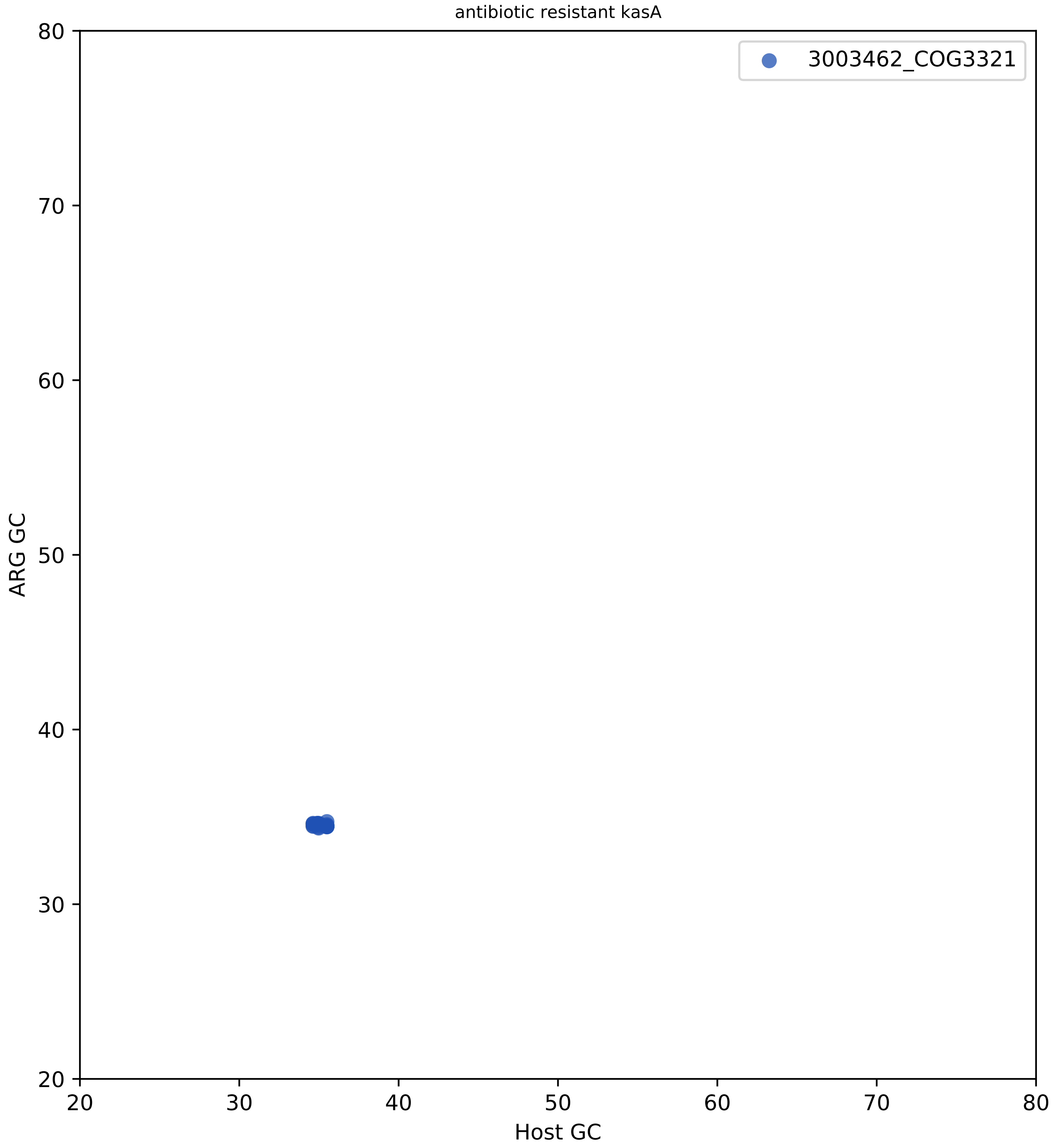

Supplementary Figure S1: (continued).

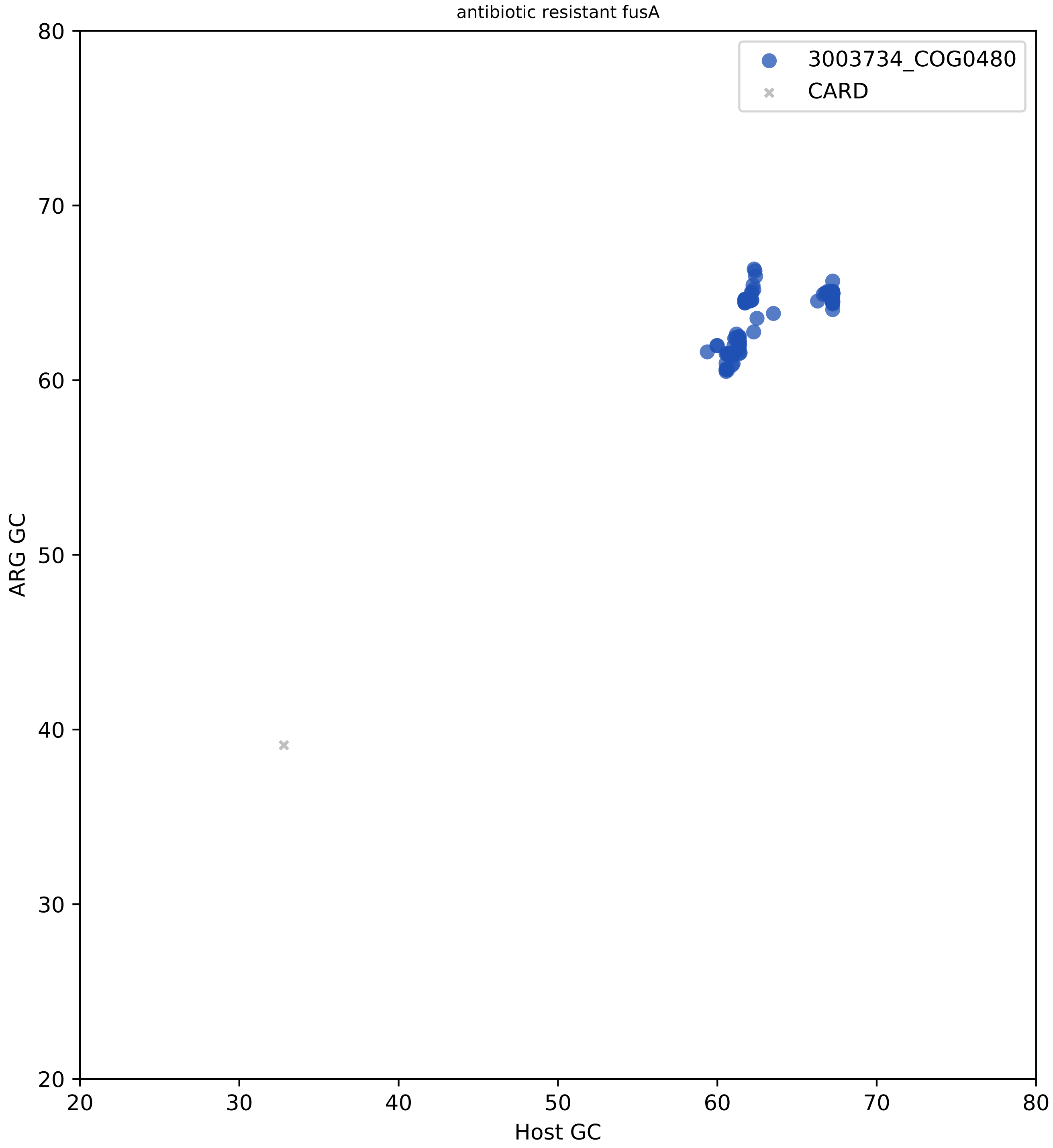

Supplementary Figure S1: (continued).

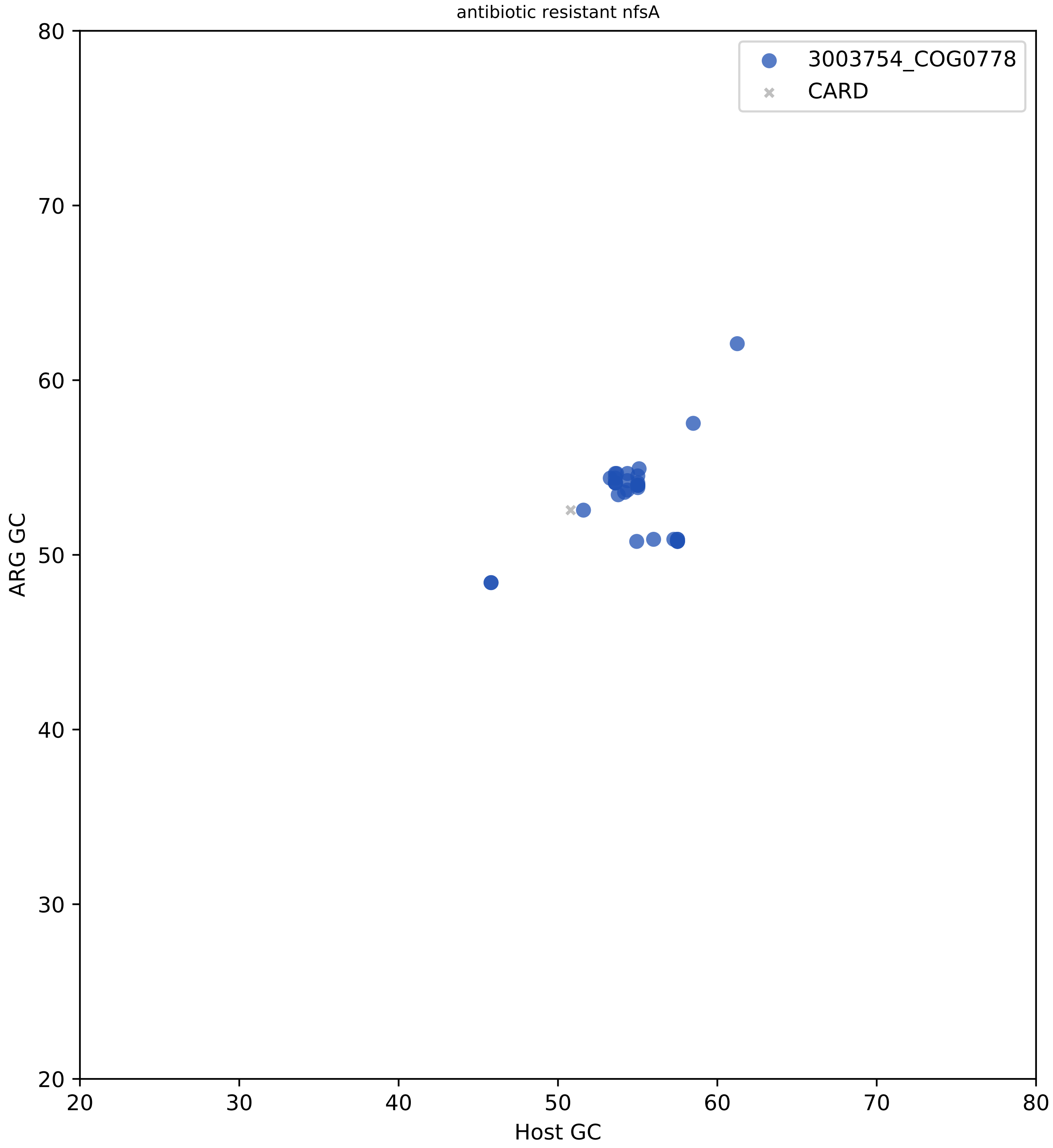

Supplementary Figure S1: (continued).

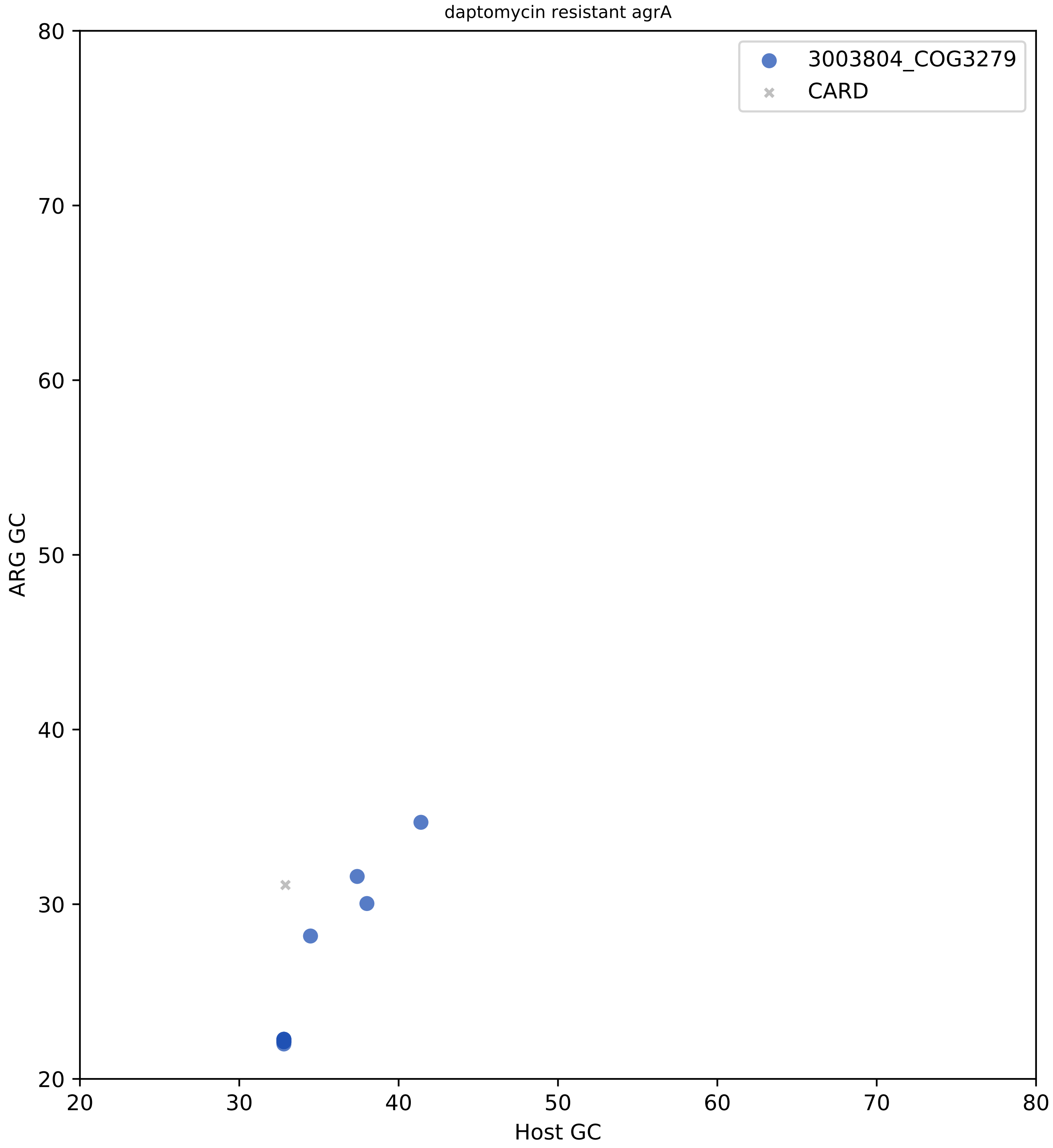

Supplementary Figure S1: (continued).

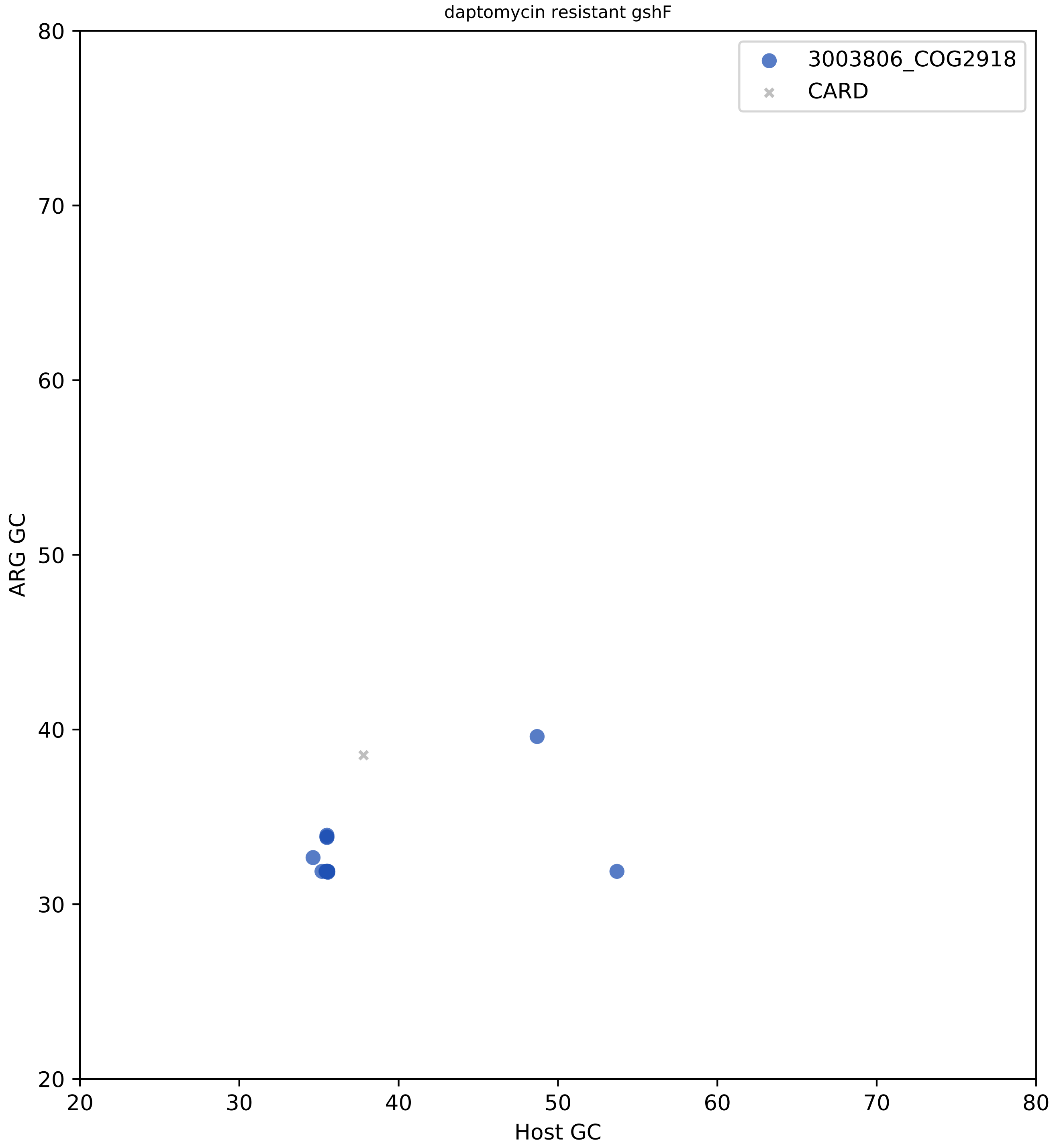

Supplementary Figure S1: (continued).

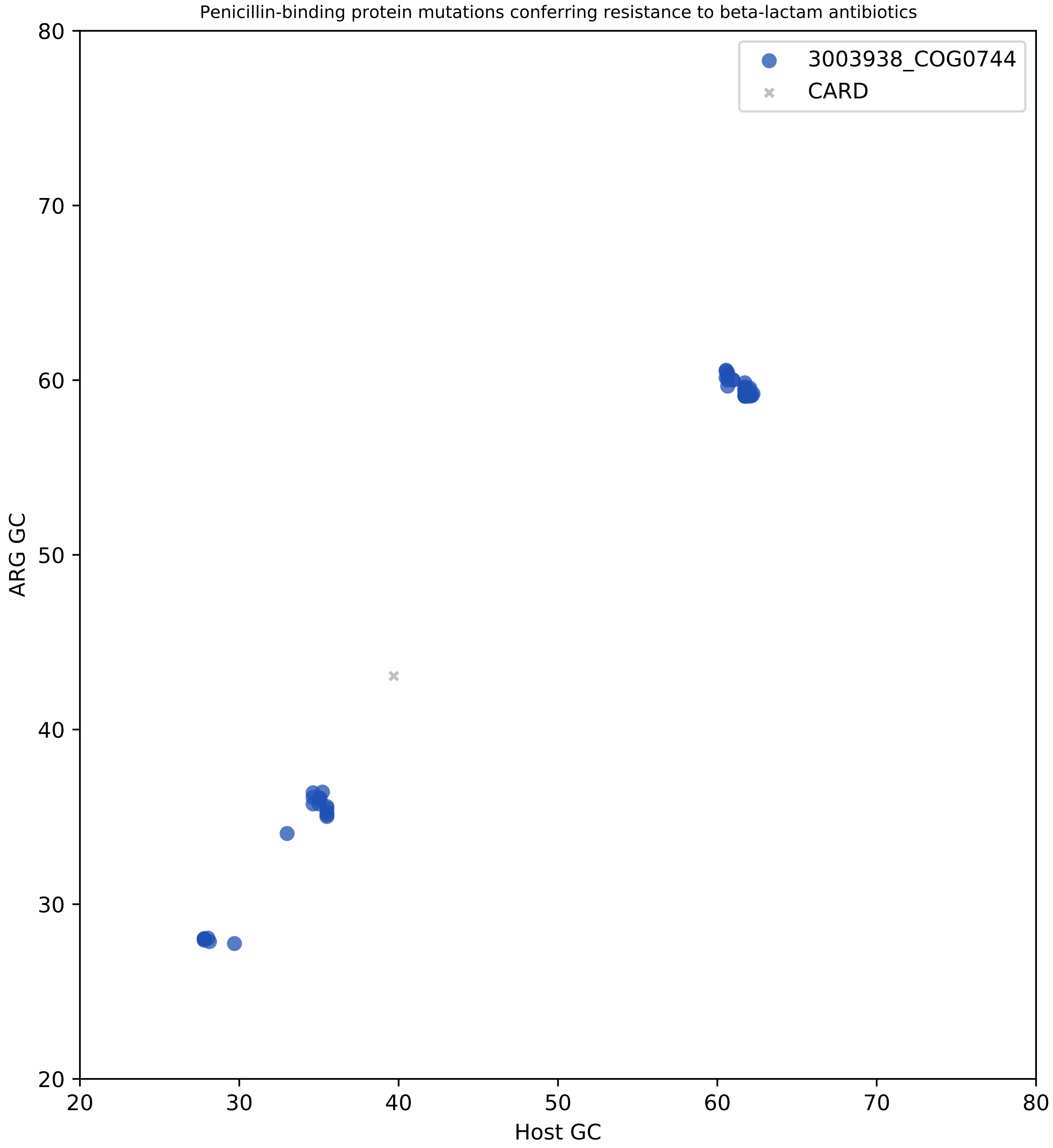

Supplementary Figure S1: (continued).

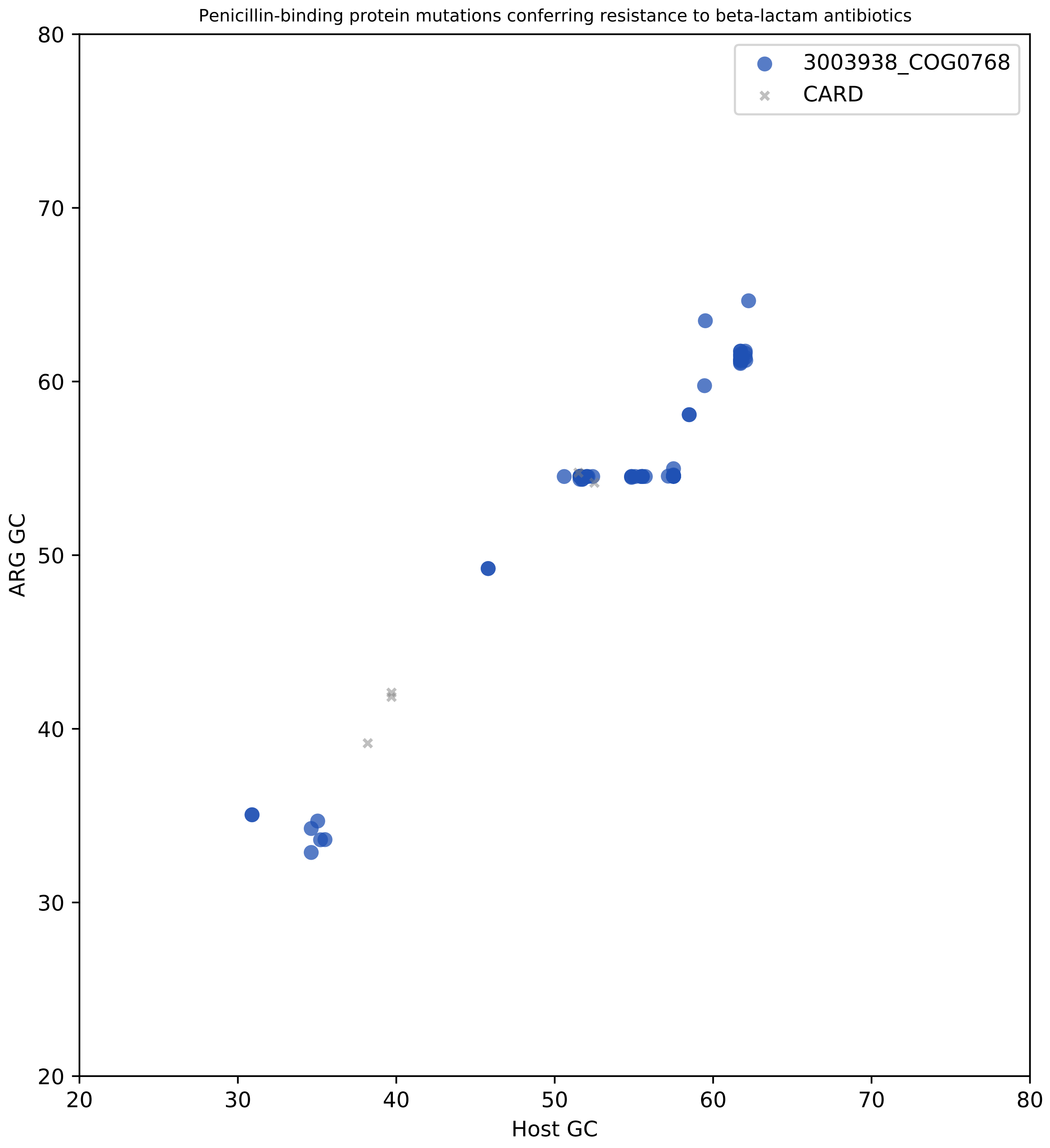

Supplementary Figure S1: (continued).

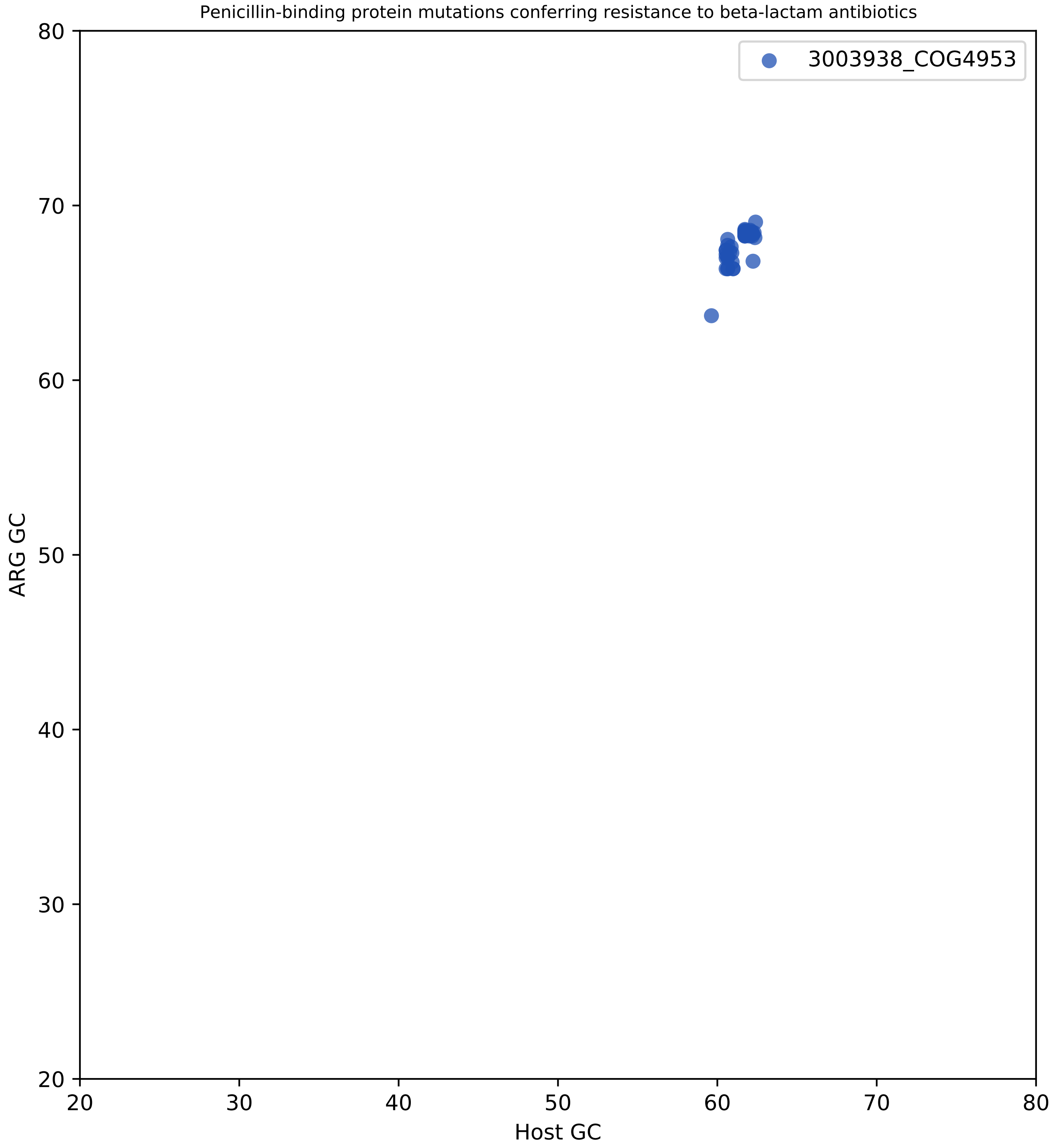

Supplementary Figure S1: (continued).

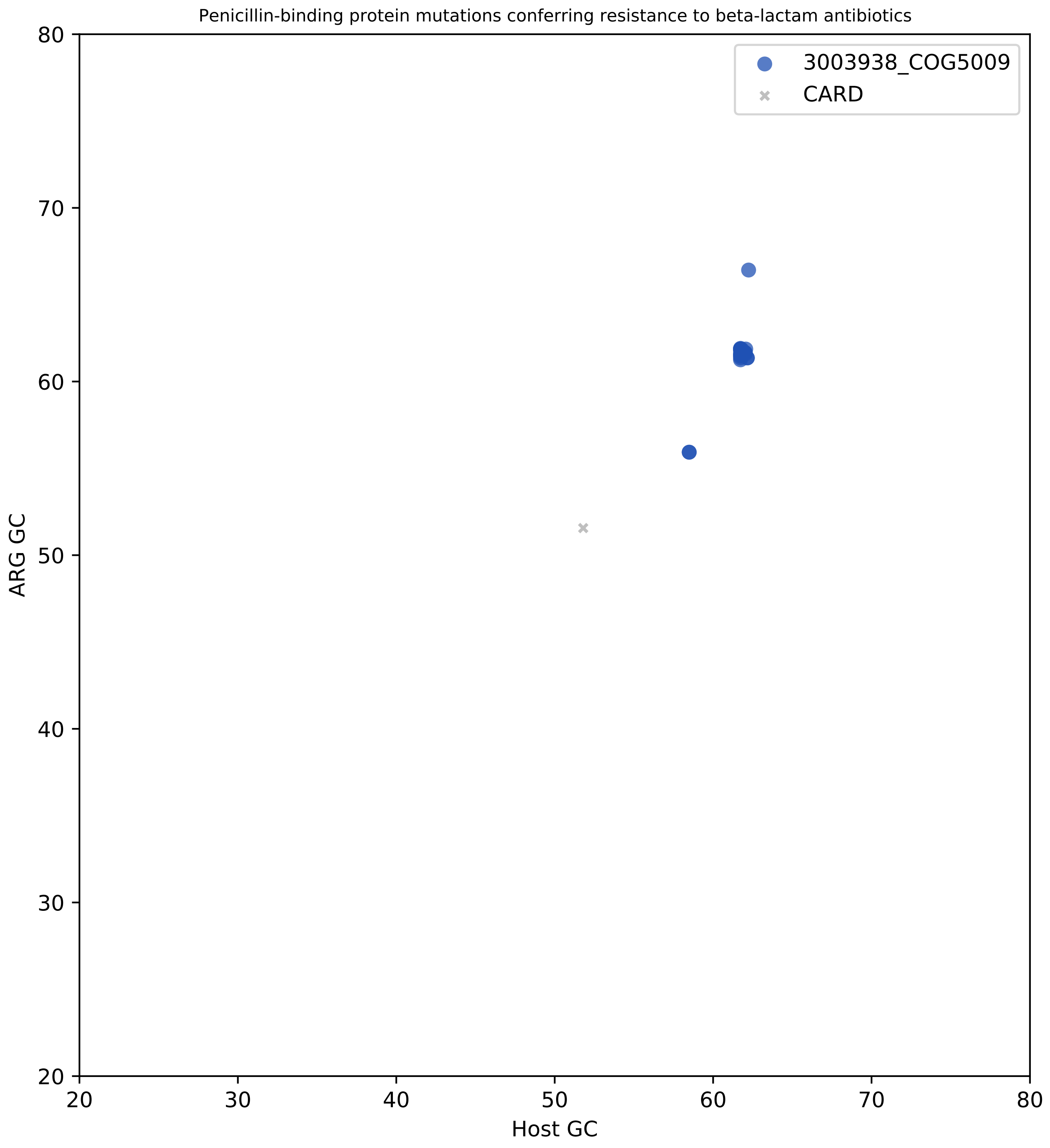

Supplementary Figure S1: (continued).

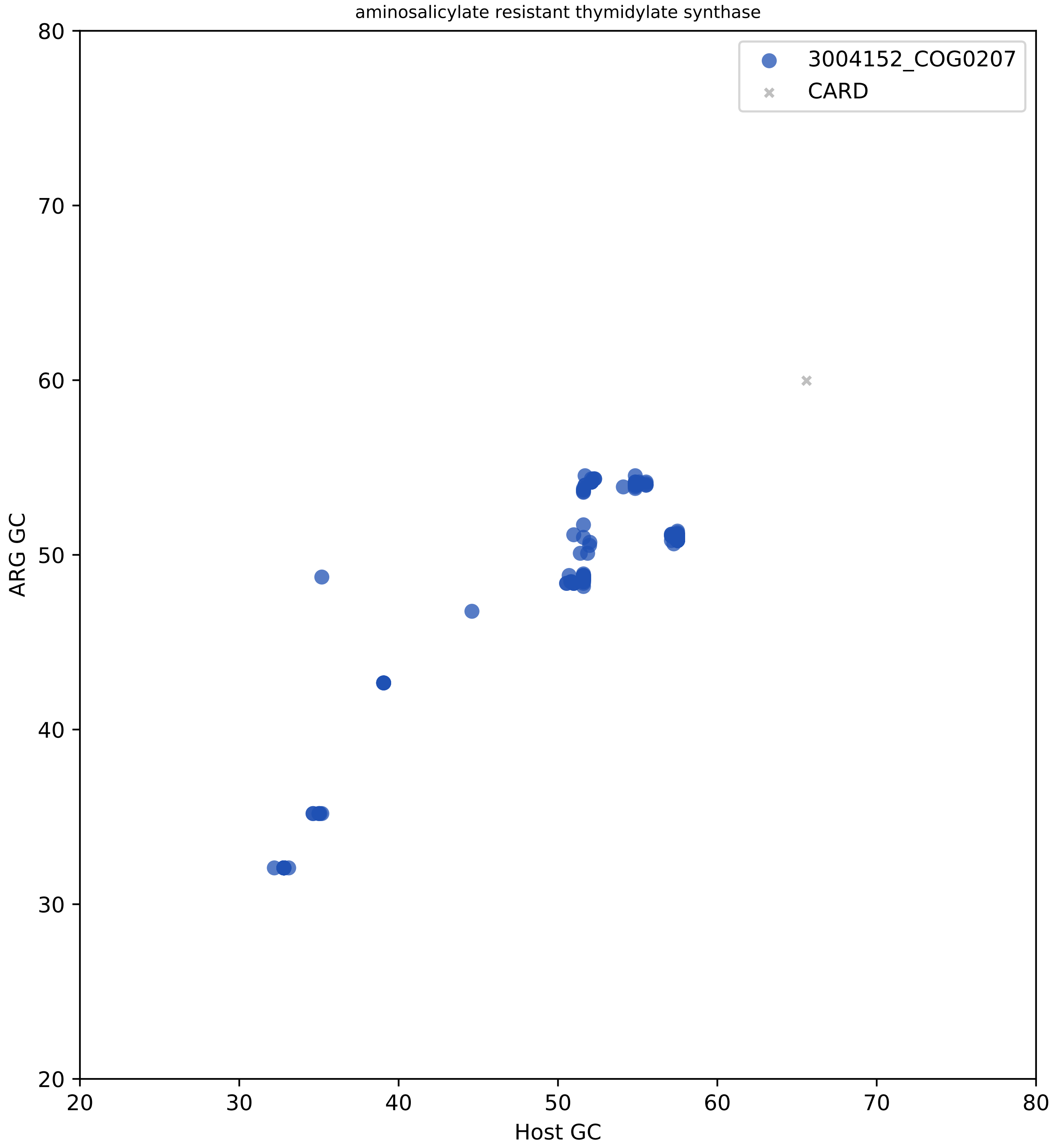

Supplementary Figure S1: (continued).

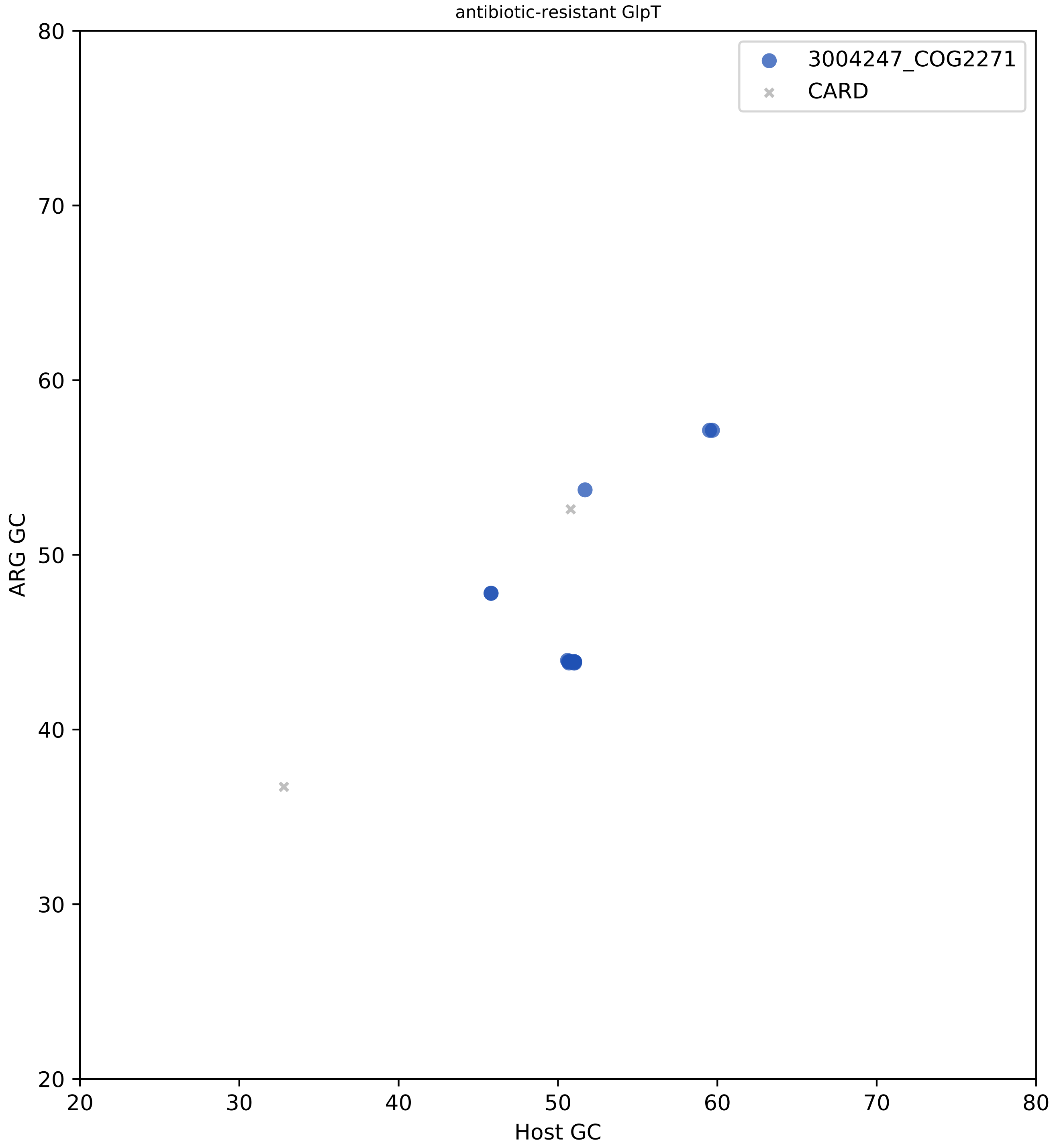

Supplementary Figure S1: (continued).

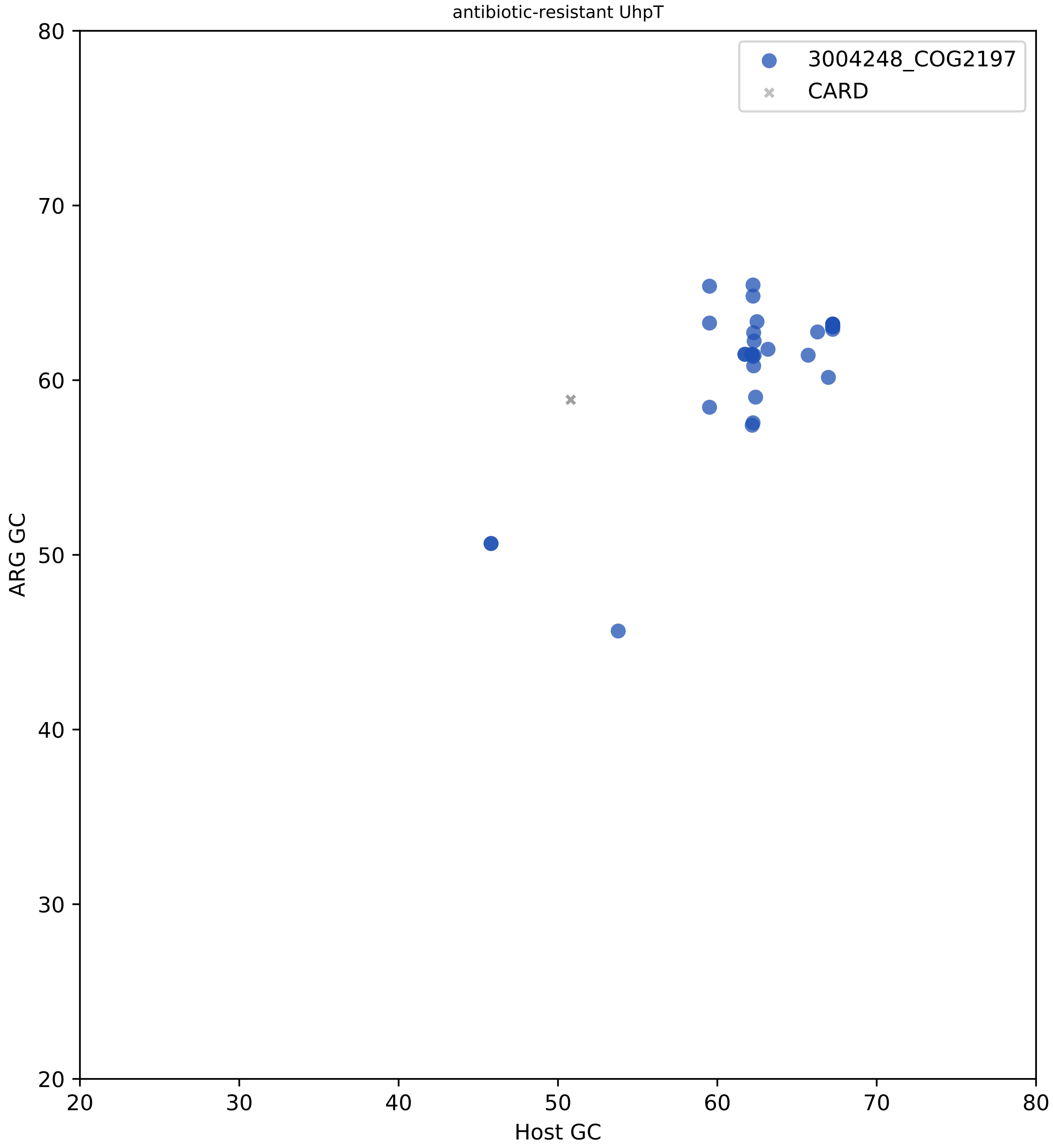

Supplementary Figure S1: (continued).

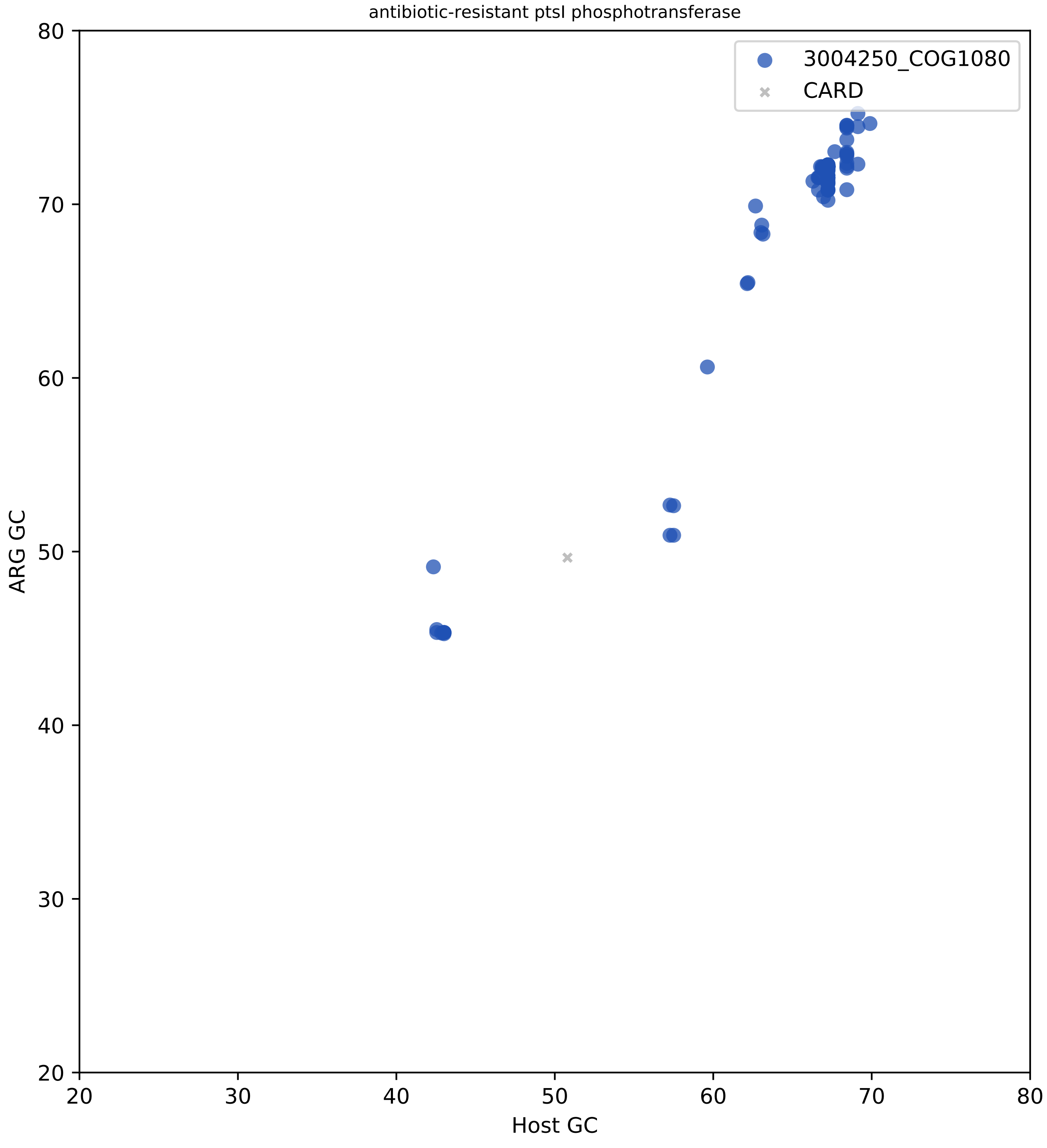

Supplementary Figure S1: (continued).

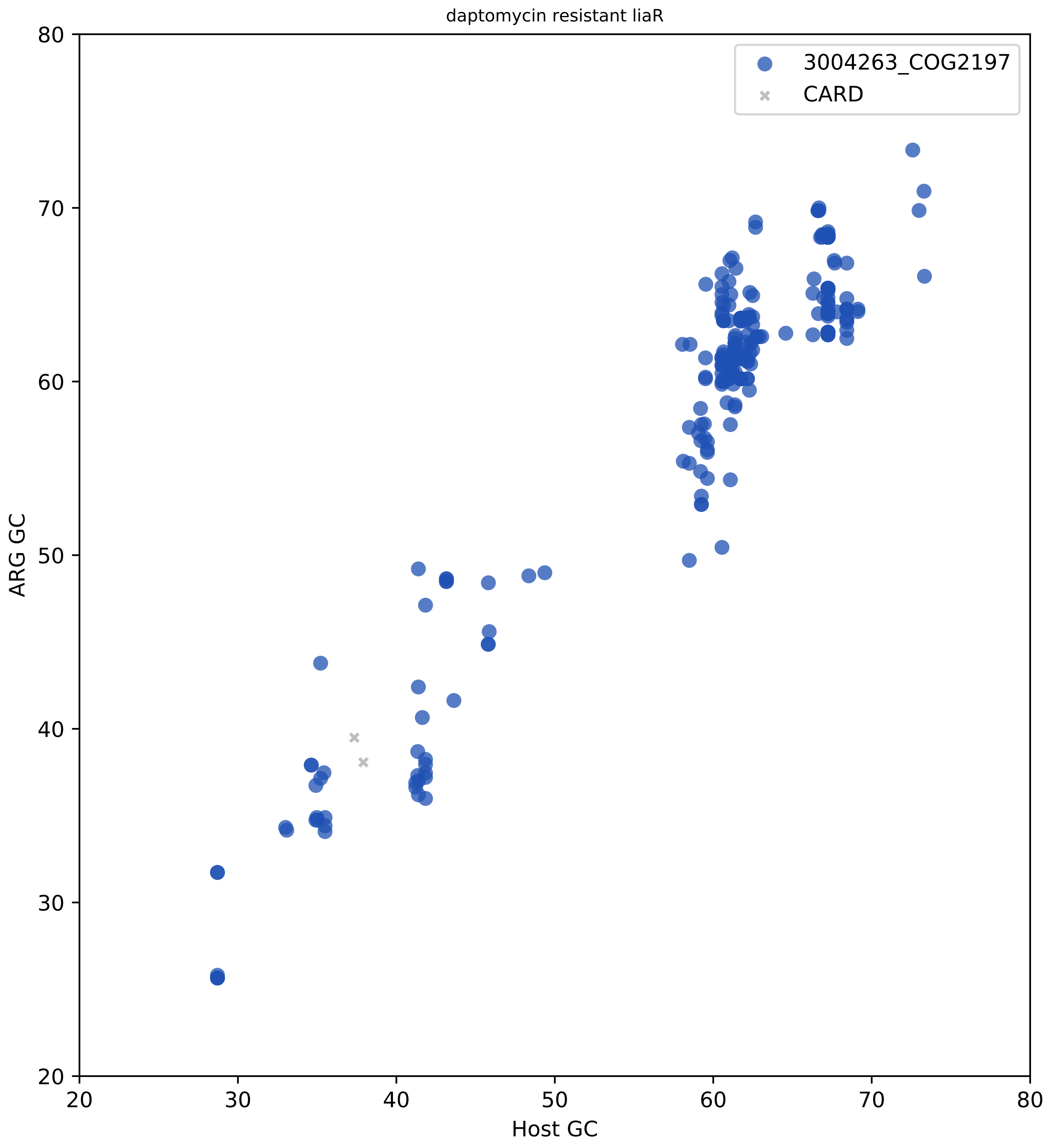

Supplementary Figure S1: (continued).

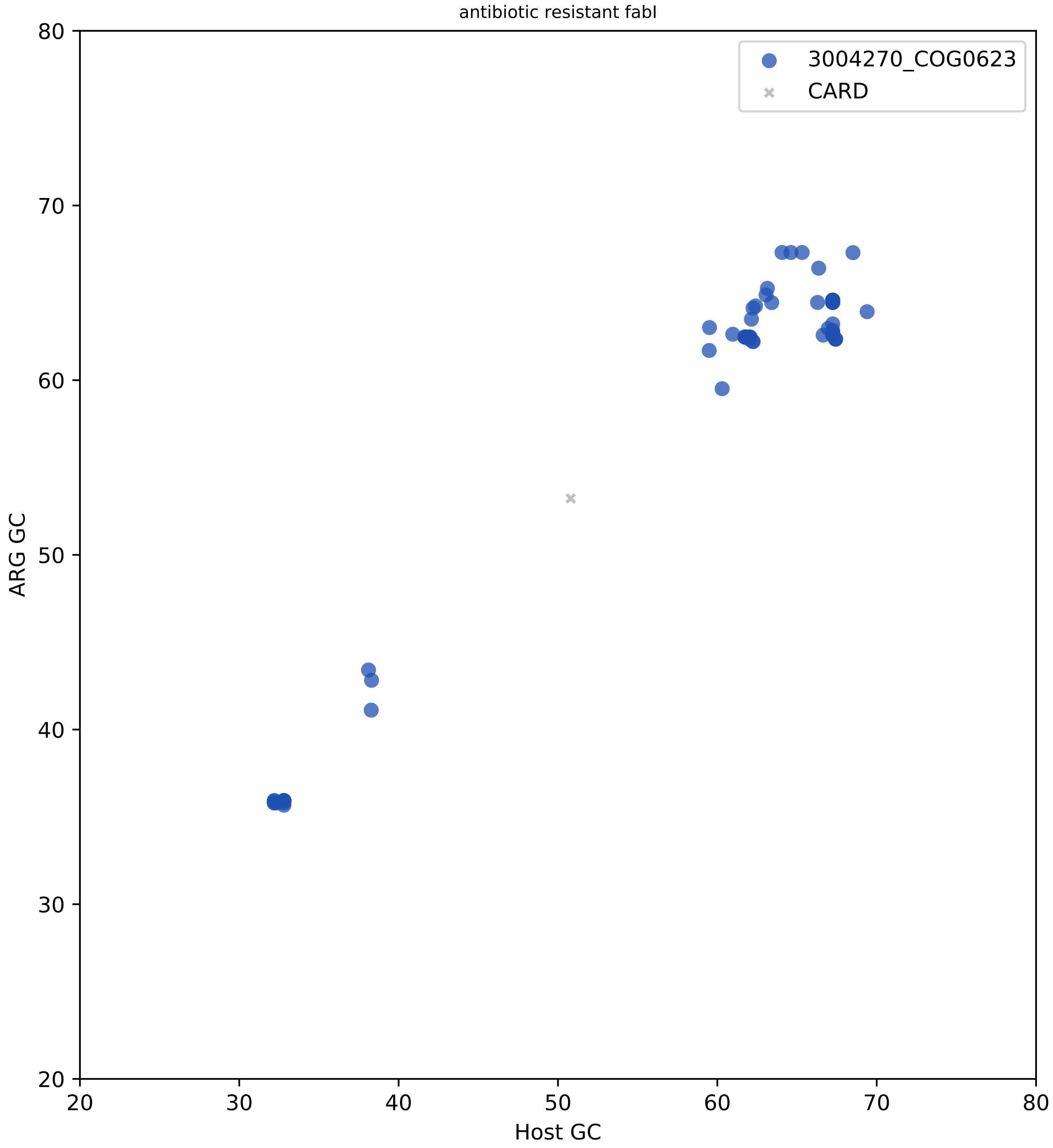

Supplementary Figure S1: (continued).

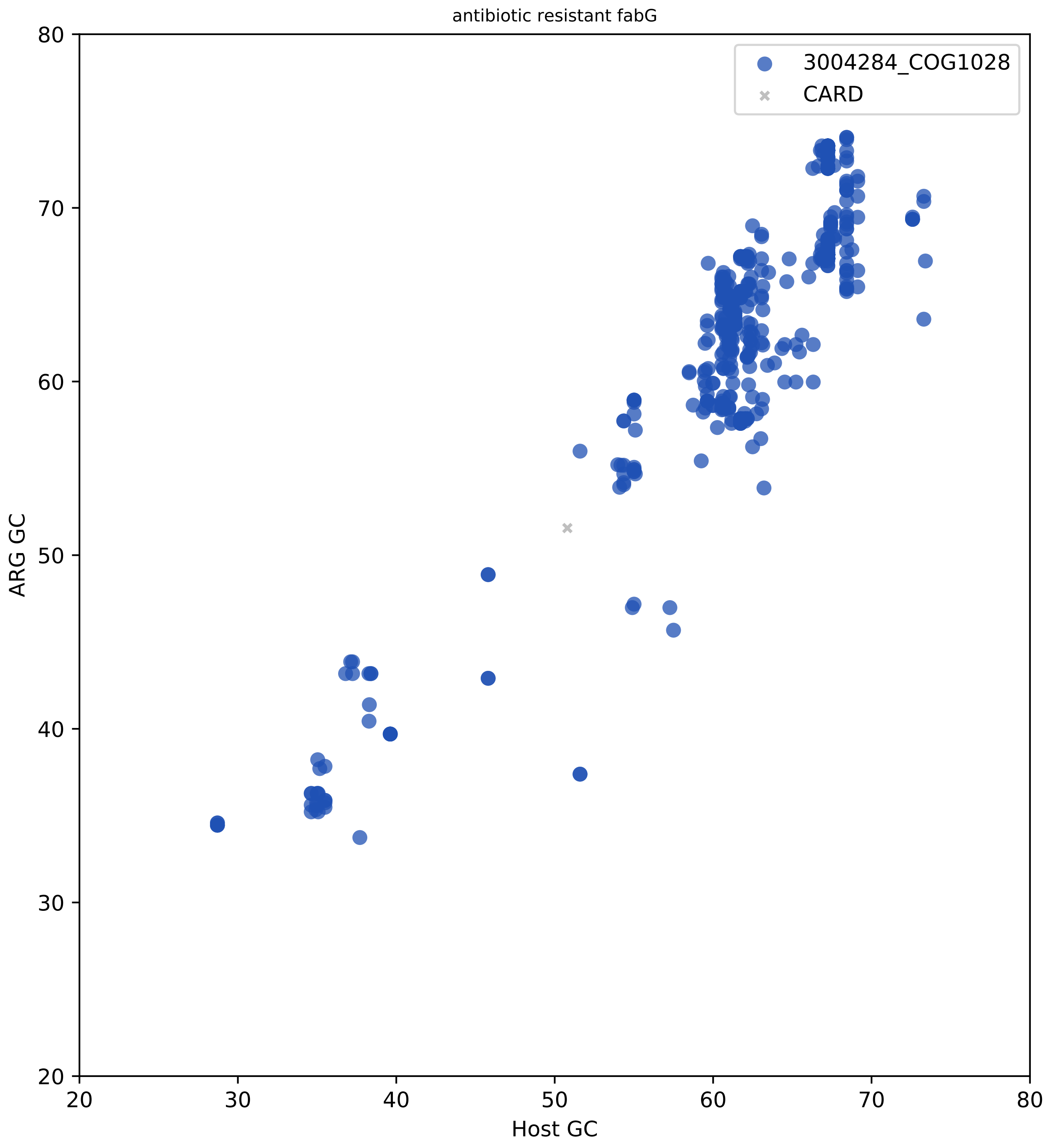

Supplementary Figure S1: (continued).

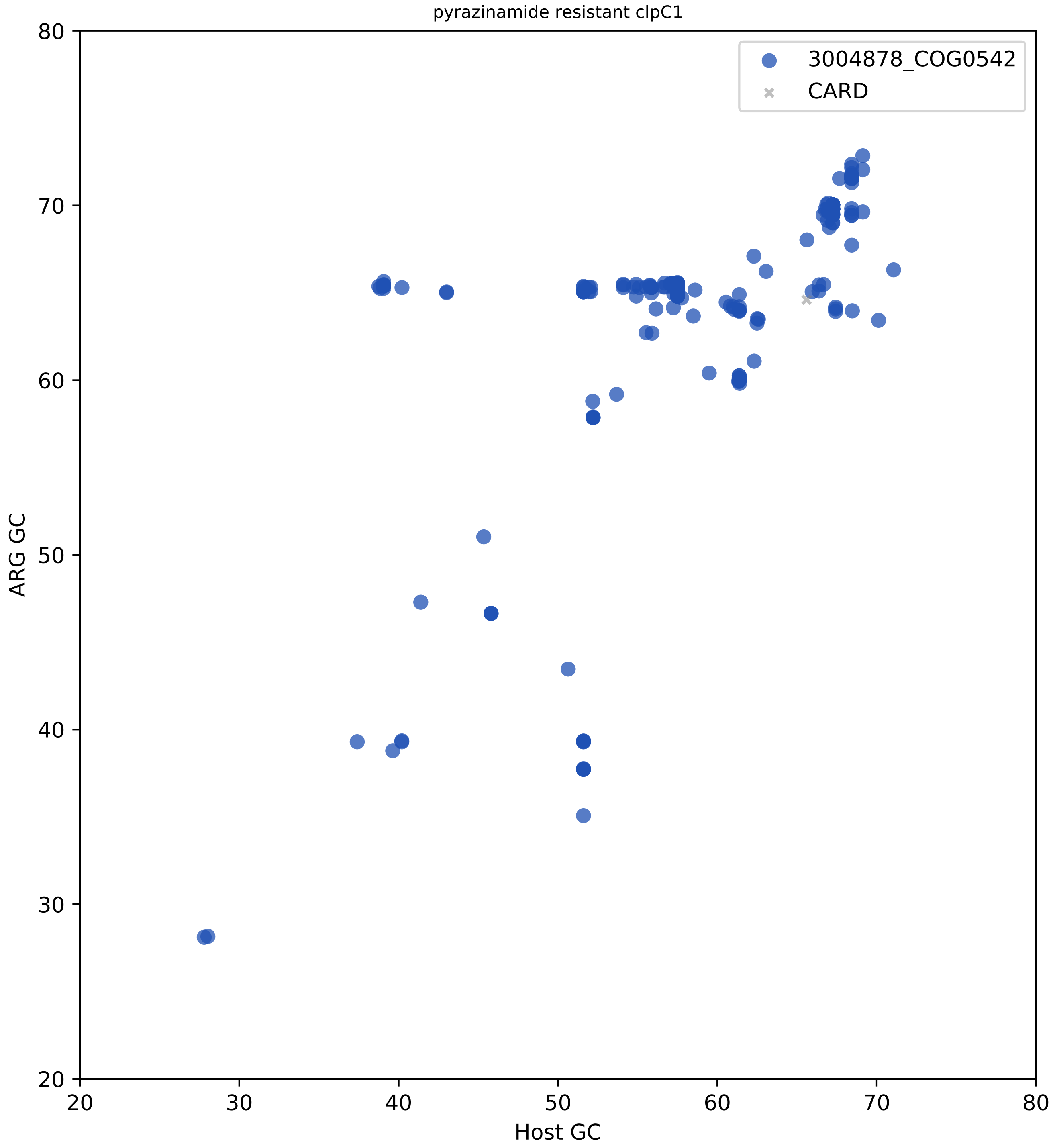

Supplementary Figure S1: (continued).

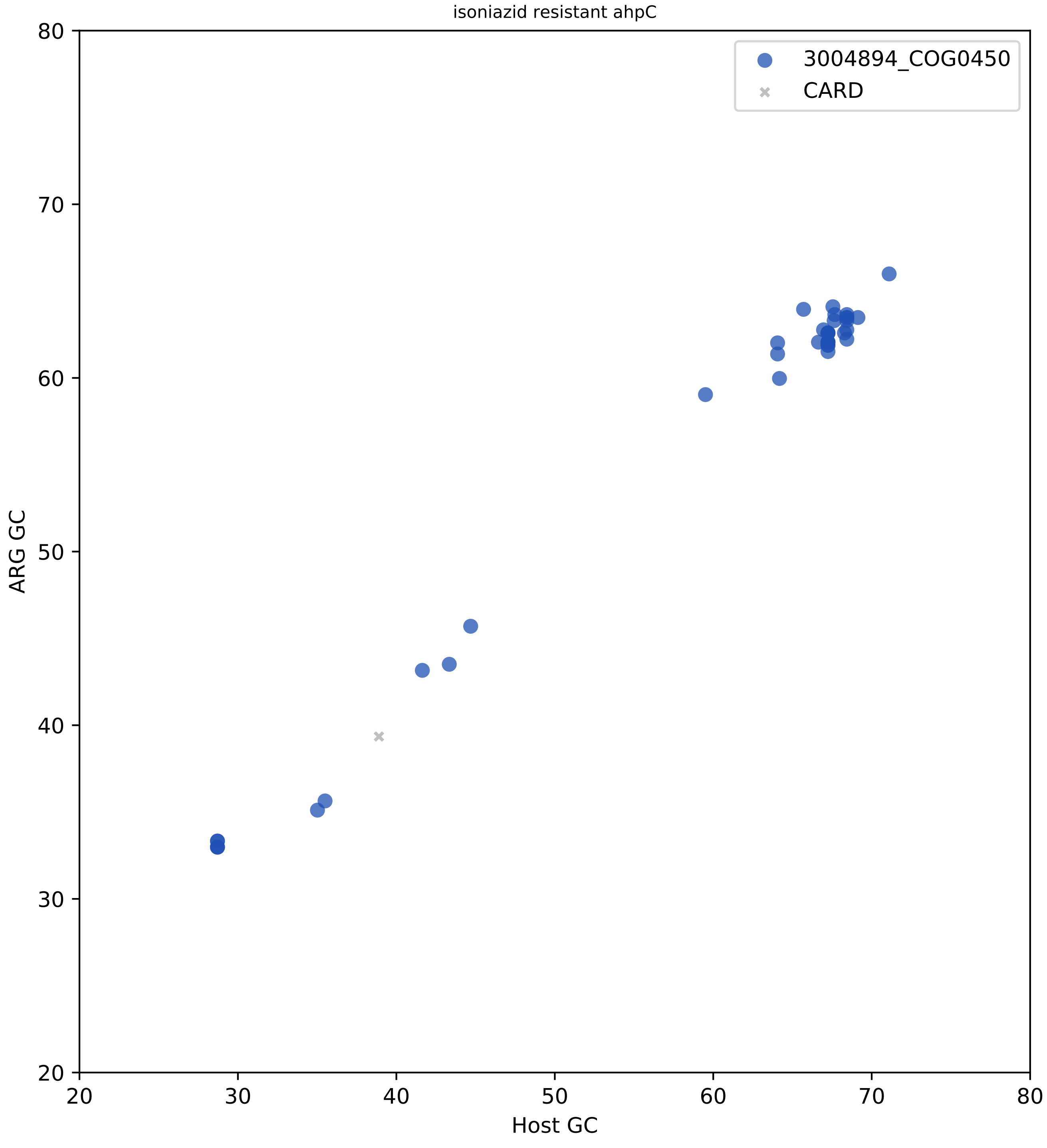

Supplementary Figure S1: (continued).

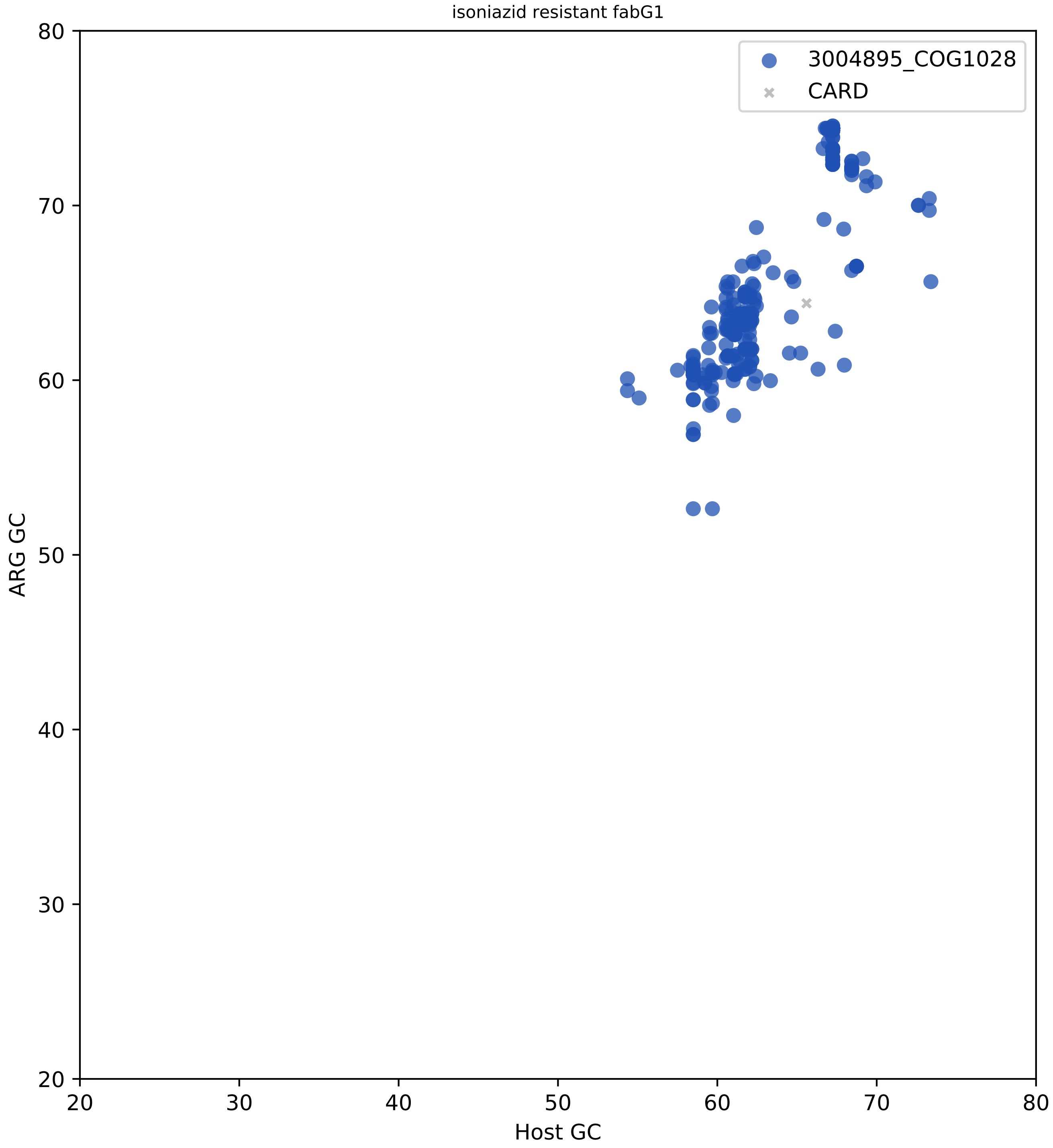

Supplementary Figure S1: (continued).

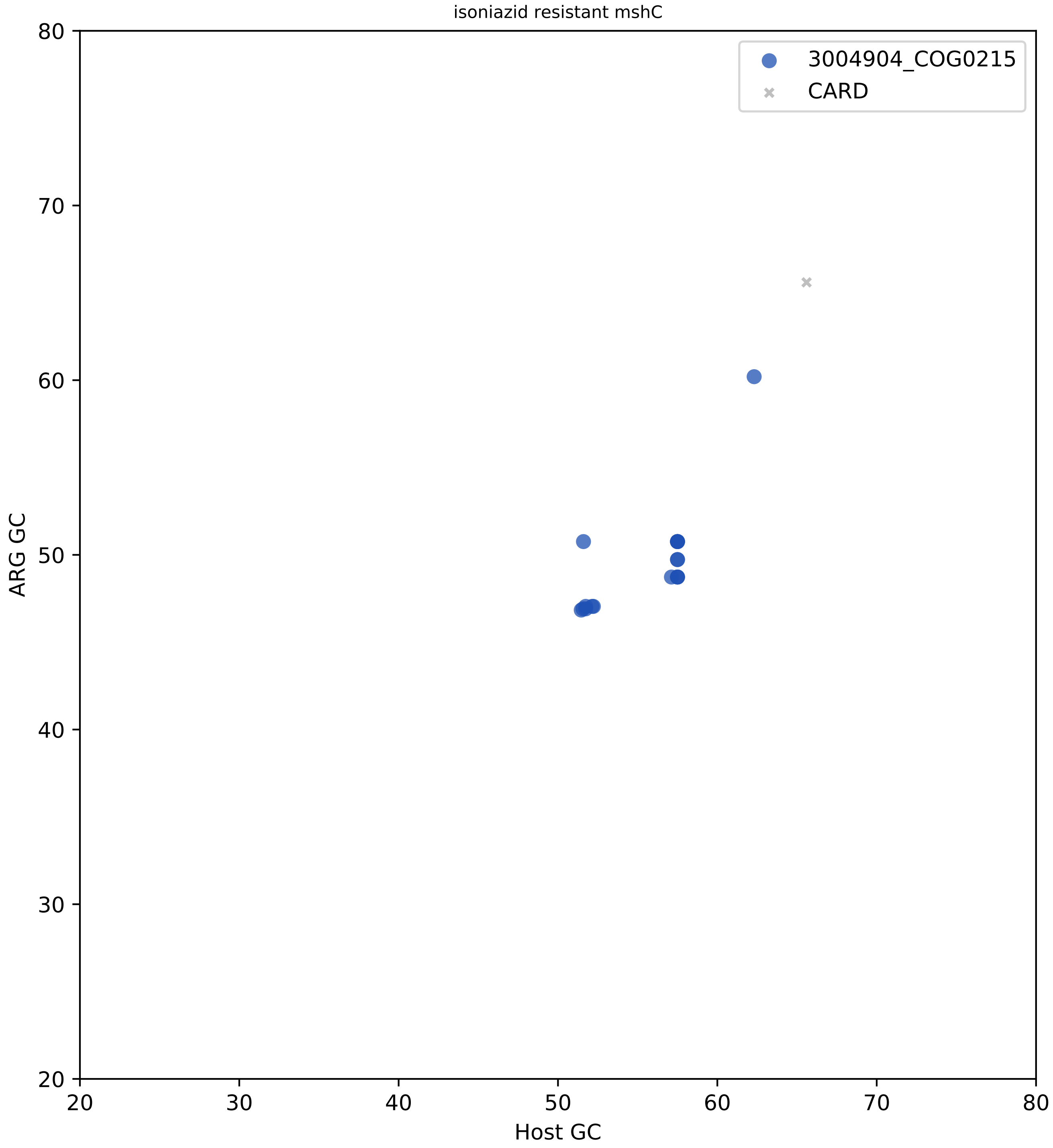

Supplementary Figure S1: (continued).

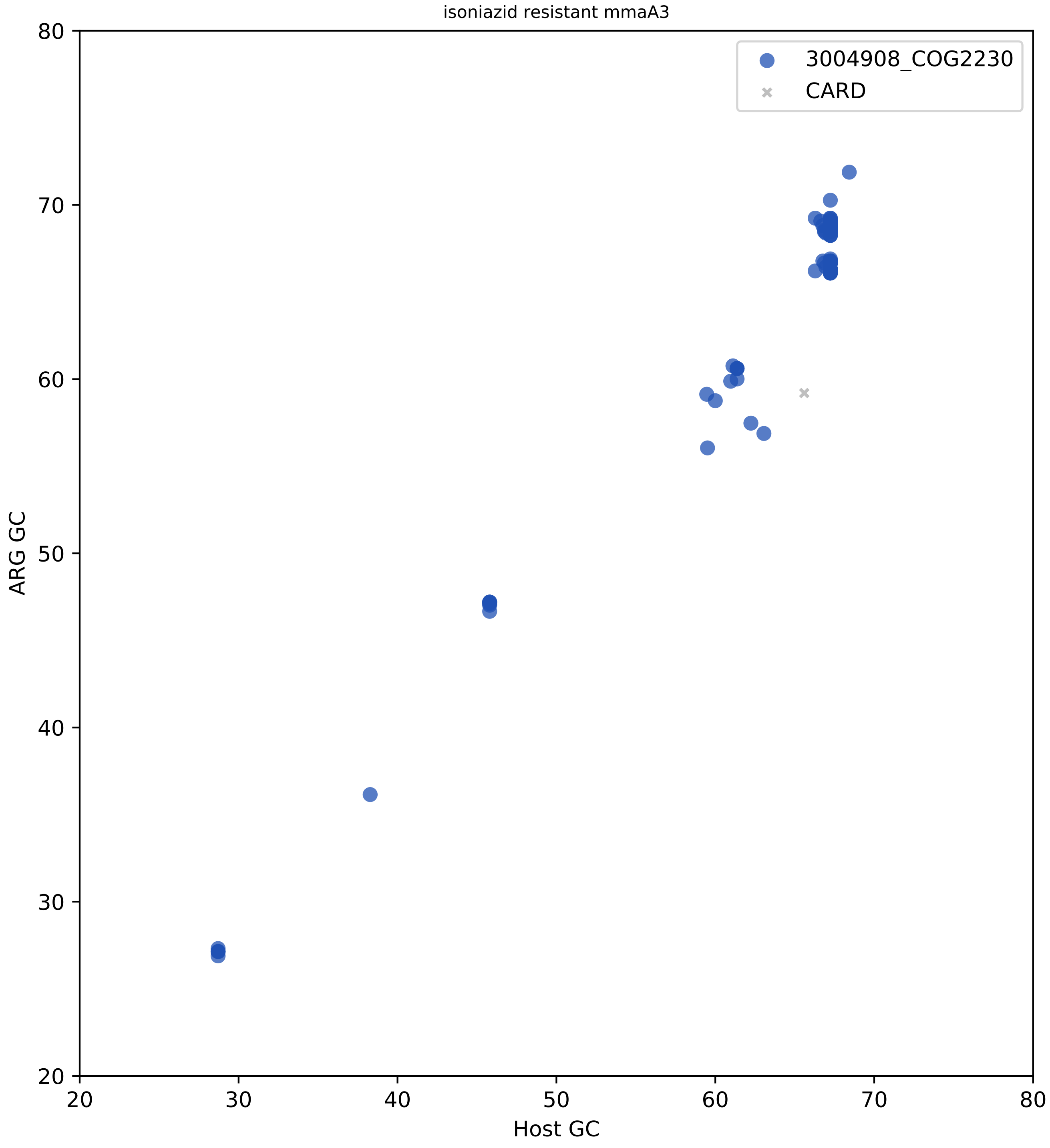

Supplementary Figure S1: (continued).

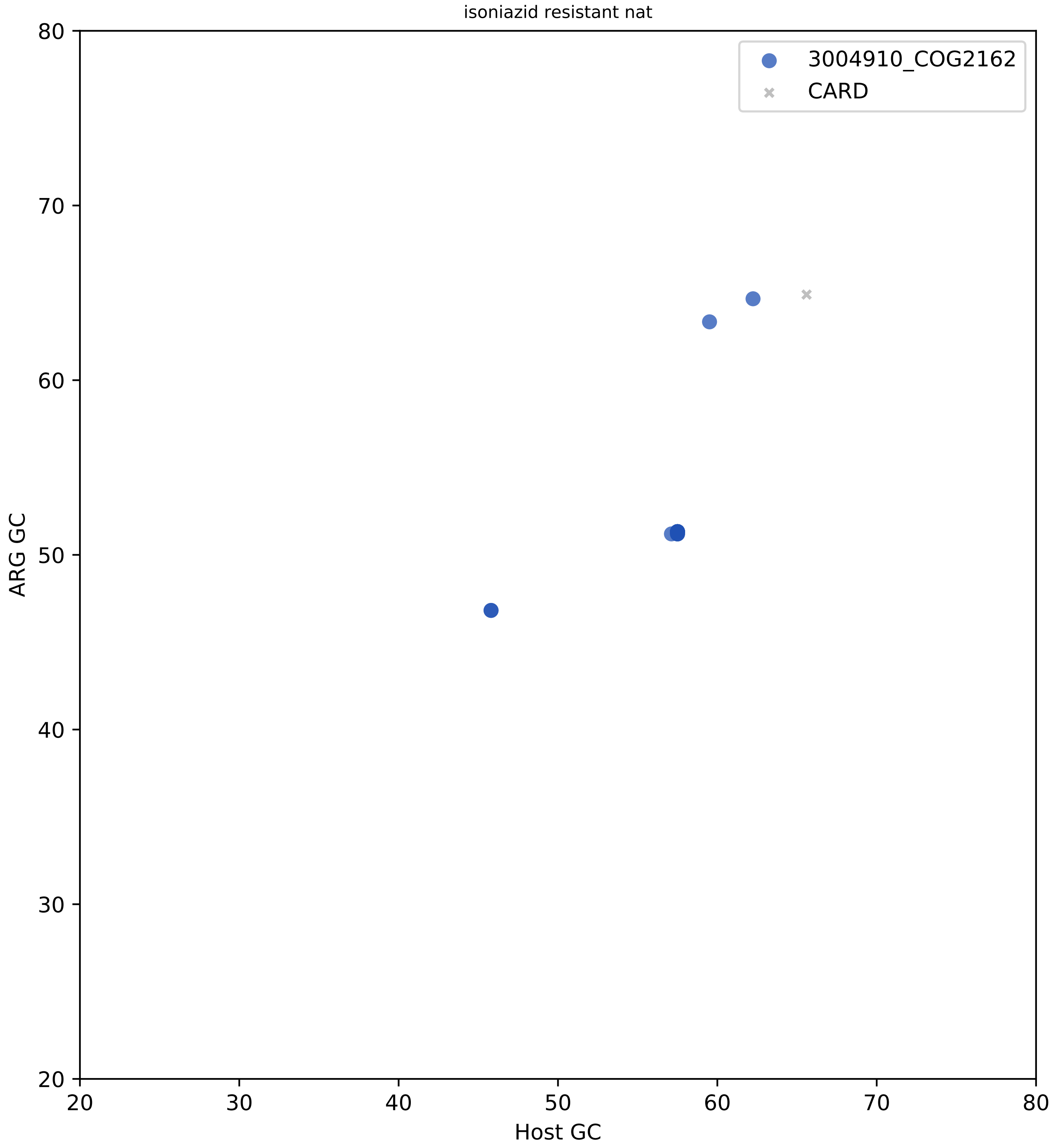

Supplementary Figure S1: (continued).

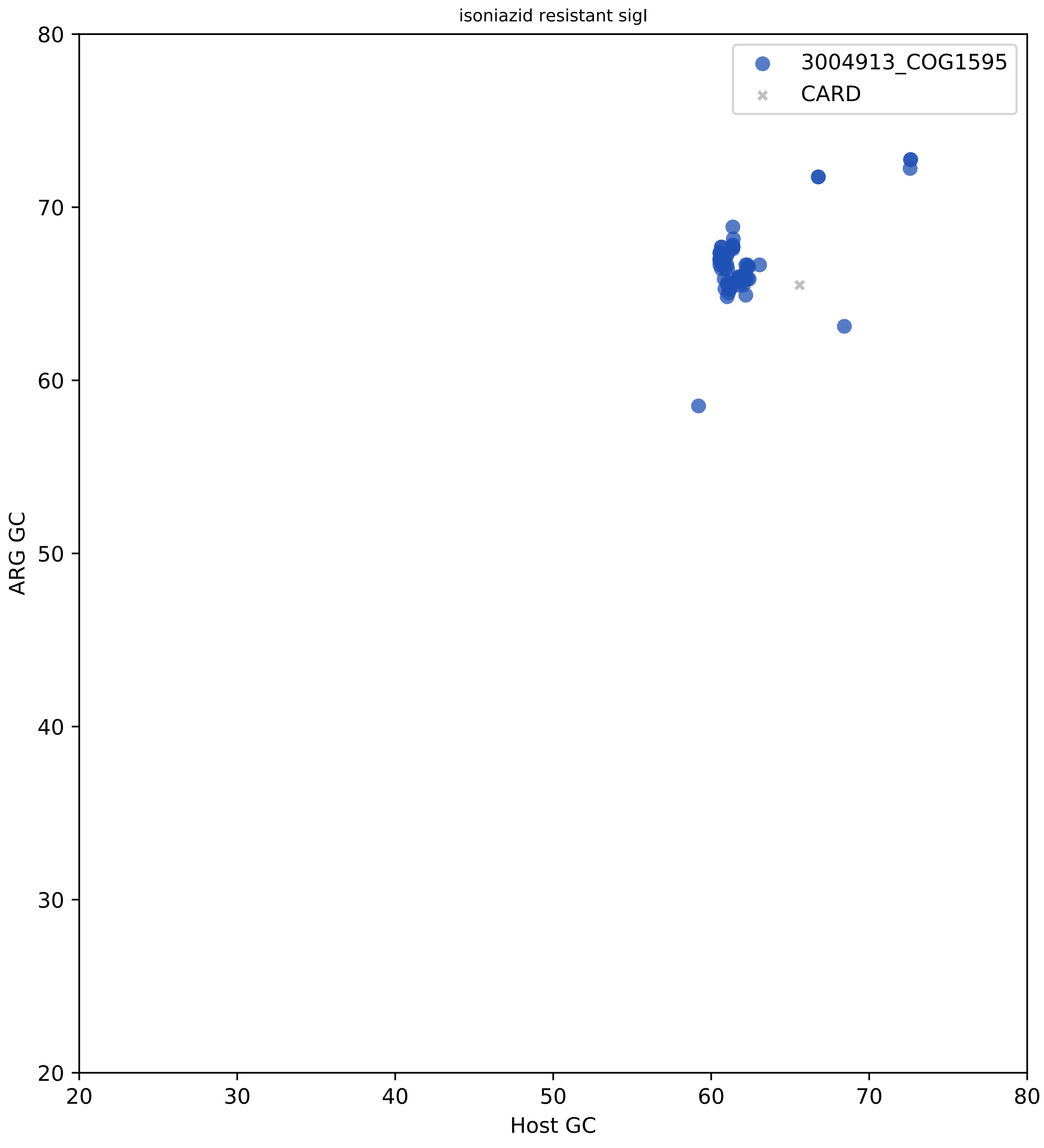

Supplementary Figure S1: (continued).

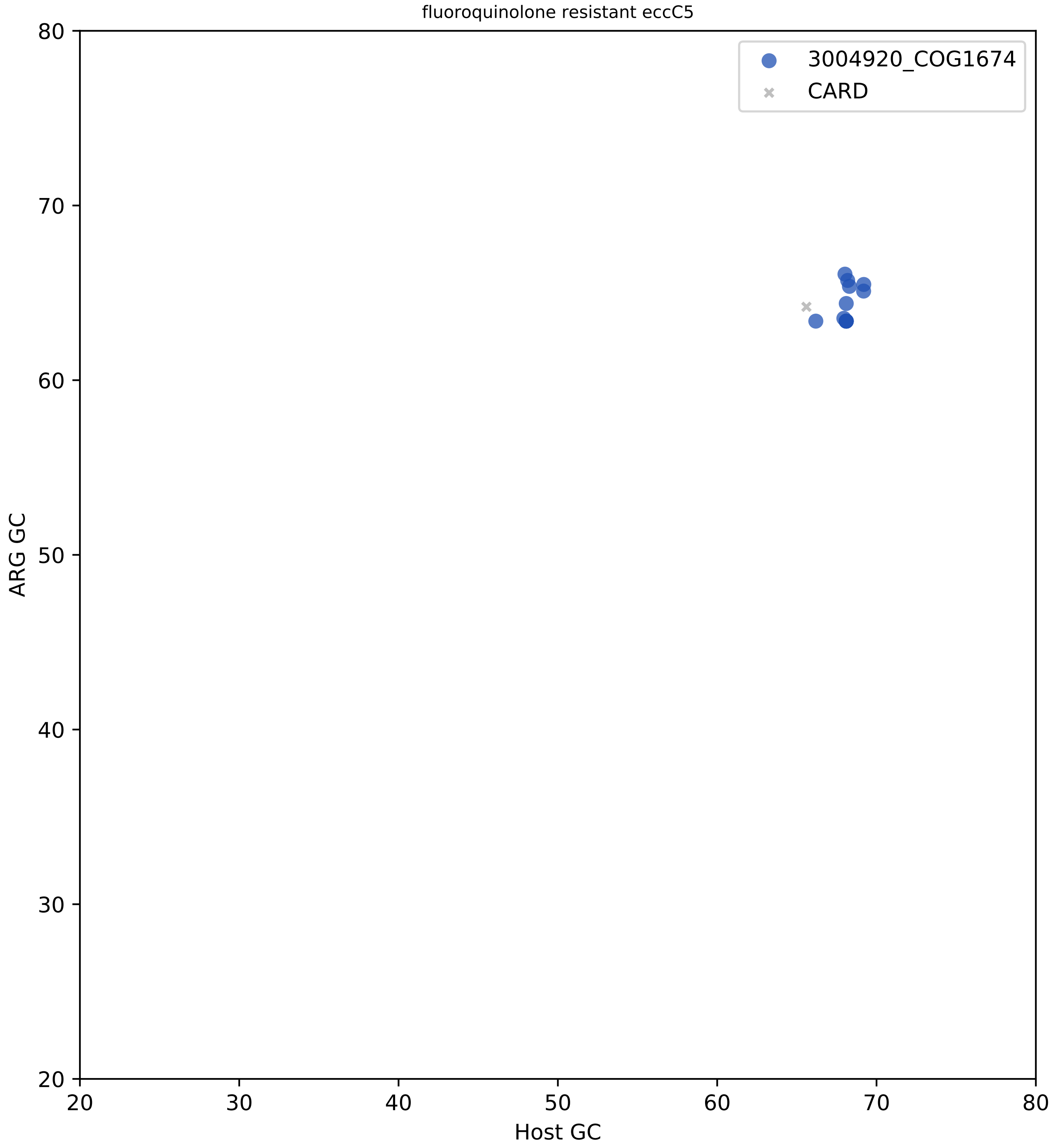

Supplementary Figure S1: (continued).

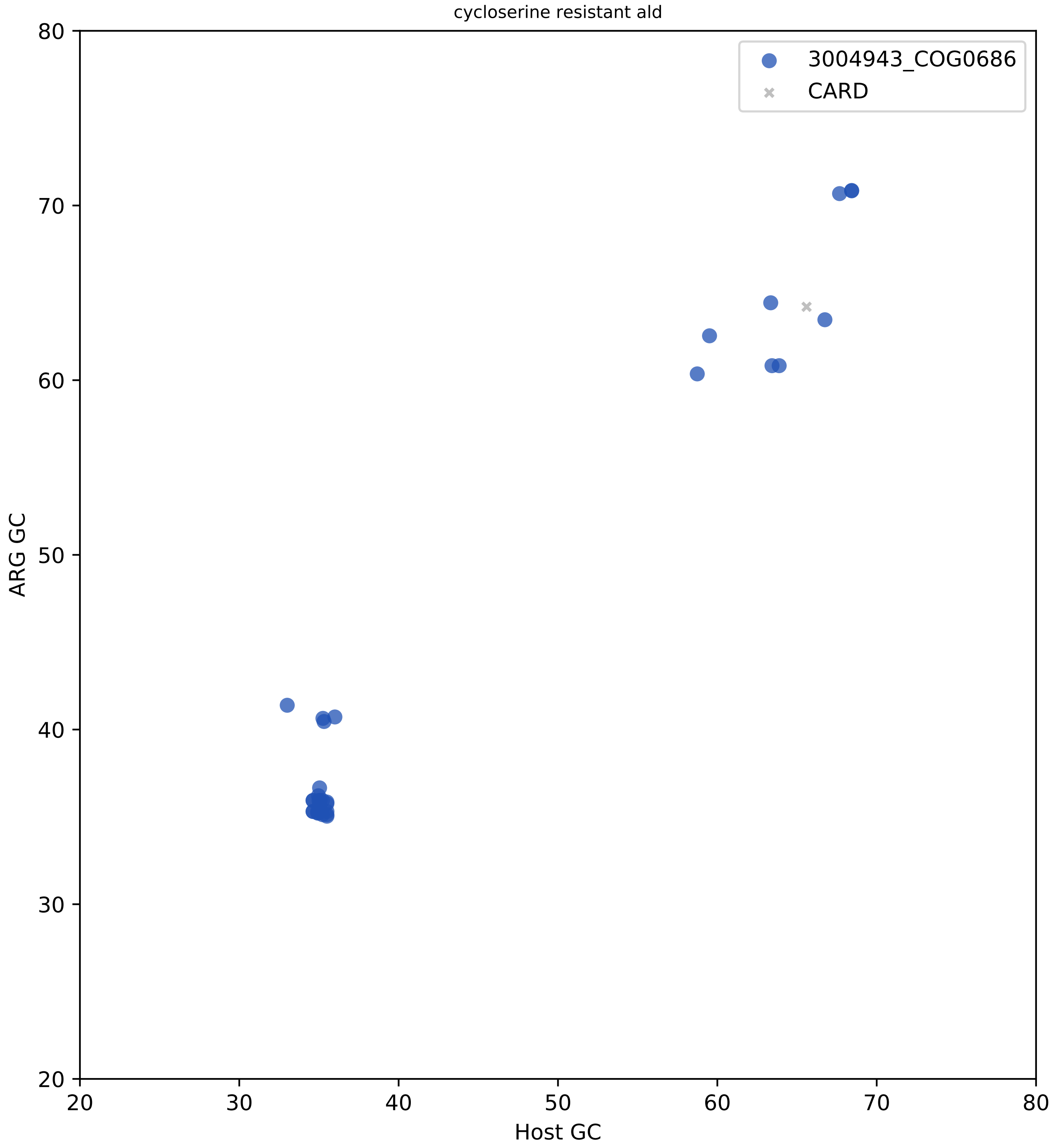

Supplementary Figure S1: (continued).

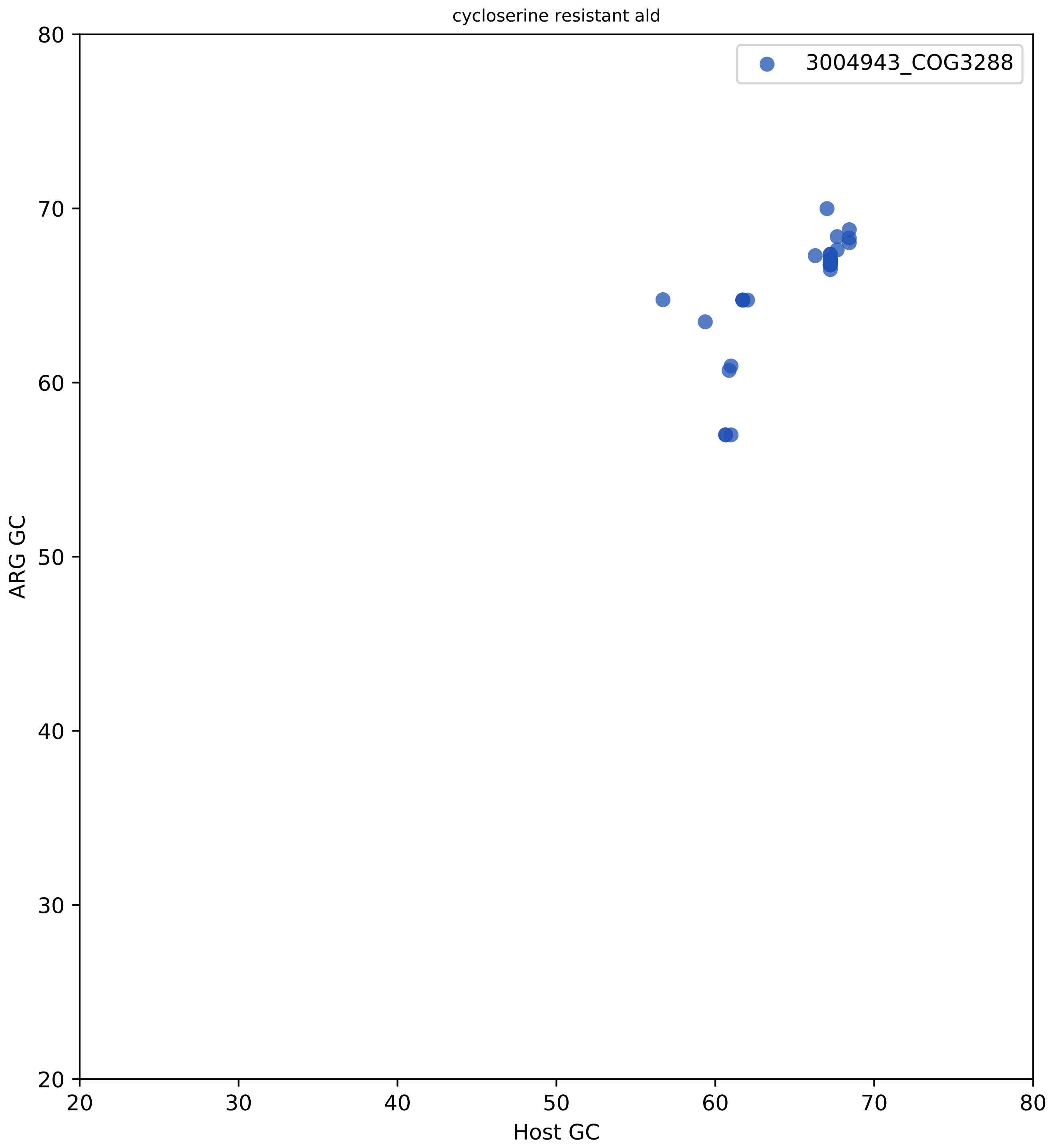

Supplementary Figure S1: (continued).

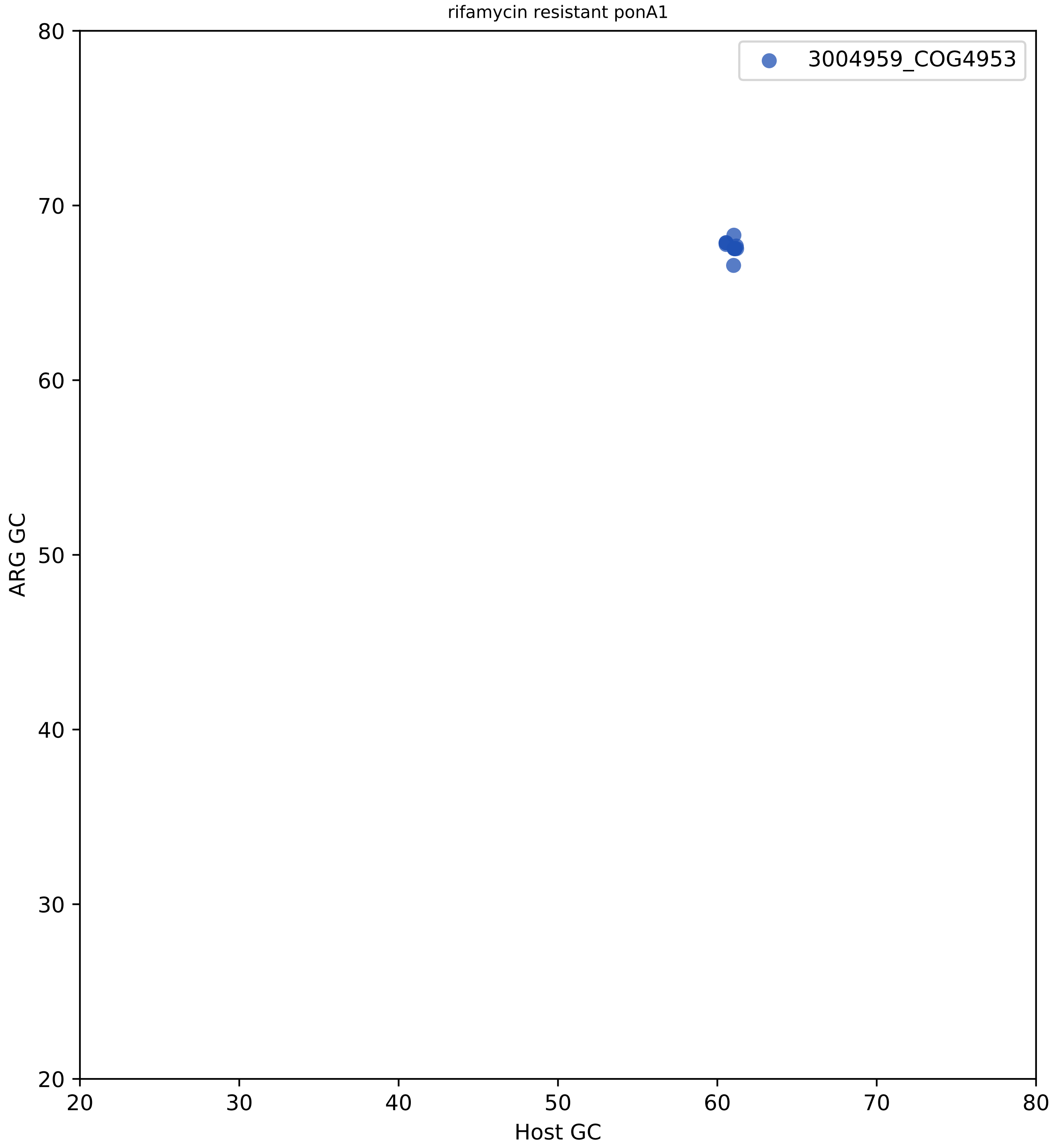

Supplementary Figure S1: (continued).

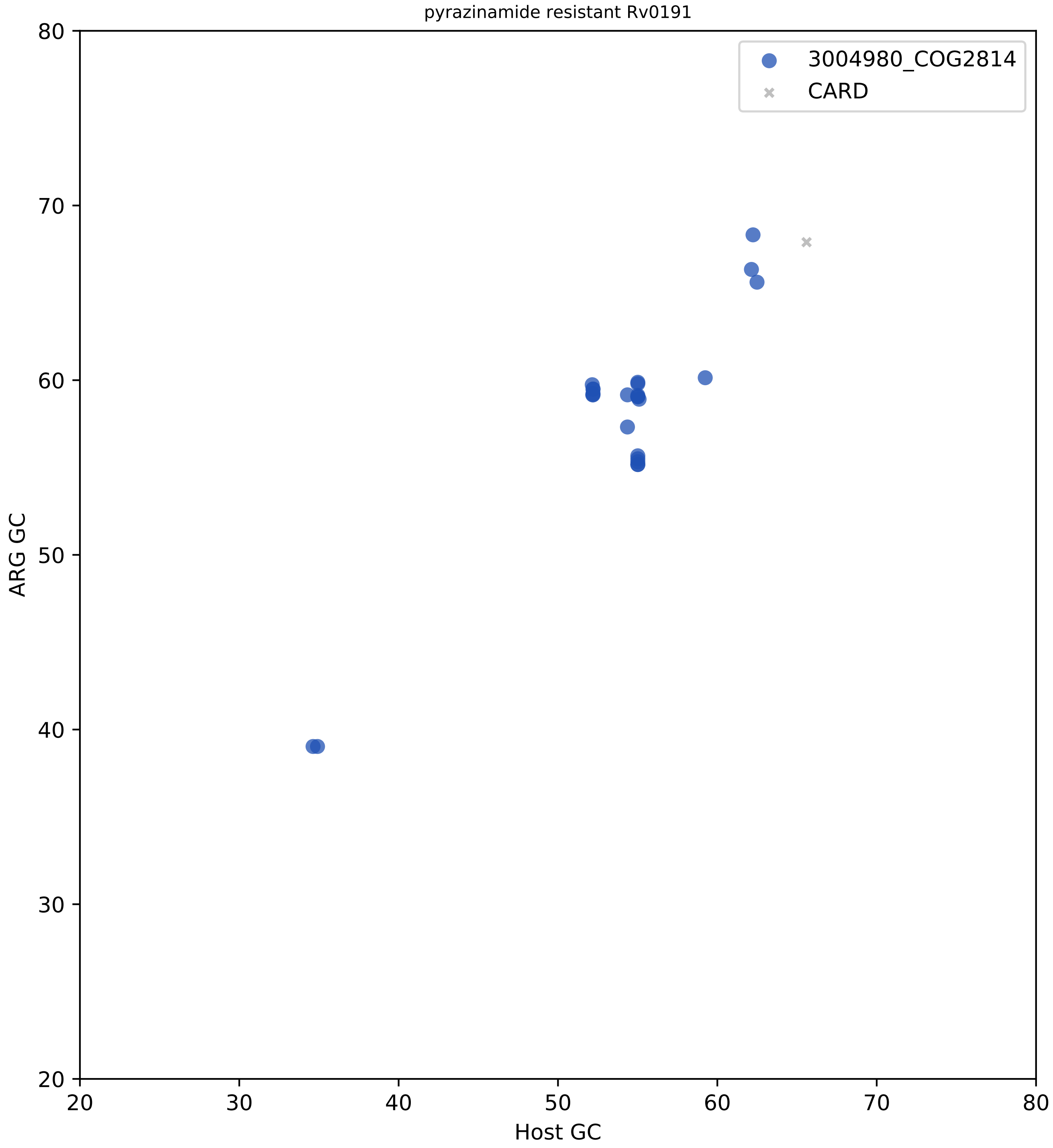

Supplementary Figure S1: (continued).

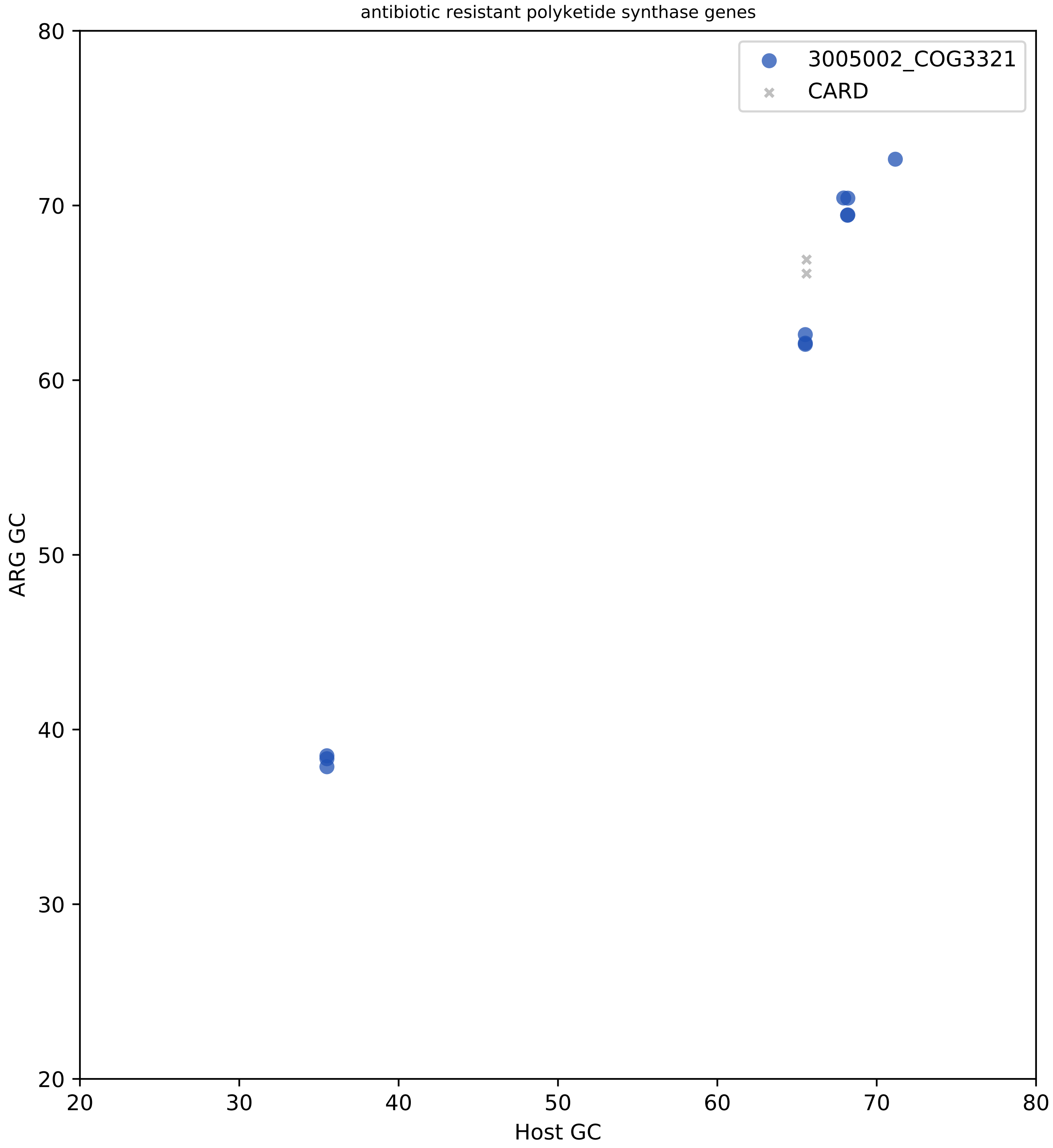

Supplementary Figure S1: (continued).

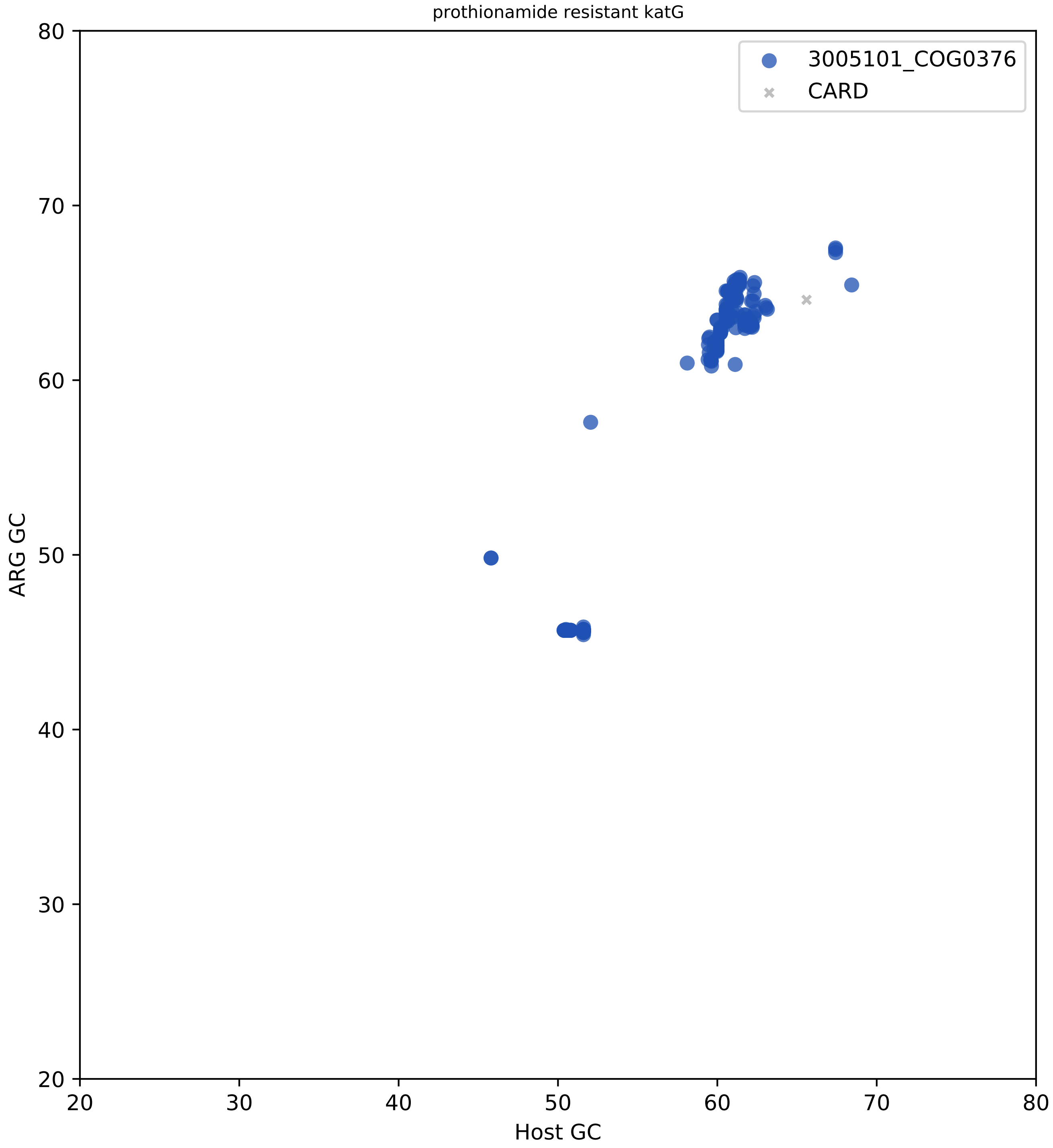

Supplementary Figure S1: (continued).

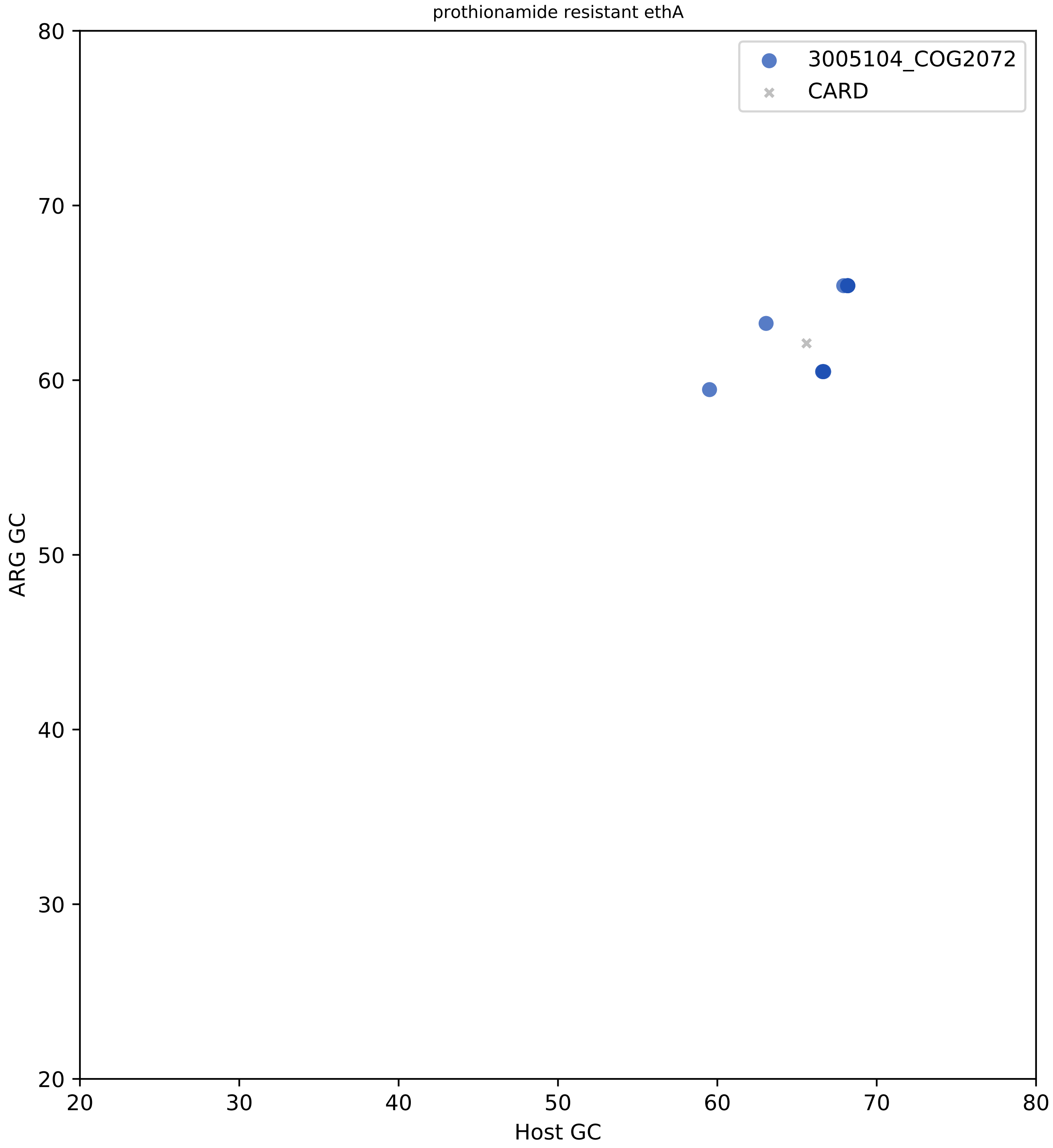

Supplementary Figure S1: (continued).

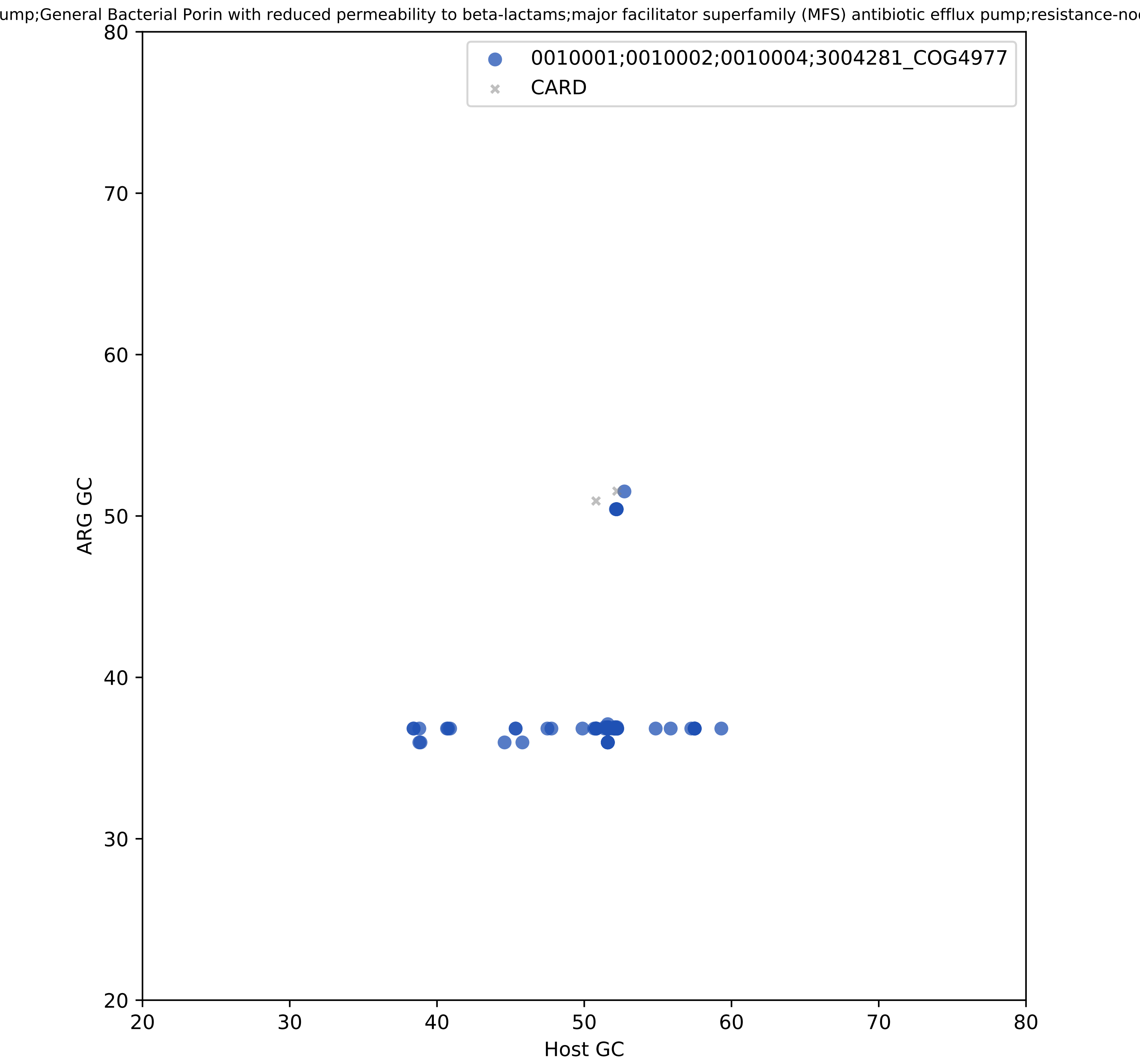

Supplementary Figure S1: (continued).

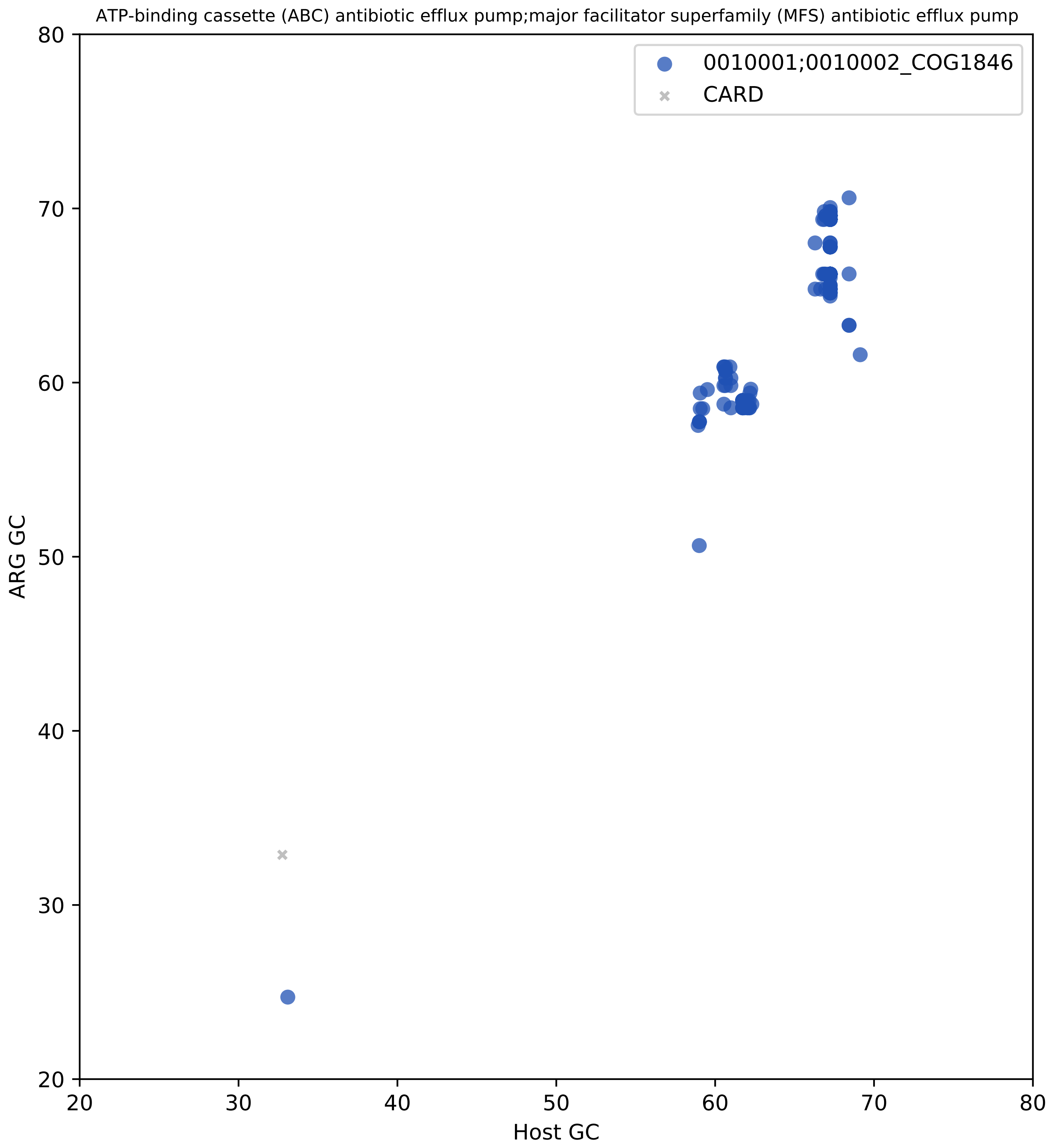

Supplementary Figure S1: (continued).

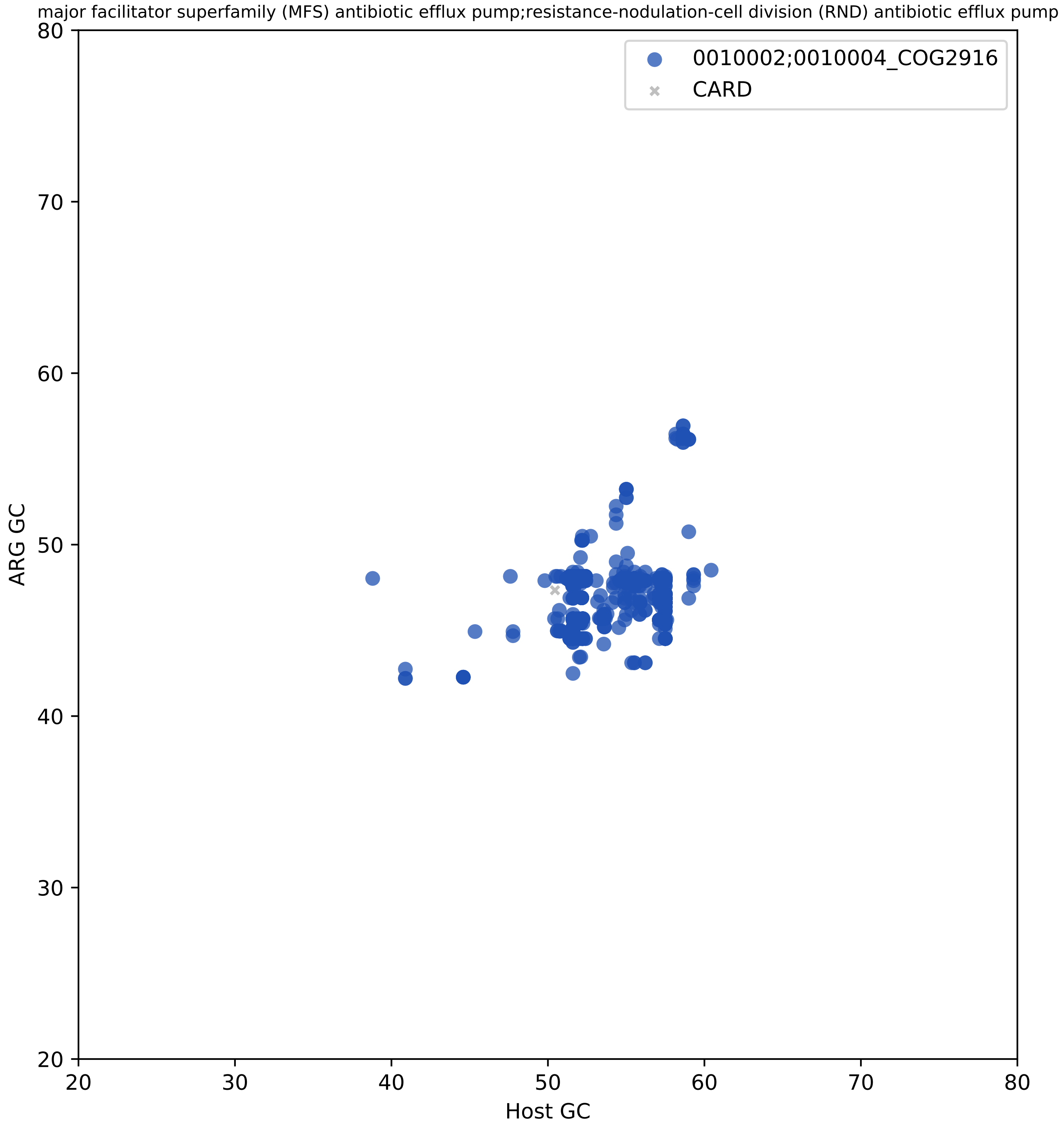

Supplementary Figure S1: (continued).

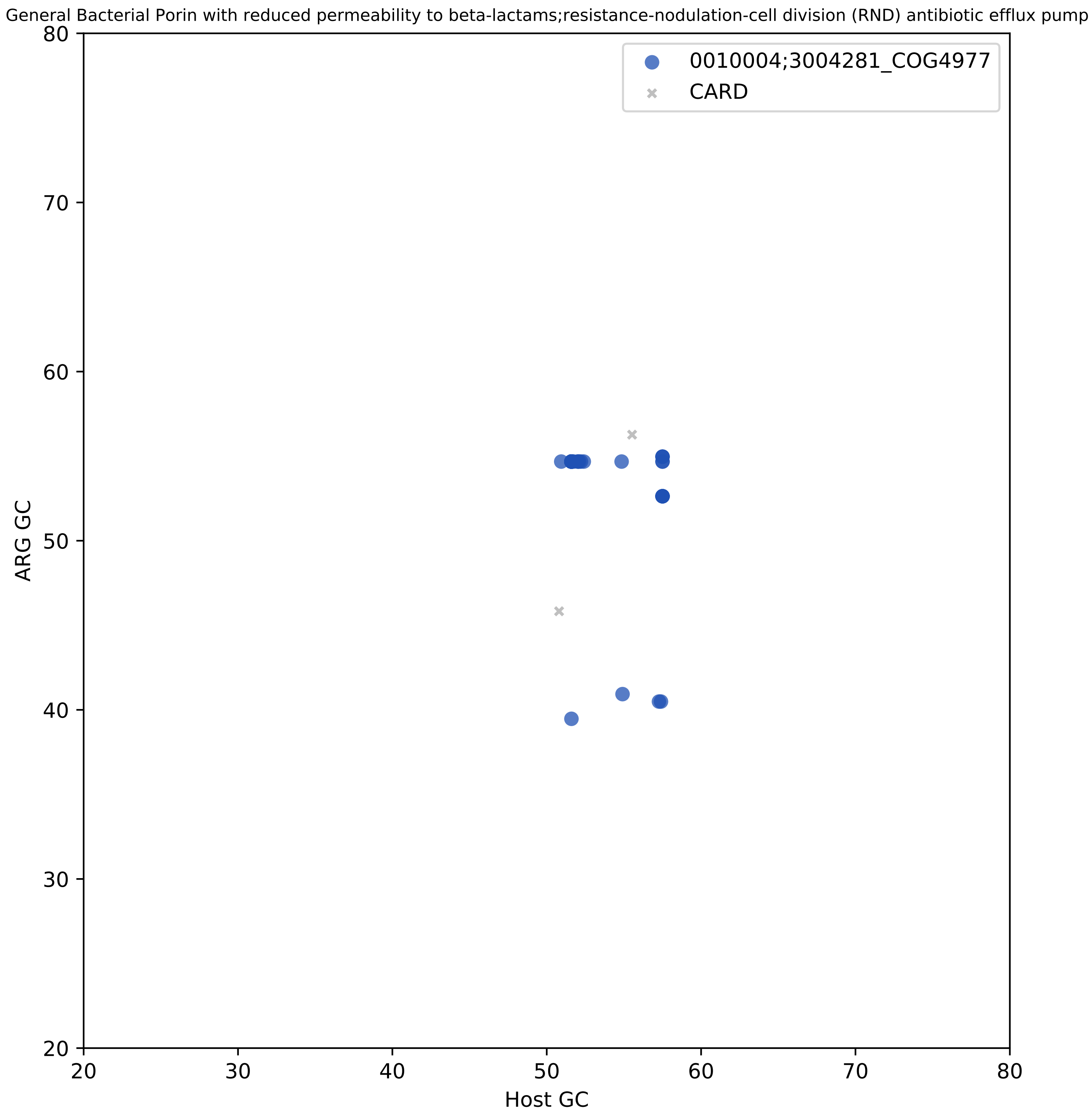

Supplementary Figure S1: (continued).

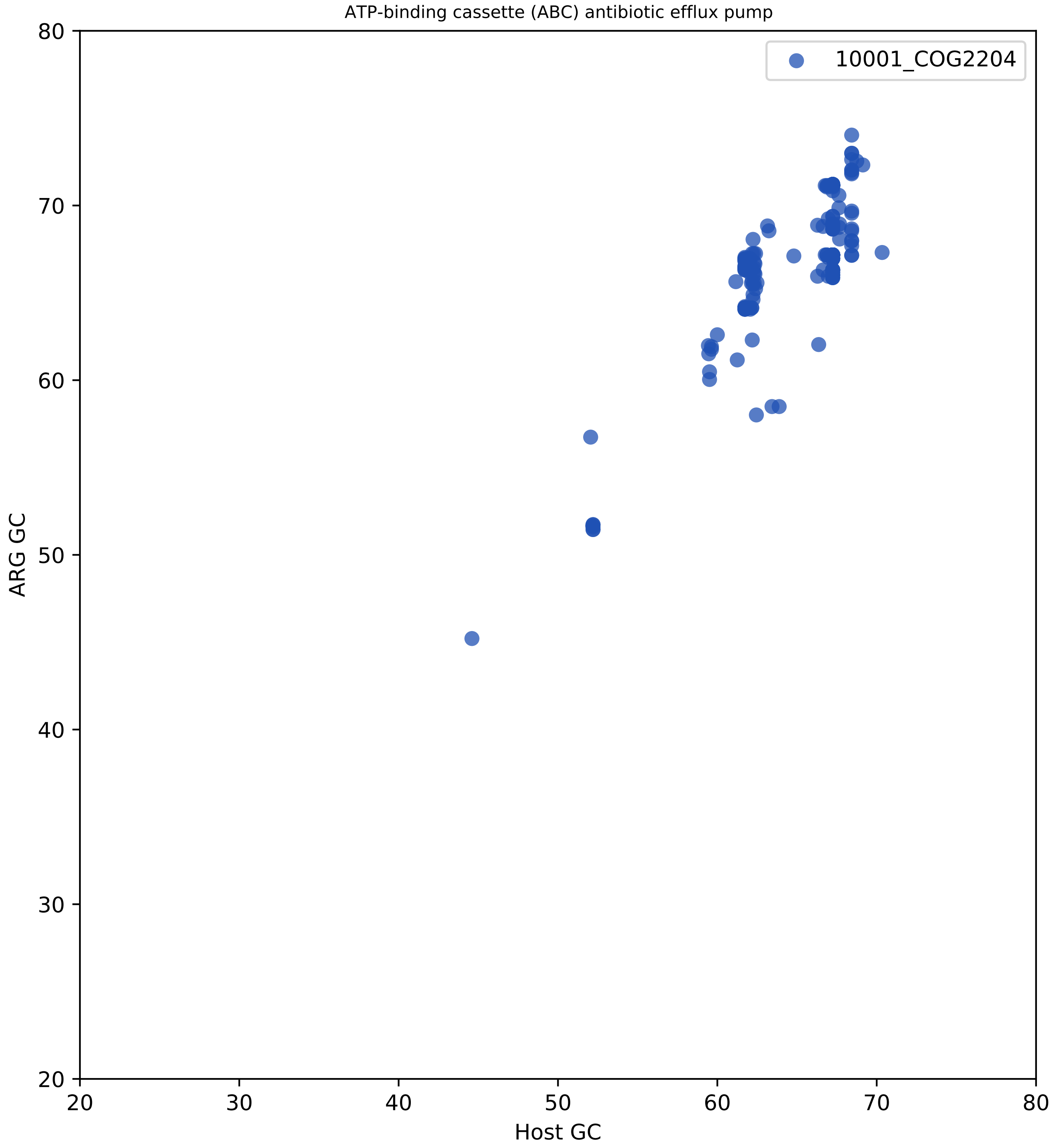

Supplementary Figure S1: (continued).

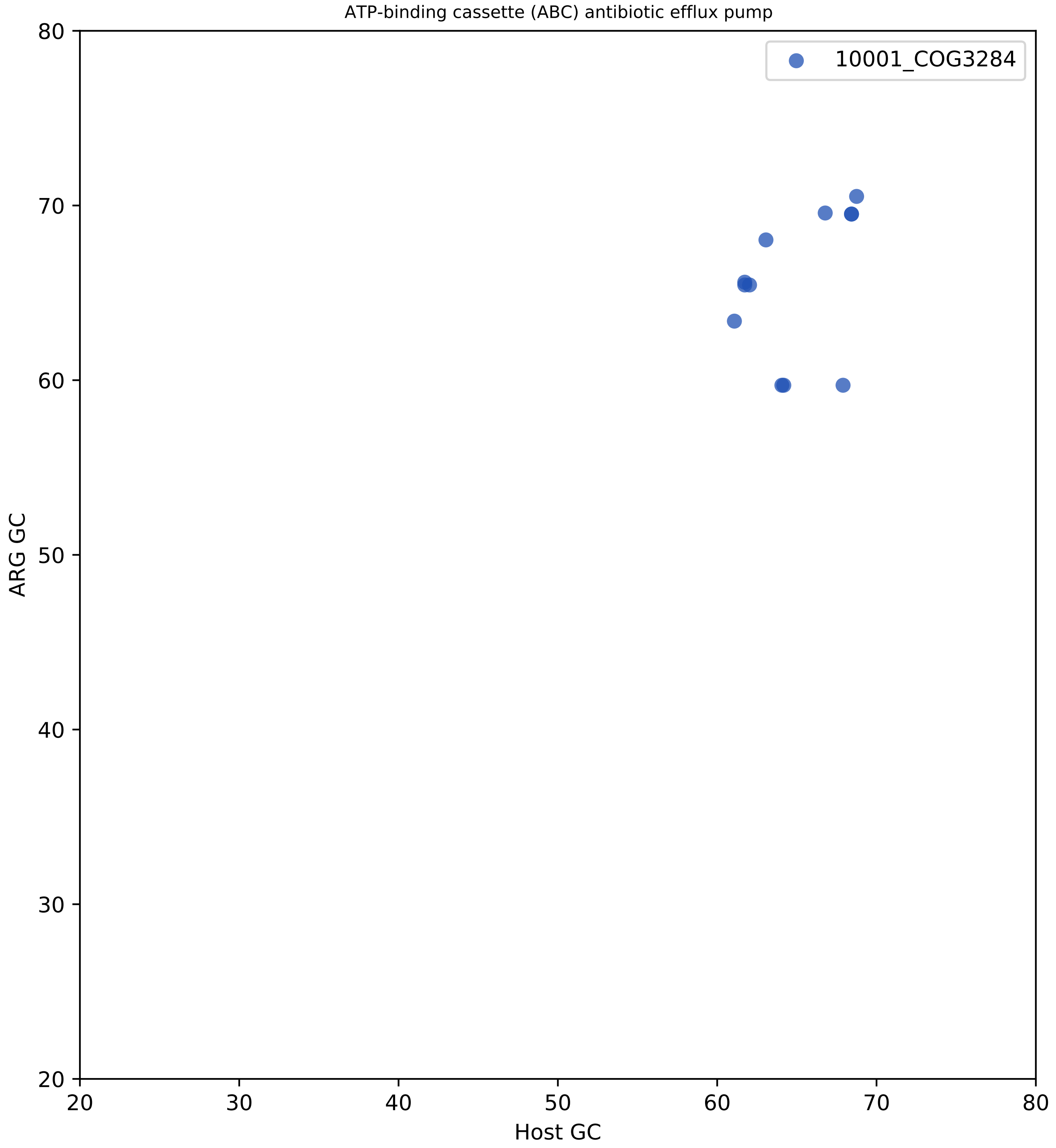

Supplementary Figure S1: (continued).

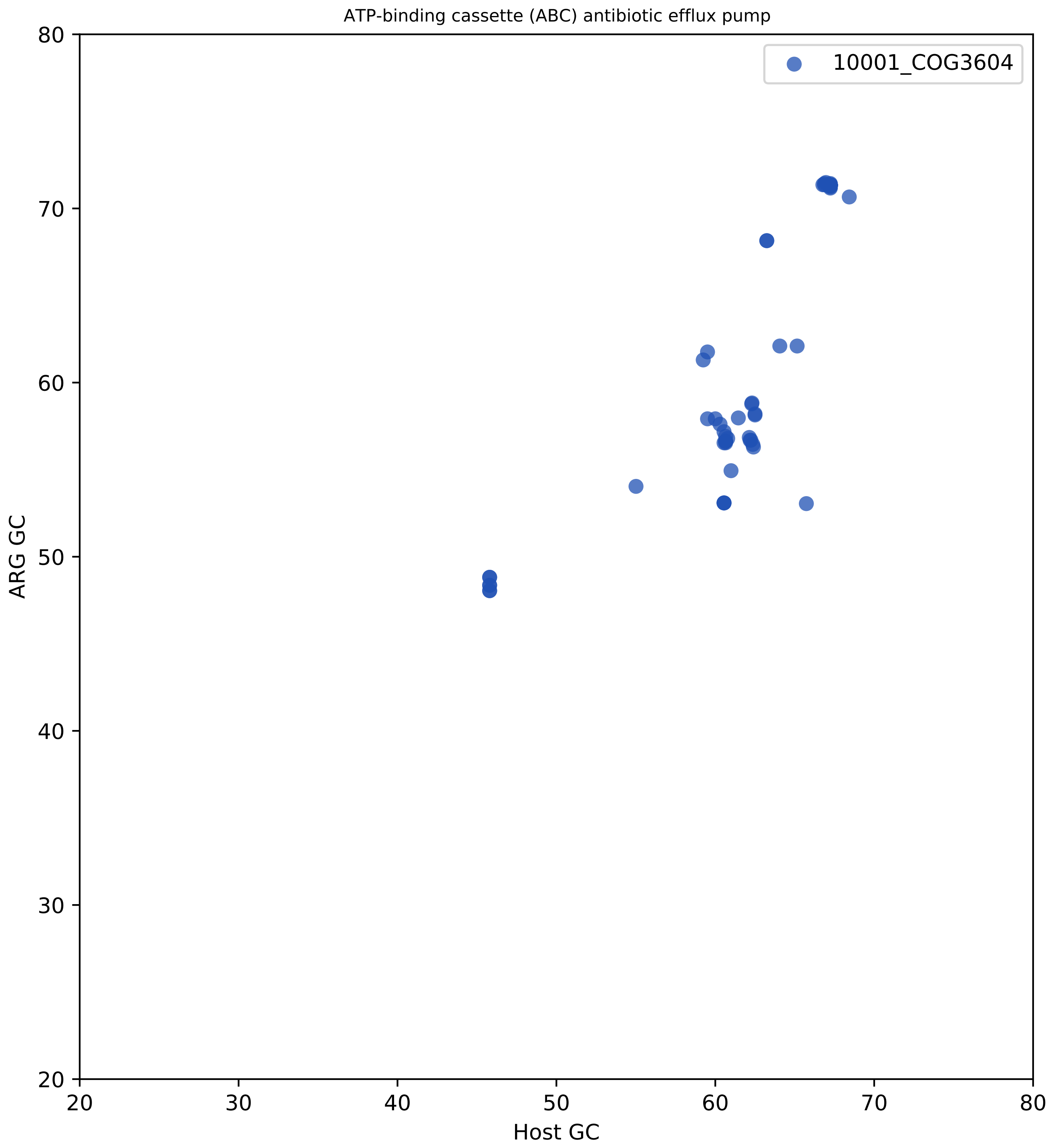

Supplementary Figure S1: (continued).

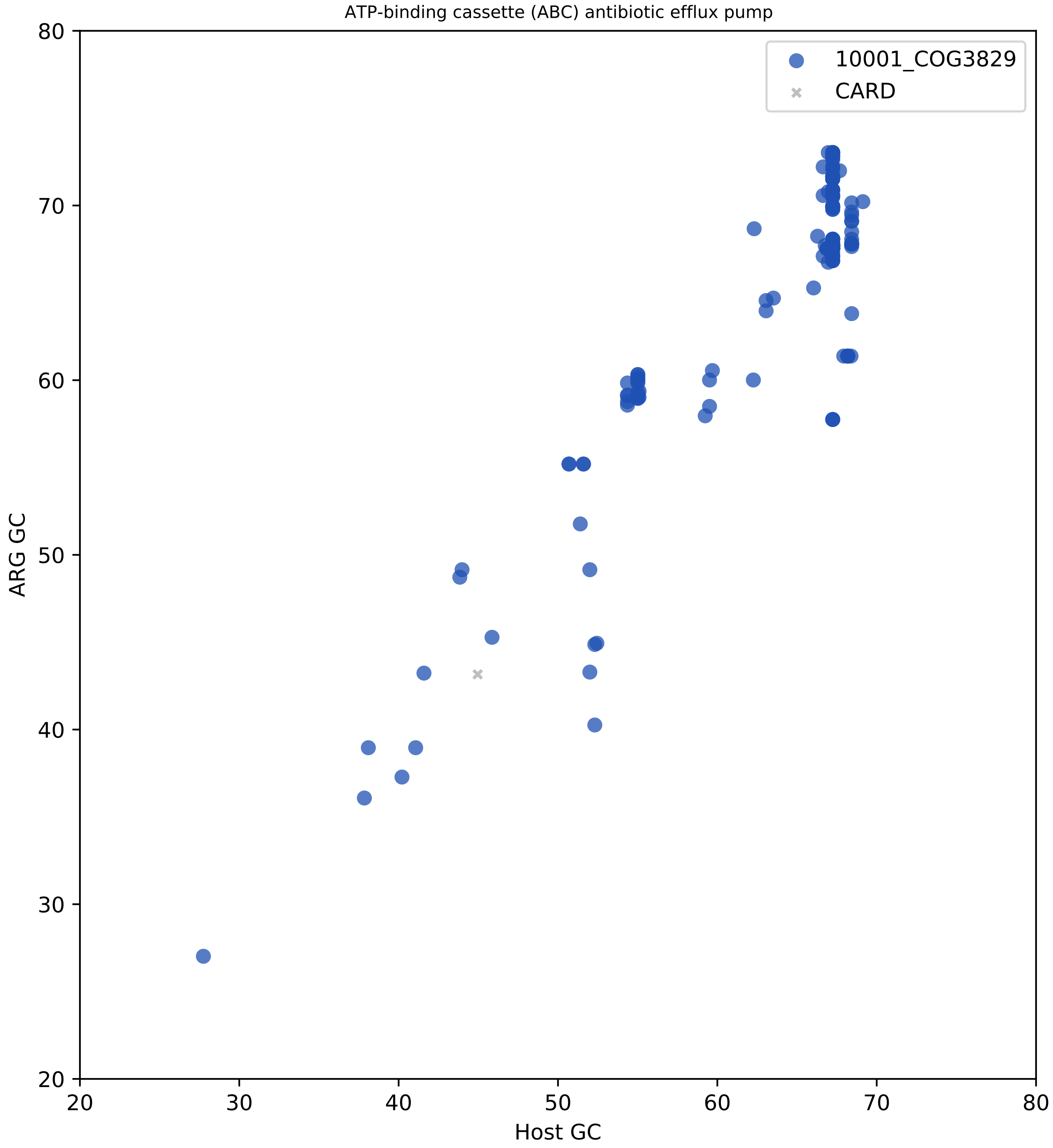

Supplementary Figure S1: (continued).

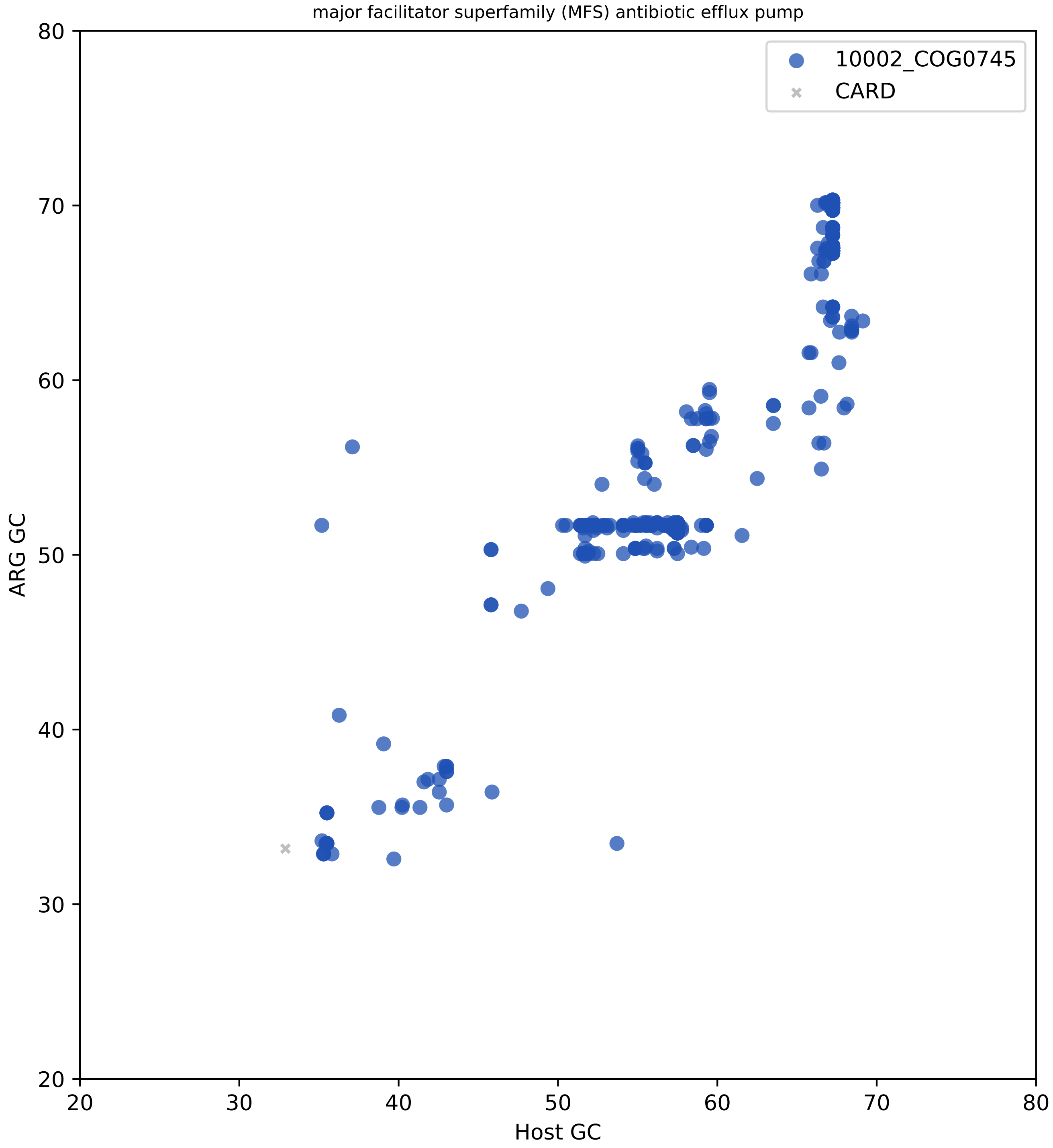

Supplementary Figure S1: (continued).

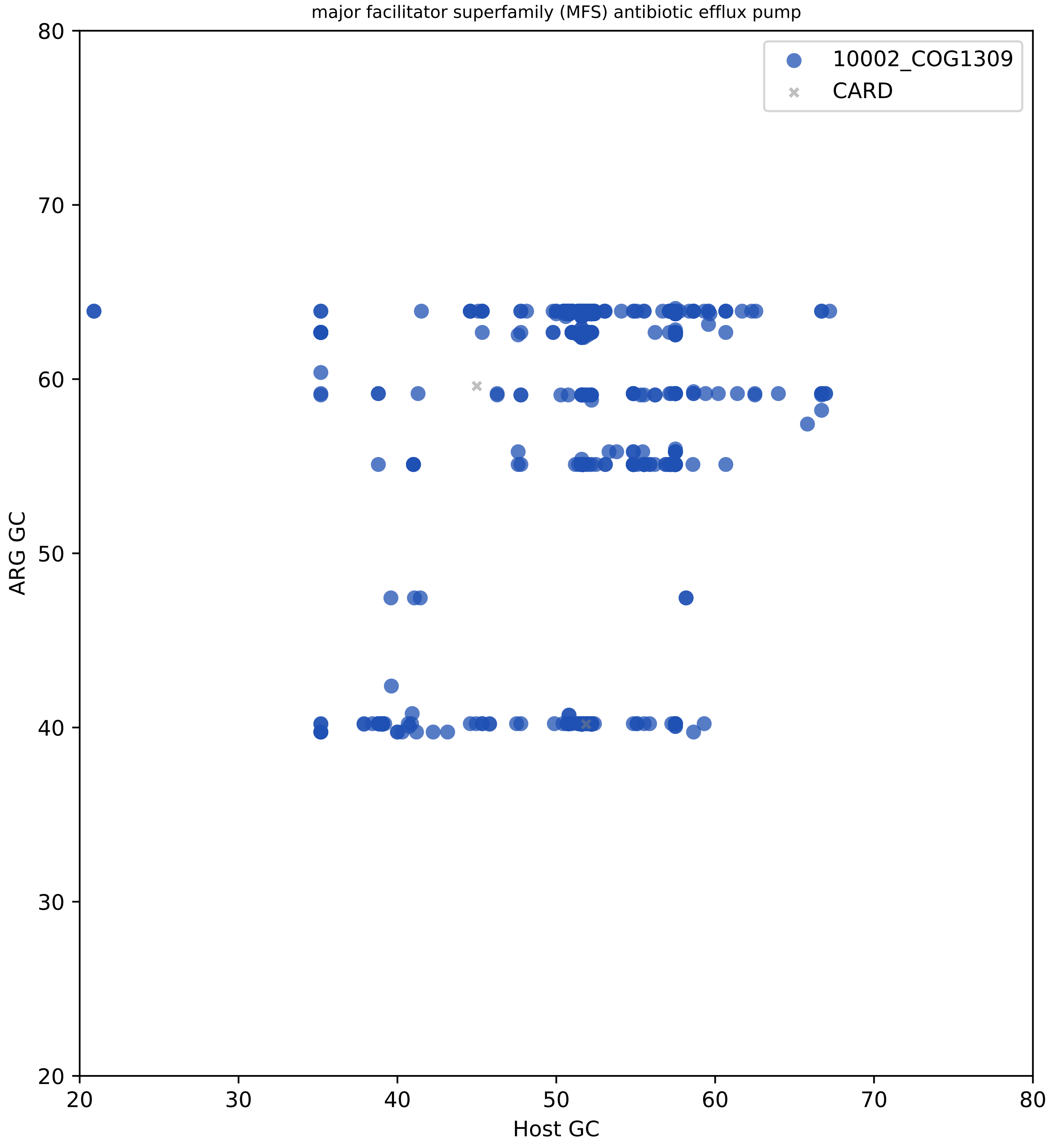

Supplementary Figure S1: (continued).

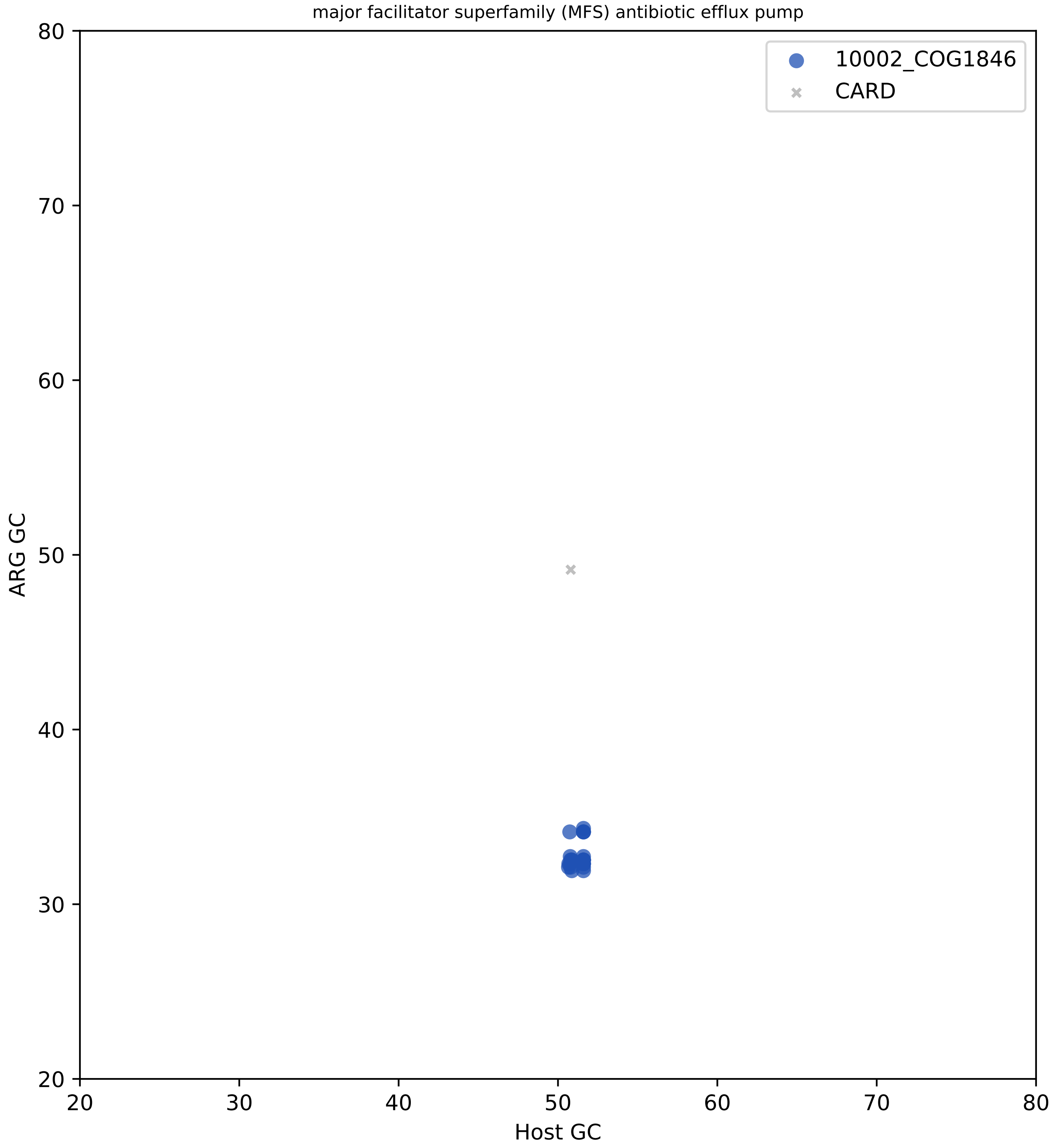

Supplementary Figure S1: (continued).

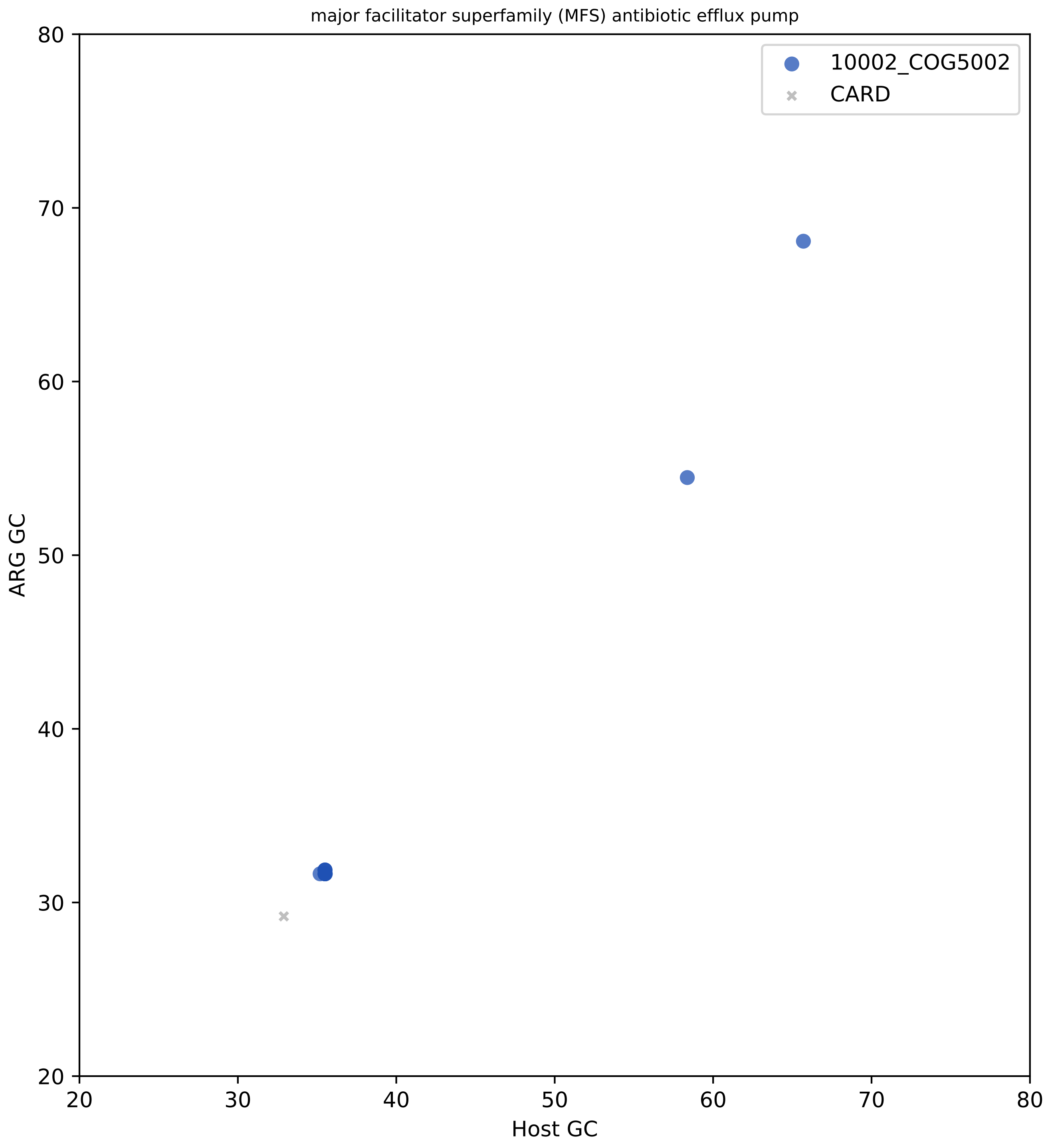

Supplementary Figure S1: (continued).

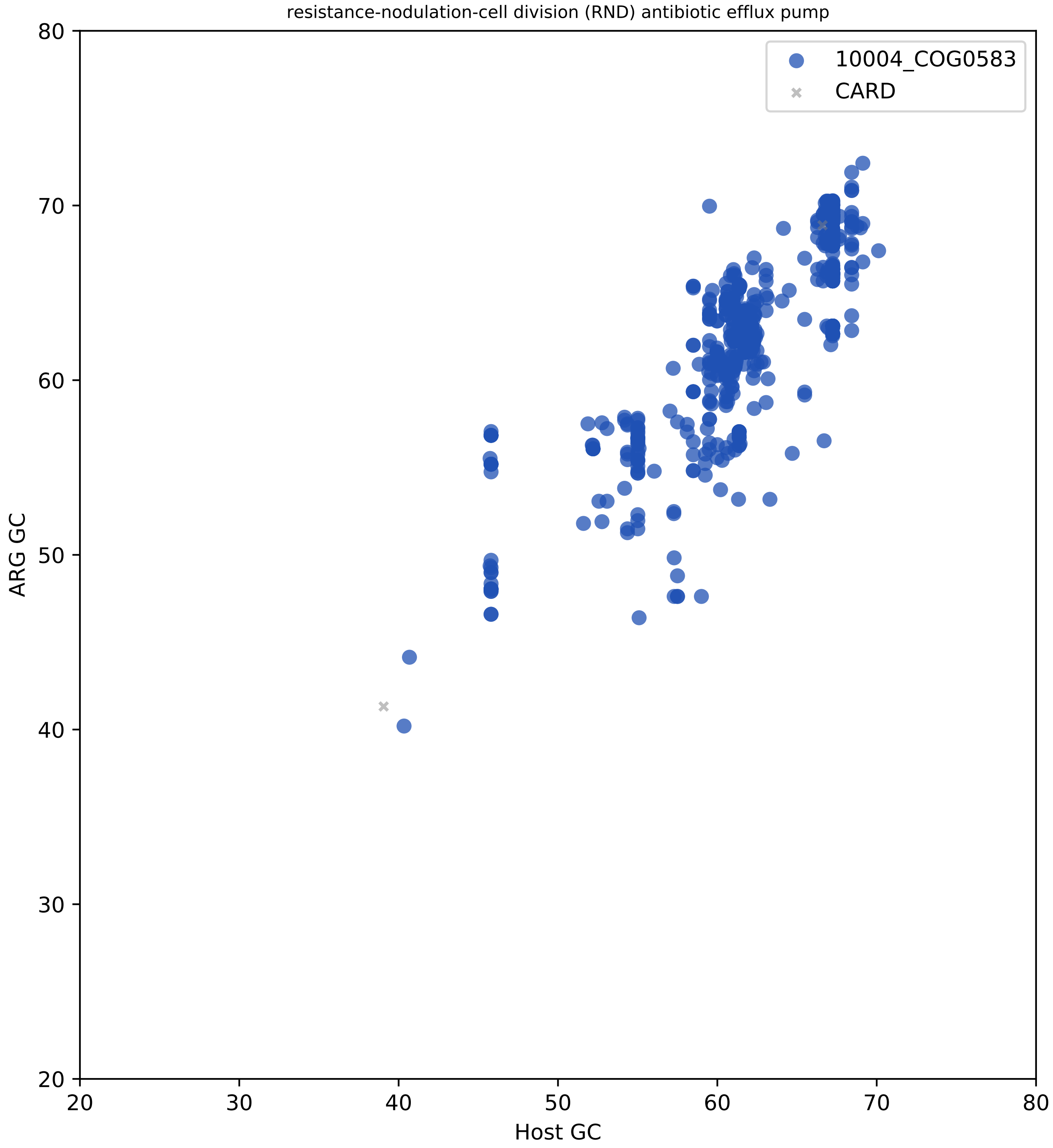

Supplementary Figure S1: (continued).

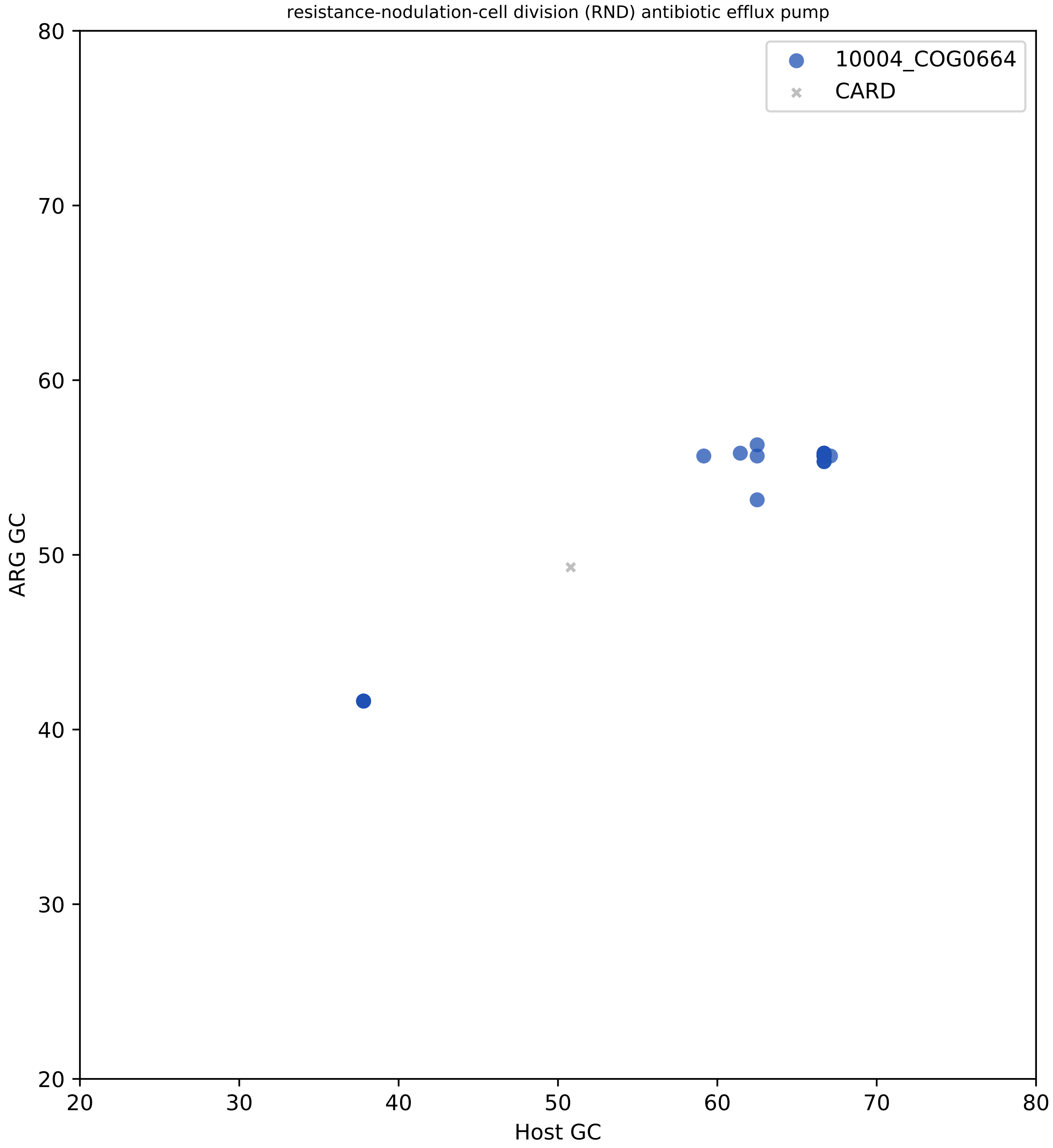

Supplementary Figure S1: (continued).

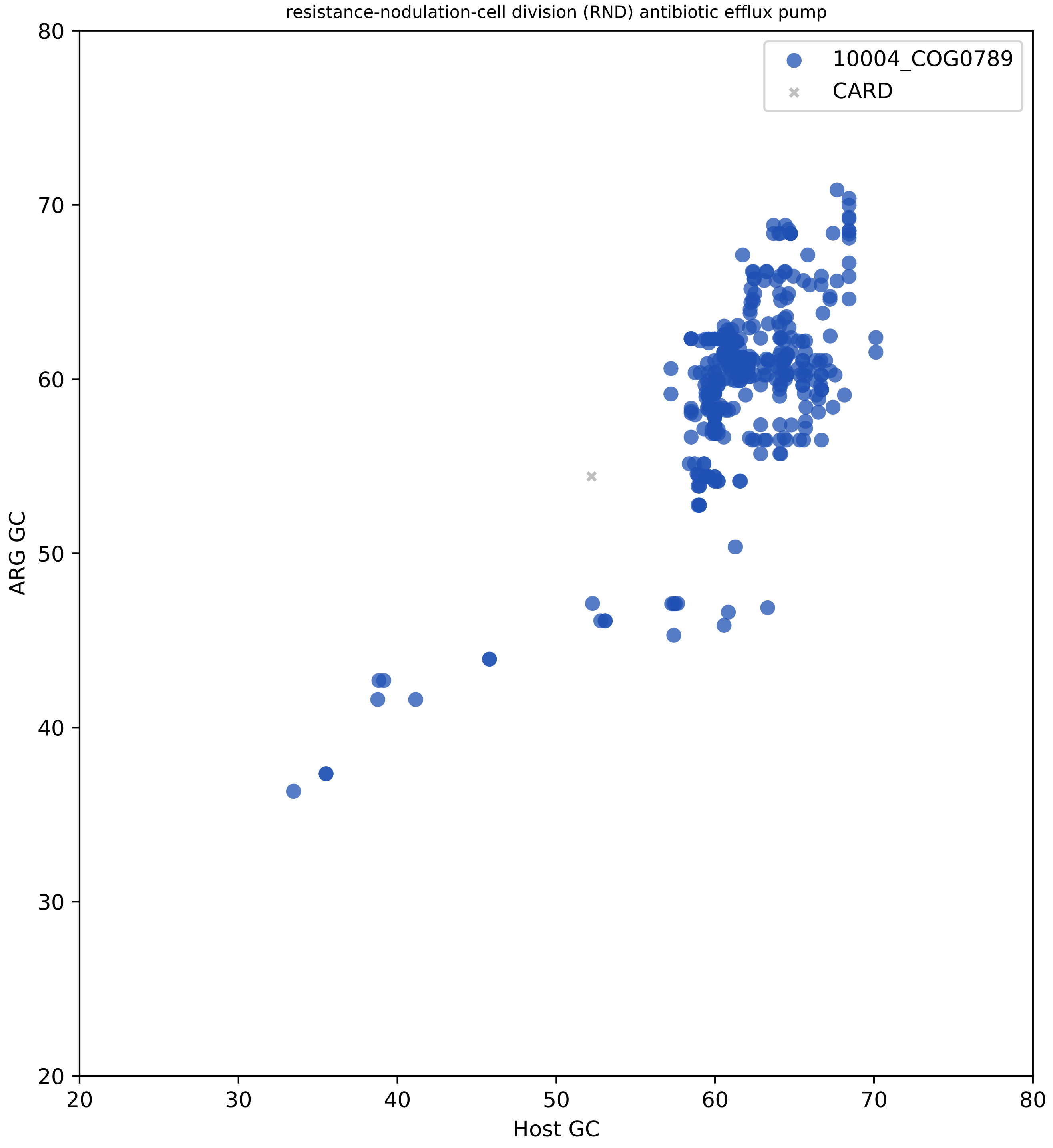

Supplementary Figure S1: (continued).

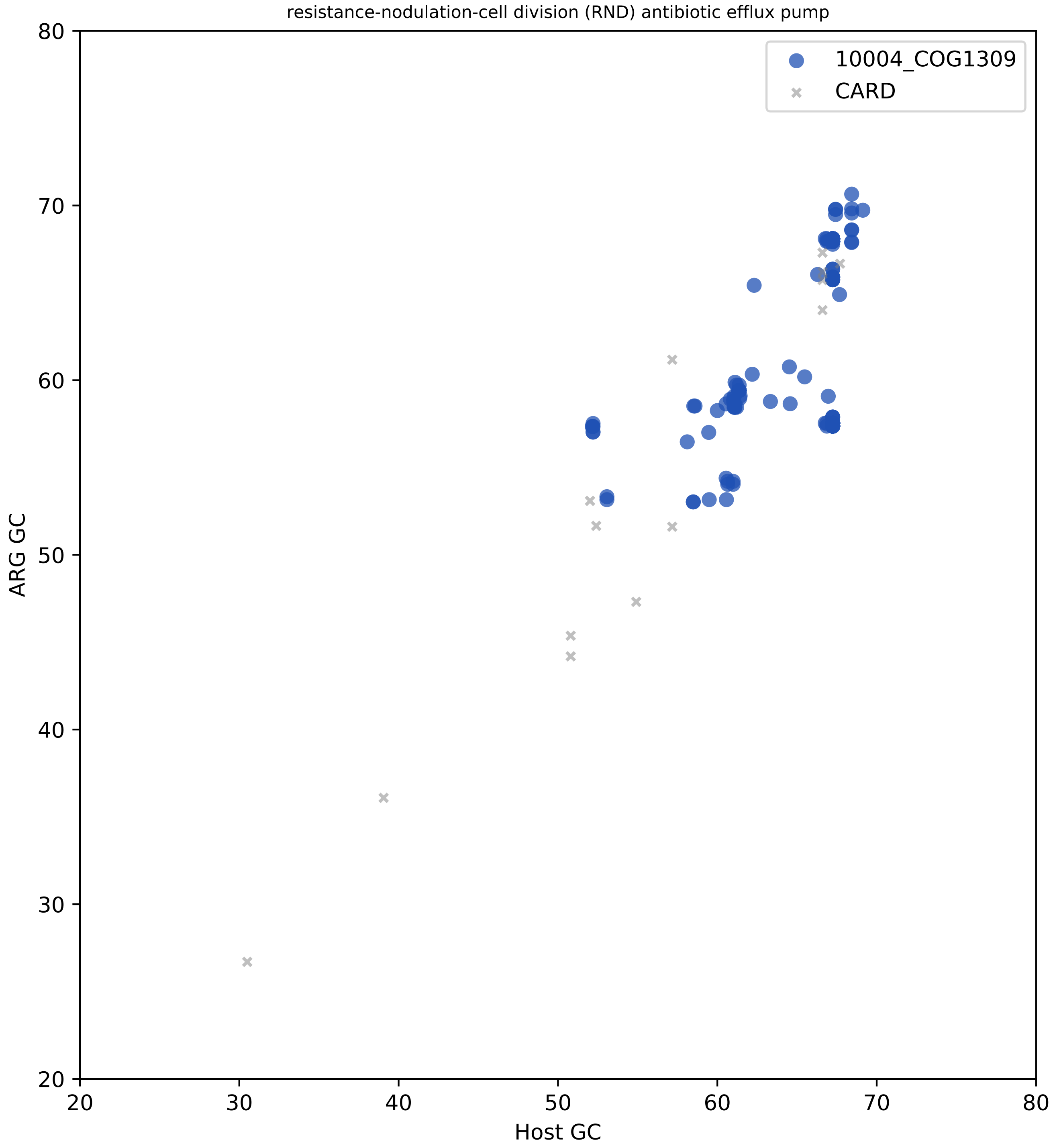

Supplementary Figure S1: (continued).

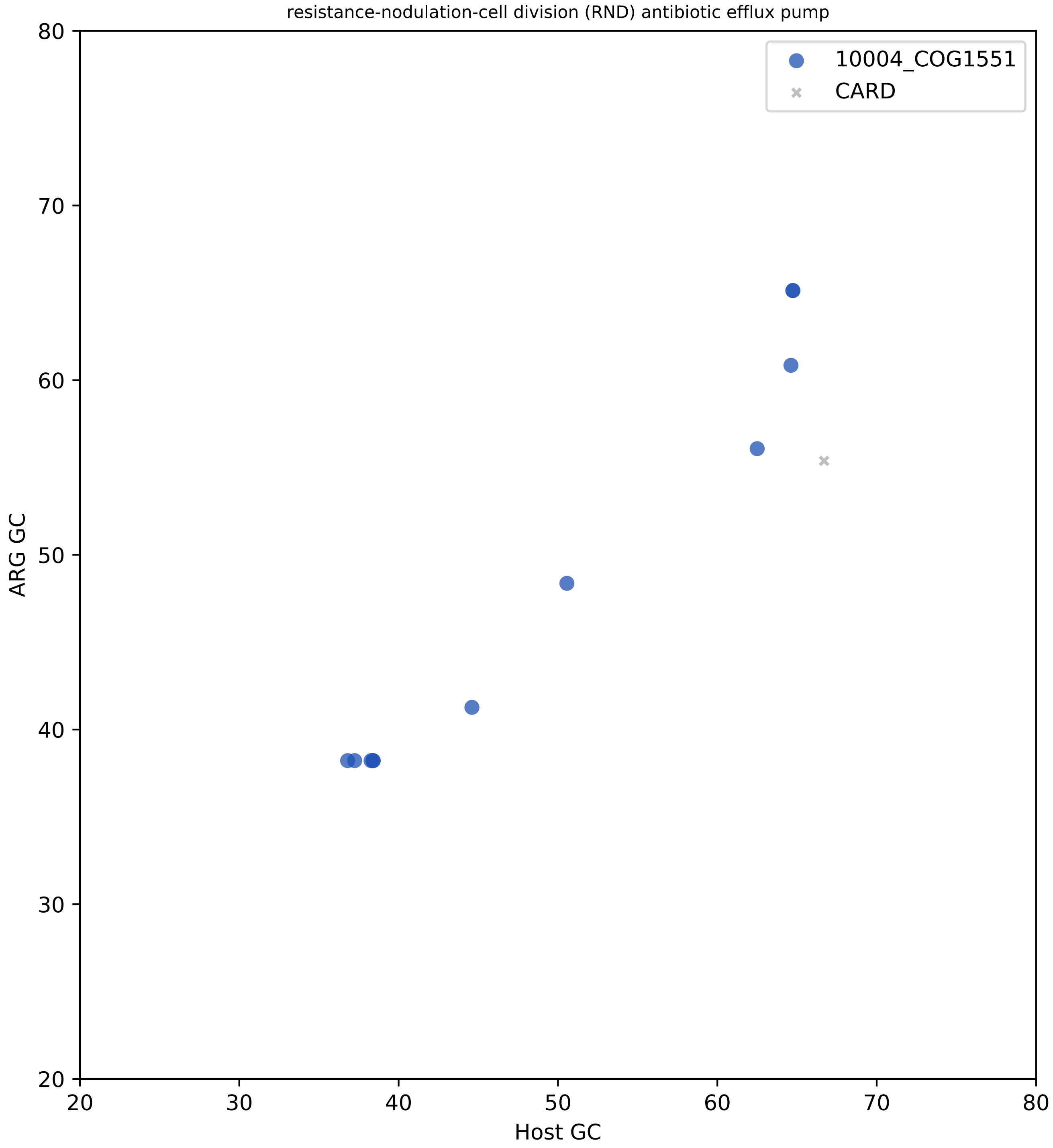

Supplementary Figure S1: (continued).

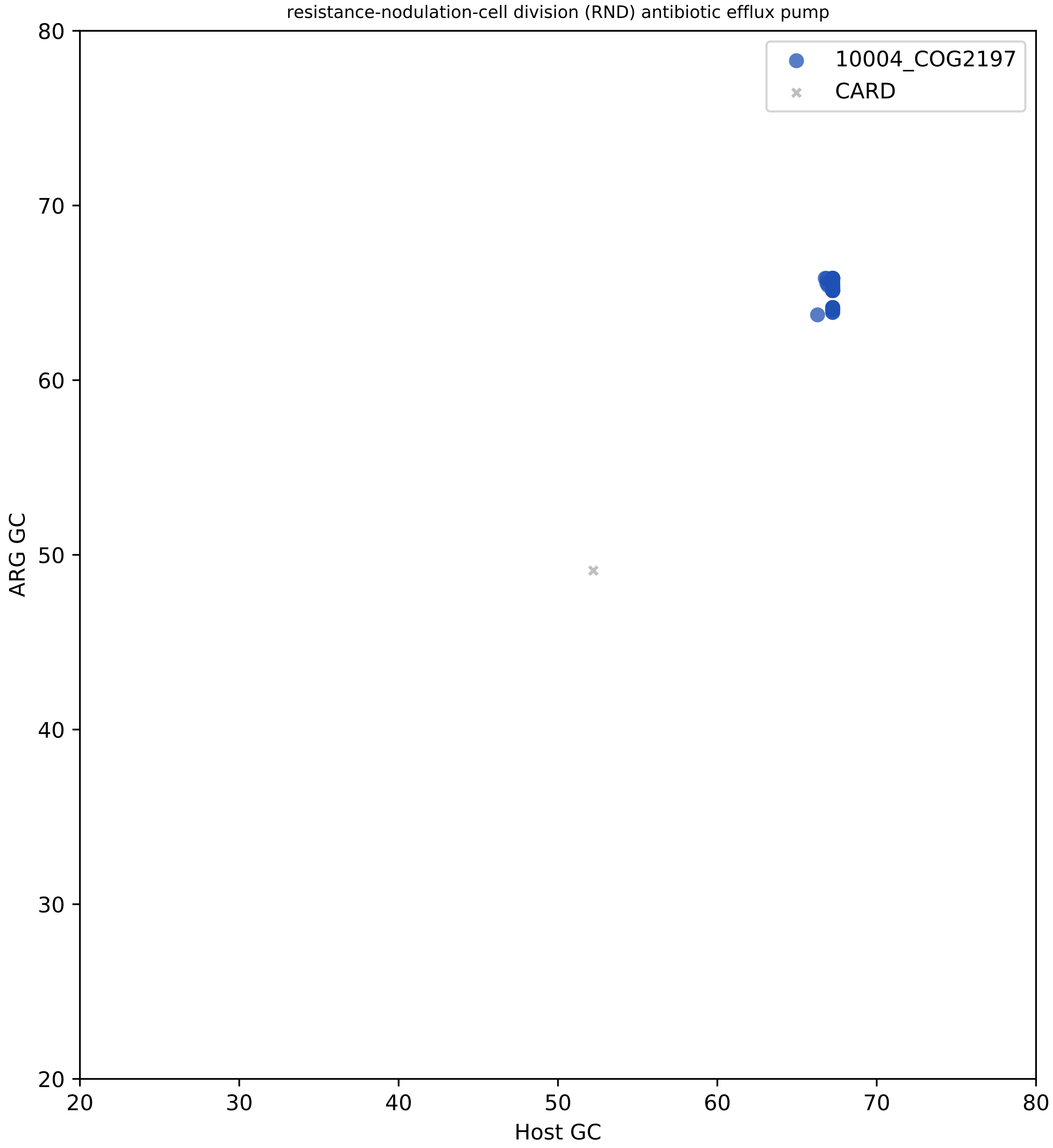

Supplementary Figure S1: (continued).

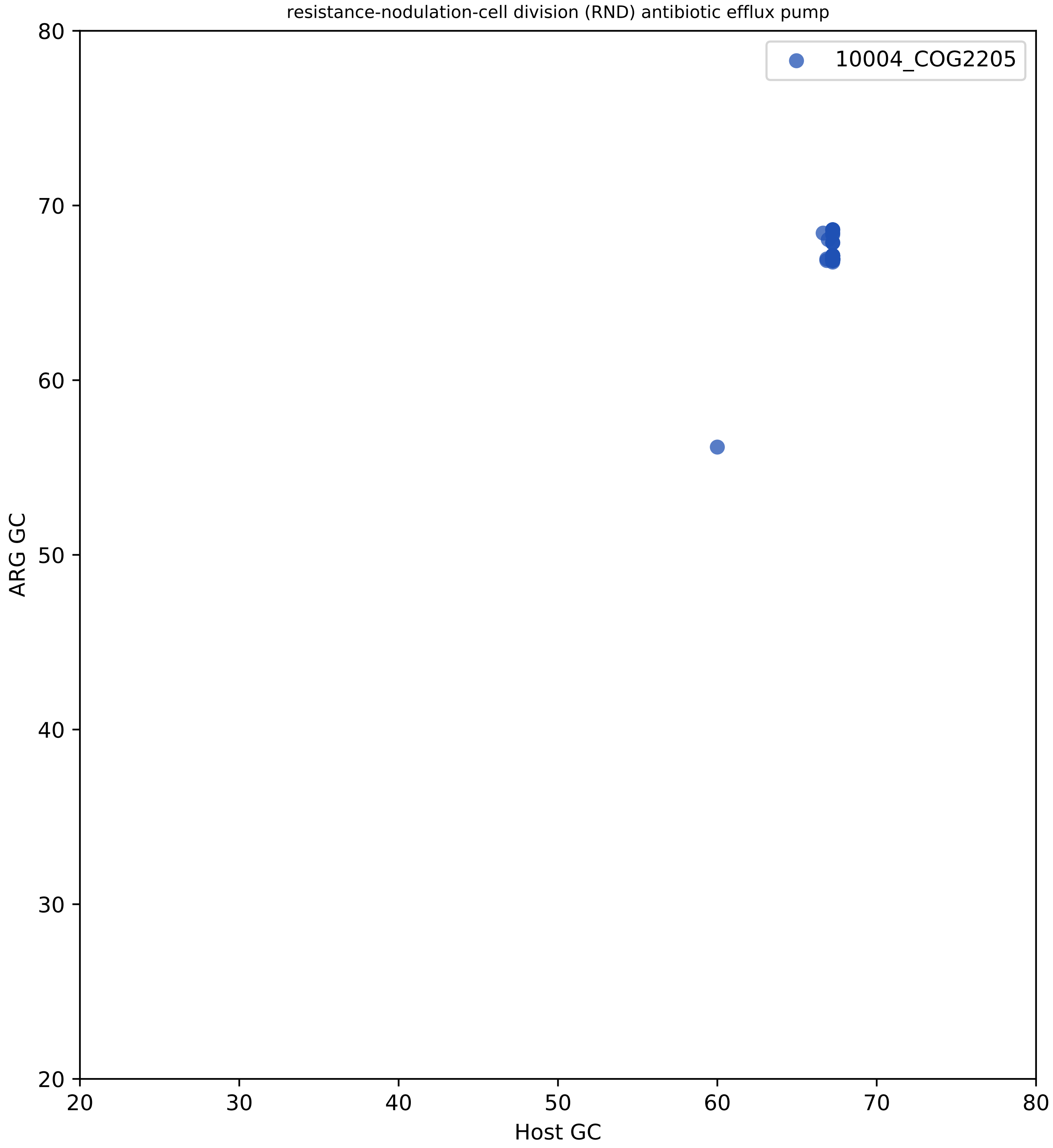

Supplementary Figure S1: (continued).

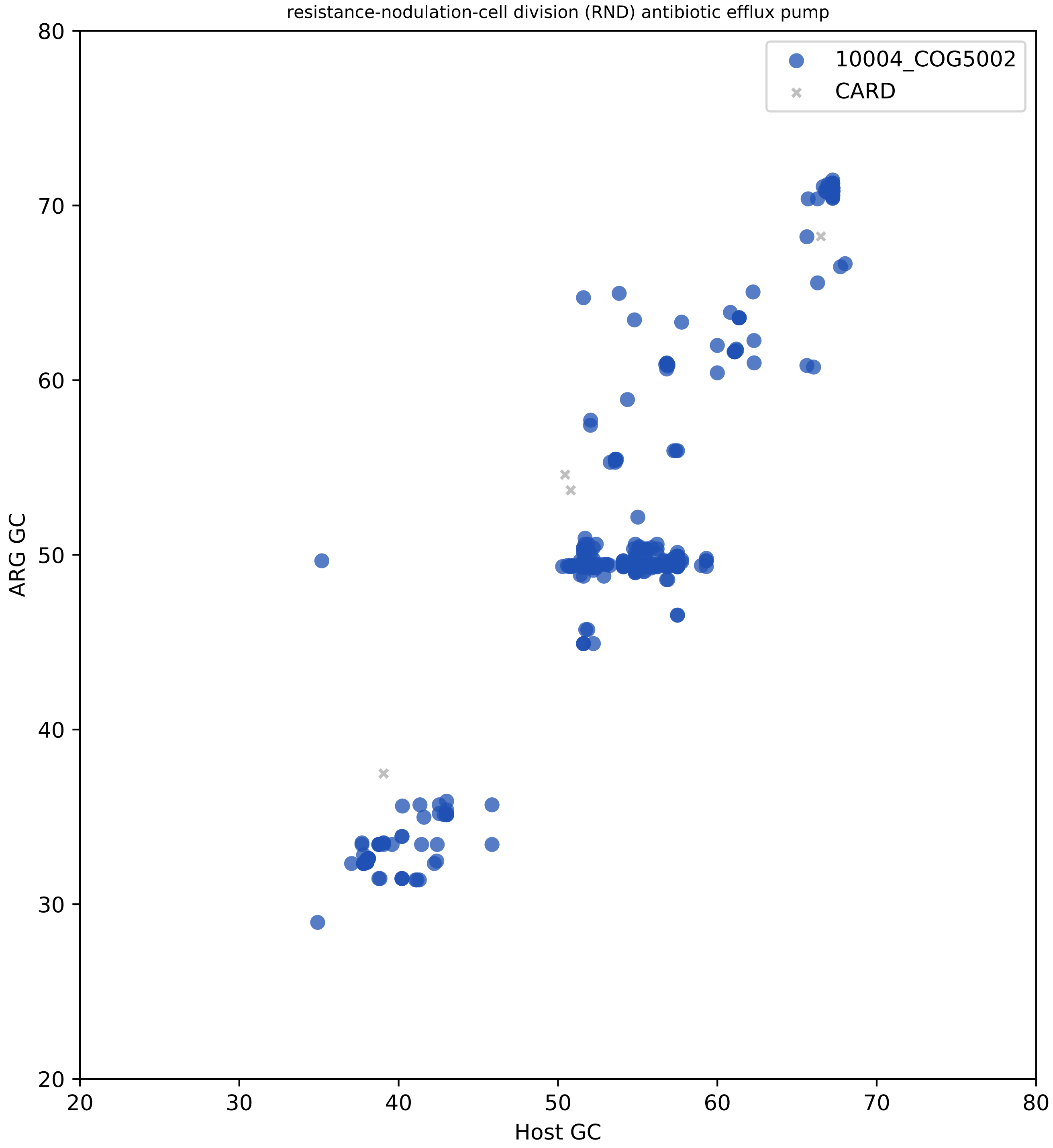

Supplementary Figure S1: (continued).

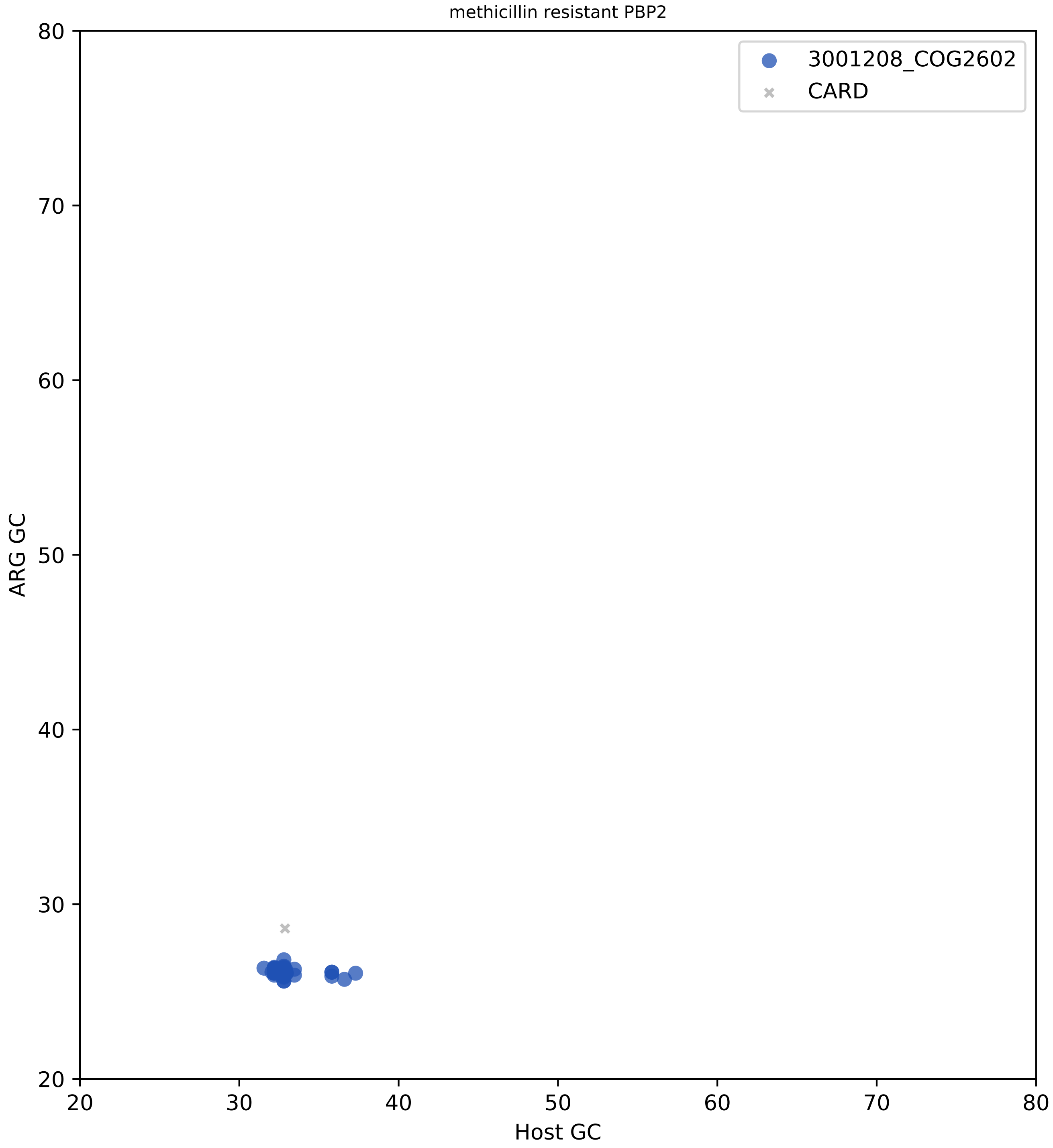

Supplementary Figure S1: (continued).

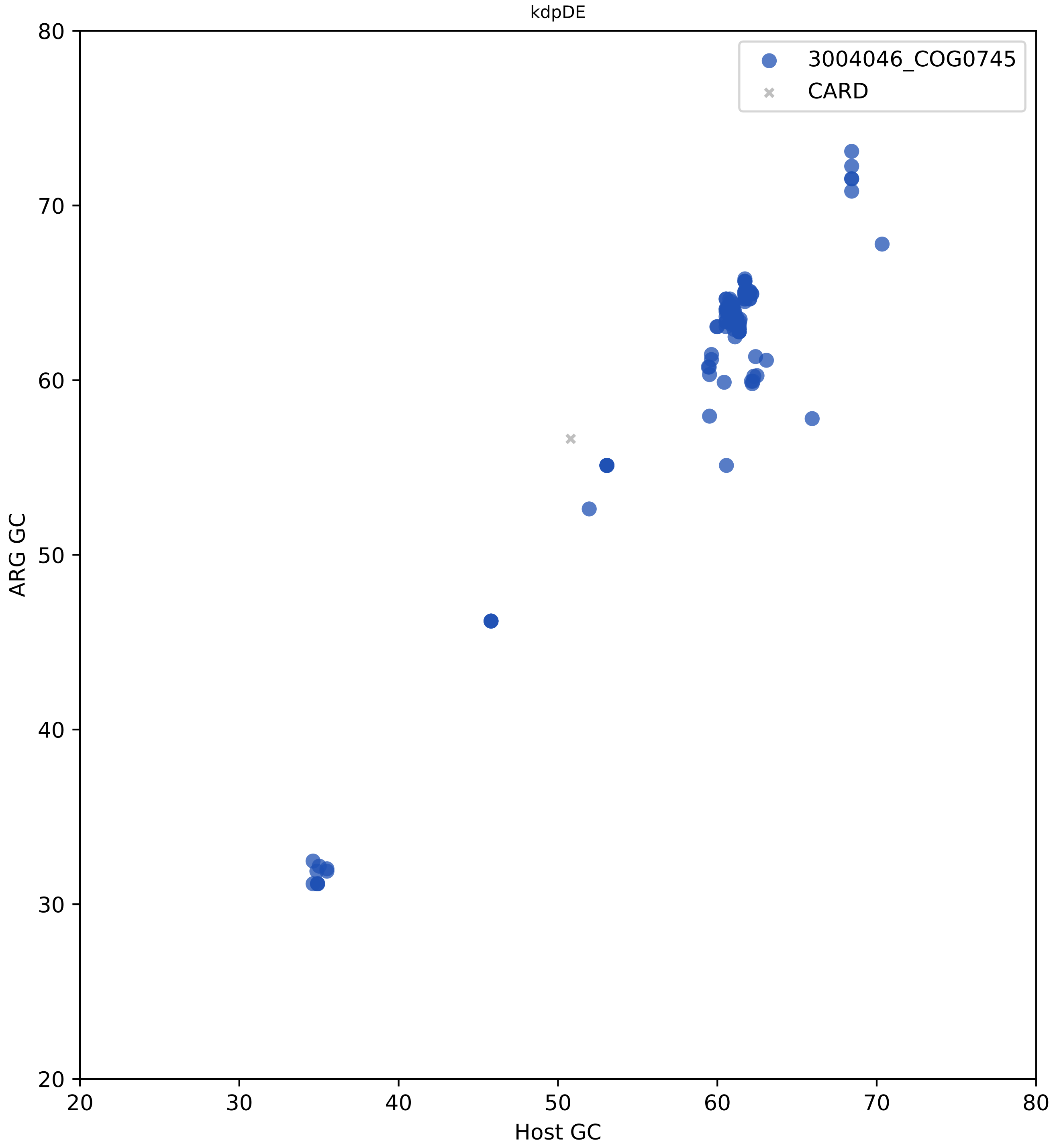

Supplementary Figure S1: (continued).

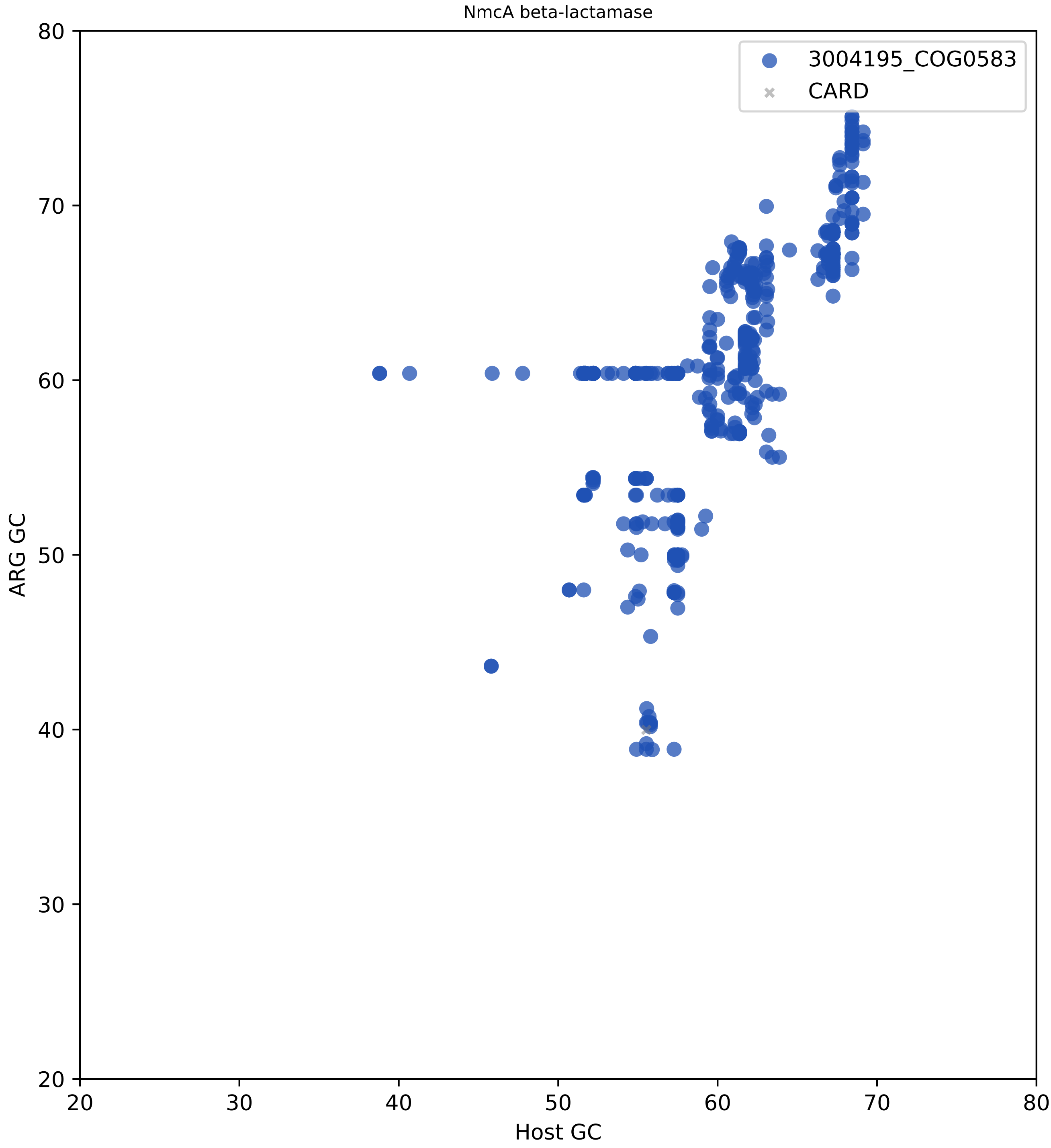

Supplementary Figure S1: (continued).

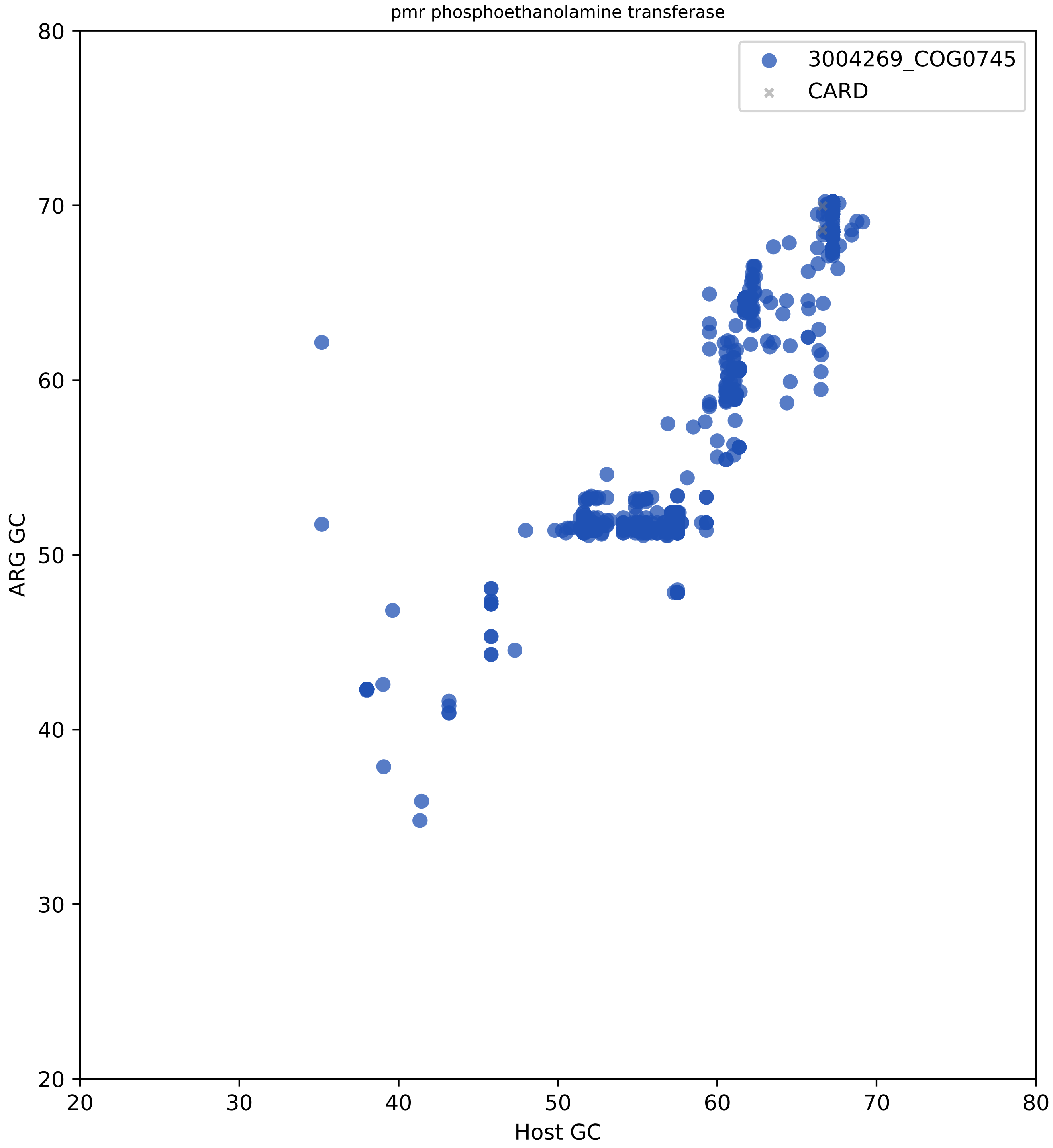

Supplementary Figure S1: (continued).

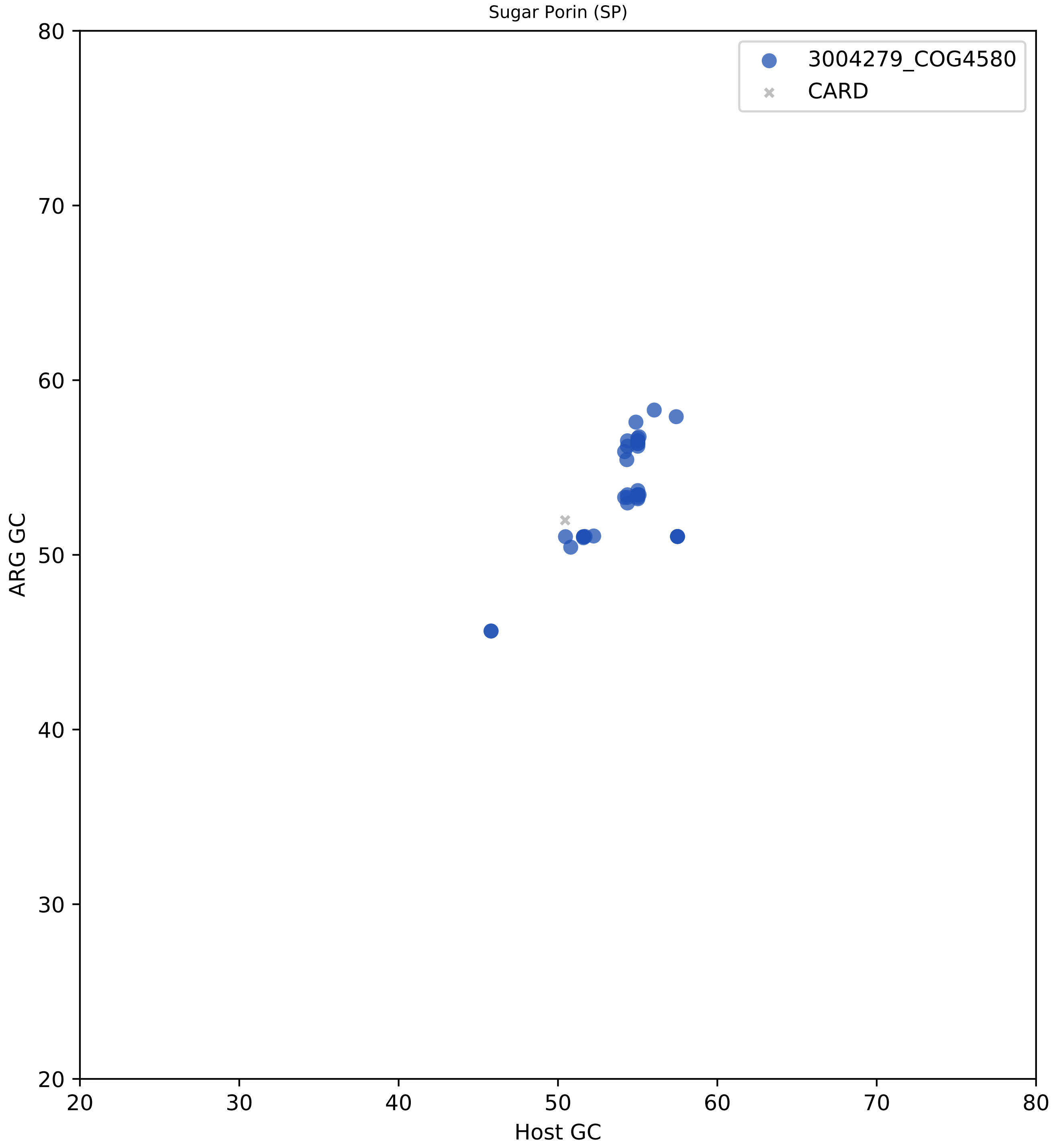

Supplementary Figure S1: (continued).

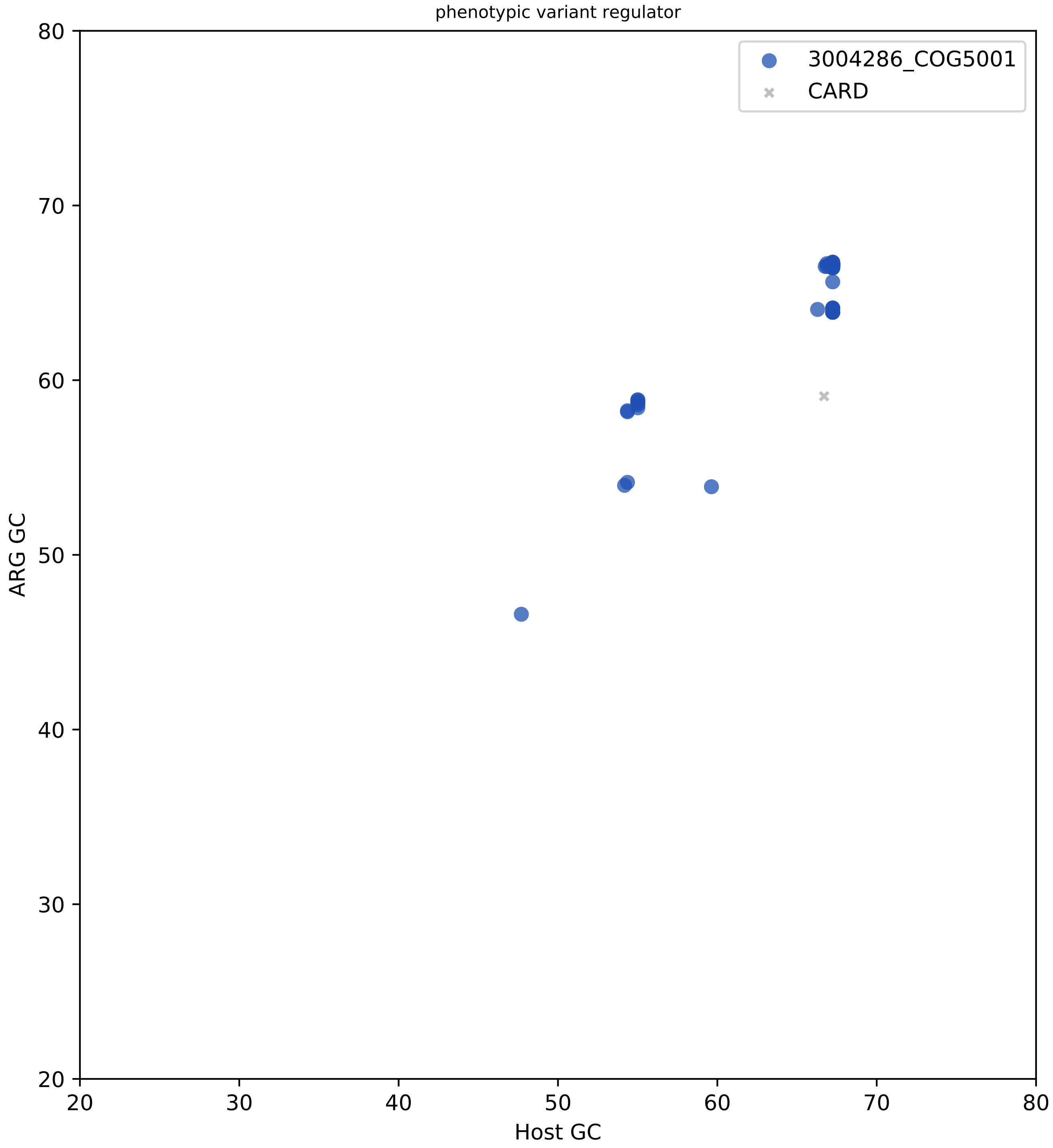

Supplementary Figure S1: (continued).
