# Supplementary material for: Systematic In Silico Assessment of Antimicrobial Resistance Dissemination across the Global Plasmidome
Source: Antibiotics (Basel). 2023 Feb 1;12(2):281. doi: 10.3390/antibiotics12020281 (PMC9951915; doi:10.3390/antibiotics12020281)
Supplement: Supplementary file 1 [file antibiotics-12-00281-s001.zip › FigureS3.pdf]

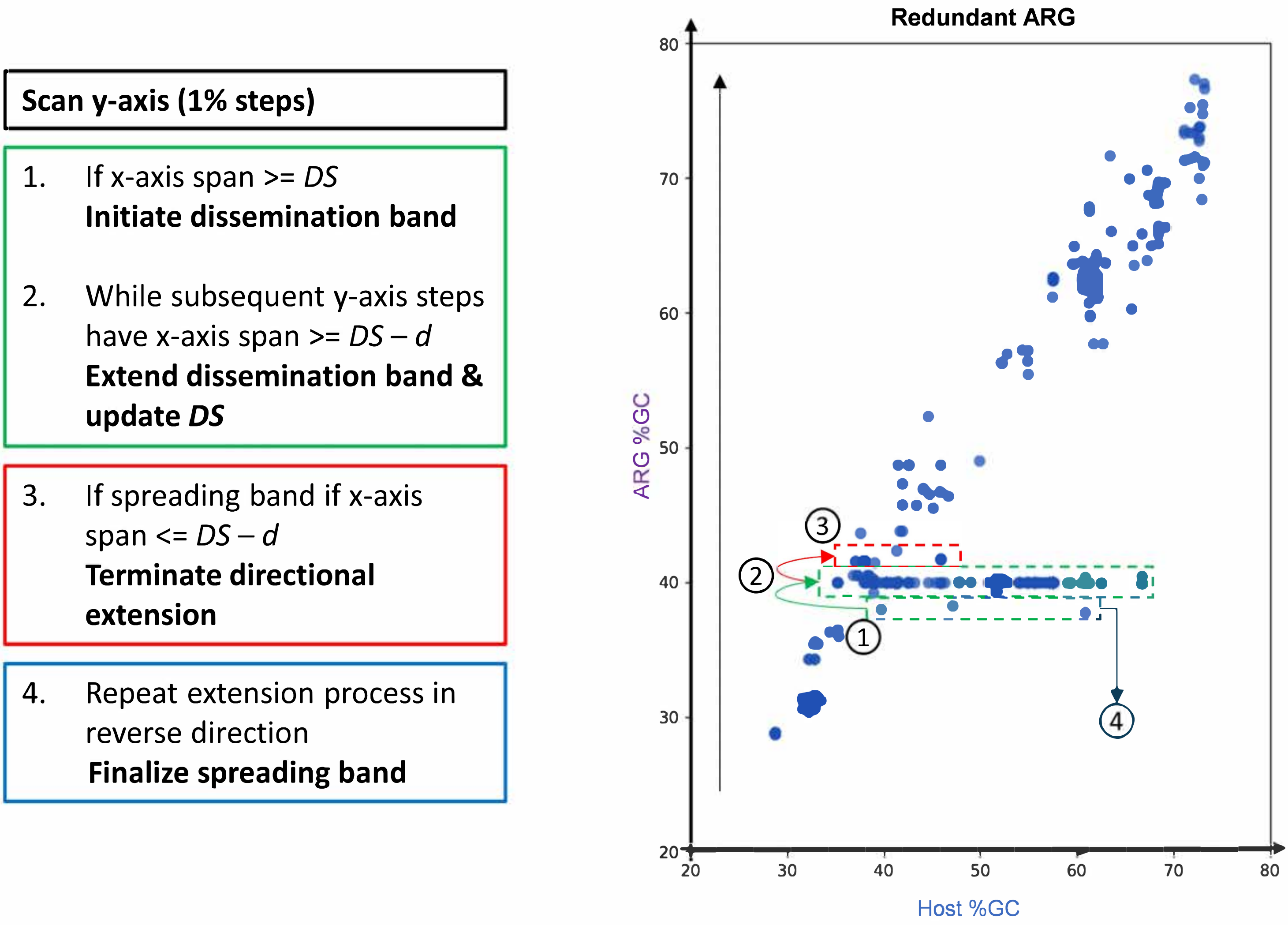

Supplementary Figure S3: Schematic diagram of the proposed methodology for dissemination band detection and dissemination index calculation.
